# Modular Generation of Alkyl Selenyl Radicals for the Synthesis of Alkyl Selenides via a Mechanochemical Approach

**DOI:** 10.1002/advs.202522609

**Published:** 2026-03-06

**Authors:** Xiaochun He, Yu Zhou, Fei Zhou, Zixi Ai, Peiyu Hu, Xuemei Zhang, Xiaofeng Wei, Gui‐Juan Cheng, Zhong Lian

**Affiliations:** ^1^ Department of Dermatology State Key Laboratory of Biotherapy and Cancer Center West China Hospital Sichuan University Chengdu P. R. China; ^2^ Warshel Institute for Computational Biology School of Medicine The Chinese University of Hong Kong Shenzhen Guangdong P. R. China; ^3^ School of Pharmacy Xi'an Jiaotong University Xi'an P. R. China

**Keywords:** alkyl selenyl radicals, elemental selenium, hydroselenation, mechanochemistry, water activation

## Abstract

Radical‐mediated selenylation has emerged as a powerful strategy for constructing carbon‐selenium bonds. While effective for generating aryl selenyl radicals, current approaches for producing alkyl selenyl radicals remain underdeveloped. In this study, we present a mechanochemical approach that enables the modular generation of alkyl selenyl radicals from elemental selenium and alkyl halides, thereby advancing the hydroselenation of alkenes. The modular generation of alkyl selenyl radicals utilizes abundant and commercially available alkyl (pseudo)halides as radical precursors, activated through mechanical force to overcome selenium's inherent inertness. The reaction proceeds under ambient conditions with minimal solvent usage, achieving a broad substrate scope encompassing alkyl iodides, bromides, chlorides, and sulfonates. Notably, mechanistic experiments and density functional theory (DFT) calculations indicate that both PhSiH_3_ or/and H_2_O can function as hydrogen atom donors through a radical pathway. This finding appears to be significant in the case of water, where the oxophilicity of silicon likely provides the driving force, pointing to a plausible silicon‐mediated activation of water. We therefore propose a mechanochemical silicon‐mediated radical process where coordination‐induced bond weakening could enable intermolecular hydrogen atom transfer (HAT) from H_2_O to carbon radicals.

## Graphical abstract

We present a mechanochemical, radical‐mediated hydroselenation of alkenes under ambient conditions. This method modularly generates alkyl selenyl radicals from elemental selenium and alkyl (pseudo)halides, utilizing PhSiH_3_ and H_2_O as hydrogen atom donors, with the water likely originating from the air and/or trace water present in the reagents.

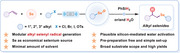

## 1 Introduction

**FIGURE 1:**
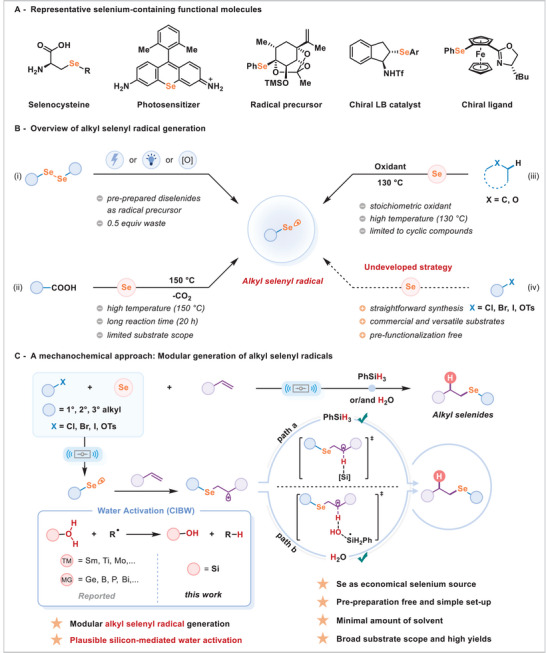
(A) Representative selenium‐containing functional molecules. (B) Overview of alkyl selenyl radical generation. (C) A Mechanochemical approach: Modular generation of alkyl selenyl radicals.

Selenium, an indispensable trace element, plays a pivotal role in biological systems through its incorporation into selenoproteins, thereby orchestrating vital functions such as antioxidant defense and metabolic regulation [1]. Beyond its biological significance, selenium stands out as a valuable byproduct of sulfide mineral processing, fueling innovation across a spectrum of domains—including pharmaceuticals, catalysis, agrochemistry, and materials science (Figure 1A) [2, 3, 4]. This expansive range of applications has spurred the development of various synthetic methodologies, among which radical‐mediated selenylation has emerged as a particularly potent strategy for the construction of C─Se bonds, offering transformative opportunities in both research and industrial contexts.

Currently, radical selenylation methodologies for the formation of organoselenium compounds predominantly rely on arylselenyl radicals, which are conventionally derived from arylselenium reagents, such as diaryl diselenides [5, 6], aryl selenocyanates [7], aryl selenols [8]. Although these strategies proficiently produce aryl selenyl radicals, they remain inadequately developed for the generation of alkyl selenyl radicals [9, 10, 11, 12, 15, 16]. The conventional approach to activating alkyl diselenides for the formation of alkyl Se‐centered radicals often requires electrochemical or photochemical conditions, or the use of external oxidants (Figure 1B(i)) [11, 12]. Furthermore, these strategies typically involve laborious pre‐synthetic procedures, including reactions of organolithium compounds or Grignard reagents with elemental selenium, oxidation of alkyl selenols, or alkylation of selenolates, and suffer from the use of polar and toxic solvents, which complicate practical applications [13, 14]. Recent work by Yu introduced an efficient approach to synthesizing organoselenium compounds via the decarboxylative selenylation of phenylacetic acid derivatives with elemental selenium (Figure 1B(ii)) [15]. However, this approach necessitates harsh reaction conditions, including elevated temperatures and extended reaction times, and is restricted to the generation of benzylic selenyl radicals. Similarly, Zhao and co‐workers demonstrated an oxidative C–H selenation of ethers or alkanes using selenium powder to form alkyl selenyl radicals (Figure 1B(iii)) [16], but this method employs stoichiometric oxidants, high temperatures, and is limited to cyclic substrates. In contrast, a more direct strategy that employs elemental selenium and commercially available alkyl halides to generate alkyl selenyl radicals in situ is highly desirable, as it circumvents the need for tedious and laborious pre‐functionalization steps (Figure 1B(iv)). Elemental selenium is particularly appealing due to its commercial availability, stability, low toxicity, and potential for environmentally benign synthesis [17, 18]. However, the practical application of selenium powder in organic synthesis is largely hindered by its chemical inertness, extremely low solubility in common solvents, and its tendency to deactivate metal catalysts.

Recently, mechanochemistry has emerged as a solvent‐minimized and energy‐efficient approach that facilitates novel and challenging transformations under solid‐state conditions [19, 20, 21, 22, 23, 24, 25, 26, 27, 28, 29, 30, 31, 32, 33, 34, 35, 36, 37, 38, 39, 40, 41, 42, 43, 44, 45, 46, 47, 48, 49, 50, 51, 52, 53, 54]. For instance, Wei described a mechanochemical method for synthesizing organoselenium compounds, utilizing elemental magnesium to activate selenium powder [55]. Inspired by this successful application of elemental selenium under grinding conditions, we have designed and implemented a mechanochemical strategy for the modular generation of alkyl selenyl radicals from cheap, bench‐stable elemental selenium and versatile alkyl (pseudo)halides. This approach enables the *anti*‐Markovnikov hydroselenation of alkenes employing phenylsilane (PhSiH_3_) or/and H_2_O (likely from atmospheric or/and reagent‐sourced moisture) as hydrogen sources (Figure 1C). Under ball‐milling conditions, alkyl halide and elemental selenium generate the key alkyl selenyl radical, which adds to the alkene to form a carbon‐centered radical. This intermediate undergoes hydroselenation through two pathways: one in which the carbon radical directly abstracts a hydrogen atom from PhSiH_3_ via Si─H coordination; the other involves H_2_O as the hydrogen source, where in situ‐generated PhSiH_2_• activates water through coordination‐induced bond weakening (CIBW) [56, 57, 58], enabling hydrogen abstraction by the carbon radical. In our studies, a variety of alkyl electrophiles, including alkyl iodides, bromides, chlorides, sulfonates, and even secondary and tertiary alkyl halides, can function as radical precursors. Notably, this method is conducted under ambient air conditions in a straightforward manner, significantly simplifying the operational procedures for the modular generation of alkyl selenyl radicals. Mechanistic studies and density functional theory (DFT) calculations elucidate the roles of PhSiH_3_ and H_2_O as hydrogen sources.

## 2 Results and Discussion

**SCHEME 1:**
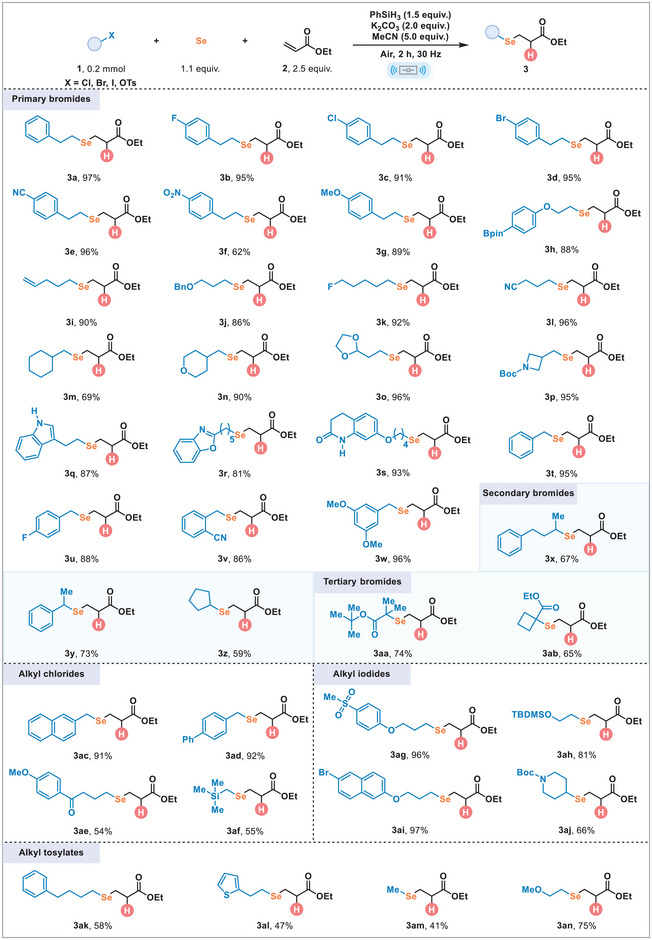
Substrate scope of alkyl electrophiles. Reaction conditions: alkyl electrophiles **1** (0.2 mmol), ethyl acrylate **2a** (2.5 equiv.), 200 mesh Se power (1.1 equiv.), PhSiH_3_ (1.5 equiv.), K_2_CO_3_ (2.0 equiv.), MeCN (5.0 equiv.), in a stainless‐steel milling jar (10.0 mL) with two stainless‐steel balls (10 mm, diameter) in air, ball milling 2 h at 30 Hz. Isolated yields.

We developed a mechanochemical approach for the synthesis of alkyl selenides using elemental selenium (Se), specifically focusing on the model reaction between (2‐bromoethyl)benzene (**1a**) and ethyl acrylate (**2a**). Optimization studies (see Tables S1–S6) revealed that effective hydroselenation required 1.0 equiv. of **1a**, 2.5 equiv. of **2a**, 1.1 equiv. of Se, 2.0 equiv. of K_2_CO_3_ as the base, 1.5 equiv. of PhSiH_3_ serving as both radical initiator and hydrogen source, and 5.0 equiv. of MeCN to facilitate liquid‐assisted grinding (LAG). The reaction was conducted in a 10 mL stainless‐steel milling jar at a frequency of 30 Hz, utilizing two stainless‐steel balls with a diameter of 10 mm. Notably, the target hydroselenation product (**3a**) was successfully obtained in an impressive isolated yield of 97%. It is remarkable that the reaction demonstrates excellent tolerance to water, either from atmospheric moisture or as an additive. Following this, we explored the scope of this reaction to assess its tolerance toward various alkyl bromides as electrophiles (Scheme 1). Alkyl bromides containing aromatic rings with diverse substituents were efficiently converted into corresponding products (**3b‐3h**) with satisfactory yields. Additionally, other primary alkyl bromides featuring remote functional groups, including alkenyl (**3i**), benzyloxy (**3j**), fluorine (**3k**), and cyanide (**3l**), demonstrated compatibility under standard reaction conditions. Substrates incorporating cyclic alkyl, as well as oxygen‐ and nitrogen‐containing heterocycles, were also successfully reacted, yielding products (**3m‐3p**) with yields ranging from 69%–96%. Furthermore, unprotected indoles (**3q**), oxazole (**3r**), and lactam (**3s**) were compatible with this transformation, underscoring the effectiveness and robustness of this method. We also evaluated the compatibility of benzyl bromides, revealing that substrates with both electron‐withdrawing and electron‐donating groups on the aromatic ring led to high yields (**3t‐3w**). Furthermore, sterically hindered secondary and tertiary alkyl bromides were tolerated, leading to the formation of linear selenides (**3x‐3ab**). Encouraged by the successful hydroselenation of ethyl acrylate (**2a**) involving alkyl bromides, we expanded our investigation to include a broader range of alkyl electrophiles in this transformation. Pleasingly, we found that an array of primary alkyl chlorides proceeded the reaction smoothly, delivering the desired products in moderate to excellent yields (**3ac‐3af**). Additionally, both primary and secondary alkyl iodides participated effectively in the hydroselenation reactions, resulting in linear selenides (**3ag‐3aj**) with yields between 66%–97%. Remarkably, alkyl tosylates (**3ak‐3an**) were also effective electrophilic reagents, achieving moderate to good yields under the optimal ball‐milling conditions.

**SCHEME 2:**
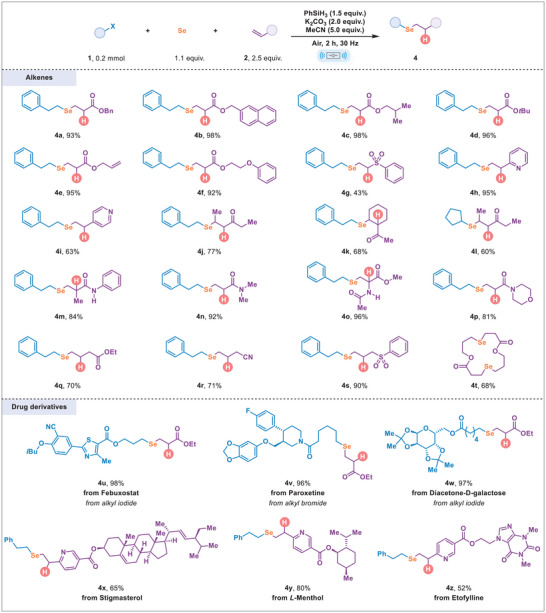
Substrate scope. Reaction conditions: alkyl halides **1** (0.20 mmol), alkene **2** (2.5 equiv.), 200 mesh Se power (1.1 equiv.), PhSiH_3_ (1.5 equiv.), K_2_CO_3_ (2.0 equiv.), MeCN (5.0 equiv.), in a stainless‐steel milling jar (10.0 mL) with two stainless‐steel balls (10 mm, diameter) in air, ball milling 2 h at 30 Hz. Isolated yields.

A comprehensive evaluation of olefin substrates for the transformation was performed (Scheme 2). A variety of *α*,*β*‐unsaturated esters yielded corresponding products with excellent yields (**4a‐4f**). Vinyl sulfone also acted as an effective substrate (**4g**). The reaction between 2‐vinylpyridine and **1a** provided product **4h** in a 95% yield, and 4‐vinylpyridine was also compatible with the reaction conditions (**4i**). Internal olefins were tolerated as well, achieving moderate yields (**4j** and **4k**). Notably, this method enabled the insertion of selenium between a secondary bromide and an internal alkene, successfully producing a sterically hindered organoselenium compound (**4l**). Additionally, products **4m‐4p** were obtained in high to excellent yields from their respective *α*,*β*‐unsaturated amides. This protocol works effectively with several unactivated alkenes, including ethyl but‐3‐enoate, 3‐butenenitrile, and allylic sulfone, all of which were successfully converted to the corresponding hydroselenation products in good yields (**4q‐4s**). Interestingly, the reaction of 3‐bromopropyl acrylate afforded a 16‐membered bis‐selenide macrocycle in 68% yield (**4t**), likely formed through an intermolecular hydroselenation followed by intramolecular cyclization. Moreover, the late‐stage modifications of drug derivatives (**4u‐4z**) demonstrated the practicality of this mechanochemical strategy for synthesizing alkyl selenides.

**FIGURE 2:**
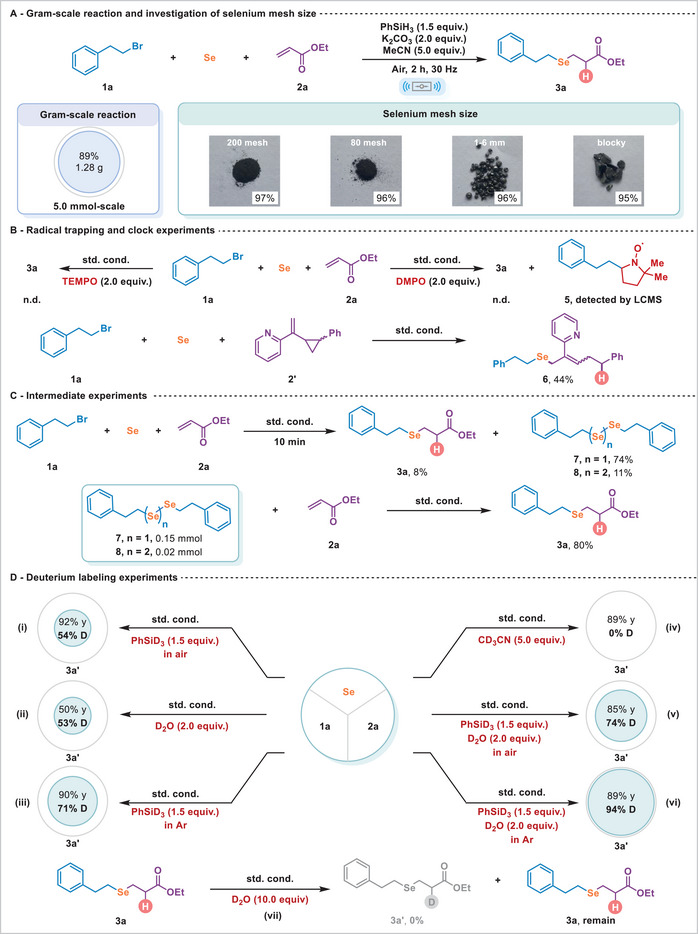
(A) Gram‐scale reaction and investigation of selenium mesh size; (B) Radical trapping and clock experiments; (C) Intermediate experiments; (D) Deuterium labeling experiments.

Subsequently, we scaled the template reaction between **1a**, **2a,** and Se powder to 5 mmol, successfully delivering the *anti*‐Markovnikov selenide product **3a** on a gram scale with a yield of 89% (Figure 2A). Further investigations involved employing elemental selenium of varying particle sizes to assess the impact on hydroselenation. Results revealed no yield dependence on particle size under standard mechanochemical conditions (Figure 2A). Notably, bulk selenium retained an excellent yield of 95%, underscoring the process's robustness against variations in selenium's physical form. Next, we discovered that the desired reaction was completely inhibited in the presence of stoichiometric amounts of radical inhibitors such as TEMPO (2,2,6,6‐tetramethylpiperidine‐1‐oxyl) and DMPO (5,5‐dimethyl‐1‐pyrroline *N*‐oxide). The detection of adduct **5** via liquid chromatography‐mass spectrometry (LCMS) under DMPO‐trapping confirmed the formation of transient radical species, while the ring‐opening of vinylcyclopropane **2'** to afford product **6** served as further evidence. These findings support a radical‐mediated mechanism for the hydroselenation reaction (Figure 2B). Following a 10‐min template reaction, the reaction was quenched, leading to the isolation of target compound **3a** (8% yield), along with two intermediates, symmetric diselenides (**7**, 75% yield) and triselenides (**8**, 11% yield) from the resulting mixture (Figure 2C). The mixture of **7** and **8,** when subjected to standard conditions, effectively converted to **3a** with an 80% yield. These results indicate that hydroselenation proceeds via a radical pathway involving an alkyl selenium.

To further elucidate the transformation, we performed a series of deuterium‐labeling studies (Figure 2D). Substituting PhSiH_3_ with PhSiD_3_ to the standard conditions (under air) afforded the deuterated product **3a'** in 92% yield with 54% deuterium incorporation (Figure 2D(i)). The comparable deuterium incorporation (54%) observed upon addition of 2.0 equiv. of D_2_O suggests that the water, likely from the air and/or trace water present in the reagents, can also serve as a hydrogen source (Figure 2D(ii)). This interpretation is further supported by performing the reaction under an argon atmosphere using PhSiD_3_, which increased deuterium incorporation to 71% (Figure 2D(iii)). Replacing MeCN with CD_3_CN resulted in no deuterium incorporation, ruling out acetonitrile as a hydrogen donor (Figure 2D(iv)). Moreover, the reaction affords essentially the analogous yields and deuterium incorporation of **3a'** with 2 equiv. of D_2_O (85% yield, 74% D) and in the experiment performed under dry argon (90% yield, 71% D) (Figure 2D(v)), demonstrating that water is not essential for achieving high reaction efficiency. Notably, employing PhSiD_3_ together with D_2_O under argon gave a deuteration efficiency of 94%, confirming that the hydrogen atoms incorporated into the C═C bond originate from both PhSiH_3_ and H_2_O (Figure 2D(vi)). While DFT calculations suggest that a proton exchange between PhSiH_3_ and H_2_O cannot be completely ruled out, the absence of H/D exchange between the silane and D_2_O under ball‐milling conditions does not support a hydrogen exchange between water and silane in this system (see Supporting Information). Finally, subjecting isolated **3a** to D_2_O under standard conditions yielded no detectable **3a'**, excluding a post‑synthetic H/D exchange under basic conditions as a possible pathway (Figure 2D(vii)).

**FIGURE 3:**
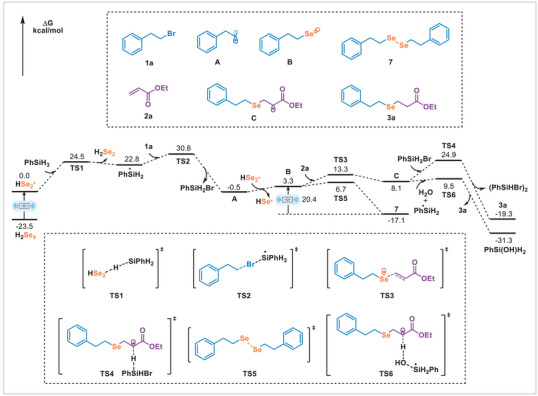
DFT calculations.

Control experiments established the essential role of mechanical force in this transformation. In both solution‐phase (conventional stirring in MeCN) and neat conditions without ball milling, the reaction afforded only trace amounts of the desired product **3a** (seeTable S6). In addition, the product yield increased with the milling frequency (Table S5). These results confirm that ball milling is essential for driving this transformation efficiently. Furthermore, a parallel experiment using a ZrO_2_ jar yielded a result comparable to that obtained with a stainless‐steel jar (Table S7), ruling out catalytic effects from the abrasion of trace metals. Guided by these experimental observations, we performed DFT calculations to investigate the detailed mechanism (Figure 3). Mechanical force is proposed to induce homolytic cleavage of the Se─Se bond in polymeric solid selenium, generating selenium‐centered radicals. Subsequent hydrogen atom transfer from PhSiH_3_ to the resulting selenium‐centered radicals affords the silyl radical. Using a simplified model comprising four Se atoms (H‐capped termini), the Se─Se bond homolysis was calculated to be endothermic by 23.5 kcal/mol. The resulting radical abstracts a hydrogen atom from PhSiH_3_ to form the silyl radical, with an activation barrier of 24.5 kcal/mol. This nucleophilic silyl radical can easily abstract a Br atom from (2‐bromoethyl)benzene (**1a**) to generate the radical intermediate **A**. The barrier for this process is 7.8 kcal/mol. Subsequently, intermediate **A** adds with the selenium radical to form alkyl selenyl radical **B**, which can easily dimerize to form symmetrical diselenium **7**. Under the ball‐milling conditions, intermediate **7** can break the Se─Se bond to regenerate selenyl radical **B**, which undergoes radical addition with ethyl acrylate (**2a**) to form intermediate **C**, with an activation energy of 10.0 kcal/mol for this step. Intermediate **C** then abstracts a hydrogen atom from PhSiH_3_ via the transition state **TS4**, leading to the formation of **3a**. Alternatively, intermediate **C** could abstract a hydrogen atom from one water molecule via a concerted pathway, which proceeds through the transition state **TS6** with an activation barrier of 1.5 kcal/mol. The computational results are consistent with the deuterium labeling experiments that the hydrogen of the final product could originate from both PhSiH_3_ and H_2_O.

## 3 Conclusions

In conclusion, we have developed a mechanochemical strategy for the direct and efficient generation of alkyl selenyl radicals from elemental selenium and readily available alkyl (pseudo)halides. This method employs hydroselenation of olefins using PhSiH_3_ and/or H_2_O as hydrogen atom donors. This radical‐mediated method operates under ambient air conditions, significantly simplifying operations and facilitating scalability. The transformation not only demonstrates substantial compatibility with a range of functional groups but also accommodates various types of alkyl electrophilic reagents, including alkyl bromides, alkyl iodides, alkyl chlorides, and alkyl sulfonates. Mechanistic studies elucidate that both PhSiH_3_ and H_2_O can serve as hydrogen atom donors in a radical process and establish a plausible mechanism for the activation of water. We anticipate that this method for synthesizing organoselenides will have practical applications in the field of medicinal chemistry.

## Conflicts of Interest

The authors declare no conflicts of interest.

## Supporting information




**Supporting File**: advs74730‐sup‐0001‐SuppMat.pdf

## Data Availability

The data that support the findings of this study are available in the supplementary material of this article.
