# Supplementary material for: Modular Generation of Alkyl Selenyl Radicals for the Synthesis of Alkyl Selenides via a Mechanochemical Approach
Source: Adv Sci (Weinh). 2026 Mar 6;13(28):e22609. doi: 10.1002/advs.202522609 (PMC13185885; doi:10.1002/advs.202522609)

Supporting Information

Modular Generation of Alkyl Selenyl Radicals for the Synthesis of Alkyl Selenides *via* a Mechanochemical Approach

Xiaochun He,[a]† Yu Zhou,[b]† Fei Zhou,[a]† Zixi Ai,[a] Peiyu Hu,[a] Xuemei Zhang,[a]* Xiaofeng Wei,[c]* Gui-Juan Cheng,[b]* Zhong Lian[a]*

Contents

1. General information........ S2
2. Optimization of reaction conditions S2
3. General procedure for the hydroselenation of olefins S5
4. Gram-scale experiment........ S5
5. Characterization data of products 3-4........ S5
6. Mechanistic experiments........ S18
7. DFT calculations S26
8. References S33
9. NMR spectra........ S34

1. General information

Unless otherwise noted, all reagents were obtained from commercial suppliers and used as received. Key materials include: selenium powder (Trigonal selenium (gray selenium), 98% metals basis, 200 mesh, Energy Chemical), phenylsilane (98%), ethyl acrylate (98%), (2-bromoethyl)benzene (98%), potassium carbonate (99%), and acetonitrile (AR grade). All reactions were performed using grinding vessels in Gladman vibration ball mill GT300. Both jars and balls were made of stainless steel. All work-up and purification procedures were carried out with reagent-grade solvents in air. Thin layer chromatography analysis was carried out on silica gel coated glass plates (0.25 mm) with fluorescence indicator UV254. Flash column chromatography was performed with silica gel 60 (particle size 200-300 mesh, Huanghai) at room temperature and under elevated pressure. Gas chromatography (GC) analysis was conducted on a Shimadzu GC-2030 instrument equipped with a Rtx-5 column (30 m × 0.25 mm) with dodecane as an internal standard. ^{1}H, ^{19}F, ^{13}C NMR spectra were recorded in Chloroform-*d* on Bruker Avance 400 MHz spectrometers. High-resolution mass spectrometric measurements (HRMS) were recorded on SHIMADZU LCMS-IT-TOF mass spectrometer. The molecular ion $[M+H]^+$, $[M+Na]^+$ and $[M+K]^+$ are given in m/z units.

2. Optimization of reaction conditions

Table S1. Optimization of base [a]

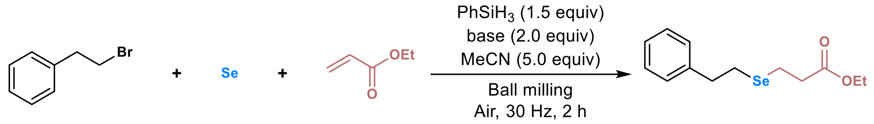

1a, 0.2 mmol 1.1 equiv **2a**, 2.5 equiv **3a**

Entry	Base	Yield (%)
1	Cs_2CO_3	23
2	CsF	27
3	Et_3N	n.d.
4	K_3PO_4	89
5	K_2CO_3	97

Reaction conditions: [a] (2-bromoethyl)benzene **1a** (0.2 mmol, 1.0 equiv), ethyl acrylate **2a** (2.5 equiv), 200 mesh Se power (1.1 equiv), $PhSiH_3$ (1.5 equiv), Base (2.0 equiv), MeCN (5.0 equiv), in a stainless-steel milling jar (10.0 mL) with two stainless-steel balls (10 mm, diameter) in air, ball milling 2 h at 30 Hz, the yields were determined by gas chromatography (GC) analysis of the crude reaction mixture using dodecane as an internal standard.

Table S2. Optimization of selenium amount [a]

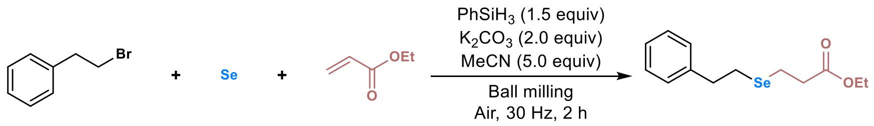

1a, 0.2 mmol x equiv **2a**, 2.5 equiv **3a**

Entry	Se (equiv)	Yield (%)
1	1.1	97

2	1.3	93
3	1.5	90

Reaction conditions: [a] (2-bromoethyl)benzene **1a** (0.2 mmol, 1.0 equiv), ethyl acrylate **2a** (2.5 equiv), 200 mesh Se power (x equiv), $PhSiH_3$ (1.5 equiv), K_2CO_3 (2.0 equiv), MeCN (5.0 equiv), in a stainless-steel milling jar (10.0 mL) with two stainless-steel balls (10 mm, diameter) in air, ball milling 2 h at 30 Hz, the yields were determined by gas chromatography (GC) analysis of the crude reaction mixture using dodecane as an internal standard.

Table S3. Optimization of $PhSiH_3$ amount [a]

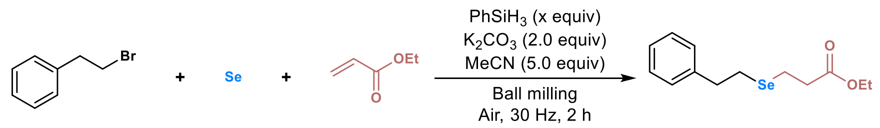

Entry	$PhSiH_3$ (equiv)	Yield (%)
1	0	n.d.
2	0.5	2
3	1.0	50
4	1.5	97

Reaction conditions: [a] (2-bromoethyl)benzene **1a** (0.2 mmol, 1.0 equiv), ethyl acrylate **2a** (2.5 equiv), 200 mesh Se power (1.1 equiv), $PhSiH_3$ (x equiv), K_2CO_3 (2.0 equiv), MeCN (5.0 equiv), in a stainless-steel milling jar (10.0 mL) with two stainless-steel balls (10 mm, diameter) in air, ball milling 2 h at 30 Hz, the yields were determined by gas chromatography (GC) analysis of the crude reaction mixture using dodecane as an internal standard.

Table S4. Optimization of acetonitrile amount [a]

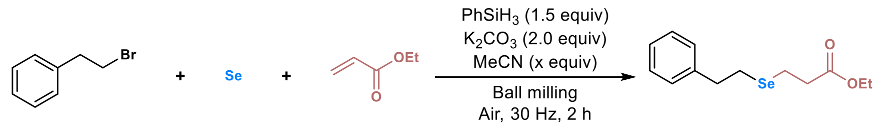

Entry	MeCN (equiv)	Yield (%)
1	5.0	97
2	3.0	73
3	2.0	70
4	1.0	63

Reaction conditions: [a] (2-bromoethyl)benzene **1a** (0.2 mmol, 1.0 equiv), ethyl acrylate **2a** (2.5 equiv), 200 mesh Se power (1.1 equiv), $PhSiH_3$ (1.5 equiv), K_2CO_3 (2.0 equiv), MeCN (x equiv), in a stainless-steel milling jar (10.0 mL) with two stainless-steel balls (10 mm, diameter) in air, ball milling 2 h at 30 Hz, the yields were determined by gas chromatography (GC) analysis of the crude reaction mixture using dodecane as an internal standard.

Table S5. Optimization of frequency [a]

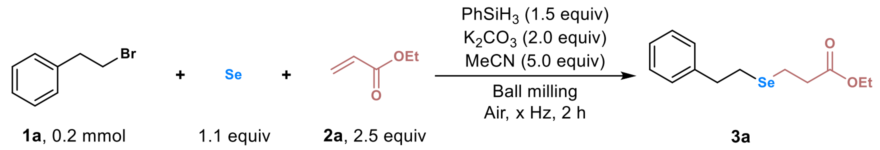

Entry	Frequency (Hz)	Yield (%)
1	5	25
2	10	43
3	20	90
4	30	97

Reaction conditions: [a] (2-bromoethyl)benzene **1a** (0.2 mmol, 1.0 equiv), ethyl acrylate **2a** (2.5 equiv), 200 mesh Se power (1.1 equiv), $PhSiH_3$ (1.5 equiv), K_2CO_3 (2.0 equiv), MeCN (5.0 equiv), in a stainless-steel milling jar (10.0 mL) with two stainless-steel balls (10 mm, diameter) in air, ball milling 2 h, the yields were determined by gas chromatography (GC) analysis of the crude reaction mixture using dodecane as an internal standard.

Table S6. Cntrol experiments in solution and neat conditions [a]

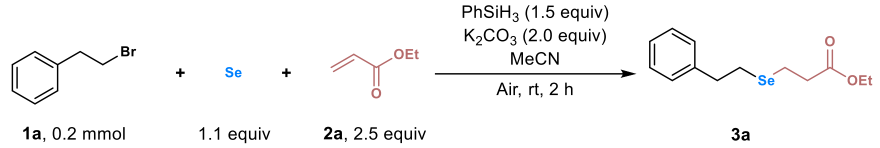

Entry	Variation from standard conditions	Yield (%)
1	MeCN (1.0 mL)	trace
2	Stirring neat	trace

Reaction conditions: [a] (2-bromoethyl)benzene **1a** (0.2 mmol, 1.0 equiv), ethyl acrylate **2a** (2.5 equiv), 200 mesh Se power (1.1 equiv), $PhSiH_3$ (1.5 equiv), K_2CO_3 (2.0 equiv), MeCN, in air, 2 h at rt, the yields were determined by gas chromatography (GC) analysis of the crude reaction mixture using dodecane as an internal standard.

Table S7. Optimization of vessel materials [a]

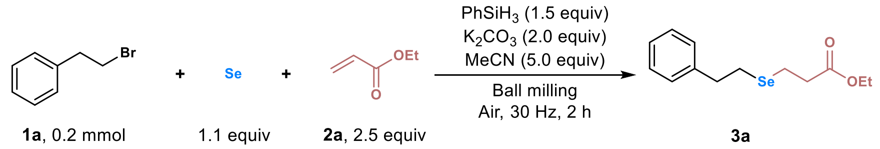

Entry	Vessel materials	Yield (%)
1	Stainless-steel jar	97
2	ZrO_2 jar	92

Reaction conditions: [a] (2-bromoethyl)benzene **1a** (0.2 mmol, 1.0 equiv), ethyl acrylate **2a** (2.5 equiv), 200 mesh Se power (1.1 equiv), $PhSiH_3$ (1.5 equiv), K_2CO_3 (2.0 equiv), MeCN (5.0 equiv), in a jar (10.0 mL) with two stainless-steel balls (10 mm, diameter) in air, ball milling 2 h at 30 Hz, the yields were determined by gas chromatography (GC) analysis of the crude reaction mixture using dodecane as an internal standard.

3. General procedure for the hydroselenation of olefins

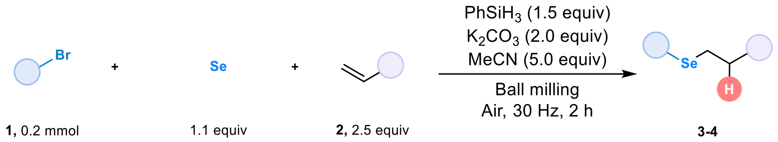

Alkyl electrophiles (-Cl/-Br/-I/-OTs) (0.2 mmol), 200 mesh selenium powder (0.22 mmol), K_2CO_3 (0.4 mmol), MeCN (1.0 mmol) and alkenes (0.5 mmol) were combined in a stainless-steel milling jar (10.0 mL) containing two stainless-steel balls (10 mm diameter) under an air atmosphere. Subsequently, $PhSiH_3$ (0.3 mmol) was added all at once. After sealing the jar in air, it was subjected to grinding at 30 Hz for 2 h to facilitate the reaction. The reaction was then quenched with ethyl acetate, after which the mixture was filtered. The resulting filtrate was concentrated, and the residue was purified by flash silica gel column chromatography, using a petroleum ether/ethyl acetate mixture as the eluent, to yield pure products **3-4**.

4. Gram-scale experiment

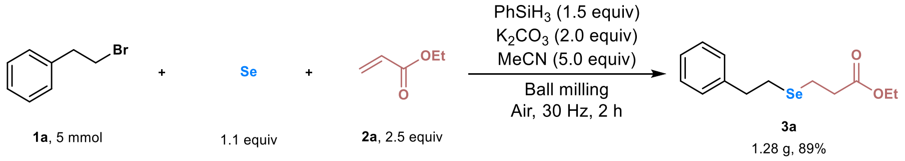

(2-bromoethyl)benzene **1a** (5.0 mmol, 1.0 equiv), 200 mesh Se powder (5.5 mmol, 1.1 equiv), K_2CO_3 (10 mmol, 2.0 equiv), MeCN (25 mmol, 25 equiv) and ethyl acrylate **2a** (12.5 mmol, 2.5 equiv), were combined in a stainless-steel milling jar (50.0 mL) containing six stainless-steel balls (10 mm diameter) under an air atmosphere. $PhSiH_3$ (7.5 mmol, 1.5 equiv) was added all at once. After sealing the jar in air, it was subjected to grinding at 30 Hz for 2 h to facilitate the reaction. The reaction was then quenched with ethyl acetate, after which the mixture was filtered. The resulting filtrate was concentrated, and the residue was purified by flash silica gel column chromatography, using a petroleum ether/ethyl acetate 20:1 as the eluent, to yield the product **3a** (1.28g, 89%).

5. Characterization data of products 3-4

ethyl 3-(phenethylselanyl)propanoate (3a)

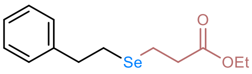

Prepared by general procedure; isolated as a colorless oil using petroleum/ethyl acetate (20:1) as eluent. (55.5 mg, 97%). **^{1}H NMR** (400 MHz, Chloroform-*d*) δ 7.34 – 7.27 (m, 2H), 7.26 – 7.16 (m, 3H), 4.16 (q, *J* = 7.2 Hz, 2H), 3.02 – 2.93 (m, 2H), 2.88 – 2.81 (m, 2H), 2.82 – 2.73 (m, 2H), 2.73 – 2.65 (m, 2H), 1.27 (t, *J* = 7.2 Hz, 3H). **^{13}C NMR** (101 MHz, Chloroform-*d*) δ 172.2, 141.1, 128.5, 128.3, 126.3, 60.6, 37.1, 35.7, 25.2, 17.7, 14.2. **HRMS (ESI-TOF)** *m/z*: Calcd for $C_{13}H_{19}O_2Se$ $[M+H]^+$: 287.0545; found 287.0545.

ethyl 3-((4-fluorophenethyl)selanyl)propanoate (3b)

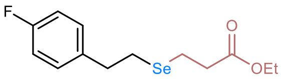

Prepared by general procedure; isolated as a colorless oil using petroleum/ethyl acetate (20:1) as eluent. (57.8 mg, 95%). **^{1}H NMR** (400 MHz, Chloroform-*d*) δ 7.18 – 7.12 (m, 2H), 7.00 – 6.93 (m, 2H), 4.15 (q, *J* = 7.2 Hz, 2H), 2.98 – 2.89 (m, 2H), 2.84 – 2.73 (m, 4H), 2.71 – 2.64 (m, 2H), 1.26 (t, *J* = 7.2 Hz, 3H).

^{13}**C NMR** (101 MHz, Chloroform-*d*) δ 172.3, 161.7 (d, *J* = 244.4 Hz), 136.8 (d, *J* = 3.4 Hz), 129.9 (d, *J* = 7.8 Hz), 115.4 (d, *J* = 21.2 Hz), 60.8, 36.3, 35.9, 25.5, 17.9, 14.3. ^{19}**F NMR** (376 MHz, Chloroform-*d*) δ -116.76. **HRMS (ESI-TOF)** *m/z*: Calcd for $C_{13}H_{18}FO_2Se$ $[M+H]^+$: 305.0451; found 305.0450.

ethyl 3-((4-chlorophenethyl)selanyl)propanoate (3c)

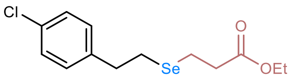

Prepared by general procedure; isolated as a colorless oil using petroleum/ethyl acetate (20:1) as eluent. (58.2 mg, 91%). ^{1}**H NMR** (400 MHz, Chloroform-*d*) δ 7.30 – 7.23 (m, 2H), 7.16 – 7.12 (m, 2H), 4.16 (q, *J* = 7.2 Hz, 2H), 2.98 – 2.91 (m, 2H), 2.85 – 2.74 (m, 4H), 2.72 – 2.66 (m, 2H), 1.27 (t, *J* = 7.2 Hz, 3H). ^{13}**C NMR** (101 MHz, Chloroform-*d*) δ 172.3, 139.6, 132.2, 129.8, 128.7, 60.8, 36.4, 35.8, 25.1, 17.9, 14.3. **HRMS (ESI-TOF)** *m/z*: Calcd for $C_{13}H_{18}ClO_2Se$ $[M+H]^+$: 321.0155; found 321.0153.

ethyl 3-((4-bromophenethyl)selanyl)propanoate (3d)

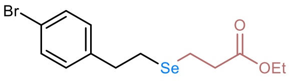

Prepared by general procedure; isolated as a white solid using petroleum/ethyl acetate (20:1) as eluent. (69.2 mg, 95%). ^{1}**H NMR** (400 MHz, Chloroform-*d*) δ 7.44 – 7.39 (m, 2H), 7.10 – 7.05 (m, 2H), 4.15 (q, *J* = 7.2 Hz, 2H), 2.96 – 2.88 (m, 2H), 2.84 – 2.73 (m, 4H), 2.70 – 2.64 (m, 2H), 1.26 (t, *J* = 7.2 Hz, 3H). ^{13}**C NMR** (101 MHz, Chloroform-*d*) δ 172.3, 140.1, 131.7, 130.3, 120.3, 60.8, 36.5, 35.9, 25.1, 18.0, 14.3. **HRMS (ESI-TOF)** *m/z*: Calcd for $C_{13}H_{18}BrO_2Se$ $[M+H]^+$: 364.9650; found 364.9644.

ethyl 3-((4-cyanophenethyl)selanyl)propanoate (3e)

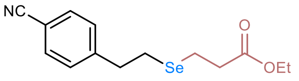

Prepared by general procedure; isolated as a yellow solid using petroleum/ethyl acetate (10:1) as eluent. (59.7 mg, 96%). ^{1}**H NMR** (400 MHz, Chloroform-*d*) δ 7.61 – 7.54 (m, 2H), 7.34 – 7.28 (m, 2H), 4.14 (q, *J* = 7.2 Hz, 2H), 3.07 – 2.98 (m, 2H), 2.86 – 2.80 (m, 2H), 2.79 – 2.73 (m, 2H), 2.70 – 2.65 (m, 2H), 1.25 (t, *J* = 7.2 Hz, 3H). ^{13}**C NMR** (101 MHz, Chloroform-*d*) δ 172.3, 146.6, 132.4, 129.4, 119.0, 110.4, 60.8, 37.1, 35.8, 24.4, 18.0, 14.3. **HRMS (ESI-TOF)** *m/z*: Calcd for $C_{14}H_{18}NO_2Se$ $[M+H]^+$: 312.0497; found 312.0494.

ethyl 3-((4-nitrophenethyl)selanyl)propanoate (3f)

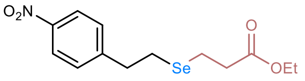

Prepared by general procedure; isolated as a yellow oil using petroleum/ethyl acetate (10:1) as eluent. (41.0 mg, 62%). ^{1}**H NMR** (400 MHz, Chloroform-*d*) δ 8.18 – 8.11 (m, 2H), 7.40 – 7.33 (m, 2H), 4.14 (q, *J* = 7.2 Hz, 2H), 3.08 (t, *J* = 7.8 Hz, 2H), 2.91 – 2.82 (m, 2H), 2.82 – 2.74 (m, 2H), 2.73 – 2.65 (m, 2H), 1.25 (t, *J* = 7.2 Hz, 3H). ^{13}**C NMR** (101 MHz, Chloroform-*d*) δ 172.3, 148.7, 146.8, 129.4, 123.9, 60.9, 36.8, 35.8, 24.4, 18.1, 14.3. **HRMS (ESI-TOF)** *m/z*: Calcd for $C_{13}H_{18}NO_4Se$ $[M+H]^+$: 332.0396; found 332.0397.

ethyl 3-((4-methoxyphenethyl) selanyl) propanoate (3g)

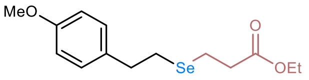

Prepared by general procedure; isolated as a colorless oil using petroleum/ethyl acetate (10:1) as eluent. (56.3 mg, 89%). ^{1}**H NMR** (400 MHz, Chloroform-*d*) δ 7.15 – 7.08 (m, 2H), 6.86 – 6.80 (m, 2H), 4.15 (q, *J* = 7.2 Hz, 2H), 3.78 (s, 3H), 2.95 – 2.88 (m, 2H), 2.85 – 2.73 (m, 4H), 2.71 – 2.65 (m, 2H), 1.26 (t, *J* = 7.2 Hz, 3H). ^{13}**C NMR** (101 MHz, Chloroform-*d*) δ 172.4, 158.3, 133.3, 129.4, 114.0, 60.8, 55.3, 36.3, 35.9, 25.7, 17.8, 14.3. **HRMS (ESI-TOF)** *m/z*: Calcd for $C_{14}H_{21}O_3Se$ $[M+H]^+$: 317.0650; found 317.0649.

ethyl 3-((2-(4-(4,4,5,5-tetramethyl-1,3,2-dioxaborolan-2-yl)phenoxy)ethyl)selanyl)propanoate (3h)

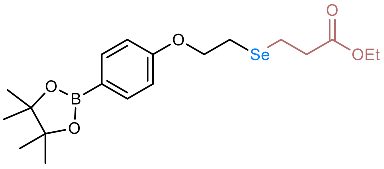

Prepared by general procedure; isolated as a white solid using petroleum/ethyl acetate (15:1) as eluent. (75.5 mg, 88%). **^{1}H NMR** (400 MHz, Chloroform-*d*) δ 7.72 (d, *J* = 8.6 Hz, 2H), 6.86 (d, *J* = 8.6 Hz, 2H), 4.22 (t, *J* = 7.0 Hz, 2H), 4.14 (q, *J* = 7.0 Hz, 2H), 2.96 – 2.84 (m, 4H), 2.78 – 2.71 (m, 2H), 1.31 (s, 12H), 1.25 (t, *J* = 7.0 Hz, 3H). **^{13}C NMR** (101 MHz, Chloroform-*d*) δ 172.3, 161.1, 136.7, 114.0, 83.7, 68.3, 60.9, 35.9, 25.0, 22.5, 18.5, 14.3. **HRMS (ESI-TOF)** *m/z*: Calcd for $C_{19}H_{30}BO_5Se$ $[M+H]^+$: 429.1346; found 429.1350.

ethyl 3-(pent-4-en-1-ylselanyl)propanoate (3i)

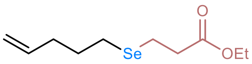

Prepared by general procedure; isolated as a colorless oil using petroleum/ethyl acetate (20:1) as eluent. (45.0 mg, 90%). **^{1}H NMR** (400 MHz, Chloroform-*d*) δ 5.78 (ddt, *J* = 17.0, 10.2, 6.8 Hz, 1H), 5.09 – 4.95 (m, 2H), 4.15 (q, *J* = 7.2 Hz, 2H), 2.81 – 2.74 (m, 2H), 2.74 – 2.66 (m, 2H), 2.63 – 2.55 (m, 2H), 2.20 – 2.10 (m, 2H), 1.80 – 1.70 (m, 2H), 1.27 (t, *J* = 7.2 Hz, 3H). **^{13}C NMR** (101 MHz, Chloroform-*d*) δ 172.5, 137.8, 115.4, 60.8, 36.0, 33.9, 29.8, 23.7, 17.6, 14.4. **HRMS (ESI-TOF)** *m/z*: Calcd for $C_{10}H_{19}O_2Se$ $[M+H]^+$: 251.0545; found 251.0542.

ethyl 3-((3-(benzyloxy)propyl)selanyl)propanoate(3j)

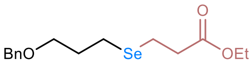

Prepared by general procedure; isolated as a colorless oil using petroleum/ethyl acetate (20:1) as eluent. (56.8 mg, 86%). **^{1}H NMR** (400 MHz, Chloroform-*d*) δ 7.37 – 7.31 (m, 4H), 7.31 – 7.26 (m, 1H), 4.50 (s, 2H), 4.15 (q, *J* = 7.2 Hz, 2H), 3.55 (t, *J* = 6.2 Hz, 2H), 2.80 – 2.75 (m, 2H), 2.73 – 2.65 (m, 4H), 2.01 – 1.92 (m, 2H), 1.26 (t, *J* = 7.2 Hz, 3H). **^{13}C NMR** (101 MHz, Chloroform-*d*) δ 172.5, 138.5, 128.5, 127.8, 127.7, 73.1, 69.6, 60.8, 35.9, 30.7, 20.9, 17.7, 14.3. **HRMS (ESI-TOF)** *m/z*: Calcd $C_{15}H_{23}O_3Se$ for $[M+H]^+$: 331.0807; found 331.0808.

ethyl 3-((5-fluoropentyl)selanyl)propanoate(3k)

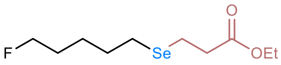

Prepared by general procedure; isolated as a yellow oil using petroleum/ethyl acetate (20:1) as eluent. (49.7 mg, 92%). **^{1}H NMR** (400 MHz, Chloroform-*d*) δ 4.48 (t, *J* = 6.2 Hz, 1H), 4.36 (t, *J* = 6.0 Hz, 1H), 4.14 (q, *J* = 7.1 Hz, 2H), 2.81 – 2.72 (m, 2H), 2.72 – 2.64 (m, 2H), 2.58 (t, *J* = 7.4 Hz, 2H), 1.81 – 1.60 (m, 4H), 1.54 – 1.42 (m, 2H), 1.25 (t, *J* = 7.2 Hz, 3H). **^{13}C NMR** (101 MHz, Chloroform-*d*) δ 172.4, 84.0 (d, *J* = 164.8 Hz), 60.8, 35.9, 30.2, 30.0 (d, *J* = 19.8 Hz), 25.6 (d, *J* = 5.2 Hz), 24.1, 17.6, 14.3. **^{19}F NMR** (376 MHz, Chloroform-*d*) δ -218.16. **HRMS (ESI-TOF)** *m/z*: Calcd for $C_{10}H_{20}FO_2Se$ $[M+H]^+$: 271.0607; found 271.0608.

ethyl 3-((3-cyanopropyl)selanyl)propanoate(3l)

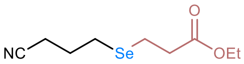

Prepared by general procedure; isolated as a yellow oil using petroleum/ethyl acetate (10:1) as eluent. (47.8 mg, 96%). **^{1}H NMR** (400 MHz, Chloroform-*d*) δ 4.15 (q, *J* = 7.2 Hz, 2H), 2.83 – 2.76 (m, 2H), 2.74 – 2.66 (m, 4H), 2.50 (t, *J* = 7.0 Hz, 2H), 2.00 (p, *J* = 7.2 Hz, 2H), 1.26 (t, *J* = 7.2 Hz, 3H). **^{13}C NMR** (101 MHz, Chloroform-*d*) δ 172.2, 119.1, 60.9, 35.7, 26.1, 22.5, 18.0, 17.2, 14.3. **HRMS (ESI-TOF)** *m/z*: Calcd $C_9H_{16}NO_2Se$ for $[M+H]^+$: 250.0341; found 250.0334.

ethyl 3-((cyclohexylmethyl)selanyl)propanoate(3m)

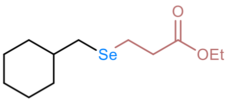

Prepared by general procedure; isolated as a colorless oil using petroleum/ethyl acetate (20:1) as eluent. (38.4 mg, 69%). **^{1}H NMR** (400 MHz, Chloroform-*d*) δ 4.15 (q, *J* = 7.2 Hz, 2H), 2.81 – 2.71 (m, 2H), 2.71 – 2.64 (m, 2H), 2.49 (d, *J* =

7.0 Hz, 2H), 1.87 – 1.77 (m, 2H), 1.74 – 1.67 (m, 2H), 1.67 – 1.60 (m, 1H), 1.51 – 1.39 (m, 1H), 1.33 – 1.06 (m, 6H), 1.00 – 0.87 (m, 2H). **^{13}C NMR** (101 MHz, Chloroform-*d*) δ 172.5, 60.7, 38.7, 36.0, 33.6, 32.8, 26.4, 26.2, 18.2, 14.3. **HRMS (ESI-TOF)** *m/z*: Calcd $C_{12}H_{23}O_2Se$ for $[M+H]^+$: 279.0858; found 279.0856.

ethyl 3-(((tetrahydro-2H-pyran-4-yl)methyl)selanyl)propanoate(3n)

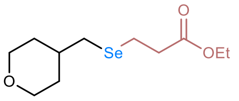

Prepared by general procedure; isolated as a colorless oil using petroleum/ethyl acetate (10:1) as eluent. (50.4 mg, 90%). **^{1}H NMR** (400 MHz, Chloroform-*d*) δ 4.15 (q, *J* = 7.2 Hz, 2H), 4.00 – 3.93 (m, 2H), 3.41 – 3.30 (m, 2H), 2.80 – 2.73 (m, 2H), 2.72 – 2.65 (m, 2H), 2.53 (d, *J* = 6.8 Hz, 2H), 1.78 – 1.64 (m, 3H), 1.36 – 1.23 (m, 5H). **^{13}C NMR** (101 MHz, Chloroform-*d*) δ 172.4, 68.0, 60.8, 36.2, 35.9, 33.4, 31.8, 18.3, 14.4. **HRMS (ESI-TOF)** *m/z*: Calcd $C_{11}H_{21}O_3Se$ for $[M+H]^+$: 281.0650; found 281.0649.

ethyl 3-((2-(1,3-dioxolan-2-yl)ethyl)selanyl)propanoate(3o)

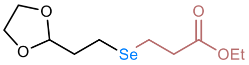

Prepared by general procedure; isolated as a colorless oil using petroleum/ethyl acetate (15:1) as eluent. (54.2 mg, 96%). **^{1}H NMR** (400 MHz, Chloroform-*d*) δ 4.93 (t, *J* = 4.6 Hz, 1H), 4.14 (q, *J* = 7.2 Hz, 2H), 4.00 – 3.80 (m, 4H), 2.78 (t, *J* = 7.0 Hz, 2H), 2.74 – 2.60 (m, 4H), 2.02 (td, *J* = 7.8, 4.6 Hz, 2H), 1.25 (t, *J* = 7.2 Hz, 3H). **^{13}C NMR** (101 MHz, Chloroform-*d*) δ 172.4, 103.8, 65.1, 60.8, 35.8, 35.0, 17.9, 17.7, 14.3. **HRMS (ESI-TOF)** *m/z*: Calcd $C_{10}H_{19}O_4Se$ for $[M+H]^+$: 283.0443; found 283.0444.

***tert*-butyl 3-(((3-ethoxy-3-oxopropyl)selanyl)methyl)azetidine-1-carboxylate(3p)**

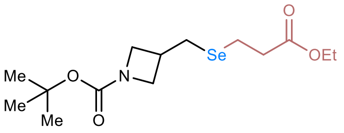

Prepared by general procedure; isolated as a white solid using petroleum/ethyl acetate (10:1) as eluent. (66.7 mg, 95%). **^{1}H NMR** (400 MHz, Chloroform-*d*) δ 4.14 (q, *J* = 7.2 Hz, 2H), 4.03 – 3.95 (m, 2H), 3.55 (dd, *J* = 8.8, 5.2 Hz, 2H), 2.82 – 2.64 (m, 7H), 1.41 (s, 9H), 1.25 (t, *J* = 7.2 Hz, 3H). **^{13}C NMR** (101 MHz, Chloroform-*d*) δ 172.2, 156.4, 79.5, 60.9, 54.9, 35.9, 29.3, 28.5, 28.3, 17.7, 14.3. **HRMS (ESI-TOF)** *m/z*: Calcd $C_{14}H_{26}NO_4Se$ for $[M+H]^+$: 352.1022; found 352.1023.

ethyl 3-((2-(1H-indol-3-yl)ethyl)selanyl)propanoate (3q)

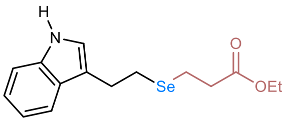

Prepared by general procedure; isolated as a yellow solid using petroleum/ethyl acetate (10:1) as eluent. (56.6 mg, 87%). **^{1}H NMR** (400 MHz, Chloroform-*d*) δ 8.07 (s, 1H), 7.62 (d, *J* = 8.0 Hz, 1H), 7.38 – 7.32 (m, 1H), 7.25 – 7.10 (m, 2H), 7.05 – 6.99 (m, 1H), 4.17 (q, *J* = 7.2 Hz, 2H), 3.20 – 3.12 (m, 2H), 3.01 – 2.91 (m, 2H), 2.86 – 2.78 (m, 2H), 2.77 – 2.68 (m, 2H), 1.28 (t, *J* = 7.2 Hz, 3H). **^{13}C NMR** (101 MHz, Chloroform-*d*) δ 172.5, 136.4, 127.1, 122.1, 121.7, 119.4, 118.7, 115.6, 111.3, 60.8, 35.9, 26.9, 24.8, 17.8, 14.3. **HRMS (ESI-TOF)** *m/z*: Calcd $C_{15}H_{20}NO_2Se$ for $[M+H]^+$: 326.0654; found 326.0652.

ethyl 3-((5-(benzo[d]oxazol-2-yl)pentyl)selanyl)propanoate(3r)

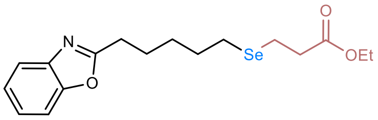

Prepared by general procedure; isolated as a white solid using petroleum/ethyl acetate (10:1) as eluent. (59.8 mg, 81%). **^{1}H NMR** (400 MHz, Chloroform-*d*) δ 7.70 – 7.62 (m, 1H), 7.54 – 7.43 (m, 1H), 7.35 – 7.27 (m, 2H), 4.14 (q, *J* = 7.2 Hz, 2H), 2.93 (t, *J* = 7.6 Hz, 2H), 2.82 – 2.73 (m, 2H), 2.73 – 2.64 (m, 2H), 2.60 (t, *J* = 7.4 Hz, 2H), 1.96 – 1.84 (m, 2H), 1.78 – 1.67 (m, 2H), 1.59 – 1.48 (m, 2H), 1.25 (t, *J* = 7.2 Hz, 3H). **^{13}C NMR** (101 MHz, Chloroform-*d*) δ 172.4,

167.1, 150.9, 141.4, 124.6, 124.2, 119.6, 110.4, 60.8, 36.0, 30.2, 29.5, 28.6, 26.4, 24.0, 17.6, 14.3. **HRMS (ESI-TOF)** *m/z*: Calcd $C_{17}H_{24}NO_3Se$ for $[M+H]^+$: 370.0916; found 370.0914.

ethyl 3-((4-((2-oxo-1,2,3,4-tetrahydroquinolin-7-yl)oxy)butyl)selanyl)propanoate (3s)

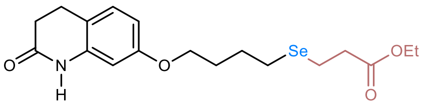

Prepared by general procedure; isolated as a white solid using petroleum/ethyl acetate (1:1) as eluent. (74.2 mg, 93%). **^{1}H NMR** (400 MHz, Chloroform-*d*) δ 8.04 (s, 1H), 7.04 (d, *J* = 8.4 Hz, 1H), 6.51 (dd, *J* = 8.4, 2.4 Hz, 1H), 6.33 (d, *J* = 2.4 Hz, 1H), 4.16 (q, *J* = 7.0 Hz, 2H), 4.01 – 3.88 (m, 2H), 2.93 – 2.84 (m, 2H), 2.84 – 2.74 (m, 2H), 2.76 – 2.67 (m, 2H), 2.69 – 2.55 (m, 4H), 1.93 – 1.78 (m, 4H), 1.27 (t, *J* = 7.1 Hz, 3H). **^{13}C NMR** (101 MHz, Chloroform-*d*) δ 172.3, 158.5, 138.1, 128.5, 115.6, 108.6, 102.2, 67.3, 60.6, 35.7, 31.0, 29.2, 26.9, 24.5, 23.8, 17.5, 14.2. **HRMS (ESI-TOF)** *m/z*: Calcd for $C_{18}H_{26}NO_4Se$ $[M+H]^+$: 400.1022; found 400.1015.

ethyl 3-(benzylselanyl)propanoate (3t)

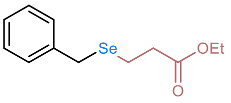

Prepared by general procedure; isolated as a colorless oil using petroleum/ethyl acetate (20:1) as eluent. (51.7 mg, 95%). **^{1}H NMR** (400 MHz, Chloroform-*d*) δ 7.29 (d, *J* = 4.4 Hz, 4H), 7.22 (h, *J* = 4.4 Hz, 1H), 4.14 (q, *J* = 7.0 Hz, 2H), 3.81 (s, 2H), 2.71 (td, *J* = 7.0, 1.6 Hz, 2H), 2.63 (td, *J* = 7.0, 1.6 Hz, 2H), 1.26 (t, *J* = 7.2 Hz, 3H).**^{13}C NMR** (101 MHz, Chloroform-*d*) δ 172.4, 139.2, 129.0, 128.7, 126.9, 60.8, 35.6, 27.5, 17.8, 14.3. **HRMS (ESI-TOF)** *m/z*: Calcd $C_{12}H_{17}O_2Se$ for $[M+H]^+$: 273.0388; found 273.0387.

ethyl 3-((4-fluorobenzyl)selanyl)propanoate(3u)

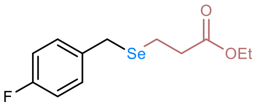

Prepared by general procedure; isolated as a colorless oil using petroleum/ethyl acetate (20:1) as eluent. (51.0 mg, 88%). **^{1}H NMR** (400 MHz, Chloroform-*d*) δ 7.34 – 7.17 (m, 2H), 7.05 – 6.87 (m, 2H), 4.14 (q, *J* = 7.2 Hz, 2H), 3.78 (s, 2H), 2.75 – 2.67 (m, 2H), 2.67 – 2.60 (m, 2H), 1.25 (t, *J* = 7.2 Hz, 3H). **^{13}C NMR** (101 MHz, Chloroform-*d*) δ 172.3, 161.8 (d, *J* = 245.4 Hz), 135.0 (d, *J* = 3.2 Hz), 130.5 (d, *J* = 8.2 Hz), 115.5 (d, *J* = 21.6 Hz), 60.8, 35.6, 26.6, 17.8, 14.3. **^{19}F NMR** (376 MHz, Chloroform-*d*) δ -115.78. **HRMS (ESI-TOF)** *m/z*: Calcd $C_{12}H_{16}FO_2Se$ for $[M+H]^+$: 291.0294; found 291.0296.

ethyl 3-((2-cyanobenzyl)selanyl)propanoate(3v)

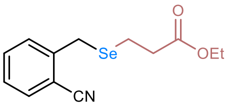

Prepared by general procedure; isolated as a yellow oil using petroleum/ethyl acetate (10:1) as eluent. (51.1 mg, 86%). **^{1}H NMR** (400 MHz, Chloroform-*d*) δ 7.63 – 7.59 (m, 1H), 7.54 – 7.49 (m, 1H), 7.45 – 7.41 (m, 1H), 7.34 – 7.28 (m, 1H), 4.14 (q, *J* = 7.2 Hz, 2H), 3.98 (s, 2H), 2.83 – 2.76 (m, 2H), 2.73 – 2.65 (m, 2H), 1.25 (t, *J* = 7.2 Hz, 3H). **^{13}C NMR** (101 MHz, Chloroform-*d*) δ 172.2, 143.8, 133.2, 133.0, 130.1, 127.4, 117.8, 112.2, 60.9, 35.6, 25.0, 18.5, 14.3. **HRMS (ESI-TOF)** *m/z*: Calcd $C_{13}H_{16}NO_2Se$ for $[M+H]^+$: 298.0341; found 298.0341.

ethyl 3-((2-cyanobenzyl)selanyl)propanoate(3w)

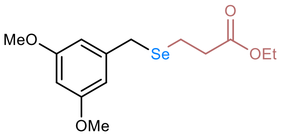

Prepared by general procedure; isolated as a white solid using petroleum/ethyl acetate (10:1) as eluent. (63.8 mg, 96%). **^{1}H NMR** (400 MHz, Chloroform-*d*) δ 6.45 (d, *J* = 2.4 Hz, 2H), 6.31 (t, *J* = 2.4 Hz, 1H), 4.14 (q, *J* = 7.1 Hz, 2H), 3.77 (s, 6H), 3.74 (s, 2H), 2.76 – 2.70 (m, 2H), 2.68 – 2.61 (m, 2H), 1.25 (t, *J* = 7.2 Hz, 3H). **^{13}C NMR** (101 MHz, Chloroform-*d*) δ 172.4, 160.9, 141.4, 106.9, 99.1, 60.7, 55.4, 35.6, 27.7, 17.9, 14.3. **HRMS (ESI-TOF)** *m/z*: Calcd $C_{14}H_{21}O_4Se$ for $[M+H]^+$: 333.0600; found 333.0596.

ethyl 3-((4-phenylbutan-2-yl)selanyl)propanoate(3x)

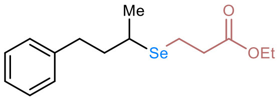

Prepared by general procedure; isolated as a colorless oil using petroleum/ethyl acetate (20:1) as eluent. (42.0 mg, 67%). **^{1}H NMR** (400 MHz, Chloroform-*d*) δ 7.25 – 7.17 (m, 2H), 7.15 – 7.08 (m, 3H), 4.08 (q, *J* = 7.2 Hz, 2H), 2.90 (h, *J* = 7.0 Hz, 1H), 2.77 – 2.57 (m, 6H), 1.94 – 1.73 (m, 2H), 1.40 (d, *J* = 7.0 Hz, 3H), 1.19 (t, *J* = 7.2 Hz, 3H). **^{13}C NMR** (101 MHz, Chloroform-*d*) δ 172.5, 141.8, 128.5, 126.0, 60.8, 39.7, 36.1, 34.8, 34.1, 22.7, 16.4, 14.4. **HRMS (ESI-TOF)** *m/z*: Calcd $C_{15}H_{23}O_2Se$ for $[M+H]^+$: 315.0858; found 315.0859.

ethyl 3-((1-phenylethyl)selanyl)propanoate(3y)

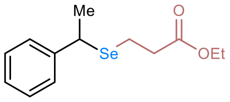

Prepared by general procedure; isolated as a colorless oil using petroleum/ethyl acetate (20:1) as eluent. (41.7 mg, 73%). **^{1}H NMR** (400 MHz, Chloroform-*d*) δ 7.39 – 7.27 (m, 4H), 7.25 – 7.17 (m, 1H), 4.22 (q, *J* = 7.2 Hz, 1H), 4.12 (q, *J* = 7.2 Hz, 2H), 2.70 – 2.57 (m, 2H), 2.59 – 2.48 (m, 2H), 1.72 (d, *J* = 7.2 Hz, 3H), 1.24 (t, *J* = 7.2 Hz, 3H). **^{13}C NMR** (101 MHz, Chloroform-*d*) δ 172.5, 144.3, 128.6, 127.4, 127.1, 60.7, 37.6, 35.5, 22.6, 18.2, 14.3. **HRMS (ESI-TOF)** *m/z*: Calcd for $C_{13}H_{19}O_2Se$ $[M+H]^+$: 287.0545; found 287.0543.

ethyl 3-(cyclopentylselanyl)propanoate(3z)

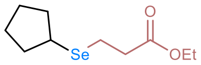

Prepared by general procedure; isolated as a colorless oil using petroleum/ethyl acetate (20:1) as eluent. (29.5 mg, 59%). **^{1}H NMR** (400 MHz, Chloroform-*d*) δ 4.15 (q, *J* = 7.2 Hz, 2H), 3.32 – 3.21 (m, 1H), 2.83 – 2.75 (m, 2H), 2.75 – 2.65 (m, 2H), 2.11 – 1.97 (m, 2H), 1.80 – 1.67 (m, 2H), 1.67 – 1.45 (m, 4H), 1.26 (t, *J* = 7.2 Hz, 3H). **^{13}C NMR** (101 MHz, Chloroform-*d*) δ 172.6, 60.8, 37.9, 36.1, 34.6, 25.0, 17.6, 14.4. **HRMS (ESI-TOF)** *m/z*: Calcd $C_{10}H_{19}O_2Se$ for $[M+H]^+$: 251.0545; found 251.0544.

***tert*-butyl 2-((3-ethoxy-3-oxopropyl)selanyl)-2-methylpropanoate (3aa)**

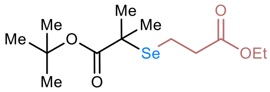

Prepared by general procedure; isolated as a colorless oil using petroleum/ethyl acetate (10:1) as eluent. (48.0 mg, 74%). **^{1}H NMR** (400 MHz, Chloroform-*d*) δ 4.13 (q, *J* = 7.2 Hz, 2H), 2.94 (t, *J* = 7.4 Hz, 2H), 2.70 (t, *J* = 7.4 Hz, 2H), 1.55 (s, 6H), 1.45 (s, 9H), 1.24 (t, *J* = 7.2 Hz, 3H). **^{13}C NMR** (101 MHz, Chloroform-*d*) δ 174.1, 172.3, 80.9, 60.8, 42.1, 35.5, 27.9, 26.2, 18.0, 14.3. **HRMS (ESI-TOF)** *m/z*: Calcd for $C_{13}H_{25}O_4Se$ $[M+H]^+$: 325.0913; found 325.0910.

ethyl 1-((3-ethoxy-3-oxopropyl)selanyl)cyclobutane-1-carboxylate (3ab)

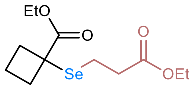

Prepared by general procedure; isolated as a colorless oil using petroleum/ethyl acetate (10:1) as eluent. (40.0 mg, 65%). **^{1}H NMR** (400 MHz, Chloroform-*d*) δ 4.25 – 4.08 (m, 4H), 2.87 (t, *J* = 7.4 Hz, 2H), 2.76 – 2.61 (m, 4H), 2.33 – 2.11 (m, 3H), 1.91 – 1.77 (m, 1H), 1.32 – 1.20 (m, 6H). **^{13}C NMR** (101 MHz, Chloroform-*d*) δ 175.5, 172.3, 61.1, 60.8, 43.5, 35.3, 32.7, 18.9, 16.4, 14.3, 14.2. **HRMS (ESI-TOF)** *m/z*: Calcd for $C_{12}H_{21}O_4Se$ $[M+H]^+$: 309.0600; found 309.0601.

ethyl 3-((naphthalen-2-ylmethyl)selanyl)propanoate (3ac)

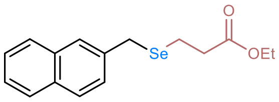

Prepared by general procedure; isolated as a white solid using petroleum/ethyl acetate (15:1) as eluent. (58.6 mg, 91%). **^{1}H NMR** (400 MHz, Chloroform-*d*) δ 7.84 – 7.75 (m, 3H), 7.69 (s, 1H), 7.53 – 7.41 (m, 3H), 4.14 (q, *J* = 7.2 Hz, 2H), 3.97 (s, 2H), 2.78 – 2.53 (m, 4H), 1.25 (t, *J* = 7.2 Hz, 3H). **^{13}C NMR** (101 MHz, Chloroform-*d*) δ 172.2, 136.3, 133.2, 132.3, 128.4, 127.5, 127.6, 127.2, 126.9, 126.1, 125.7, 60.6,

35.4, 27.6, 17.5, 14.1. **HRMS (ESI-TOF)** *m/z*: Calcd for C16H19O2Se $[M+H]^+$: 323.0545; found 323.0545.

ethyl 3-((2-methylallyl)selanyl)propanoate (3ad)

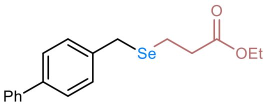

Prepared by general procedure; isolated as a white solid using petroleum/ethyl acetate (10:1) as eluent. (64.0 mg, 92%). **^{1}H NMR** (400 MHz, Chloroform-*d*) δ 7.62 – 7.56 (m, 2H), 7.56 – 7.50 (m, 2H), 7.48 – 7.41 (m, 2H), 7.40 – 7.31 (m, 3H), 4.16 (q, *J* = 7.2 Hz, 2H), 3.87 (s, 2H), 2.82 – 2.73 (m, 2H), 2.72 – 2.62 (m, 2H), 1.27 (t, *J* = 7.2 Hz, 3H). **^{13}C NMR** (101 MHz, Chloroform-*d*) δ 172.4, 140.8, 139.8, 138.3, 129.4, 128.9, 127.4, 127.3, 127.1, 60.8, 35.6, 27.1, 17.8, 14.3. **HRMS (ESI-TOF)** *m/z*: Calcd for $[M+H]^+$ $C_{18}H_{21}O_2Se$: 349.0701; found 349.0700.

ethyl 3-(([1,1'-biphenyl]-4-ylmethyl)selanyl)propanoate (3ae)

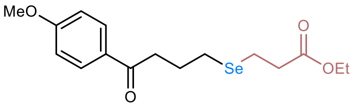

Prepared by general procedure; isolated as a colorless oil using petroleum/ethyl acetate (10:1) as eluent. (38.7 mg, 54%). **^{1}H NMR** (400 MHz, Chloroform-*d*) δ 7.95 (d, *J* = 8.8 Hz, 2H), 6.93 (d, *J* = 8.8 Hz, 2H), 4.15 (q, *J* = 7.2 Hz, 2H), 3.87 (s, 3H), 3.06 (t, *J* = 7.2 Hz, 2H), 2.89 – 2.74 (m, 2H), 2.75 – 2.59 (m, 4H), 2.09 (p, *J* = 7.2 Hz, 2H), 1.26 (t, *J* = 7.2 Hz, 3H). **^{13}C NMR** (101 MHz, Chloroform-*d*) δ 198.2, 172.4, 163.6, 130.4, 130.1, 113.8, 60.8, 55.6, 37.9, 35.8, 24.8, 23.9, 17.6, 14.3. **HRMS (ESI-TOF)** *m/z*: Calcd for $[M+H]^+$ $C_{16}H_{23}O_4Se$: 359.0756; found 359.0751.

ethyl 3-(((trimethylsilyl)methyl)selanyl)propanoate (3af)

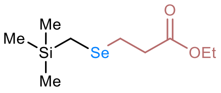

Prepared by general procedure; isolated as a colorless oil using petroleum/ethyl acetate (20:1) as eluent. (29.5 mg, 55%). **^{1}H NMR** (400 MHz, Chloroform-*d*) δ 4.15 (q, *J* = 7.2 Hz, 2H), 2.89 – 2.57 (m, 4H), 1.75 (s, 2H), 1.26 (t, *J* = 7.2 Hz, 3H), 0.08 (s, 9H). **^{13}C NMR** (101 MHz, Chloroform-*d*) δ 172.6, 60.8, 35.5, 20.4, 14.3, 9.1, -1.2. **HRMS (ESI-TOF)** *m/z*: Calcd for $C_9H_{21}O_2SeSi$ $[M+H]^+$: 269.0471; found 269.0471.

ethyl 3-((3-(4-(methylsulfonyl)phenoxy)propyl)selanyl)propanoate (3ag)

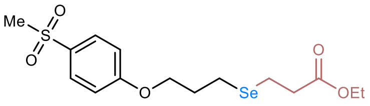

Prepared by general procedure; isolated as a white solid using petroleum/ethyl acetate (5:1) as eluent. (75.7 mg, 96%). **^{1}H NMR** (400 MHz, Chloroform-*d*) δ 7.85 (d, *J* = 8.8 Hz, 2H), 7.01 (d, *J* = 8.8 Hz, 2H), 4.19 – 4.09 (m, 4H), 3.02 (s, 3H), 2.83 – 2.64 (m, 6H), 2.17 (p, *J* = 6.8 Hz, 2H), 1.26 (t, *J* = 7.0 Hz, 3H). **^{13}C NMR** (101 MHz, Chloroform-*d*) δ 172.3, 163.0, 132.3, 129.7, 115.0, 67.5, 60.8, 45.0, 35.7, 29.7, 20.3, 17.9, 14.3. **HRMS (ESI-TOF)** *m/z*: Calcd for $C_{15}H_{23}O_5SSe$ $[M+H]^+$: 395.0426; found 395.0418.

ethyl 3-((2-((tert-butyldimethylsilyl)oxy)ethyl)selanyl)propanoate (3ah)

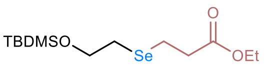

Prepared by general procedure; isolated as a colorless oil using petroleum/ethyl acetate (15:1) as eluent. (55.1 mg, 81%). **^{1}H NMR** (400 MHz, Chloroform-*d*) δ 4.15 (q, *J* = 7.0 Hz, 2H), 3.83 (t, *J* = 7.0 Hz, 2H), 2.88 – 2.77 (m, 2H), 2.77 – 2.59 (m, 4H), 1.26 (t, *J* = 7.0 Hz, 3H), 0.89 (s, 9H), 0.06 (s, 6H). **^{13}C NMR** (101 MHz, Chloroform-*d*) δ 172.4, 64.0, 60.8, 36.0, 26.6, 26.0, 18.5, 18.1, 14.3, -5.1. **HRMS (ESI-TOF)** *m/z*: Calcd for $C_{13}H_{29}O_3SeSi$ $[M+H]^+$: 341.1046; found 341.1051.

ethyl 3-((3-((6-bromonaphthalen-2-yl)oxy)propyl)selanyl)propanoate (3ai)

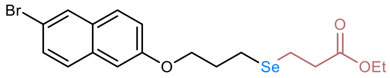

Prepared by general procedure; isolated as a white solid using petroleum/ethyl acetate (15:1) as eluent. (86.1 mg, 97%). **^{1}H NMR** (400 MHz, Chloroform-*d*) δ 7.91 (d, *J* = 2.0 Hz, 1H), 7.64 (d, *J* = 9.0 Hz, 1H), 7.59 (d, *J* = 8.8 Hz, 1H), 7.49 (dd, *J* = 8.8, 2.0 Hz, 1H), 7.15 (dd, *J* = 9.0, 2.4 Hz, 1H), 7.10 (d, *J* = 2.4 Hz, 1H), 4.27 – 4.09 (m, 4H), 2.93 – 2.77 (m, 4H), 2.76 – 2.68 (m, 2H), 2.21 (p, *J* = 6.6 Hz, 2H), 1.25 (t, *J* = 7.0 Hz, 3H). **^{13}C NMR** (101 MHz, Chloroform-*d*) δ 172.4, 157.1, 133.1, 130.1, 129.7, 129.7, 128.6, 128.5, 120.0, 117.1, 106.6, 67.0, 60.8, 35.8, 30.0, 20.6, 17.8, 14.3. **HRMS (ESI-TOF)** *m/z*: Calcd for $C_{18}H_{22}BrO_3Se$ $[M+H]^+$: 444.9912; found 444.9916.

***tert*-butyl 4-((3-ethoxy-3-oxopropyl)selanyl)piperidine-1-carboxylate (3aj)**

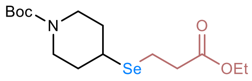

Prepared by general procedure; isolated as a white solid using petroleum/ethyl acetate (10:1) as eluent. (48.2 mg, 66%). **^{1}H NMR** (400 MHz, Chloroform-*d*) δ 4.14 (q, *J* = 7.2 Hz, 2H), 4.02 – 3.73 (m, 2H), 3.09 – 2.98 (m, 1H), 2.99 – 2.86 (m, 2H), 2.85 – 2.76 (m, 2H), 2.74 – 2.65 (m, 2H), 2.01 – 1.90 (m, 2H), 1.67 – 1.54 (m, 2H), 1.44 (s, 9H), 1.26 (t, *J* = 7.2 Hz, 3H). **^{13}C NMR** (101 MHz, Chloroform-*d*) δ 172.4, 154.8, 79.7, 60.8, 43.7, 36.1, 36.0, 33.4, 28.5, 16.4, 14.3. **HRMS (ESI-TOF)** *m/z*: Calcd for $C_{15}H_{28}NO_4Se$ $[M+H]^+$: 366.1178; found 366.1176.

ethyl 3-((4-phenylbutyl)selanyl)propanoate(3ak)

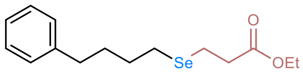

Prepared by general procedure; isolated as a colorless oil using petroleum/ethyl acetate (20:1) as eluent. (36.4 mg, 58%). **^{1}H NMR** (400 MHz, Chloroform-*d*) δ 7.26 – 7.17 (m, 2H), 7.17 – 7.03 (m, 3H), 4.08 (q, *J* = 7.2 Hz, 2H), 2.78 – 2.34 (m, 8H), 1.74 – 1.57 (m, *J* = 2.3 Hz, 4H), 1.19 (t, *J* = 7.2 Hz, 3H). **^{13}C NMR** (101 MHz, Chloroform-*d*) δ 172.5, 142.3, 128.5, 128.4, 125.9, 60.8, 35.9, 35.5, 31.7, 30.1, 24.2, 17.6, 14.3. **HRMS (ESI-TOF)** *m/z*: Calcd for $C_{15}H_{23}O_2Se$ $[M+H]^+$: 315.0858; found 315.0857.

ethyl 3-((2-(thiophen-2-yl)ethyl)selanyl)propanoate(3al)

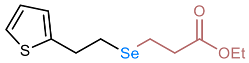

Prepared by general procedure; isolated as a colorless oil using petroleum/ethyl acetate (20:1) as eluent. (27.4 mg, 47%). **^{1}H NMR** (400 MHz, Chloroform-*d*) δ 7.17 (dd, *J* = 5.2, 1.2 Hz, 1H), 6.95 (dd, *J* = 5.2, 3.4 Hz, 1H), 6.90 – 6.83 (m, 1H), 4.18 (q, *J* = 7.2 Hz, 2H), 3.22 (t, *J* = 7.6 Hz, 2H), 2.90 (t, *J* = 7.6 Hz, 2H), 2.86 – 2.78 (m, 2H), 2.77 – 2.68 (m, 2H), 1.29 (t, *J* = 7.2 Hz, 3H). **^{13}C NMR** (101 MHz, Chloroform-*d*) δ 172.4, 143.8, 127.0, 124.9, 123.7, 60.8, 35.9, 31.4, 25.5, 17.9, 14.3. **HRMS (ESI-TOF)** *m/z*: Calcd for $C_{11}H_{17}O_2SSe$ $[M+H]^+$: 293.0109; found 293.0104.

ethyl 3-(methylselanyl)propanoate(3am)

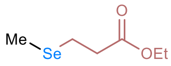

Prepared by general procedure; isolated as a colorless oil using petroleum/ethyl acetate (20:1) as eluent. (16.1 mg, 41%). **^{1}H NMR** (400 MHz, Chloroform-*d*) δ 4.15 (q, *J* = 7.2 Hz, 2H), 2.83 – 2.64 (m, 4H), 2.02 (s, 3H), 1.27 (t, *J* = 7.2 Hz, 3H). **^{13}C NMR** (101 MHz, Chloroform-*d*) δ 172.5, 60.8, 35.5, 19.1, 14.3, 4.4. **HRMS (GCQ-TOF)** *m/z*: Calcd for $C_6H_{12}O_2Se$ $[M]^+$:195.9997; found 196.0000.

ethyl 3-((2-methoxyethyl)selanyl)propanoate(3an)

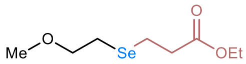

Prepared by general procedure; isolated as a colorless oil using petroleum/ethyl acetate (15:1) as eluent. (36.0 mg, 75%). **^{1}H NMR** (400 MHz, Chloroform-*d*) δ 4.15 (q, *J* = 7.2 Hz, 2H), 3.62 (t, *J* = 7.0 Hz, 2H), 3.36 (s, 3H), 2.87 – 2.68 (m, 6H), 1.26 (t, *J* = 7.2 Hz, 3H). **^{13}C NMR** (101 MHz, Chloroform-*d*) δ 172.4, 72.9, 60.8, 58.7, 36.0, 23.3, 18.1, 14.3. **HRMS (ESI-**

TOF) *m/z*: Calcd for $C_8H_{17}O_3Se$ $[M+H]^+$: 241.0338; found 241.0345.

benzyl 3-(phenethylselanyl)propanoate(4a)

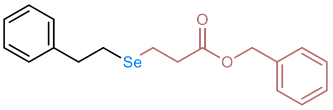

Prepared by general procedure; isolated as a white solid using petroleum/ethyl acetate (20:1) as eluent. (64.7 mg, 93%). **^{1}H NMR** (400 MHz, Chloroform-*d*) δ 7.39 – 7.33 (m, 5H), 7.33 – 7.27 (m, 2H), 7.25 – 7.18 (m, 3H), 5.15 (s, 2H), 3.01 – 2.94 (m, 2H), 2.90 – 2.82 (m, 2H), 2.82 – 2.70 (m, 4H). **^{13}C NMR** (101 MHz, Chloroform-*d*) δ 172.2, 141.2, 135.9, 128.7, 128.6, 128.5, 128.4, 126.5, 66.6, 37.2, 35.9, 25.4, 17.8. **HRMS (ESI-TOF)** *m/z*: Calcd $C_{18}H_{21}O_2Se$ for $[M+H]^+$:349.0702; found 349.0708.

naphthalen-2-ylmethyl 3-(phenethylselanyl)propanoate(4b)

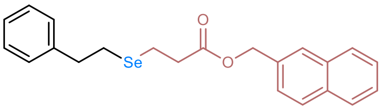

Prepared by general procedure; isolated as a white solid using petroleum/ethyl acetate (10:1) as eluent. (78.0 mg, 98%). **^{1}H NMR** (400 MHz, Chloroform-*d*) δ 7.89 – 7.83 (m, 4H), 7.54 – 7.46 (m, 3H), 7.33 – 7.27 (m, 2H), 7.25 – 7.18 (m, 3H), 5.32 (s, 2H), 3.04 – 2.93 (m, 2H), 2.89 – 2.83 (m, 2H), 2.83 – 2.76 (m, 4H). **^{13}C NMR** (101 MHz, Chloroform-*d*) δ 172.2, 141.1, 133.3,133.2, 128.6, 128.5, 127.5, 126.4,126.1, 126.0, 66.8, 37.2, 35.9, 25.4, 17.8. **HRMS (ESI-TOF)** *m/z*: Calcd $C_{22}H_{23}O_2Se$ for $[M+H]^+$:399.0858; found 399.0854.

isobutyl 3-(phenethylselanyl)propanoate(4c)

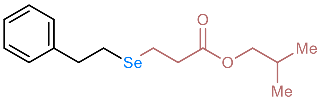

Prepared by general procedure; isolated as a colorless oil using petroleum/ethyl acetate (20:1) as eluent. (61.5 mg, 98%). **^{1}H NMR** (400 MHz, Chloroform-*d*) δ 7.33 – 7.27 (m, 2H), 7.25 – 7.18 (m, 3H), 3.89 (d, *J* = 6.6 Hz, 2H), 3.02 – 2.94 (m, 2H), 2.88 – 2.82 (m, 2H), 2.81 – 2.74 (m, 2H), 2.74 – 2.65 (m, 2H), 1.94 (dp, *J* = 13.4, 6.8 Hz, 1H), 0.94 (d, *J* = 6.8 Hz, 6H). **^{13}C NMR** (101 MHz, Chloroform-*d*) δ 172.4, 141.2, 128.6, 128.5, 126.5, 70.9, 37.2, 35.9, 27.8, 25.3, 19.2, 17.9. **HRMS (ESI-TOF)** *m/z*: Calcd $C_{15}H_{23}O_3Se$ for $[M+H]^+$:315.0858; found 315.0856.

***tert*-butyl 3-(phenethylselanyl)propanoate(4d)**

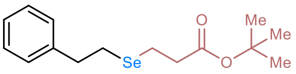

Prepared by general procedure; isolated as a colorless oil using petroleum/ethyl acetate (20:1) as eluent. (60.3 mg, 96%). **^{1}H NMR** (400 MHz, Chloroform-*d*) δ 7.33 – 7.27 (m, 2H), 7.24 – 7.18 (m, 3H), 3.06 – 2.93 (m, 2H), 2.89 – 2.78 (m, 2H), 2.74 (t, *J* = 7.6 Hz, 2H), 2.65 – 2.55 (m, 2H), 1.46 (s, 9H). **^{13}C NMR** (101 MHz, Chloroform-*d*) δ 171.7, 141.3, 128.6, 128.5, 126.5, 80.9, 37.3, 37.0, 28.2, 25.3, 18.2. **HRMS (ESI-TOF)** *m/z*: Calcd $C_{15}H_{23}O_2Se$ for $[M+H]^+$:315.0858; found 315.0861.

allyl 3-(phenethylselanyl)propanoate (4e)

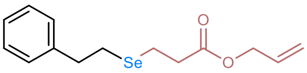

Prepared by general procedure; isolated as a colorless oil using petroleum/ethyl acetate (20:1) as eluent. (56.6 mg, 95%). **^{1}H NMR** (400 MHz, Chloroform-*d*) δ 7.30 (m, 2H), 7.22 (m, 3H), 5.98 - 5.86 (m, 1H), 5.33 (dq, *J* = 17.2, 1.6 Hz, 1H), 5.25 (dq, *J* = 10.4, 1.4 Hz, 1H), 4.60 (dt, *J* = 5.8, 1.4 Hz, 2H), 3.03 – 2.94 (m, 2H), 2.87 – 2.82 (m, 2H), 2.82 – 2.70 (m, 4H). **^{13}C NMR** (101 MHz, Chloroform-*d*) δ 172.0, 141.2, 132.2, 128.6, 128.5, 126.5, 118.6, 65.5, 37.2, 35.8, 25.4, 17.8. **HRMS (ESI-TOF)** *m/z*: Calcd $C_{14}H_{19}O_2Se$ for $[M+H]^+$:299.0545; found 299.0545.

2-phenoxyethyl 3-(phenethylselanyl)propanoate(4f)

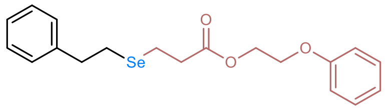

Prepared by general procedure; isolated as a white solid using petroleum/ethyl acetate (10:1) as eluent. (69.6 mg, 92%). **^{1}H NMR** (400 MHz, Chloroform-*d*) δ 7.34 – 7.27 (m, 4H), 7.25 – 7.17 (m, 3H), 6.98 (m, 1H), 6.95 – 6.89 (m, 2H), 4.49 – 4.44 (m, 2H), 4.21 – 4.16 (m, 2H), 3.02 – 2.93 (m, 2H), 2.89 – 2.81 (m, 2H), 2.82 – 2.70 (m, 4H). **^{13}C NMR** (101 MHz, Chloroform-*d*) δ 172.3, 158.6, 141.2, 129.7, 128.6, 128.5, 126.5, 121.3, 114.8, 65.9, 63.2, 37.2, 35.7, 25.4, 17.7. **HRMS (ESI-TOF)** *m/z*: Calcd $C_{19}H_{23}O_3Se$ for $[M+H]^+$:379.0807; found 379.0806.

phenethyl(2-(phenylsulfonyl)ethyl)selane(4g)

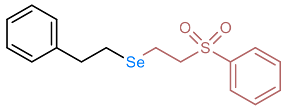

Prepared by general procedure; isolated as a white solid using petroleum/ethyl acetate (5:1) as eluent. (30.4 mg, 43%). **^{1}H NMR** (400 MHz, Chloroform-*d*) δ 7.91 – 7.87 (m, 2H), 7.71 – 7.66 (m, 1H), 7.62 – 7.56 (m, 2H), 7.31 – 7.26 (m, 2H), 7.23 – 7.18 (m, 1H), 7.16 – 7.12 (m, 2H), 3.32 – 3.26 (m, 2H), 2.95 – 2.88 (m, 2H), 2.84 – 2.79 (m, 2H), 2.75 – 2.67 (m, 2H). **^{13}C NMR** (101 MHz, Chloroform-*d*) δ 140.6, 138.7, 134.1, 129.6, 128.7, 128.5, 128.3, 126.7, 57.5, 36.9, 26.0, 14.0. **HRMS (ESI-TOF)** *m/z*: Calcd $C_{16}H_{19}O_2SSe$ for $[M+H]^+$: 355.0271; found 355.0266.

2-(2-(phenethylselanyl)ethyl)pyridine(4h)

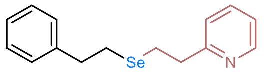

Prepared by general procedure; isolated as a colorless oil using petroleum/ethyl acetate (1:1) as eluent. (55.3 mg, 95%). **^{1}H NMR** (400 MHz, Chloroform-*d*) δ 8.54 (ddd, *J* = 5.0, 2.0, 1.0 Hz, 1H), 7.60 (td, *J* = 7.6, 2.0 Hz, 1H), 7.32 – 7.26 (m, 2H), 7.24 – 7.08 (m, 5H), 3.15 (dd, *J* = 8.2, 7.0 Hz, 2H), 2.96 (m, 4H), 2.85 – 2.72 (m, 2H). **^{13}C NMR** (101 MHz, Chloroform-*d*) δ 160.7, 149.5, 141.4, 136.5, 128.6, 128.5, 126.4, 123.2, 121.6, 39.4, 37.2, 25.2, 23.1. **HRMS (ESI-TOF)** *m/z*: Calcd $C_{15}H_{18}NSe$ for $[M+H]^+$:292.0599; found 292.0596.

4-(2-(phenethylselanyl)ethyl)pyridine(4i)

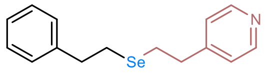

Prepared by general procedure; isolated as a colorless oil using petroleum/ethyl acetate (1:1) as eluent. (36.7 mg, 63%). **^{1}H NMR** (400 MHz, Chloroform-*d*) δ 8.55 – 8.48 (m, 2H), 7.33 – 7.28 (m, 2H), 7.25 – 7.22 (m, 1H), 7.22 – 7.17 (m, 2H), 7.13 – 7.09 (m, 2H), 3.00 – 2.90 (m, 4H), 2.84 – 2.73 (m, 4H). **^{13}C NMR** (101 MHz, Chloroform-*d*) δ 150.1, 149.9, 141.1, 128.7, 128.5, 126.6, 123.9, 37.2, 36.4, 25.5, 23.5. **HRMS (ESI-TOF)** *m/z*: Calcd $C_{15}H_{18}NSe$ for $[M+H]^+$:292.0599; found 292.0599.

5-(phenethylselanyl)hexan-3-one(4j)

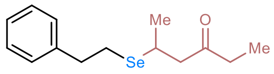

Prepared by general procedure; isolated as a colorless oil using petroleum/ethyl acetate (20:1) as eluent. (43.6 mg, 77%). **^{1}H NMR** (400 MHz, Chloroform-*d*) δ 7.31 – 7.25 (m, 2H), 7.22 – 7.15 (m, 3H), 3.45 – 3.33 (m, 1H), 3.00 – 2.90 (m, 2H), 2.87 – 2.81 (m, 2H), 2.77 (dd, *J* = 16.8, 6.4 Hz, 1H), 2.63 (dd, *J* = 16.8, 8.0 Hz, 1H), 2.39 (qd, *J* = 7.4, 2.6 Hz, 2H), 1.40 (d, *J* = 7.0 Hz, 3H), 1.04 (t, *J* = 7.4 Hz, 3H). **^{13}C NMR** (101 MHz, Chloroform-*d*) δ 209.6, 141.4, 128.6, 128.5, 126.5, 50.8, 37.3, 36.8, 28.8, 24.6, 22.9, 7.8. **HRMS (ESI-TOF)** *m/z*: Calcd $C_{14}H_{21}OSe$ for $[M+H]^+$:285.0752; found 285.0749.

1-(2-(phenethylselanyl)cyclohexyl)ethan-1-one(4k)

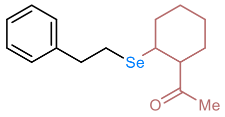

Prepared by general procedure; isolated as a colorless oil using petroleum/ethyl acetate (15:1) as eluent. (42.2 mg, 68%). **^{1}H NMR** (400 MHz, Chloroform-*d*) δ 7.33 – 7.25 (m, 2H), 7.24 – 7.14 (m, 3H), 3.49 – 3.40 (m, 1H), 2.99 – 2.90 (m, 2H), 2.87 – 2.73 (m, 2H), 2.61 (dt, *J* = 10.7, 3.7 Hz, 1H), 2.16 (s, 3H), 2.09 – 1.94 (m, 1H), 1.88 – 1.75

(m, 2H), 1.74 – 1.63 (m, 2H), 1.63 – 1.60 (m, 1H), 1.57 – 1.43 (m, 1H), 1.32 – 1.15 (m, 1H). **^{13}C NMR** (101 MHz, Chloroform-*d*) δ 209.7, 141.4, 128.6, 128.5, 126.4, 54.9, 41.8, 37.4, 33.5, 28.3, 26.1, 25.2, 24.6, 22.7. **HRMS (ESI-TOF)** *m/z*: Calcd $C_{16}H_{23}OSe$ for $[M+H]^+$:311.0909; found 311.0905.

5-(cyclopentylselanyl)hexan-3-one (4l)

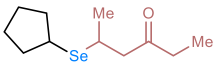

Prepared by general procedure; isolated as a colorless oil using petroleum/ethyl acetate (20:1) as eluent. (29.8 mg, 60%). **^{1}H NMR** (400 MHz, Chloroform-*d*) δ 3.43 (h, *J* = 7.0 Hz, 1H), 3.27 (p, *J* = 7.0 Hz, 1H), 2.83 (dd, *J* = 16.8, 6.0 Hz, 1H), 2.68 (dd, *J* = 16.8, 8.0 Hz, 1H), 2.53 – 2.33 (m, 2H), 2.15 – 1.99 (m, 2H), 1.80 – 1.68 (m, 2H), 1.63 – 1.55 (m, 4H), 1.45 (d, *J* = 7.0 Hz, 3H), 1.06 (t, *J* = 7.2 Hz, 3H). **^{13}C NMR** (101 MHz, Chloroform-*d*) δ 209.8, 51.1, 37.5, 36.9, 35.0, 34.9, 29.0, 25.1, 25.0, 23.4, 7.8. **HRMS (ESI-TOF)** *m/z*: Calcd $C_{11}H_{21}OSe$ for $[M+H]^+$: 249.0752; found 251.0544.

2-methyl-3-(phenethylselanyl)-N-phenylpropanamide (4m)

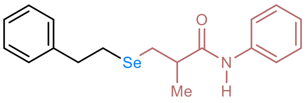

Prepared by general procedure; isolated as a white solid using petroleum/ethyl acetate (5:1) as eluent. (58.3 mg, 84%) **^{1}H NMR** (400 MHz, Chloroform-*d*) δ 7.55 (d, *J* = 8.0 Hz, 2H), 7.42 – 7.30 (m, 4H), 7.27 – 7.11 (m, 4H), 3.09 – 2.81 (m, 5H), 2.67 (dd, *J* = 12.4, 5.6 Hz, 1H), 2.53 (h, *J* = 6.8 Hz, 1H), 1.33 (d, *J* = 6.8 Hz, 3H). **^{13}C NMR** (101 MHz, Chloroform-*d*) δ 173.3, 141.1, 137.8, 129.1, 128.6, 126.5, 124.5, 120.0, 44.1, 37.1, 27.9, 26.5, 18.8. **HRMS (ESI-TOF)** *m/z*: Calcd for $C_{18}H_{22}NOSe$ $[M+H]^+$: 348.0861; found . 348.0861

***N*,*N*-dimethyl-3-(phenethylselanyl)propanamide (4n)**

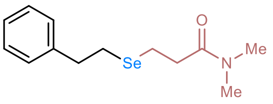

Prepared by general procedure; isolated as a colorless oil using petroleum/ethyl acetate (5:1) as eluent. (52.5 mg, 92%) **^{1}H NMR** (400 MHz, Chloroform-*d*) δ 7.35 – 7.27 (m, 2H), 7.27 – 7.17 (m, 3H), 3.08 – 2.94 (m, 8H), 2.92 – 2.80 (m, 4H), 2.69 (t, *J* = 7.6 Hz, 2H). **^{13}C NMR** (101 MHz, Chloroform-*d*) δ 171.7, 141.3, 128.6, 128.5, 126.4, 37.3, 37.2, 35.6, 34.8, 25.6, 18.7. **HRMS (ESI-TOF)** *m/z*: Calcd for $C_{13}H_{20}NOSe$ $[M+H]^+$: 286.0705; found 286.0697.

methyl 2-acetamido-3-(phenethylselanyl)propanoate (4o)

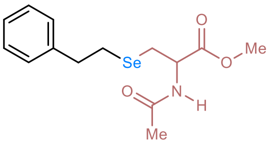

Prepared by general procedure; isolated as a white solid using petroleum/ethyl acetate (5:1) as eluent. (63.2 mg, 96%). **^{1}H NMR** (400 MHz, Chloroform-*d*) δ 7.34 – 7.25 (m, 2H), 7.25 – 7.14 (m, 3H), 6.36 (d, *J* = 7.6 Hz, 1H), 4.88 (dt, *J* = 7.5, 5.0 Hz, 1H), 3.74 (s, 3H), 3.08 – 2.90 (m, 4H), 2.86 – 2.75 (m, 2H), 2.02 (s, 3H). **^{13}C NMR** (101 MHz, Chloroform-*d*) δ 171.5, 169.9, 140.8, 128.7, 128.5, 126.6, 52.8, 52.2, 37.0, 26.0, 25.8, 23.3. **HRMS (ESI-TOF)** *m/z*: Calcd for $C_{14}H_{20}NO_3Se$ $[M+H]^+$: 330.0603; found 336.0600.

1-morpholino-3-(phenethylselanyl)propan-1-one (4p)

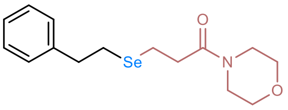

Prepared by general procedure; isolated as a white solid using petroleum/ethyl acetate (3:1) as eluent. (53.0 mg, 81%). **^{1}H NMR** (400 MHz, Chloroform-*d*) δ 7.33 – 7.23 (m, 2H), 7.25 – 7.14 (m, 3H), 3.70 – 3.56 (m, 6H), 3.47 – 3.34 (m, 2H), 3.03 – 2.91 (m, 2H), 2.82 (q, *J* = 8.0 Hz, 4H), 2.64 (t, *J* = 7.6 Hz, 2H). **^{13}C NMR** (101 MHz, Chloroform-*d*) δ 170.4, 141.2, 128.6, 128.5, 126.5, 67.0, 66.7, 45.9, 42.1, 37.2, 34.5, 25.8, 18.6. **HRMS (ESI-TOF)** *m/z*: Calcd for $C_{15}H_{22}NO_2Se$ $[M+H]^+$: 328.0811; found 328.0803.

ethyl 4-(phenethylselanyl)butanoate (4q)

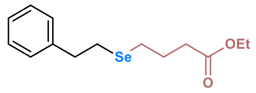

Prepared by general procedure; isolated as a colorless oil using petroleum/ethyl acetate (20:1) as eluent. (42.3 mg, 70%). **^{1}H NMR** (400 MHz, Chloroform-*d*) δ 9.07 7.34 – 7.27 (m, 2H), 7.25 – 7.18 (m, 3H), 4.16 (q, *J* = 6.9 Hz, 2H), 3.40 – 3.30 (m, 1H), 3.20 – 2.94 (m, 3H), 2.91 – 2.82 (m, 2H), 2.75 – 2.53 (m, 2H), 1.50 – 1.40 (m, 2H), 1.27 (t, *J* = 7.3 Hz, 3H). **^{13}C NMR** (101 MHz, Chloroform-*d*) δ 177.0, 141.4, 128.6, 128.5, 126.5, 60.7, 43.4, 37.2, 29.6, 24.3, 22.6, 14.4. **HRMS (ESI-TOF)** *m/z*: Calcd $C_{14}H_{21}O_2Se$ for $[M+H]^+$: 301.0701; found 301.0706.

4-(phenethylselanyl)butanenitrile (4r)

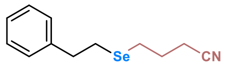

Prepared by general procedure; isolated as a yellow oil using petroleum/ethyl acetate (20:1) as eluent. (35.9 mg, 71%). **^{1}H NMR** (400 MHz, Chloroform-*d*) δ 7.28 – 7.20 (m, 2H), 7.19 – 7.10 (m, 3H), 3.05 – 2.96 (m, 1H), 2.95 – 2.89 (m, 2H), 2.88 – 2.80 (m, 2H), 2.63 (dd, *J* = 16.9, 5.6 Hz, 1H), 2.51 (dd, *J* = 16.9, 7.7 Hz, 1H), 1.48 – 1.39 (m, 3H). **^{13}C NMR** (101 MHz, Chloroform-*d*) δ 140.8, 128.7, 128.5, 126.7, 118.1, 37.0, 28.7, 27.2, 25.3, 21.9. **HRMS (ESI-TOF)** *m/z*: Calcd $C_{12}H_{19}N_2Se$ for $[M+NH_4]^+$: 271.0708; found 271.0712.

phenethyl(3-(phenylsulfonyl)propyl)selane (4s)

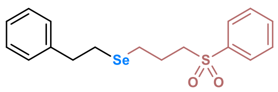

Prepared by general procedure; isolated as a yellow oil using petroleum/ethyl acetate (20:1) as eluent. (66.2 mg, 90%). **^{1}H NMR** (400 MHz, Chloroform-*d*) δ 8.04 – 7.94 (m, 2H), 7.77 – 7.71 (m, 1H), 7.69 – 7.61 (m, 2H), 7.40 – 7.32 (m, 2H), 7.32 – 7.25 (m, 1H), 7.24 – 7.19 (m, 2H), 3.55 – 3.31 (m, 3H), 3.02 – 2.93 (m, 2H), 2.91 – 2.81 (m, 2H), 1.67 (d, *J* = 6.5 Hz, 3H). **^{13}C NMR** (101 MHz, Chloroform-*d*) δ 140.7, 139.6, 134.0, 129.5, 128.6, 128.4, 128.0, 126.6, 63.9, 36.7, 26.0, 25.0, 21.8. **HRMS (ESI-TOF)** *m/z*: Calcd $C_{17}H_{24}NO_2SSe$ for $[M+NH_4]^+$: 386.0687; found 386.0688.

1,9-dioxa-5,13-diselenacyclohexadecane-2,10-dione (4t)

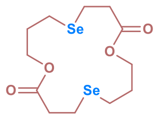

Prepared by general procedure; isolated as a yellow solid using ethyl acetate as eluent (52.4 mg, 68%). **^{1}H NMR** (400 MHz, Chloroform-*d*) δ 4.18 (t, *J* = 6.3 Hz, 4H), 2.83 – 2.68 (m, 8H), 2.68 – 2.57 (m, 4H), 2.06 – 1.94 (m, 4H). **^{13}C NMR** (101 MHz, Chloroform-*d*) δ 172.3, 64.2, 35.7, 29.6, 20.3, 17.8. **HRMS (ESI-TOF)** *m/z*: Calcd $C_{12}H_{21}O_4Se_2$ for $[M+H]^+$: 388.9765; found 388.9771.

3-((3-ethoxy-3-oxopropyl)selanyl)propyl 2-(3-cyano-4-isobutoxyphenyl)-4-methylthiazole -5-carboxylate (4u)

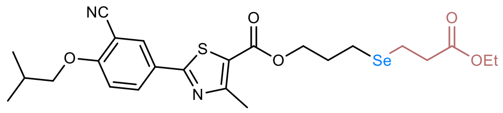

Prepared by general procedure; isolated as a white solid using petroleum/ethyl acetate (1:2) as eluent. (105.5 mg, 98%). **^{1}H NMR** (400 MHz, Chloroform-*d*) δ 8.16 (d, *J* = 2.2 Hz, 1H), 8.08 (dd, *J* = 8.8, 2.4 Hz, 1H), 7.00 (d, *J* = 9.0 Hz, 1H), 4.37 (t, *J* = 6.2 Hz, 2H), 4.14 (q, *J* = 7.2 Hz, 2H), 3.89 (d, *J* = 6.6 Hz, 2H), 2.86 – 2.77 (m, 2H), 2.74 (s, 3H), 2.74 – 2.68 (m, 4H), 2.23 – 2.14 (m, 1H), 2.14 – 2.06 (m, 2H), 1.25 (t, *J* = 7.2 Hz, 3H), 1.08 (d, *J* = 6.8 Hz, 6H). **^{13}C NMR** (101 MHz, Chloroform-*d*) δ 172.3, 167.4, 162.6, 162.0, 161.5, 132.7, 132.2, 126.1, 121.6, 115.5, 112.7, 103.1, 75.8, 64.7, 60.8, 35.8, 29.6, 28.2, 20.3, 19.1, 17.9, 17.6, 14.3. **HRMS (ESI-TOF)** *m/z*: Calcd $C_{24}H_{31}N_2O_5SSe$ for $[M+H]^+$: 539.1113; found 359.1103.

ethyl 3-((6-((3S,4R)-3-((benzo[d][1,3]dioxol-5-yloxy)methyl)-4-(4-fluorophenyl)piperidin-1-yl)-6-oxohexyl)selanyl)propanoate (4v)

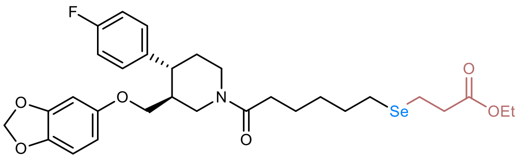

Prepared by general procedure; isolated as a white solid using ethyl acetate as eluent. (116.6 mg, 96%). **^{1}H NMR** (400 MHz, Chloroform-*d*) δ 7.12 (dd, *J* = 8.6, 5.6 Hz, 2H), 7.02 – 6.91 (m, 2H), 6.62 (t, *J* = 8.8 Hz, 1H), 6.34 (d, *J* = 2.4 Hz, 1H), 6.13 (dd, *J* = 8.6, 2.2 Hz, 1H), 5.87 (d, *J* = 7.0 Hz, 2H), 4.98 – 4.73 (m, 1H), 4.22 – 3.92 (m, 3H), 3.62 (dd, *J* = 9.6, 3.0 Hz, 1H), 3.46 (ddd, *J* = 10.2, 6.6, 4.4 Hz, 1H), 3.18 – 2.98 (m, 1H), 2.85 – 2.53 (m, 8H), 2.39 (td, *J* = 7.6, 4.4 Hz, 2H), 2.08 – 1.92 (m, 1H), 1.85 (dt, *J* = 20.2, 3.6 Hz, 1H), 1.76 – 1.59 (m, 5H), 1.56 – 1.36 (m, 2H), 1.25 (t, *J* = 7.2 Hz, 3H). **^{13}C NMR** (101 MHz, Chloroform-*d*) δ172.4, 171.4, 161.8 (d, *J* = 244.4 Hz), (154.4, 153.9), (148.3,148.2), (141.9, 141.8), 138.7, (128.9 (d, *J* = 9.0 Hz), 128.8 (d, *J* = 8.1 Hz)), (115.8 (d, *J* = 21.3 Hz), 115.7 (d, *J* = 21.3 Hz)), (108.0, 107.9), 105.6, (101.3, 101.2), (98.1 98.0,) (68.7,68.6), 60.8, (49.2, 46.2), (45.0, 44.5), (43.9, 42.9), (42.3, 41.9), 35.9, (34.7, 33.7), 33.3, 30.4, 29.8 ,25.0, 24.2,17.6,14.3. **^{19}F NMR** (376 MHz, Chloroform-*d*) δ -115.6, -115.8. **HRMS (ESI-TOF)** *m/z*: Calcd $C_{30}H_{39}FNO_6Se$ for $[M+H]^+$: 608.1921; found 608.1920.

((3aR,5R,5aS,8aS,8bR)-2,2,7,7-tetramethyltetrahydro-5H-bis([1,3]dioxolo)[4,5-b:4',5'-d]pyran-5-yl)methyl 6-((3-ethoxy-3-oxopropyl)selanyl)hexanoate(4w)

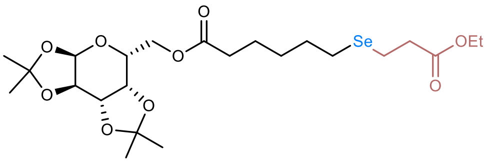

Prepared by general procedure; isolated as a colorless oil using petroleum/ethyl acetate (2:1) as eluent. (104.4 mg, 97%). **^{1}H NMR** (400 MHz, Chloroform-*d*) δ 5.51 (d, *J* = 5.0 Hz, 1H), 4.60 (dd, *J* = 8.0, 2.6 Hz, 1H), 4.32 – 4.25 (m, 2H), 4.21 (dd, *J* = 8.0, 2.0 Hz, 1H), 4.18 – 4.14 (m, 1H), 4.12 (dd, *J* = 8.0, 6.4 Hz, 2H), 3.99 (ddd, *J* = 8.0, 4.6, 2.0 Hz, 1H), 2.74 (td, *J* = 6.8, 1.8 Hz, 2H), 2.70 – 2.64 (m, 2H), 2.56 (t, *J* = 7.6 Hz, 2H), 2.33 (t, *J* = 7.6 Hz, 2H), 1.70 – 1.56 (m, 4H), 1.48 (s, 3H), 1.43 (s, 3H), 1.42 – 1.35 (m, 2H), 1.31 (d, *J* = 3.6 Hz, 6H), 1.24 (t, *J* = 7.2 Hz, 3H). **^{13}C NMR** (101 MHz, Chloroform-*d*) δ 173.6, 172.4, 109.7, 108.8, 96.4, 71.1, 70.8, 70.5, 66.1, 63.4, 60.7, 35.9, 34.1, 30.3, 29.4, 26.1, 26.0, 25.0, 24.6, 24.6, 24.0, 17.5, 14.3. **HRMS (ESI-TOF)** *m/z*: Calcd $C_{23}H_{39}O_9Se$ for $[M+NH_4]^+$: 556.2019; found 556.2011.

(3*S*,8*S*,9*S*,10*R*,13*R*,14*S*,17*R*)-17-((2*R*,5*S*,*E*)-5-ethyl-6-methylhept-3-en-2-yl)-10,13-dimethyl-2,3,4,7,8,9,10,11,12,13,14,15,16,17-tetradecahydro-1H-cyclopenta[a]phenanthren-3-yl 6-(2-(phenethylselanyl)ethyl)nicotinate (4x)

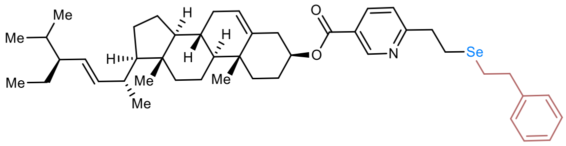

Prepared by general procedure; isolated as a yellow solid using petroleum/ethyl acetate (20:1) as eluent. (95.3 mg, 65%). **^{1}H NMR** (400 MHz, Chloroform-*d*) δ 9.07 (s, 1H), 8.16 – 8.10 (m, 1H), 7.24 – 7.17 (m, 2H), 7.17 – 7.08 (m, 4H), 5.38 – 5.33 (m, 1H), 5.13 – 5.04 (m, 1H), 5.01 – 4.90 (m, 1H), 4.85 – 4.74 (m, 1H), 3.17 – 3.09 (m, 2H), 2.93 – 2.84 (m, 4H), 2.76 – 2.68 (m, 2H), 2.42 – 2.36 (m, 2H), 2.04 – 1.82 (m, 6H), 1.71 – 1.59 (m, 3H), 1.42 – 1.29 (m, 2H), 1.26 – 1.05 (m, 7H), 1.03 – 0.90 (m, 10H), 0.82 – 0.70 (m, 10H), 0.64 (s, 3H). **^{13}C NMR** (101 MHz, Chloroform-*d*) δ 164.9, 164.8, 150.8, 141.2, 139.5, 138.9, 137.6, 129.3, 128.6, 128.0, 126.5, 125.5, 124.7, 123.1, 122.8, 75.1, 57.3, 56.0, 51.7, 50.1, 44.1, 41.1, 39.7, 39.3, 38.2, 37.6, 37.1, 36.7, 32.0, 31.9, 29.1, 27.9, 26.2, 25.3, 24.5, 22.7, 21.4, 21.3, 21.1, 19.5, 19.1, 12.4, 12.2. **HRMS (ESI-TOF)** *m/z*: Calcd $C_{45}H_{64}NO_2Se$ for $[M+H]^+$: 730.4097; found 730.4095.

(1R,2S,5R)-2-isopropyl-5-methylcyclohexyl 6-(2-(phenethylselanyl)ethyl)nicotinate (4y)

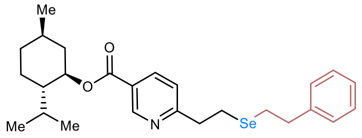

Prepared by general procedure; isolated as a yellow oil using petroleum/ethyl acetate (20:1) as eluent. (75.6 mg, 80%). **^{1}H NMR** (400 MHz, Chloroform-*d*) δ 9.07 (s, 1H), 8.16 – 8.09 (m, 1H), 7.26 – 7.18 (m, 2H), 7.17 – 7.07 (m, 4H), 4.87 (td, *J* = 10.8, 4.4 Hz, 1H), 3.13 (t, *J* = 7.5 Hz, 2H), 2.93 – 2.84 (m, 4H), 2.78 – 2.69 (m, 2H), 2.08 – 2.01 (m, 1H), 1.91 – 1.80 (m, 1H), 1.70 – 1.62 (m, 2H), 1.53 – 1.43 (m, 2H), 1.13 – 0.97 (m, 3H), 0.92 – 0.81 (m, 6H), 0.72 (d, *J* = 6.9 Hz, 3H). **^{13}C NMR** (101 MHz, Chloroform-*d*) δ 165.0, 164.9, 149.7, 141.2, 137.6, 128.6, 127.7, 126.5, 124.7, 122.4, 75.4, 47.2, 41.0, 39.3, 37.2, 34.3, 31.5, 26.6, 25.3, 23.6, 22.6, 22.2, 20.9, 16.6. **HRMS (ESI-TOF)** *m/z*: Calcd $C_{26}H_{36}NO_2Se$ for $[M+H]^+$: 474.1906; found 474.1910.

2-(1,3-dimethyl-2,6-dioxo-1,2,3,6-tetrahydro-7H-purin-7-yl)ethyl 6-(2-(phenethylselanyl)ethyl)nicotinate (4z)

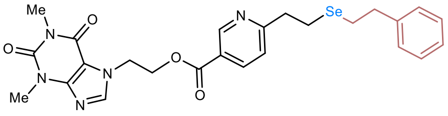

Prepared by general procedure; isolated as a yellow solid using ethyl acetate as eluent. (56.2 mg, 52%). **^{1}H NMR** (400 MHz, Chloroform-*d*) δ 9.00 (s, 1H), 8.06 (d, *J* = 8.1 Hz, 1H), 7.52 (s, 1H), 7.23 (d, *J* = 8.4 Hz, 2H), 7.19 – 7.11 (m, 4H), 4.71 – 4.62 (m, 4H), 3.54 (s, 3H), 3.36 (s, 3H), 3.15 (t, *J* = 7.5 Hz, 2H), 2.91 (t, *J* = 7.5 Hz, 4H), 2.80 – 2.72 (m, 2H). **^{13}C NMR** (101 MHz, Chloroform-*d*) δ 165.7, 164.8, 155.4, 151.7, 150.6, 149.2, 141.6, 141.2, 137.6, 128.6, 128.5, 126.5, 123.2, 123.1, 106.9, 63.5, 46.2, 39.3, 37.1, 30.0, 28.1, 25.4, 22.4, 1.1. **HRMS (ESI-TOF)** *m/z*: Calcd $C_{25}H_{28}N_5O4Se$ for $[M+H]^+$: 542.1301; found 542.1301.

6. Mechanistic experiments

6.1 Radical-trapping experiments

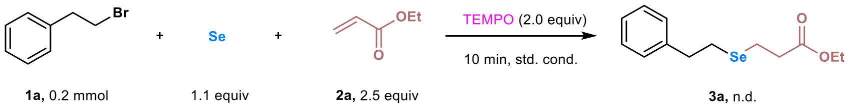

(2-bromoethyl)benzene **1a** (0.2 mmol, 1.0 equiv), 200 mesh Se powder (0.22 mmol, 1.1 equiv), K_2CO_3 (0.4 mmol, 2.0 equiv), MeCN (1.0 mmol, 5.0 equiv), ethyl acrylate **2a** (0.5 mmol, 2.5 equiv), and 2,2,6,6-tetramethylpiperidinyloxy (TEMPO, 0.4 mmol) were combined in a stainless-steel milling jar (10.0 mL) containing two stainless-steel balls (10 mm diameter) under an air atmosphere. $PhSiH_3$ (0.3 mmol, 1.5 equiv) was added all at once. After sealing the jar in air, it was subjected to grinding at 30 Hz for 10 min. The reaction was then quenched with ethyl acetate, after which the mixture was filtered. LC-MS analysis demonstrated the product **3a** was not found.

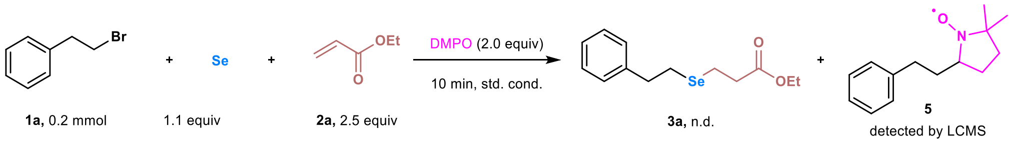

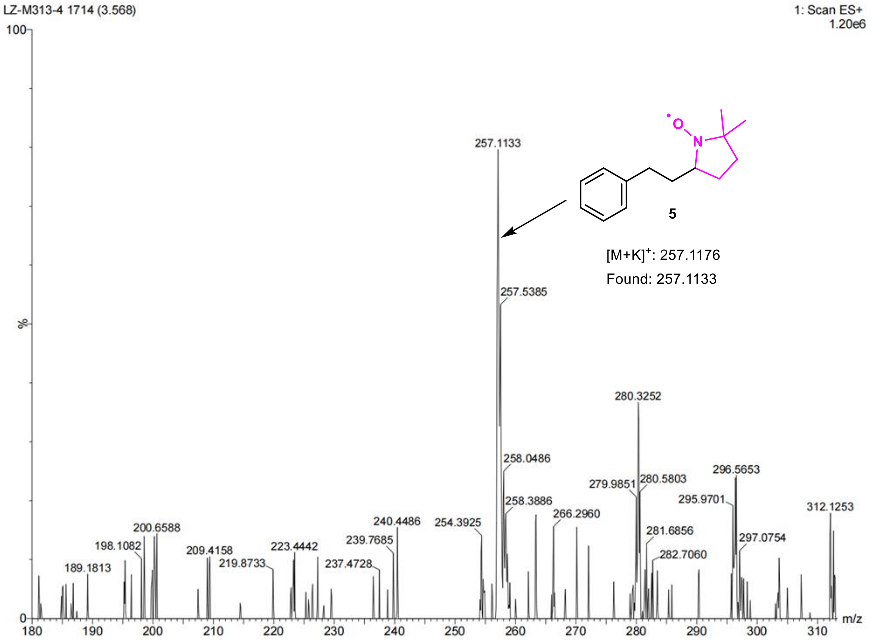

(2-bromoethyl)benzene **1a** (0.2 mmol, 1.0 equiv), 200 mesh Se powder (0.22 mmol, 1.1 equiv), K_2CO_3 (0.4 mmol, 2.0 equiv), MeCN (1.0 mmol, 5.0 equiv), ethyl acrylate **2a** (0.5 mmol, 2.5 equiv), and 5,5-dimethyl-1-pyrroline *N*-oxide (DMPO, 0.4 mmol) were combined in a stainless-steel milling jar (10.0 mL) containing two stainless-steel balls (10 mm diameter) under an air atmosphere. $PhSiH_3$ (0.3 mmol, 1.5 equiv) was added all at once. After sealing the jar in air, it was subjected to grinding at 30 Hz for 10 min. The reaction was then quenched with ethyl acetate, after which the mixture was filtered. LC-MS analysis demonstrated the product **3a** was not found. The benzyl radical combined with DMPO (**5**) was detected by LCMS.

6.2 Radical clock experiment

(2-bromoethyl)benzene **1a** (0.2 mmol, 1.0 equiv), 200 mesh Se powder (0.22 mmol, 1.1 equiv), K_2CO_3 (0.4 mmol, 2.0 equiv), MeCN (1.0 mmol, 5.0 equiv), vinylcyclopropane **2'** (0.5 mmol, 2.5 equiv) were combined in a stainless-steel milling jar (10.0 mL) containing two stainless-steel balls (10 mm diameter) under an air atmosphere. $PhSiH_3$ (0.3 mmol, 1.5 equiv) was added all at once. After sealing the jar in air, it was subjected to grinding at 30 Hz for 10 min. The reaction was then quenched with ethyl acetate, after which the mixture was filtered. Then the crude residue was purified by flash column chromatography on silica (pentane to pentane/EtOAc 20/1) to afford the product **6** (35.8 mg, 44%) as a colorless liquid.

^{1}H NMR (400 MHz, $CDCl_3$, **6**) δ 8.60 (d, J = 4.9 Hz, 1H), 7.69 – 7.61 (m, 1H), 7.52 – 7.45 (m, 1H), 7.37 – 7.22 (m, 9H), 7.19 – 7.13 (m, 2H), 6.35 (t, J = 7.3 Hz, 1H), 3.88 (s, 2H), 2.96 (t, J = 7.9 Hz, 2H), 2.89 – 2.79 (m, 4H), 2.72 – 2.61 (m, 2H). ^{13}C NMR (101 MHz, $CDCl_3$) 158.4, 148.9, 141.6, 141.5, 137.8, 136.6, 132.6, 128.6, 128.5, 126.4, 126.2, 122.0, 120.7, 37.4, 35.7, 31.2, 25.7, 20.5.

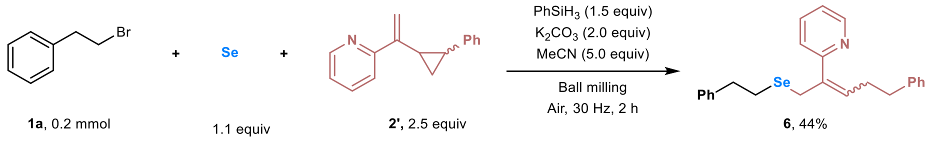

6.3 Intermediate experiments

(2-bromoethyl)benzene **1a** (0.2 mmol, 1.0 equiv), 200 mesh Se powder (0.22 mmol, 1.1 equiv), K_2CO_3 (0.4 mmol, 2.0 equiv), MeCN (1.0 mmol, 5.0 equiv), and ethyl acrylate **2a** (0.5 mmol, 2.5 equiv) were combined in a stainless-steel milling jar (10.0 mL) containing two stainless-steel balls (10 mm diameter) under an air atmosphere. $PhSiH_3$ (0.3 mmol, 1.5 equiv) was added all at once. After sealing the jar in air, it was subjected to grinding at 30 Hz for 10 min. The reaction was then quenched with ethyl acetate, after which the mixture was filtered. The resulting filtrate was concentrated, and the residue was purified by flash silica gel column chromatography, using a petroleum ether/ethyl acetate mixture as the eluent, to yield **3a**, along with the mixture of **7** and **8**.

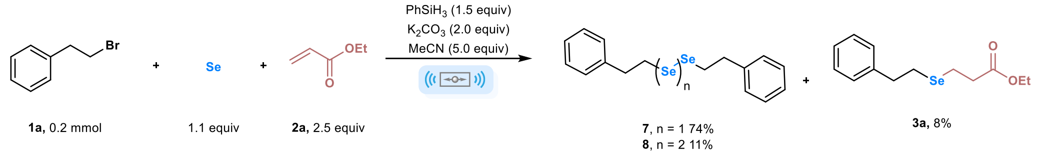

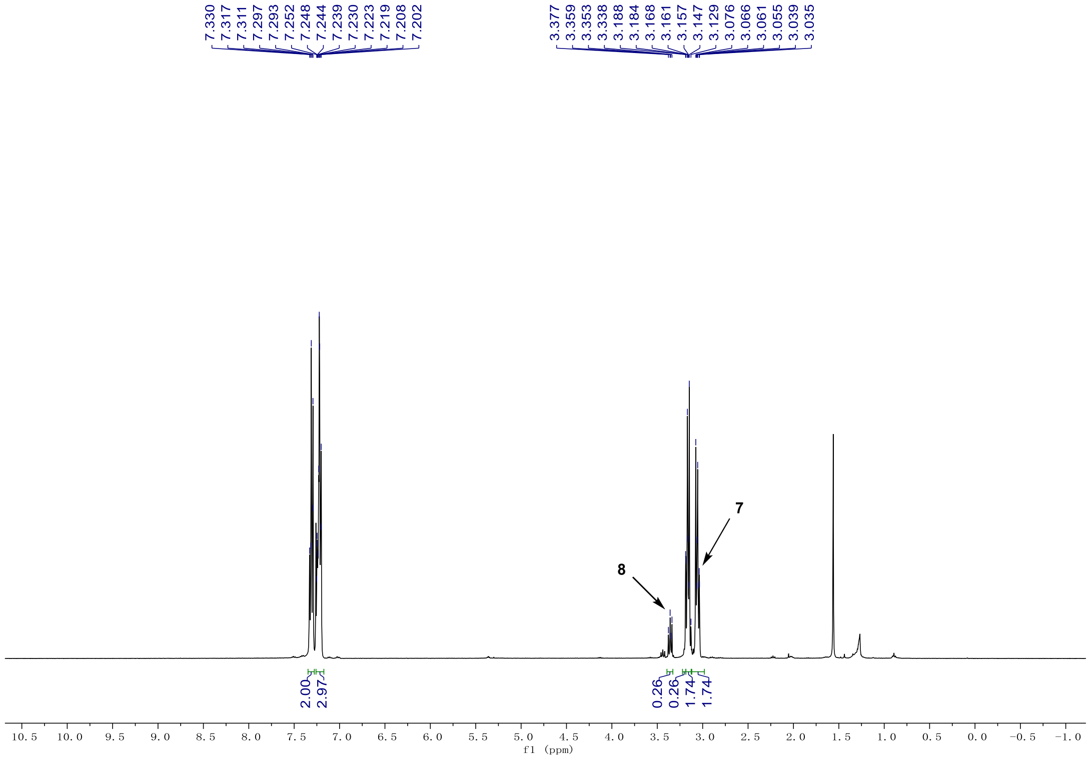

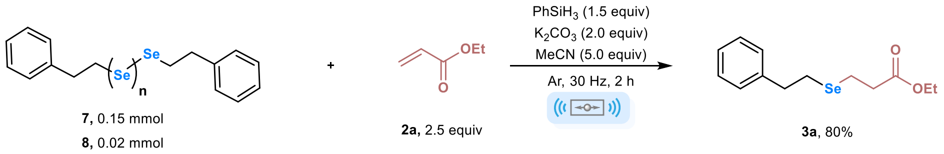

The above mixture of **7** (0.15 mmol) and **8** (0.02 mmol), K_2CO_3 (2.0 equiv), MeCN (5.0 equiv), and ethyl acrylate **2a** (2.5 equiv) were combined in a stainless-steel milling jar (10.0 mL) containing two stainless-steel balls (10 mm diameter) under an air atmosphere. $PhSiH_3$ (1.5 equiv) was added all at once. After sealing the jar in air, it was subjected to grinding at 30 Hz for 2 h. The reaction was then quenched with ethyl acetate, after which the mixture was filtered. The resulting filtrate was concentrated, and the residue was purified by flash silica gel column chromatography, using a petroleum ether/ethyl acetate mixture as the eluent, to yield the product **3a** (80% yield).

6.4 Deuterium labeling experiments

(2-bromoethyl)benzene **1a** (0.2 mmol, 1.0 equiv), 200 mesh Se powder (0.22 mmol, 1.1 equiv), K_2CO_3 (0.4 mmol, 2.0 equiv), MeCN (1.0 mmol, 5.0 equiv), and ethyl acrylate **2a** (0.5 mmol, 2.5 equiv) were combined in a stainless-steel milling jar (10.0 mL) containing two stainless-steel balls (10 mm diameter) under an air atmosphere. $PhSiD_3$ (0.3 mmol, 1.5 equiv) was added all at once. After sealing the jar in air, it was subjected to grinding at 30 Hz for 2 h. The reaction was then quenched with ethyl acetate, after which the mixture was filtered. The resulting filtrate was concentrated, and the residue was purified by flash silica gel column chromatography, using a petroleum ether/ethyl acetate mixture as the eluent, to yield the D-product **3a'** (52.8 mg, 92% yield, 54% D).

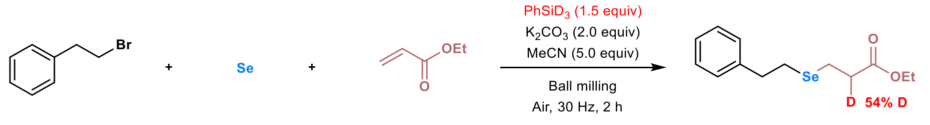

1a, 0.2 mmol   1.1 equiv   **2a,** 2.5 equiv   **3a'**, 92%

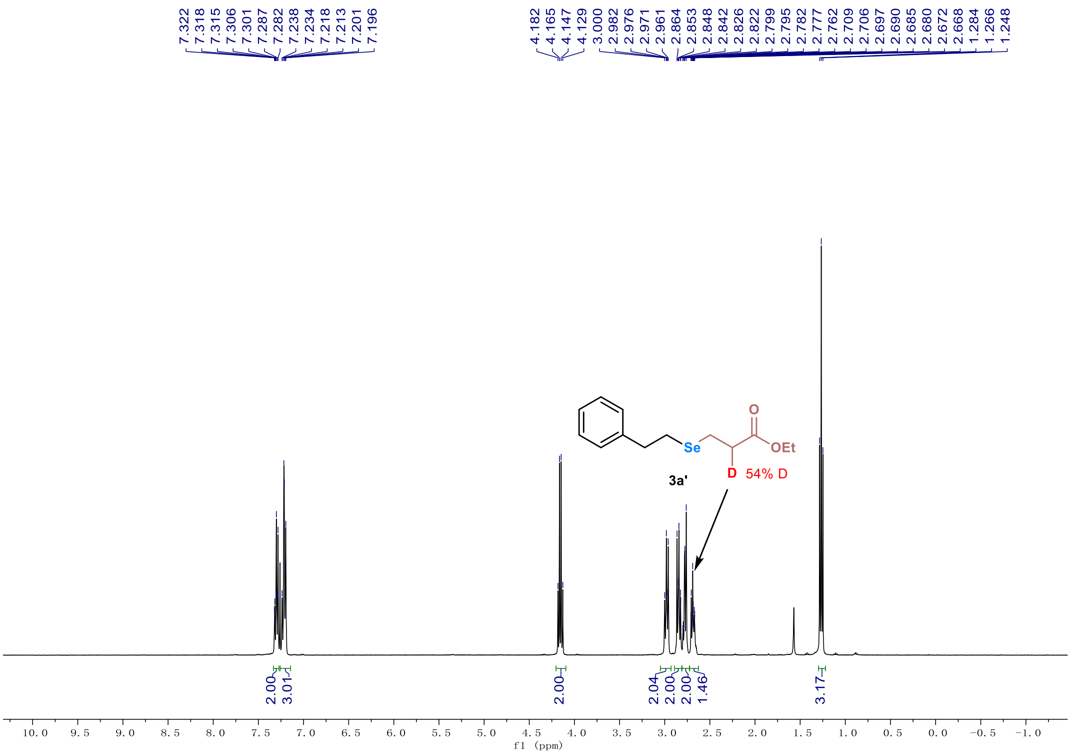

(2-bromoethyl)benzene **1a** (0.2 mmol, 1.0 equiv), 200 mesh Se powder (0.22 mmol, 1.1 equiv), K_2CO_3 (0.4 mmol, 2.0 equiv), MeCN (1.0 mmol, 5.0 equiv), and ethyl acrylate **2a** (0.5 mmol, 2.5 equiv) were combined in a stainless-steel milling jar (10.0 mL) containing two stainless-steel balls (10 mm diameter) under an Ar atmosphere. $PhSiD_3$ (0.3 mmol, 1.5 equiv) was added all at once. After sealing the jar in Ar, it was subjected to grinding at 30 Hz for 2 h. The reaction was then quenched with ethyl acetate, after which the mixture was filtered. The resulting filtrate was concentrated, and the residue was purified by flash silica gel column chromatography, using a petroleum ether/ethyl acetate mixture as the eluent, to yield the D-product **3a'** (51.6 mg, 90% yield, 71% D).

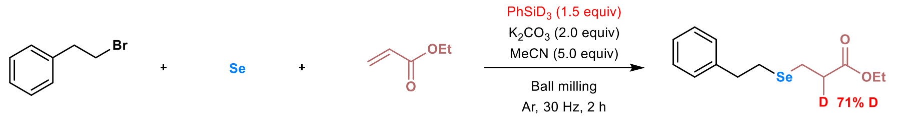

1a, 0.2 mmol   1.1 equiv   **2a,** 2.5 equiv   **3a'**, 90%

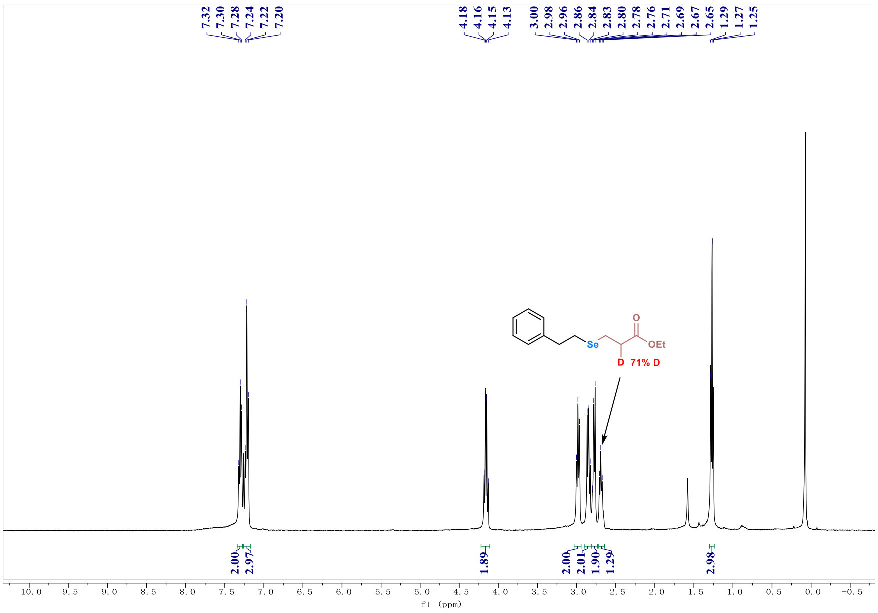

(2-bromoethyl)benzene **1a** (0.2 mmol, 1.0 equiv), 200 mesh Se powder (0.22 mmol, 1.1 equiv), K_2CO_3 (0.4 mmol, 2.0 equiv), CD_3CN (1.0 mmol, 5.0 equiv), and ethyl acrylate **2a** (0.5 mmol, 2.5 equiv) were combined in a stainless-steel milling jar (10.0 mL) containing two stainless-steel balls (10 mm diameter) under an air atmosphere. $PhSiH_3$ (0.3 mmol, 1.5 equiv) was added all at once. After sealing the jar in air, it was subjected to grinding at 30 Hz for 2 h. The reaction was then quenched with ethyl acetate, after which the mixture was filtered. The resulting filtrate was concentrated, and the residue was purified by flash silica gel column chromatography, using a petroleum ether/ethyl acetate mixture as the eluent, to yield the D-product **3a'** (51.0 mg, 89% yield).

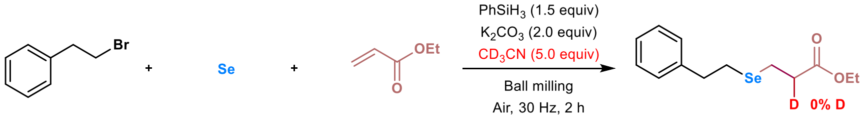

1a, 0.2 mmol 1.1 equiv **2a,** 2.5 equiv **3a'**, 89%

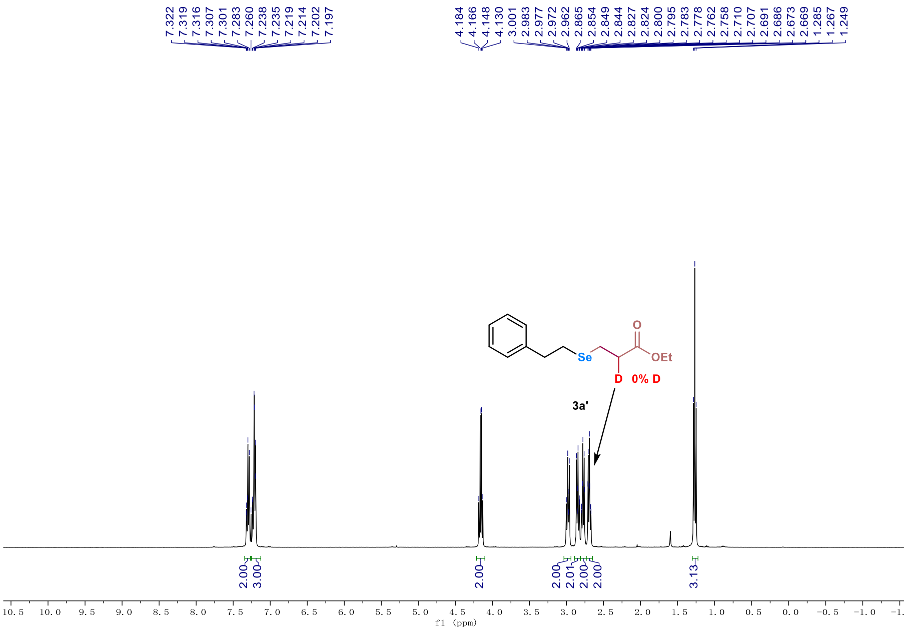

(2-bromoethyl)benzene **1a** (0.2 mmol, 1.0 equiv), 200 mesh Se powder (0.22 mmol, 1.1 equiv), K_2CO_3 (0.4 mmol, 2.0 equiv), CH_3CN (1.0 mmol, 5.0 equiv), ethyl acrylate **2a** (0.5 mmol, 2.5 equiv), and D_2O (0.4 mmol, 2.0 equiv) were combined in a stainless-steel milling jar (10.0 mL) containing two stainless-steel balls (10 mm diameter) under an air atmosphere. $PhSiH_3$ (0.3 mmol, 1.5 equiv) was added all at once. After sealing the jar in air, it was subjected to grinding at 30 Hz for 2 h. The reaction was then quenched with ethyl acetate, after which the mixture was filtered. The resulting filtrate was concentrated, and the residue was purified by flash silica gel column chromatography, using a petroleum ether/ethyl acetate mixture as the eluent, to yield the D-product **3a'** (28.7 mg, 50% yield, 53% D).

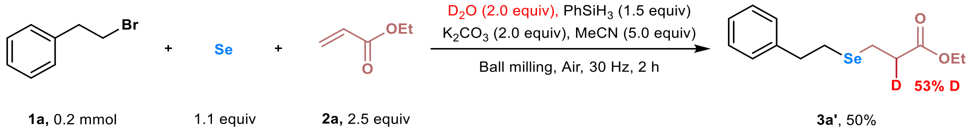

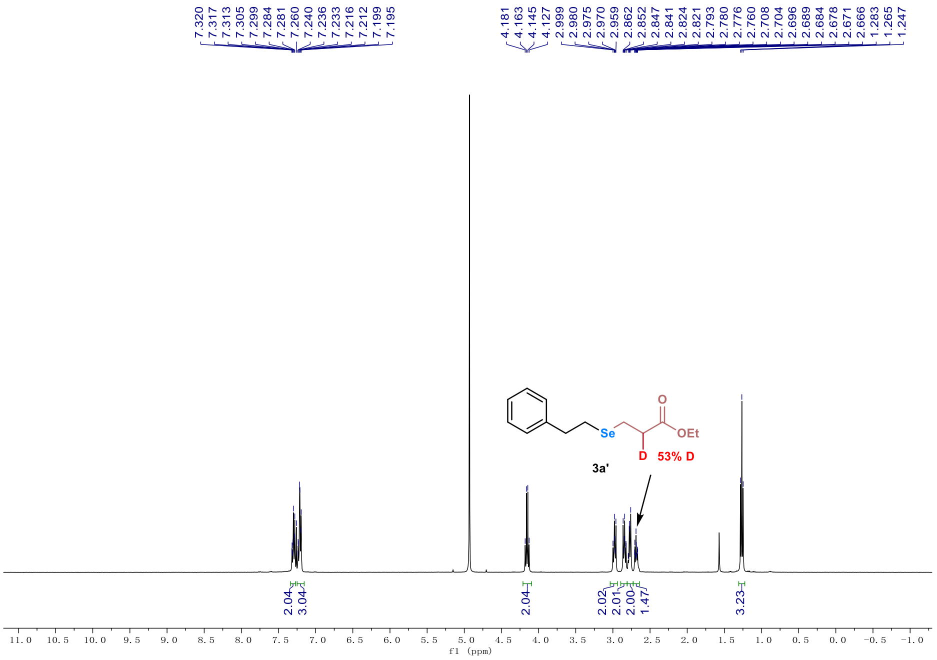

(2-bromoethyl)benzene **1a** (0.2 mmol, 1.0 equiv), 200 mesh Se powder (0.22 mmol, 1.1 equiv), K_2CO_3 (0.4 mmol, 2.0 equiv), CH_3CN (1.0 mmol, 5.0 equiv), ethyl acrylate **2a** (0.5 mmol, 2.5 equiv), and D_2O (0.4 mmol, 2.0 equiv) were combined in a stainless-steel milling jar (10.0 mL) containing two stainless-steel balls (10 mm diameter) under an air atmosphere. $PhSiD_3$ (0.3 mmol, 1.5 equiv) was added all at once. After sealing the jar in air, it was subjected to grinding at 30 Hz for 2 h. The reaction was then quenched with ethyl acetate, after which the mixture was filtered. The resulting filtrate was concentrated, and the residue was purified by flash silica gel column chromatography, using a petroleum ether/ethyl acetate mixture as the eluent, to yield the D-product **3a'** (49.0 mg, 85% yield, 74% D).

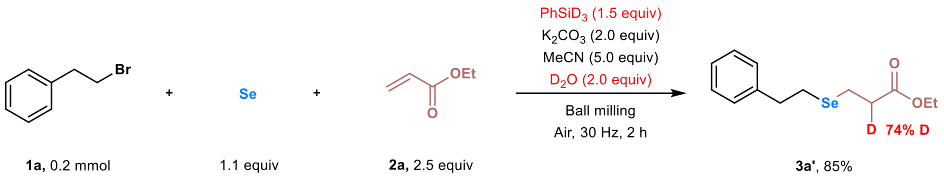

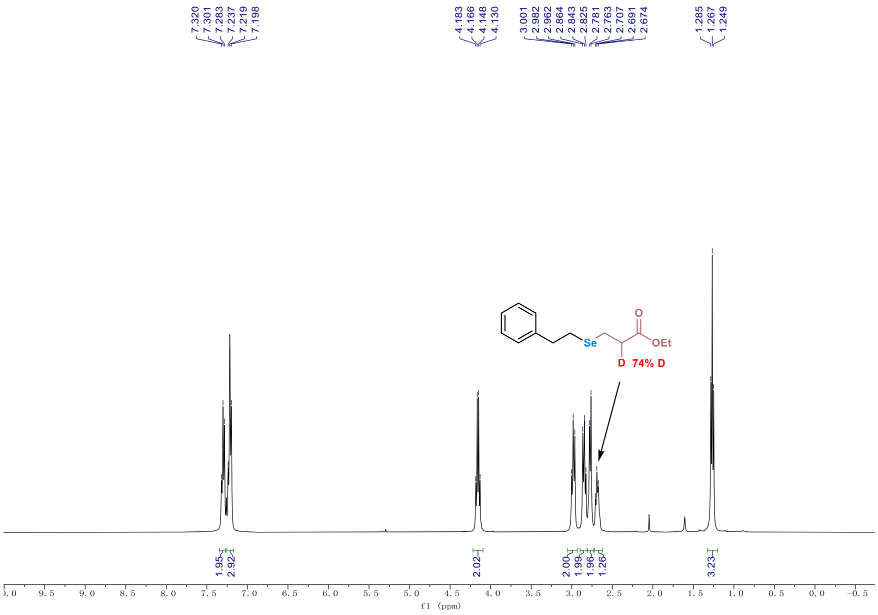

(2-bromoethyl)benzene **1a** (0.2 mmol, 1.0 equiv), 200 mesh Se powder (0.22 mmol, 1.1 equiv), K_2CO_3 (0.4 mmol, 2.0 equiv), CH_3CN (1.0 mmol, 5.0 equiv), ethyl acrylate **2a** (0.5 mmol, 2.5 equiv), and D_2O (0.4 mmol, 2.0 equiv) were combined in a stainless-steel milling jar (10.0 mL) containing two stainless-steel balls (10 mm diameter) under an Ar atmosphere. $PhSiD_3$ (0.3 mmol, 1.5 equiv) was added all at once. After sealing the jar in Ar, it was subjected to grinding at 30 Hz for 2 h. The reaction was then quenched with ethyl acetate, after which the mixture was filtered. The resulting filtrate was concentrated, and the residue was purified by flash silica gel column chromatography, using a petroleum ether/ethyl acetate mixture as the eluent, to yield the D-product **3a'** (51.1 mg, 89% yield, 94% D).

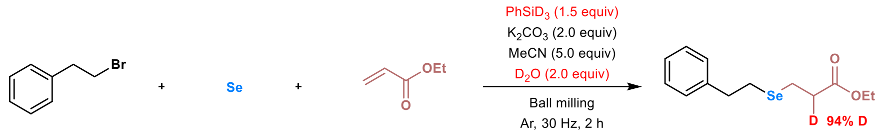

1a, 0.2 mmol 1.1 equiv **2a,** 2.5 equiv **3a'**, 89%

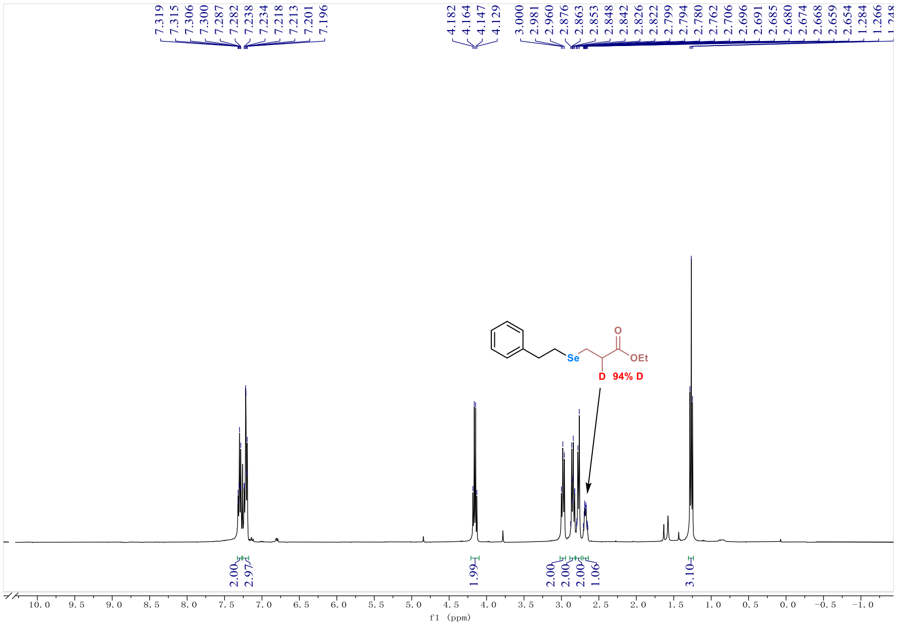

3a (0.2 mmol, 1.0 equiv), 200 mesh Se powder (0.22 mmol, 1.1 equiv), K_2CO_3 (0.4 mmol, 2.0 equiv), CH_3CN (1.0 mmol, 5.0 equiv), and D_2O (2.0 mmol, 10.0 equiv) were combined in a stainless-steel milling jar (10.0 mL) containing two stainless-steel balls (10 mm diameter) under an air atmosphere. $PhSiH_3$ (0.3 mmol, 1.5 equiv) was added all at once. After sealing the jar in air, it was subjected to grinding at 30 Hz for 2 h. The reaction was then quenched with ethyl acetate, after which the mixture was filtered. The resulting filtrate was concentrated, and the residue was purified by flash silica gel column chromatography, using a petroleum ether/ethyl acetate mixture as the eluent, to yield the D-product **3a'** (48.8 mg, 85% yield).

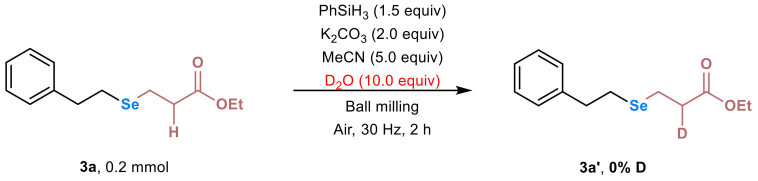

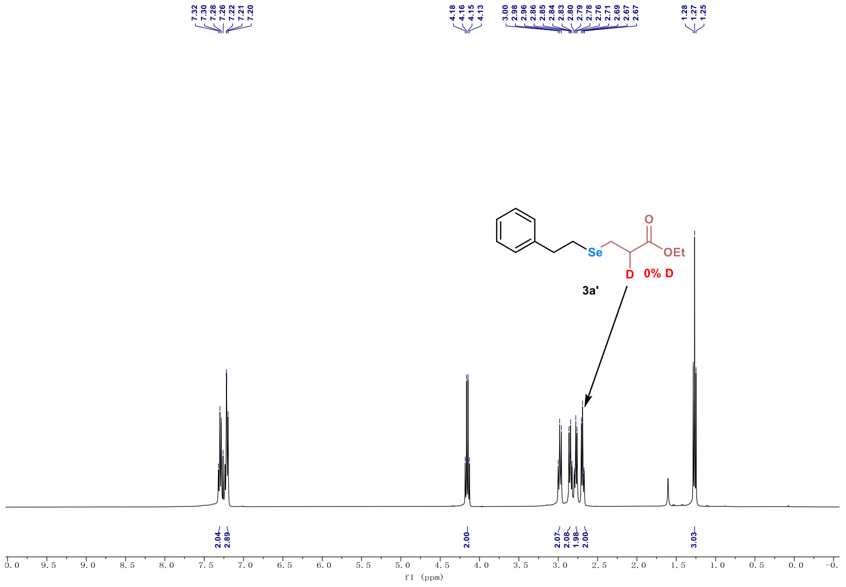

Naphthylsilane-d_3 (0.3 mmol) and H_2O (2.0 equiv) were placed in a stainless-steel milling jar (10.0 mL) containing two stainless-steel balls (10 mm diameter) under an air atmosphere. The jar was sealed and the mixture was subjected to grinding at 30 Hz for 2 h. The reaction mixture was then collected by washing with deuterated chloroform and analyzed by ^1H NMR.

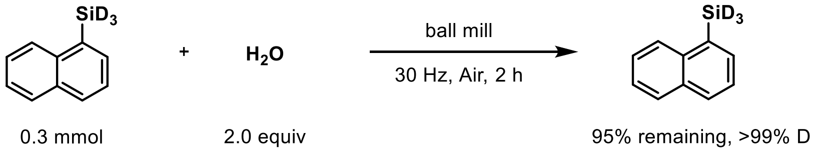

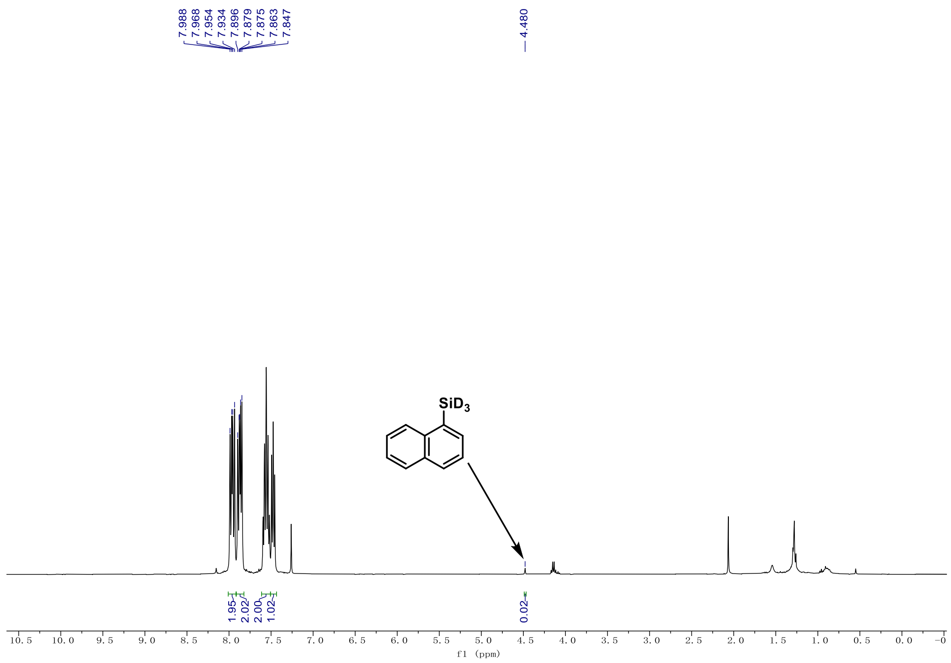

7. DFT calculations

7.1 Computational details

DFT calculations were performed with the Gaussian software.[1] The simulation of the mechanochemical environment is performed under vacuum condition. This treatment can be justified on the fact that these reactions occur on the solid state and the interactions between organic solids are weak, as well as in gas phase. [2-4] All of the structures were optimized in the gas phase using the B3LYP[5] functional in conjunction with Grimme's D3 dispersion correction.[6] The double-ζ basis set (def2-SVP)[7] were chosen for all of the atoms. Optimized geometries and transition states were verified by frequency calculations as minima (zero imaginary frequency) and saddle points (only one imaginary frequency), respectively. Intrinsic Reaction Coordinates (IRC) methods were employed to ensure the transition states indeed connected the corresponding intermediates. Single point energies were calculated at the level of B3LYP-D3/def2-TZVP.

7.2 Energy table

Table S7. Zero-point energy (ZPE), thermal correction to Enthalpy (Hcorr), thermal correction to Gibbs Free Energy (Gcorr), the singlet point energy (E_{SP}), and the imaginary frequency (cm^{-1}) of transition states of optimized structures. Energies are in Hartree.

Structures	ZPE	Hcorr	Gcorr	E_{SP}	Imaginary frequency
H_2Se_2	0.0157	0.0204	-0.0113	-4804.3932	\
HSe_radical	0.0055	0.0088	-0.0145	-2402.1535	\
H_2Se_4	0.0174	0.0262	-0.0196	-9607.6158	\
HSe_2_radical	0.0077	0.0120	-0.0199	-4803.7791	\
TS1	0.1198	0.1321	0.0785	-5326.8221	-609.4
$PhSiH_3$	0.1153	0.1233	0.0830	-523.0668	\
$PhCH_2CH_2Br$	0.1476	0.1569	0.1118	-2884.5908	\
$PhSiH_2$_radical	0.1058	0.1134	0.0743	-522.4161	\

$PhSiH_2Br$	0.1093	0.1183	0.0725	-3096.7056	\
TS2	0.2529	0.2706	0.1987	-3407.0072	-162.5
A	0.1417	0.1503	0.1082	-310.3331	\
B	0.1461	0.1556	0.1095	-2711.9592	\
2a	0.1233	0.1321	0.0907	-345.9395	\
$(PhSiHBr)_2$	0.2032	0.2209	0.1529	-6192.2281	\
TS3	0.2711	0.2891	0.2218	-3057.9044	-220.9
C	0.2714	0.2900	0.2197	-3057.9106	\
TS4	0.3801	0.4075	0.3167	-6154.6138	-1371.3
3a	0.2858	0.3037	0.2385	-3058.5686	\
TS5	0.2932	0.3123	0.2397	-5423.9286	-8.4
7	0.2956	0.3152	0.2405	-5424.0050	\
H_2O	0.0212	0.0250	0.0036	-76.4629	\
TS6	0.4055	0.4328	0.3447	-3656.8519	-553.4
$PhSi(OH)H_2$	0.1219	0.1310	0.0877	-598.3786	\

7.3 Cartesian coordinates

H_2Se_2

Se 0.000000 -1.182078 -0.029716
Se -0.000000 1.182078 -0.029716
H 1.040721 1.358991 1.010347
H -1.040721 -1.358991 1.010347

HSe_radical

H -0.000000 -0.000000 -1.437343
Se 0.000000 0.000000 0.042275

H_2Se_4

Se 0.990030 -0.863056 0.648277
Se -0.990107 -0.863223 -0.648199
Se 2.386498 0.806885 -0.282442
Se -2.386420 0.807010 0.282379
H 1.895965 1.910607 0.576478
H -1.896016 1.910465 -0.576975

HSe_2_radical

Se 0.021271 1.157844 -0.000000
Se 0.021271 -1.117320 0.000000
H -1.446428 -1.377828 0.000000

TS1

Se -2.155107 0.441341 0.442709
Se -1.051365 -1.419266 -0.457278
H 0.008558 -1.528781 0.573559
Si 0.563670 2.436017 -0.607157
H 0.754549 2.872256 -2.020126
C 1.688574 1.026153 -0.132910
C 1.836780 0.661071 1.222626
C 2.294260 0.210392 -1.111482
C 2.564717 -0.474265 1.584477
H 1.366615 1.262534 2.006342
C 3.029027 -0.921099 -0.750041
H 2.184762 0.457105 -2.171476
C 3.162718 -1.268744 0.598240
H 2.666999 -0.741936 2.639405
H 3.492708 -1.538755 -1.523551
H 3.731569 -2.157999 0.881787
H 0.626030 3.574240 0.354851
H -1.159568 1.545461 -0.210711

$PhSiH_3$

Si -2.352678 0.000016 0.006442
H -2.865957 -1.215490 -0.690811
H -2.865111 1.224627 -0.675275
H -2.880615 -0.009014 1.404523
C -0.468635 0.000055 -0.015454
C 0.256331 -1.207316 -0.010788
C 0.256374 1.207330 -0.010816
C 1.654329 -1.209705 0.003466
H -0.274461 -2.164473 -0.023087
C 1.654436 1.209635 0.003484
H -0.274391 2.164485 -0.023098
C 2.355950 -0.000031 0.011323
H 2.198035 -2.158194 0.005140
H 2.198151 2.158118 0.005162
H 3.449138 -0.000096 0.019978

$PhCH_2CH_2Br$

C	1.318255	-0.000034	0.415669
C	2.008525	1.206500	0.217849
C	2.008559	-1.206516	0.217812
C	3.353371	1.208780	-0.162323
H	1.485606	2.155539	0.369981
C	3.353426	-1.208745	-0.162352
H	1.485700	-2.155594	0.369909
C	4.030083	0.000026	-0.353868
H	3.876086	2.157977	-0.306190
H	3.876141	-2.157939	-0.306239
H	5.082627	0.000066	-0.648283
C	-0.152312	-0.000048	0.775260
C	-1.007478	0.000024	-0.490582
H	-0.403373	0.885511	1.379838
H	-0.403381	-0.885670	1.379743
H	-0.824732	0.892790	-1.103505
H	-0.824731	-0.892671	-1.103606
Br	-2.937844	0.000002	-0.079327

$PhSiH_2$_radical

Si	-2.388642	-0.000000	-0.080728
H	-2.965307	1.236310	0.528296
H	-2.964872	-1.236400	0.528556
C	-0.523575	-0.000040	-0.005587
C	0.204526	1.210491	-0.006197
C	0.204567	-1.210525	-0.006201
C	1.600612	1.210581	0.006333
H	-0.326886	2.167206	-0.009026
C	1.600664	-1.210534	0.006296
H	-0.326835	-2.167239	-0.009008
C	2.303092	0.000030	0.012491
H	2.144677	2.158868	0.013230
H	2.144771	-2.158798	0.013185
H	3.396119	0.000044	0.022153

$PhSiH_2Br$

Si	0.805440	0.022458	1.172167
H	1.052198	-1.188672	1.997895
H	1.050478	1.264373	1.951356
C	-0.935851	0.008939	0.491072
C	-1.599782	-1.206223	0.235100
C	-1.605093	1.213483	0.201160
C	-2.892765	-1.217664	-0.294583
H	-1.104231	-2.157731	0.451178
C	-2.898133	1.204363	-0.328545
H	-1.113740	2.172825	0.390493
C	-3.542852	-0.011787	-0.577183
H	-3.395335	-2.169190	-0.486640
H	-3.404874	2.147911	-0.547167
H	-4.554613	-0.019830	-0.991067
Br	2.315452	-0.008879	-0.501100

TS2

C	-3.621513	0.580417	0.065185
C	-4.391000	0.464541	1.234126
C	-4.280180	0.847244	-1.145975
C	-5.779595	0.616820	1.196074
H	-3.892819	0.258149	2.186202
C	-5.668584	1.000171	-1.188134
H	-3.694843	0.941864	-2.065568
C	-6.423329	0.884704	-0.016337
H	-6.361427	0.529306	2.117449
H	-6.163228	1.213759	-2.139359
H	-7.509077	1.006220	-0.047351
C	-2.121689	0.363428	0.100041
C	-1.799335	-1.104682	-0.119144
H	-1.709383	0.687346	1.068698
H	-1.628438	0.966087	-0.678862
H	-2.179495	-1.761247	0.673748
H	-2.098854	-1.477540	-1.106842
Br	0.288910	-1.401176	-0.072649
Si	3.081442	-1.754689	-0.000011
H	3.380657	-2.518957	1.244351
H	3.449394	-2.513474	-1.229219
C	3.841249	-0.054178	0.024927
C	4.066926	0.621685	1.243695
C	4.137976	0.625581	-1.176321
C	4.581676	1.919346	1.261075
H	3.841171	0.125660	2.192676
C	4.652762	1.923243	-1.159255
H	3.968373	0.132630	-2.138507
C	4.876246	2.573879	0.059566
H	4.755246	2.423436	2.215578
H	4.882041	2.430371	-2.100300
H	5.278994	3.590006	0.073031

A

C	0.496964	0.000018	-0.315143
C	-0.207409	1.206112	-0.175901
C	-0.207380	-1.206091	-0.175929
C	-1.579395	1.208194	0.090502

H	0.326496	2.155292	-0.281957
C	-1.579369	-1.208212	0.090477
H	0.326541	-2.155260	-0.282004
C	-2.270702	-0.000020	0.225513
H	-2.112031	2.157847	0.189855
H	-2.111976	-2.157883	0.189808
H	-3.344050	-0.000030	0.431806
C	2.004099	0.000036	-0.556065
C	2.764527	-0.000035	0.734057
H	2.270635	0.887519	-1.154957
H	2.270636	-0.887379	-1.155060
H	2.922928	0.935089	1.278735
H	2.922809	-0.935201	1.278699

B

C	1.304566	-0.000046	0.430865
C	1.993196	1.205797	0.223406
C	1.993243	-1.205823	0.223354
C	3.332446	1.208458	-0.176485
H	1.472971	2.155201	0.383132
C	3.332517	-1.208409	-0.176533
H	1.473093	-2.155276	0.383030
C	4.006812	0.000036	-0.378036
H	3.852986	2.157842	-0.327619
H	3.853066	-2.157783	-0.327699
H	5.054994	0.000088	-0.687769
C	-0.161364	-0.000069	0.807498
C	-1.057298	0.000040	-0.438864
H	-0.398012	0.883494	1.421619
H	-0.398023	-0.883730	1.421475
H	-0.856643	0.879659	-1.074537
H	-0.856634	-0.879463	-1.074696
Se	-2.990074	0.000002	-0.094358

2a

C	0.605315	0.217163	0.000109
O	0.717784	1.421581	-0.000148
O	-0.582050	-0.419458	0.000385
C	1.730273	-0.758554	0.000113
C	2.999333	-0.338307	-0.000226
H	1.462171	-1.818328	0.000344
H	3.836949	-1.040260	-0.000303
H	3.218642	0.733727	-0.000476
C	-1.755418	0.409247	0.000304
C	-2.965491	-0.501256	-0.000429
H	-1.729818	1.067614	-0.884268
H	-1.730404	1.066901	0.885435
H	-3.889785	0.097143	-0.000435
H	-2.968594	-1.146379	-0.892585
H	-2.969109	-1.147152	0.891165

$(PhSiHBr)_2$

Si	1.065024	0.151361	-1.251359
H	0.847663	0.342382	-2.713103
C	0.637164	1.728424	-0.331761
C	1.049551	1.927193	0.999840
C	-0.226895	2.670961	-0.919834
C	0.605208	3.036515	1.723378
H	1.718001	1.205426	1.476463
C	-0.672257	3.780764	-0.195622
H	-0.563934	2.538121	-1.952207
C	-0.258487	3.962949	1.127613
H	0.932412	3.178669	2.756582
H	-1.343050	4.504637	-0.665605
H	-0.606299	4.830065	1.695238
Si	-0.317308	-1.546414	-0.373389
H	-0.193346	-2.858953	-1.064427
C	-2.099508	-0.961683	-0.421273
C	-3.042661	-1.600549	-1.248169
C	-2.505129	0.168223	0.317514
C	-4.354367	-1.123670	-1.337218
H	-2.755575	-2.483502	-1.827366
C	-3.815048	0.643285	0.229559
H	-1.792548	0.682187	0.968028
C	-4.741056	-0.001095	-0.598938
H	-5.076768	-1.632379	-1.980903
H	-4.113913	1.520945	0.808416
H	-5.766642	0.371301	-0.666845
Br	3.222174	-0.412093	-0.920121
Br	0.343166	-1.818080	1.772740

TS3

C	1.997424	-0.708170	-0.336138
C	2.996183	-1.629464	-0.688516
C	2.339347	0.363018	0.506723
C	4.303114	-1.485947	-0.214186
H	2.746438	-2.466165	-1.348196
C	3.646310	0.509555	0.981755
H	1.571232	1.092721	0.777300
C	4.632298	-0.415303	0.623860
H	5.069299	-2.210478	-0.502884
H	3.896358	1.352693	1.631452

H	5.654738	-0.300594	0.992937
C	0.569314	-0.883732	-0.802682
C	-0.207467	-1.856395	0.098645
H	0.539310	-1.254162	-1.840229
H	0.065431	0.092706	-0.800274
H	0.177647	-2.884036	-0.004332
H	-0.095848	-1.585180	1.159100
Se	-2.138260	-1.963868	-0.291338
C	-1.830019	1.768401	0.306031
O	-2.766468	2.078789	-0.395023
O	-0.617187	2.365573	0.240619
C	-1.830508	0.694980	1.318645
C	-2.856416	-0.219906	1.376834
H	-0.944234	0.602442	1.950112
H	-2.922452	-0.923653	2.207522
H	-3.770573	-0.010760	0.813999
C	-0.458260	3.397243	-0.748357
C	0.975134	3.882076	-0.688227
H	-1.181164	4.203928	-0.541330
H	-0.716018	2.983092	-1.737251
H	1.133866	4.680411	-1.429585
H	1.210348	4.283627	0.309676
H	1.676964	3.061898	-0.903622

C

C	-3.419048	-0.207686	-0.443157
C	-4.495583	0.391377	0.229497
C	-3.432628	-1.600266	-0.620644
C	-5.558863	-0.378407	0.709776
H	-4.501786	1.476023	0.373878
C	-4.493632	-2.373847	-0.142010
H	-2.602348	-2.082510	-1.145520
C	-5.560762	-1.764640	0.525831
H	-6.391026	0.106693	1.226762
H	-4.489108	-3.456370	-0.294374
H	-6.392698	-2.367726	0.898308
C	-2.241669	0.619017	-0.913678
C	-1.157379	0.700781	0.168313
H	-2.577953	1.635747	-1.176644
H	-1.812229	0.177282	-1.828020
H	-1.559613	1.159331	1.085038
H	-0.794653	-0.307062	0.419484
Se	0.386088	1.780438	-0.440684
C	3.437390	0.017055	0.439672
O	4.123402	0.946647	0.053193
O	3.810448	-1.278815	0.323849
C	2.138407	0.154727	1.083508
C	1.524377	1.475986	1.206533
H	1.594784	-0.752214	1.356515
H	0.854597	1.590945	2.069123
H	2.269711	2.281570	1.175217
C	5.082457	-1.522077	-0.290535
C	5.304408	-3.020198	-0.318331
H	5.867675	-0.997669	0.280519
H	5.084823	-1.084443	-1.303409
H	6.274429	-3.252070	-0.785040
H	5.300901	-3.434593	0.701768
H	4.511842	-3.521407	-0.895336

TS4

C	4.151148	0.363832	-0.093009
C	3.517969	1.277788	-0.955330
C	5.542615	0.446291	0.063299
C	4.259326	2.250860	-1.630366
H	2.435865	1.218337	-1.097353
C	6.286472	1.419764	-0.612332
H	6.050858	-0.258853	0.727829
C	5.646500	2.326740	-1.461192
H	3.750165	2.954410	-2.294819
H	7.369899	1.468910	-0.473676
H	6.225378	3.088582	-1.989849
C	3.343706	-0.699149	0.624626
C	2.787038	-1.749498	-0.346583
H	2.513861	-0.227609	1.174172
H	3.967232	-1.197380	1.383695
H	2.145764	-1.286254	-1.108491
H	3.608846	-2.266628	-0.864935
Se	1.717335	-3.183212	0.506056
C	0.056739	0.106199	0.555933
O	0.283636	0.329090	-0.620163
O	0.056066	1.056070	1.506494
C	-0.352475	-1.203980	1.099939
C	-0.107813	-2.414066	0.261261
H	-0.224481	-1.309772	2.182647
H	-0.776927	-3.241592	0.539454
H	-0.249161	-2.190703	-0.804734
C	0.299528	2.412608	1.083930
C	0.084938	3.309959	2.283473
H	-0.388132	2.655699	0.260096
H	1.328266	2.482931	0.694874

H	0.261775	4.359765	2.002681
H	-0.948648	3.220264	2.650118
H	0.772833	3.046951	3.101796
Si	-3.352671	-0.499005	0.668321
H	-4.382486	-0.647587	1.736348
H	-1.780700	-0.990433	1.074068
C	-3.209972	1.283601	0.117830
C	-2.720145	1.626911	-1.157090
C	-3.504887	2.318451	1.028200
C	-2.543308	2.965475	-1.514533
H	-2.467970	0.841929	-1.873933
C	-3.320565	3.657263	0.672955
H	-3.886553	2.081199	2.026312
C	-2.841789	3.982524	-0.600887
H	-2.163474	3.215377	-2.508266
H	-3.555995	4.448604	1.389217
H	-2.700279	5.029532	-0.881134
Br	-3.866448	-1.841462	-1.063426

3a

C	-2.043444	-0.951304	0.449263
C	-2.997585	-1.772495	-0.172441
C	-4.295419	-1.311647	-0.411331
C	-4.660175	-0.015597	-0.032274
C	-3.718716	0.811529	0.588563
C	-2.421116	0.348169	0.827229
H	-1.682127	0.994037	1.307532
H	-3.996811	1.824481	0.892582
H	-5.675238	0.345984	-0.216378
H	-5.026028	-1.967749	-0.892210
H	-2.719527	-2.788842	-0.468447
C	-0.622640	-1.431544	0.656408
C	0.230212	-1.186594	-0.594508
H	-0.157206	-1.762652	-1.449906
H	0.205811	-0.128545	-0.889615
Se	2.113149	-1.772762	-0.453264
C	2.774813	-0.540809	0.945758
C	2.912362	0.920639	0.524566
C	1.598337	1.676618	0.543866
O	0.703157	1.470678	1.332436
O	1.565779	2.629469	-0.398246
C	0.395782	3.469723	-0.481725
C	-0.651787	2.861638	-1.398262
H	-1.051806	1.928103	-0.977680
H	-0.224268	2.651205	-2.390717
H	-1.493226	3.561504	-1.523862
H	-0.005051	3.632933	0.530201
H	0.768626	4.425443	-0.878042
H	3.382912	1.023843	-0.463208
H	3.563295	1.448125	1.247763
H	2.135289	-0.624723	1.835834
H	3.760178	-0.956745	1.201944
H	-0.609344	-2.507475	0.893817
H	-0.177779	-0.896182	1.507173

TS5

C	2.949963	-0.655341	0.899318
C	2.993627	0.714921	1.201013
C	4.160763	-1.329173	0.670809
C	4.213332	1.396000	1.266959
H	2.062378	1.257177	1.385802
C	5.381456	-0.653034	0.738440
H	4.145168	-2.398931	0.440288
C	5.411059	0.714162	1.035201
H	4.225289	2.463404	1.502161
H	6.314112	-1.195157	0.561271
H	6.365422	1.244188	1.088821
C	1.628564	-1.379005	0.760106
C	1.205926	-1.494793	-0.711502
H	0.845073	-0.845157	1.320551
H	1.694462	-2.389179	1.195706
H	1.169740	-0.498832	-1.195333
H	1.944715	-2.068397	-1.297869
Se	-0.525582	-2.346494	-1.031132
C	-2.975043	0.770020	0.809262
C	-3.111299	-0.588601	1.135639
C	-4.137796	1.527955	0.596502
C	-4.375772	-1.175062	1.244488
H	-2.217403	-1.196348	1.301143
C	-5.403311	0.945728	0.706652
H	-4.048884	2.589590	0.345883
C	-5.525922	-0.409740	1.030258
H	-4.461088	-2.234575	1.499640
H	-6.297538	1.552774	0.542319
H	-6.515079	-0.866414	1.117999
C	-1.607366	1.391598	0.628449
C	-1.156836	1.335759	-0.838622
H	-0.868423	0.865269	1.253420
H	-1.613623	2.441405	0.962519
H	-1.150626	0.295107	-1.214933

H	-1.865850	1.874876	-1.491852
Se	0.595113	2.124165	-1.214614

7

C	4.127037	0.747892	-0.433190
C	5.115721	0.482251	0.527301
C	3.934962	2.075689	-0.846245
C	5.894356	1.514022	1.058718
H	5.278873	-0.547567	0.859102
C	4.711412	3.110521	-0.317358
H	3.169625	2.299421	-1.595620
C	5.694083	2.832630	0.638016
H	6.662914	1.287048	1.802288
H	4.550730	4.137748	-0.655173
H	6.303907	3.640278	1.050816
C	3.248179	-0.363293	-0.966526
C	2.003964	-0.548051	-0.088649
H	3.816989	-1.307394	-1.003231
H	2.940750	-0.133448	-2.000502
H	2.278981	-0.820323	0.940043
H	1.407789	0.375286	-0.055656
Se	0.846747	-2.001225	-0.810389
C	-4.127034	0.747866	0.433165
C	-5.115679	0.482132	-0.527343
C	-3.935095	2.075680	0.846223
C	-5.894400	1.513829	-1.058775
H	-5.278727	-0.547702	-0.859147
C	-4.711633	3.110441	0.317322
H	-3.169792	2.299488	1.595610
C	-5.694256	2.832458	-0.638074
H	-6.662925	1.286784	-1.802358
H	-4.551054	4.137683	0.655141
H	-6.304151	3.640046	-1.050886
C	-3.248088	-0.363236	0.966518
C	-2.003877	-0.547985	0.088611
H	-3.816835	-1.307373	1.003293
H	-2.940636	-0.133316	2.000471
H	-2.278922	-0.820300	-0.940065
H	-1.407748	0.375382	0.055556
Se	-0.846679	-2.001093	0.810449

H_2O

O	0.000000	-0.000000	0.120201
H	-0.000000	0.757066	-0.480805
H	-0.000000	-0.757066	-0.480805

$PhSi(OH)H_2$

Si	1.933526	0.000034	-0.476829
H	2.327065	1.217666	-1.249713
H	2.327098	-1.217512	-1.249834
C	0.081585	0.000012	-0.222282
C	-0.634248	1.208083	-0.117134
C	-0.634229	-1.208074	-0.117172
C	-2.015909	1.210704	0.094261
H	-0.107255	2.163476	-0.202656
C	-2.015890	-1.210724	0.094221
H	-0.107222	-2.163457	-0.202723
C	-2.708025	-0.000017	0.201039
H	-2.554943	2.158504	0.173939
H	-2.554910	-2.158534	0.173868
H	-3.789060	-0.000028	0.364588
O	2.665091	-0.000042	1.028244
H	3.629431	-0.000163	1.044582

TS6

C	3.414476	1.512969	0.190178
C	4.428013	0.729235	-0.382629
C	3.377408	1.635509	1.588304
C	5.375139	0.082085	0.416119
H	4.467938	0.618902	-1.470244
C	4.321763	0.990683	2.392446
H	2.592746	2.241441	2.051140
C	5.323251	0.207314	1.808491
H	6.157857	-0.522604	-0.049917
H	4.276366	1.100117	3.479264
H	6.062564	-0.298159	2.434911
C	2.326672	2.122667	-0.667050
C	1.159834	1.138858	-0.798426
H	2.721523	2.371404	-1.665765
H	1.970581	3.064049	-0.216814
H	1.502475	0.200636	-1.260517
H	0.776802	0.884330	0.200363
Se	-0.347912	1.830004	-1.866754
C	-3.287116	0.539052	0.012997
O	-4.215851	0.614709	-0.782808
O	-3.429232	0.918065	1.309814
C	-1.988679	-0.078616	-0.259456
C	-1.426609	0.153516	-1.642636
H	-1.273431	0.106698	0.547544
H	-0.749683	-0.654205	-1.960502
H	-2.233638	0.242492	-2.386078
C	-4.717597	1.404353	1.699991

C	-4.650110	1.757885	3.172015
H	-5.480207	0.632292	1.496320
H	-4.982595	2.276502	1.079300
H	-5.621986	2.143193	3.518419
H	-4.390546	0.873795	3.775378
H	-3.885588	2.529761	3.350830
Si	-1.978370	-2.470042	0.666488
H	-2.449684	-1.739533	1.867061
C	-0.154930	-2.494610	0.283077
C	0.324839	-3.144468	-0.869656
C	0.769469	-1.834695	1.116145
C	1.687847	-3.129963	-1.182133
H	-0.370072	-3.661161	-1.538901
C	2.132485	-1.827584	0.810587
H	0.423850	-1.304041	2.008419
C	2.591951	-2.472343	-0.342272
H	2.043527	-3.630854	-2.086365
H	2.836959	-1.295529	1.453412
H	3.656186	-2.446688	-0.587230
O	-3.016764	-2.418448	-0.902345
H	-3.931222	-2.754891	-0.916125
H	-2.928046	-1.390552	-0.941263
H	-2.420343	-3.892653	0.882335

8. References

[1] M. J. Frisch, G. W. Trucks, H. B. Schlegel, G. E. Scuseria, M. A. Robb, J. R. Cheeseman, G. Scalmani, V. Barone, B. Mennucci, G. A. Petersson, H. Nakatsuji, M. Caricato, X. Li, H. P. Hratchian, A. F. Izmaylov, J. Bloino, G. Zheng, J. L. Sonnenberg, M. Hada, M. Ehara, K. Toyota, R. Fukuda, J. Hasegawa, M. Ishida, T. Nakajima, Y. Honda, O. Kitao, H. Nakai, T. Vreven, J. A. J. Montgomery, J. E. Peralta, F. Ogliaro, M. Bearpark, J. J. Heyd, E. Brothers, K. N. Kudin, V. N. Staroverov, R. Kobayashi, J. Normand, K. Raghavachari, A. Rendell, J. C. Burant, S. S. Iyengar, J. Tomasi, M. Cossi, N. Rega, M. J. Millam, M. Klene, J. E. Knox, J. B. Cross, V. Bakken, C. Adamo, J. Jaramillo, R. Gomperts, R. E. Stratmann, O. Yazyev, A. J. Austin, R. Cammi, C. Pomelli, J. W. Ochterski, R. L. Martin, K. Morokuma, V. G. Zakrzewski, G. A. Voth, P. Salvador, J. J. Dannenberg, S. Dapprich, A. D. Daniels, Ö. Farkas, J. B. Foresman, J. V. Ortiz, J. Cioslowski, D. J. Fox, *Gaussian 09, Revision D.01;* **2009**, Gaussian.

[2] B. S. Pladevall, A. de Aguirre, F. Maseras, Understanding Ball Milling Mechanochemical Processes with DFT Calculations and Microkinetic Modeling. *ChemSusChem* **2021,** *14*, 2763-2768.

[3] D. Barišić, I. Halasz, A. Bjelopetrović, D. Babić, M. Ćurić, Mechanistic Study of the Mechanochemical PdII-Catalyzed Bromination of Aromatic C–H Bonds by Experimental and Computational Methods. *Organometallics* **2022,** *41*, 1284-1294.

[4] E. Garcia-Padilla, F. Maseras, Understanding the divergence in outcomes between mechanochemical and solution phase reactivity of fullerenes. *Cell Rep. Phys. Sci.* **2025,** *6*, 102434.

[5] P. J. Stephens, F. J. Devlin, C. F. Chabalowski, M. J. Frisch, Ab initio calculation of vibrational absorption and circular dichroism spectra using density functional force fields. *J. Phys. Chem.* **1994**, *98*, 11623-11627.

[6] S. Grimme, J. Antony, S. Ehrlich, H. Krieg, A consistent and accurate ab initio parametrization of density functional dispersion correction (DFT-D) for the 94 elements H-Pu. *J. Chem. Phys.* **2010**, *132*, 154104.

[7] F. Weigend, R. Ahlrichs, Balanced basis sets of split valence, triple zeta valence and quadruple zeta valence quality for H to Rn: Design and assessment of accuracy. *Phys. Chem. Chem. Phys.* **2005**, *7*, 3297-3305.

9. NMR spectra

****

^{1}H NMR of 3a (400 MHz, Chloroform-*d*)

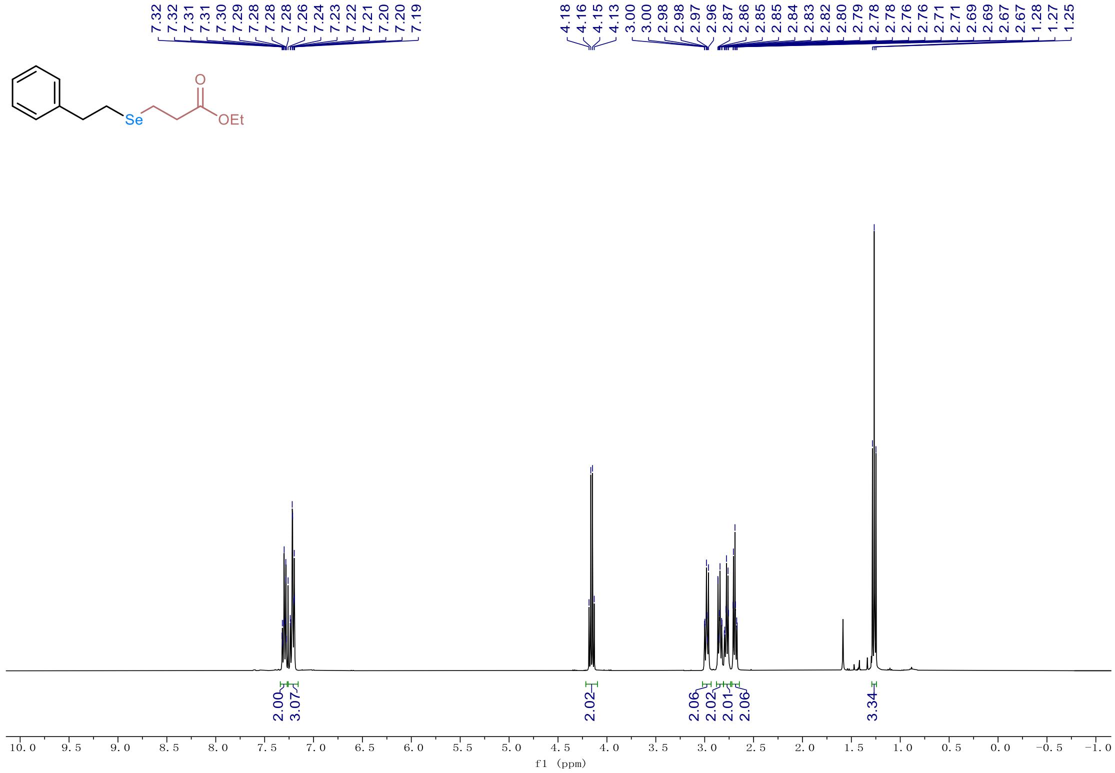

^{13}C NMR of 3a (101 MHz, Chloroform-*d*)

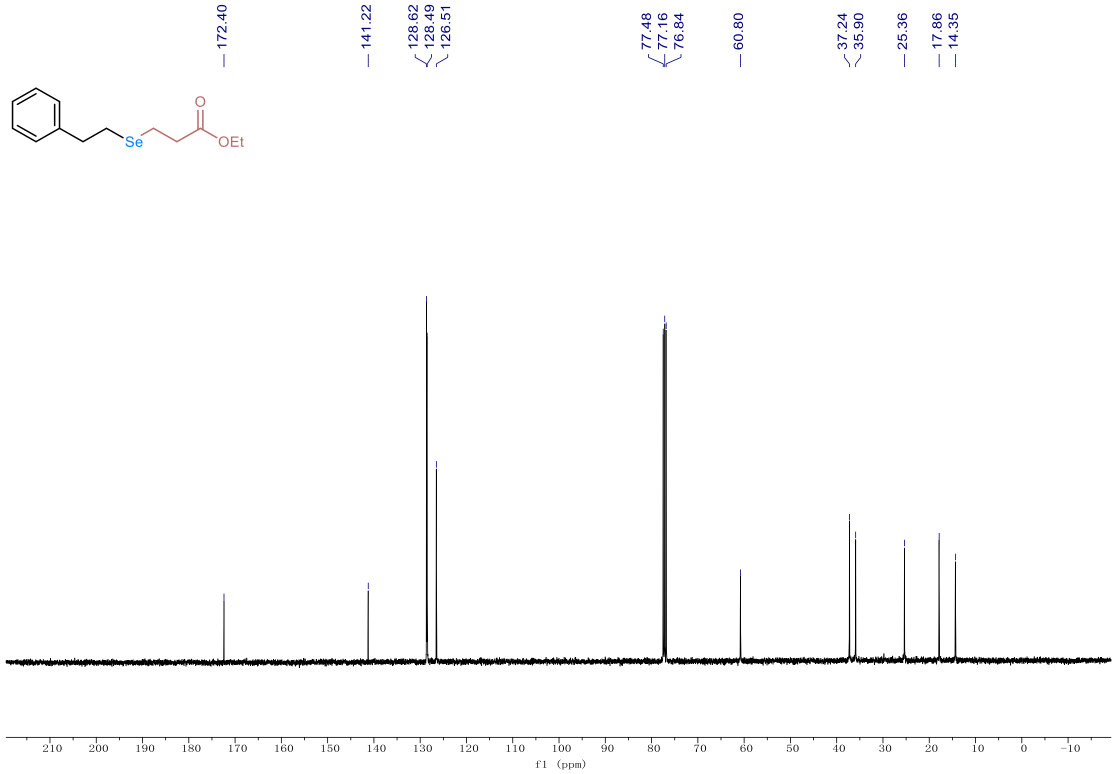

^{1}H NMR of 3b (400 MHz, Chloroform-*d*)

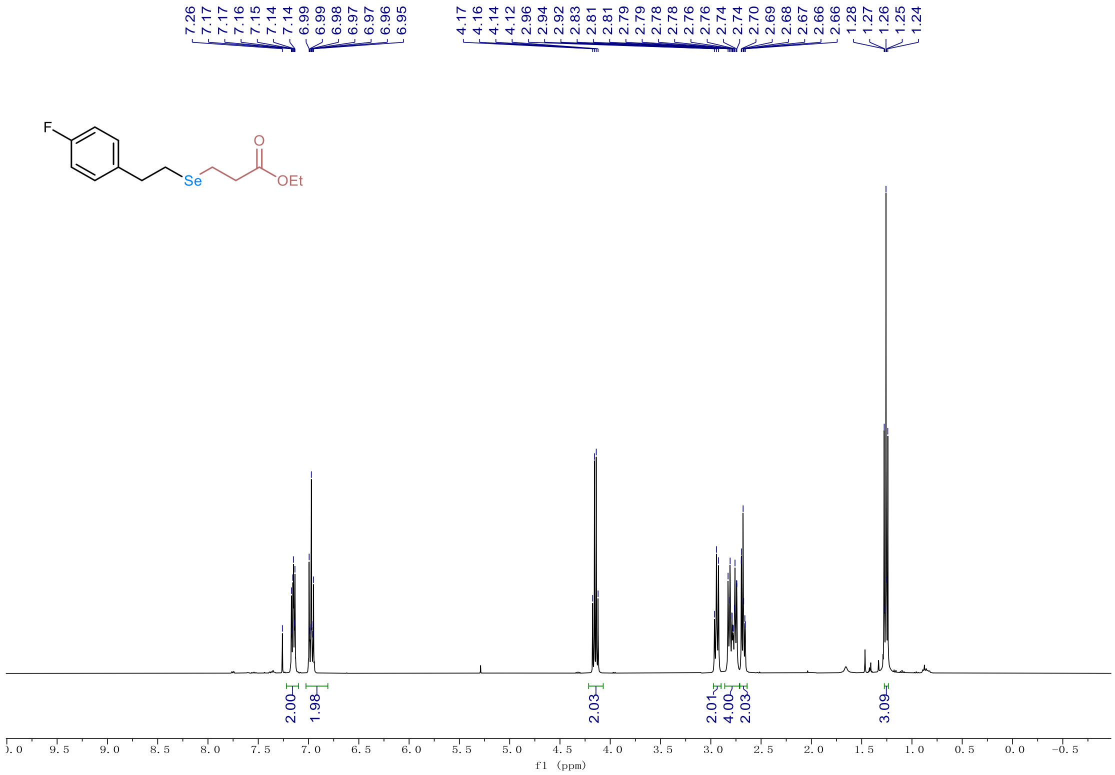

^{13}C NMR of 3b (101 MHz, Chloroform-*d*)

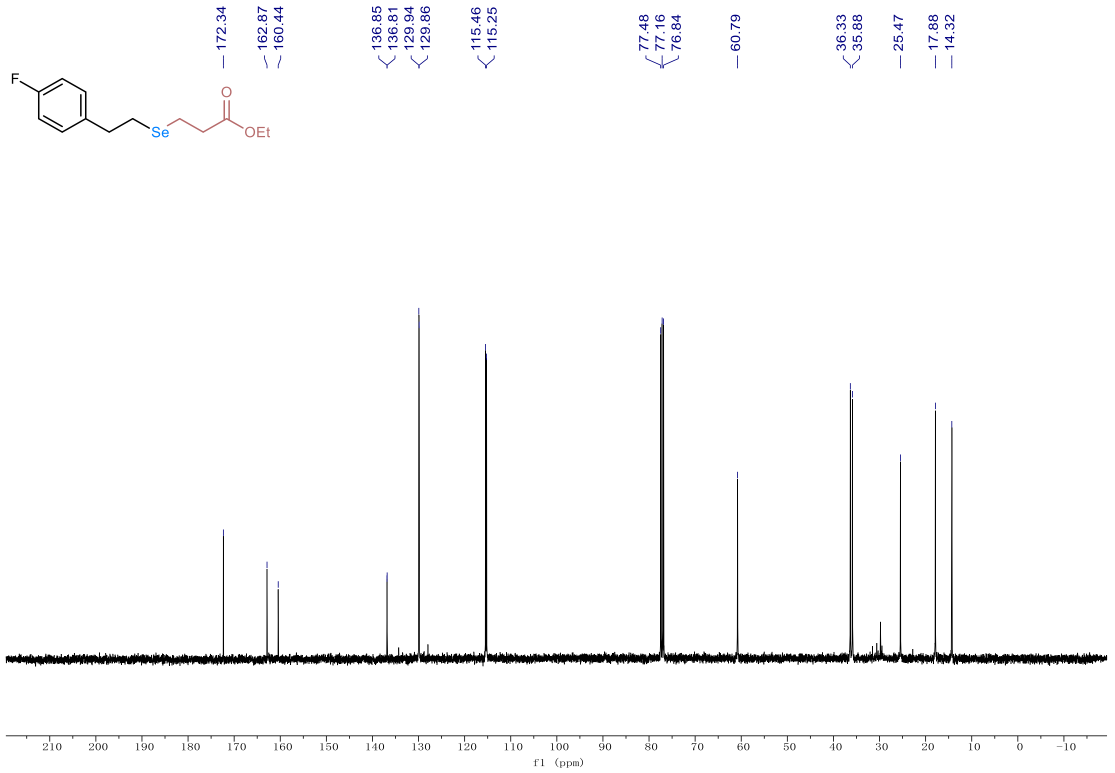

^{19}F NMR of 3b (376 MHz, Chloroform-*d*)

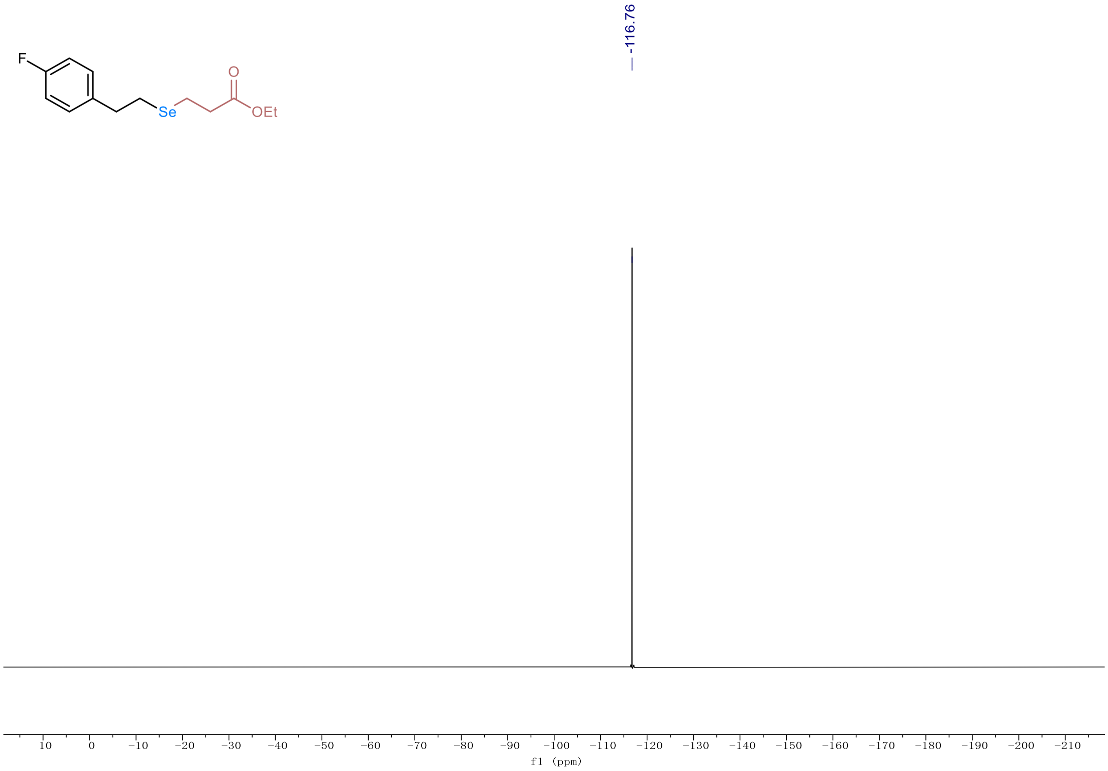

^{1}H NMR of 3c (400 MHz, Chloroform-*d*)

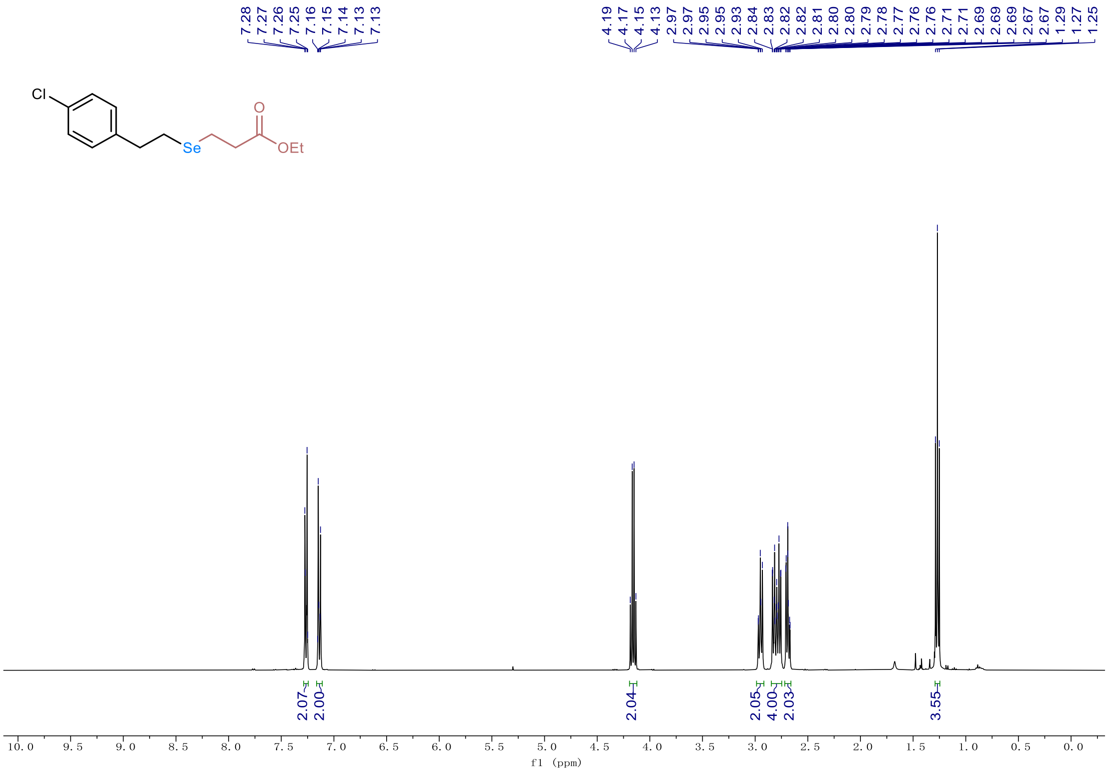

^{13}C NMR of 3c (101 MHz, Chloroform-*d*)

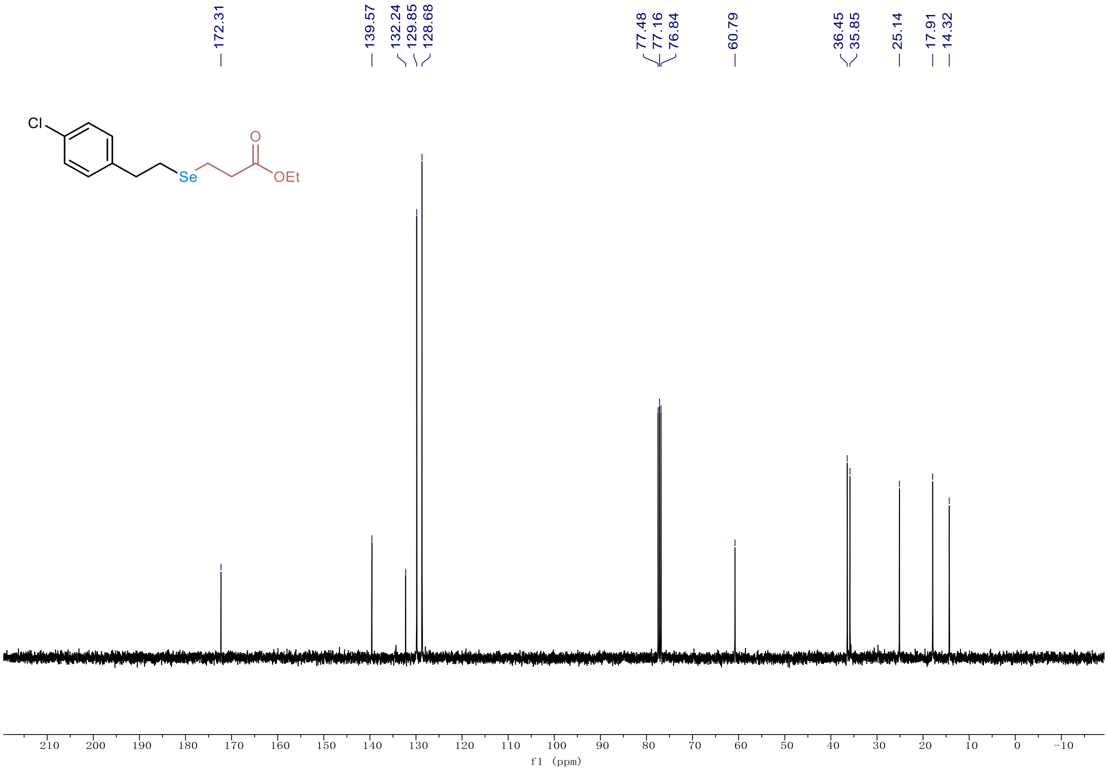

^{1}H NMR of 3d (400 MHz, Chloroform-*d*)

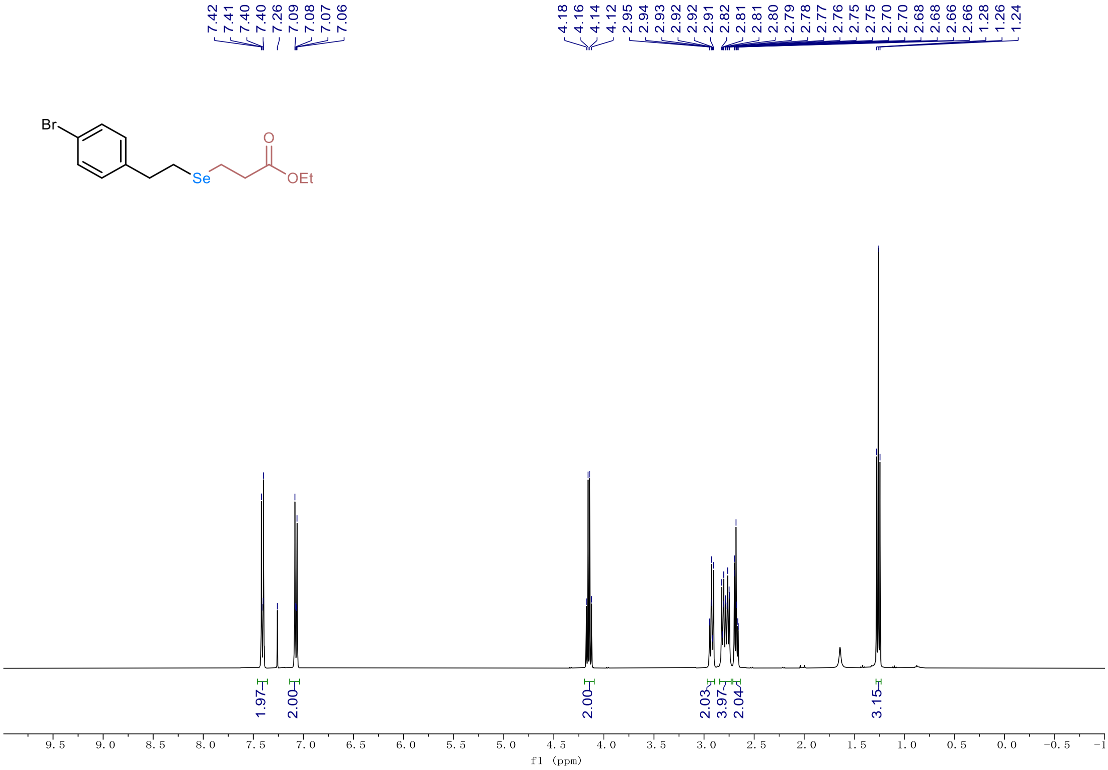

^{13}C NMR of 3d (101 MHz, Chloroform-*d*)

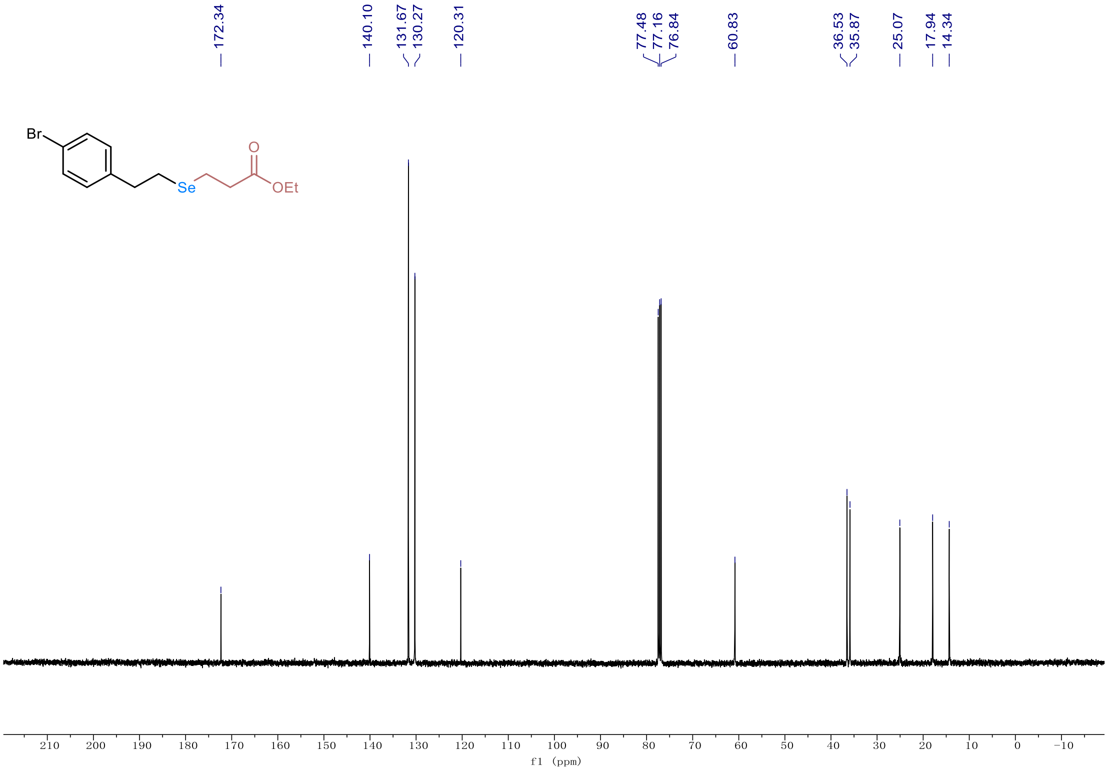

^{1}H NMR of 3e (400 MHz, Chloroform-*d*)

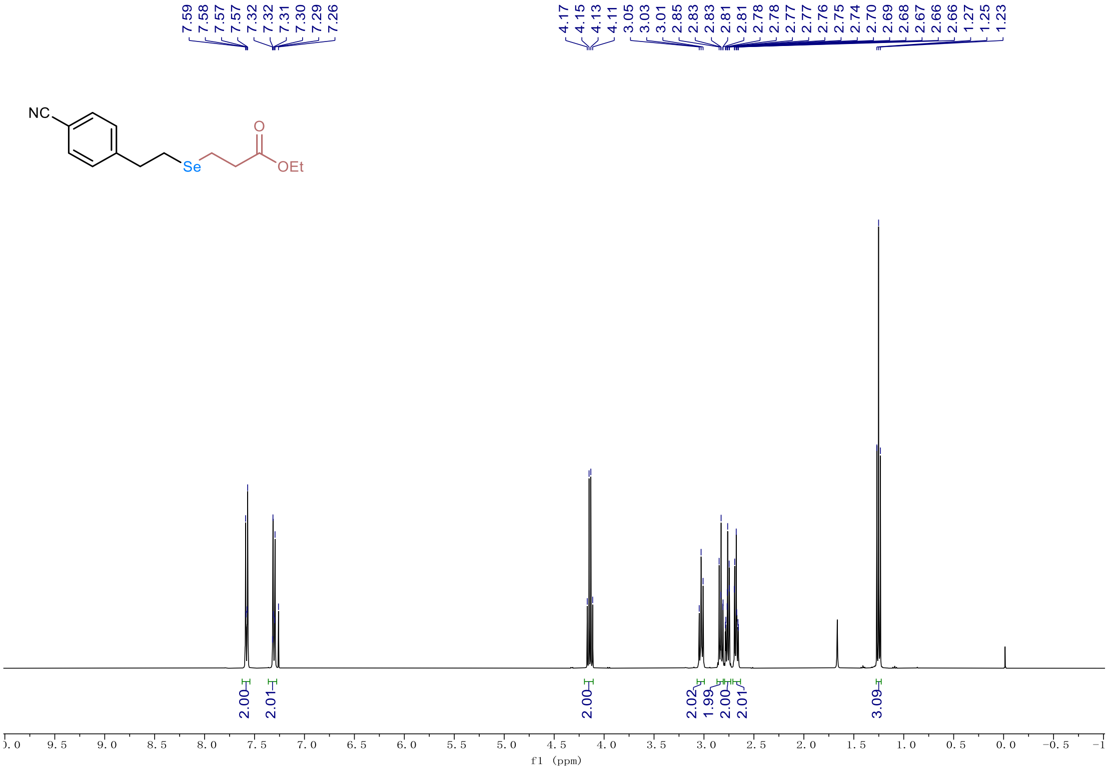

^{13}C NMR of 3e (101 MHz, Chloroform-*d*)

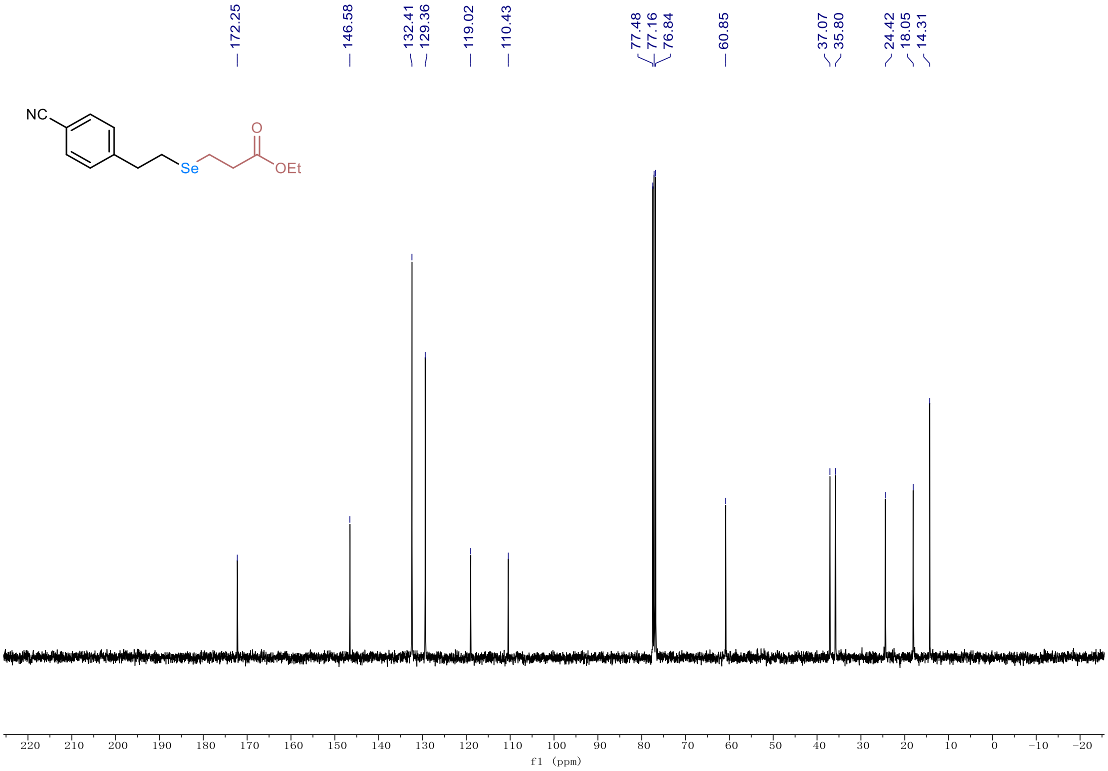

^{1}H NMR of 3f (400 MHz, Chloroform-*d*)

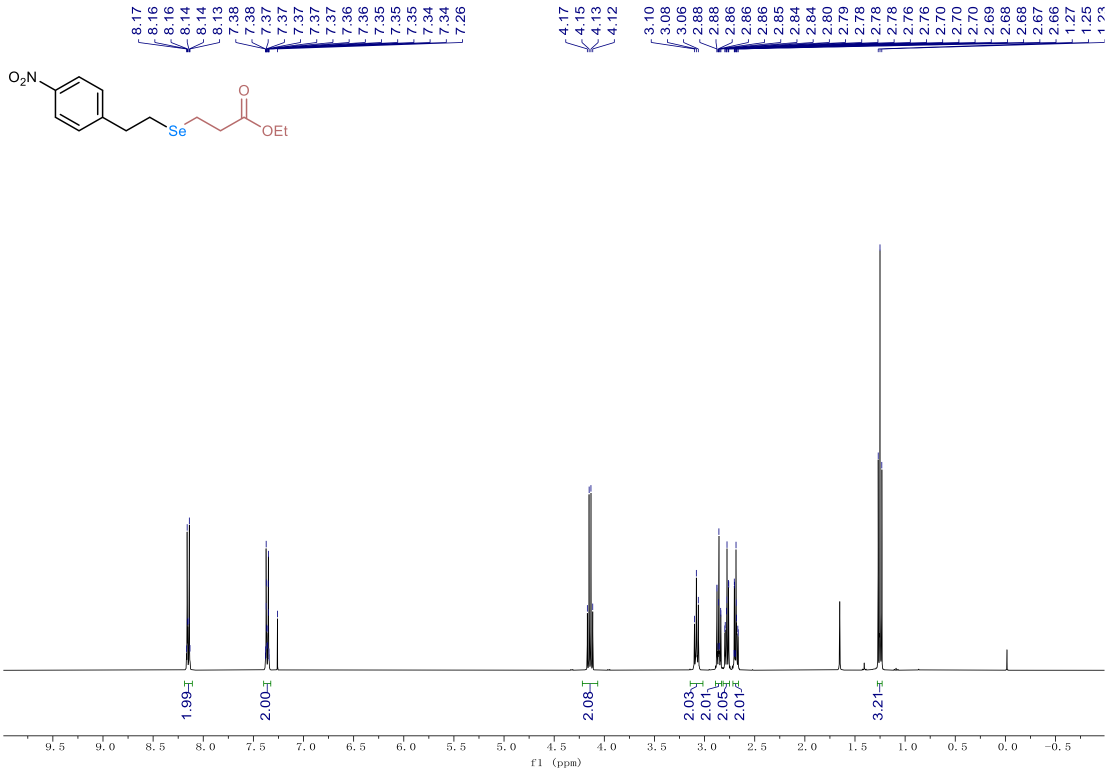

^{13}C NMR of 3f (101 MHz, Chloroform-*d*)

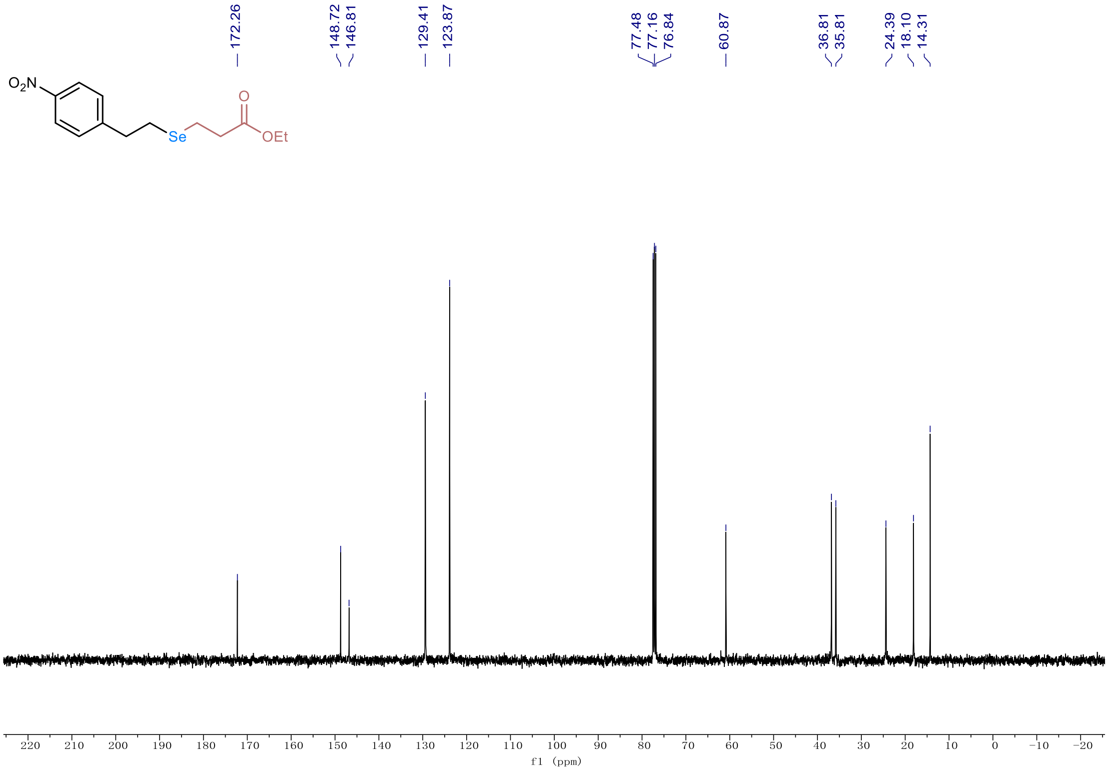

^{1}H NMR of 3g (400 MHz, Chloroform-*d*)

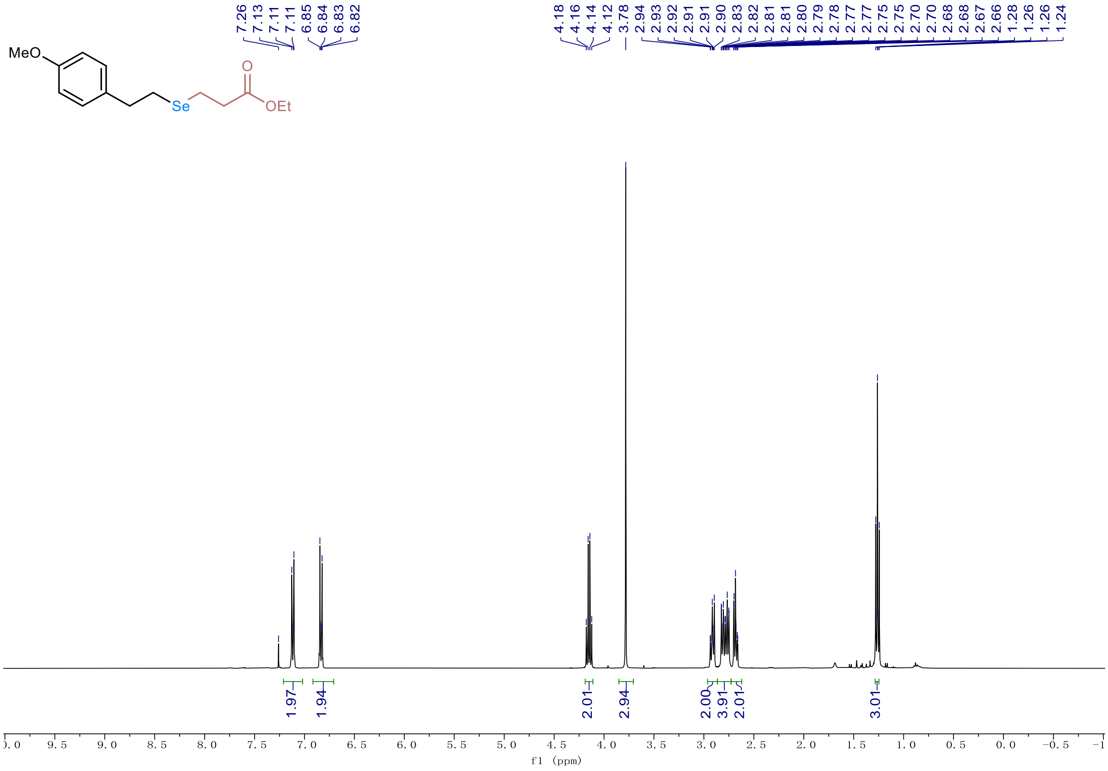

^{13}C NMR of 3g (101 MHz, Chloroform-*d*)

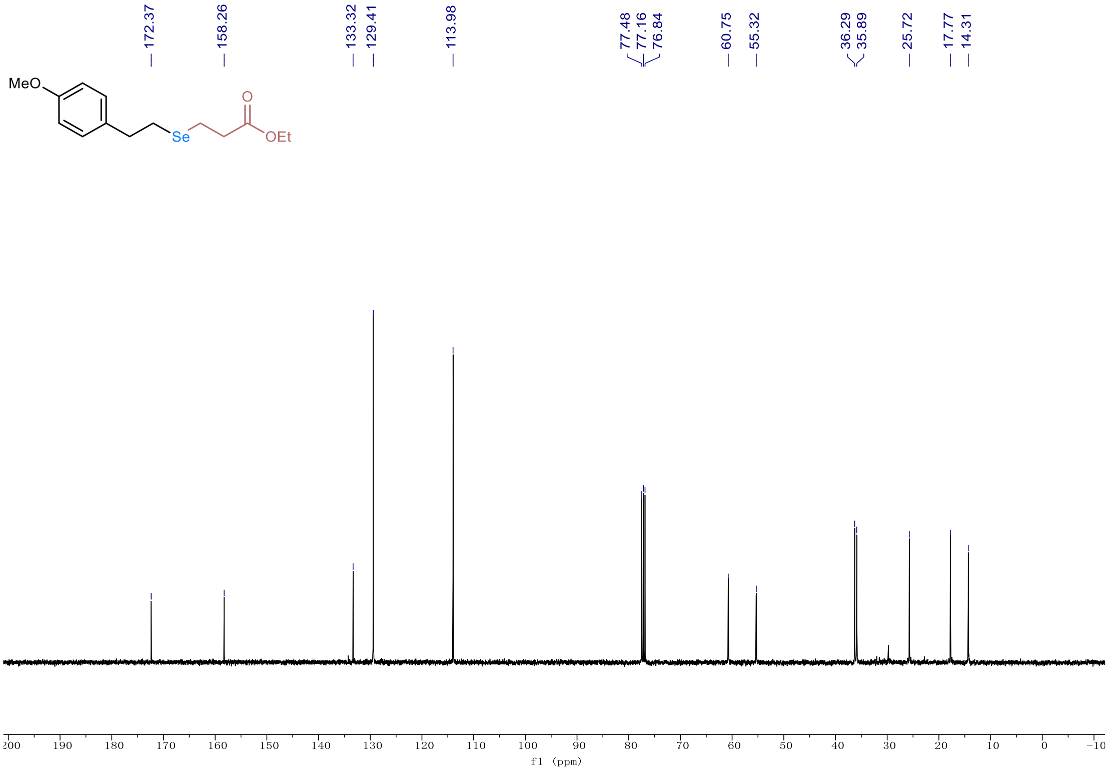

^{1}H NMR of 3h (400 MHz, Chloroform-*d*)

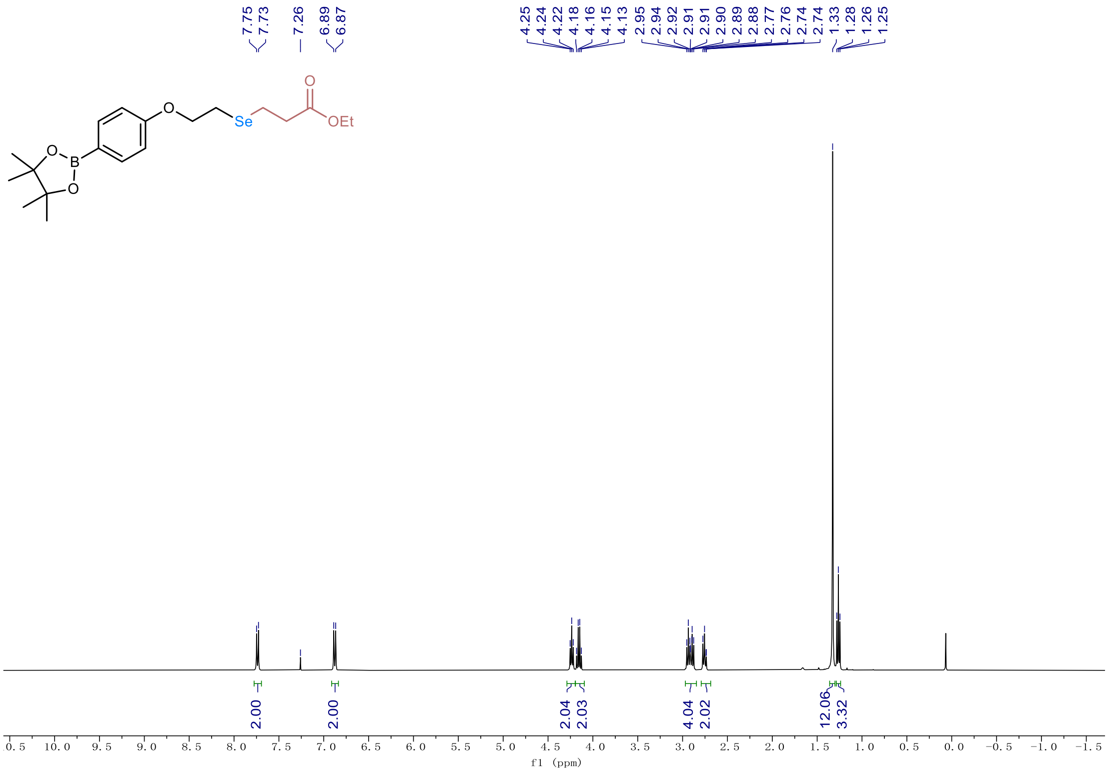

^{13}C NMR of 3h (101 MHz, Chloroform-*d*)

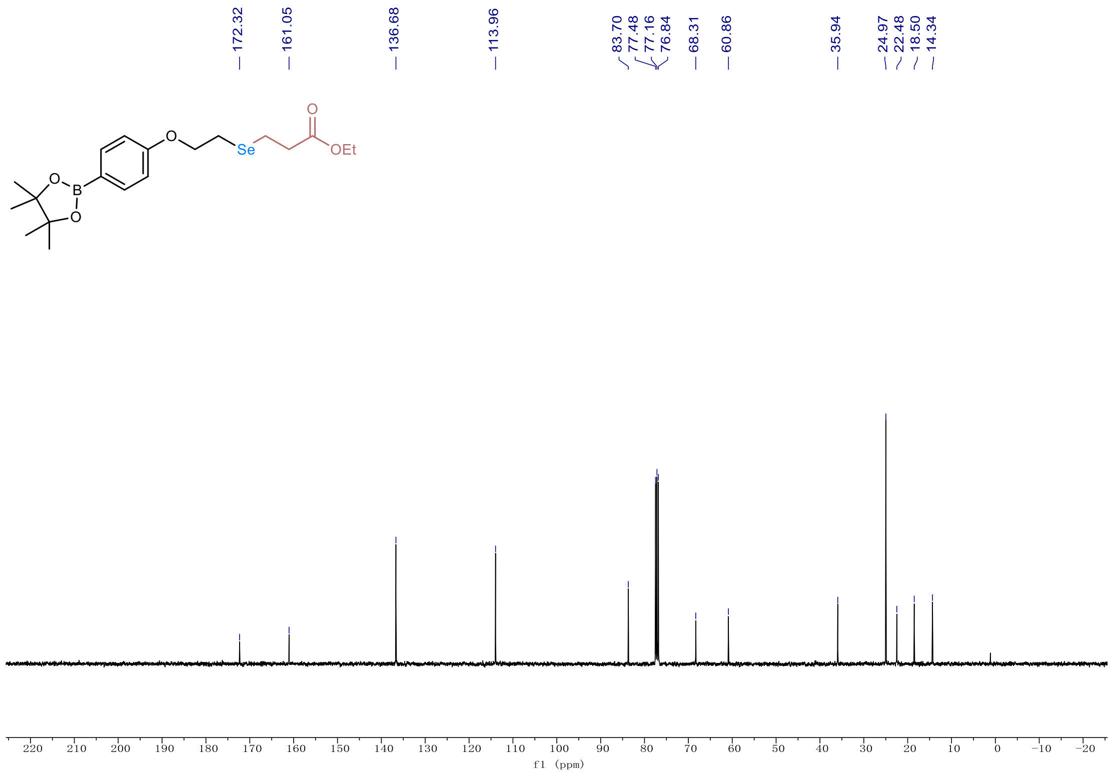

^{1}H NMR of 3i (400 MHz, Chloroform-*d*)

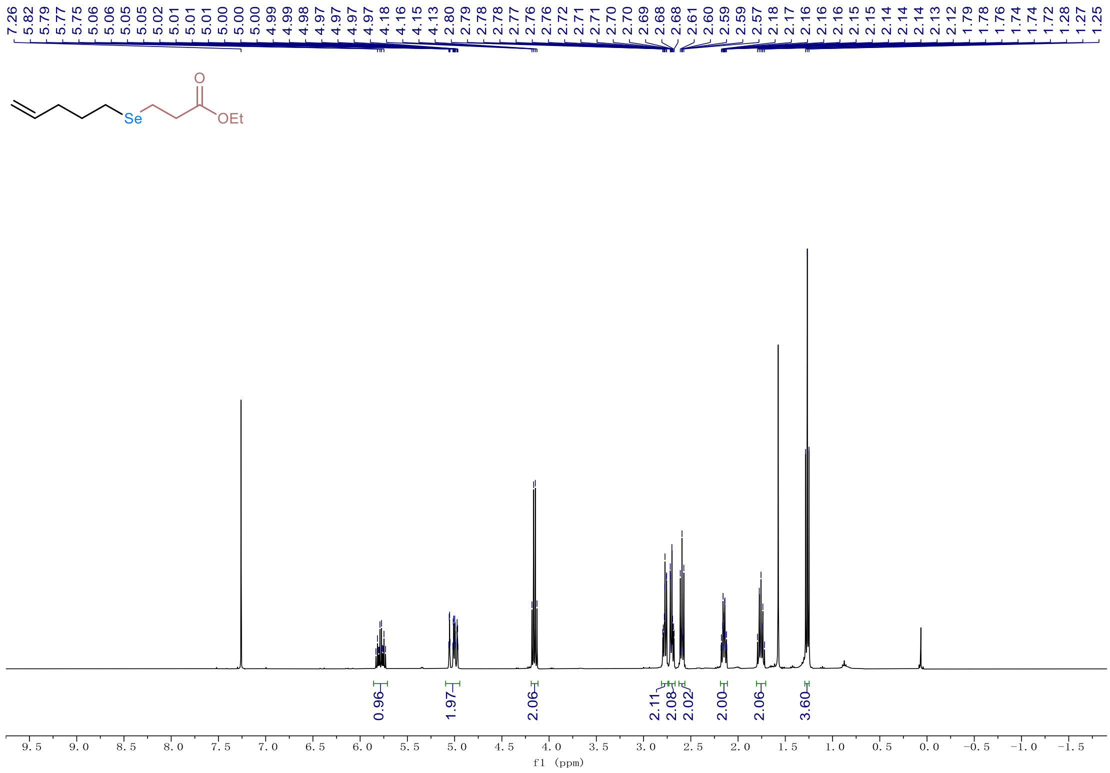

^{13}C NMR of 3i (101 MHz, Chloroform-*d*)

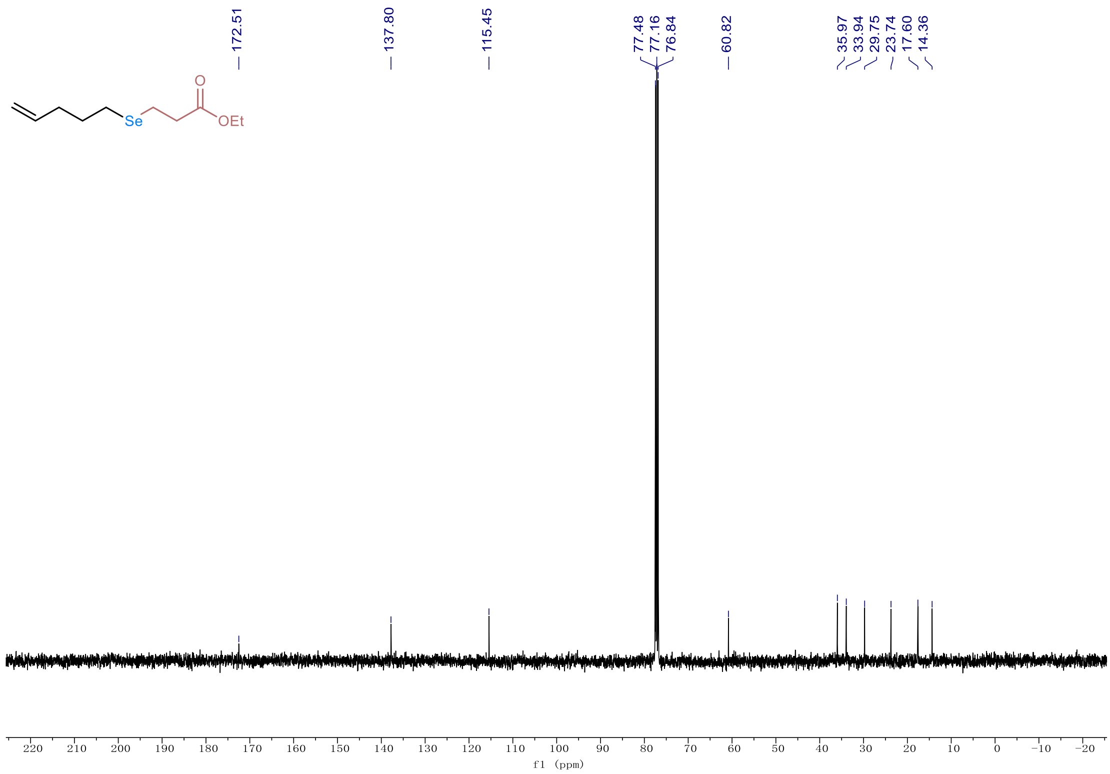

^{1}H NMR of 3j (400 MHz, Chloroform-*d*)

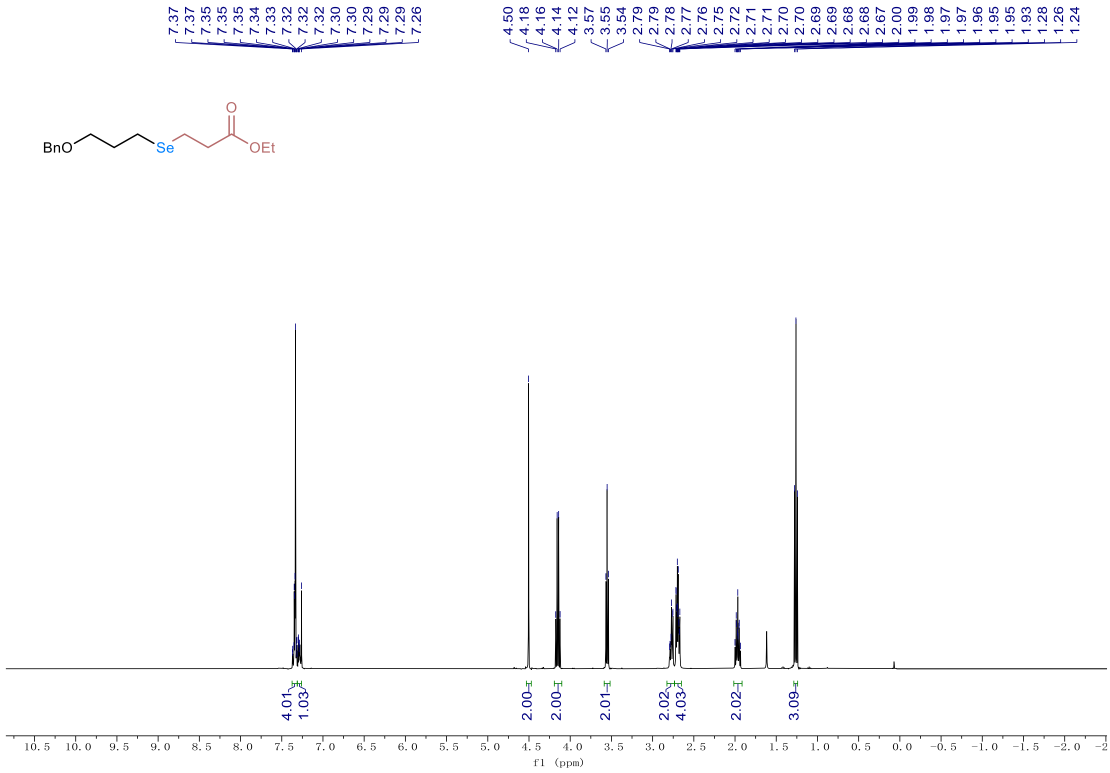

^{13}C NMR of 3j (101 MHz, Chloroform-*d*)

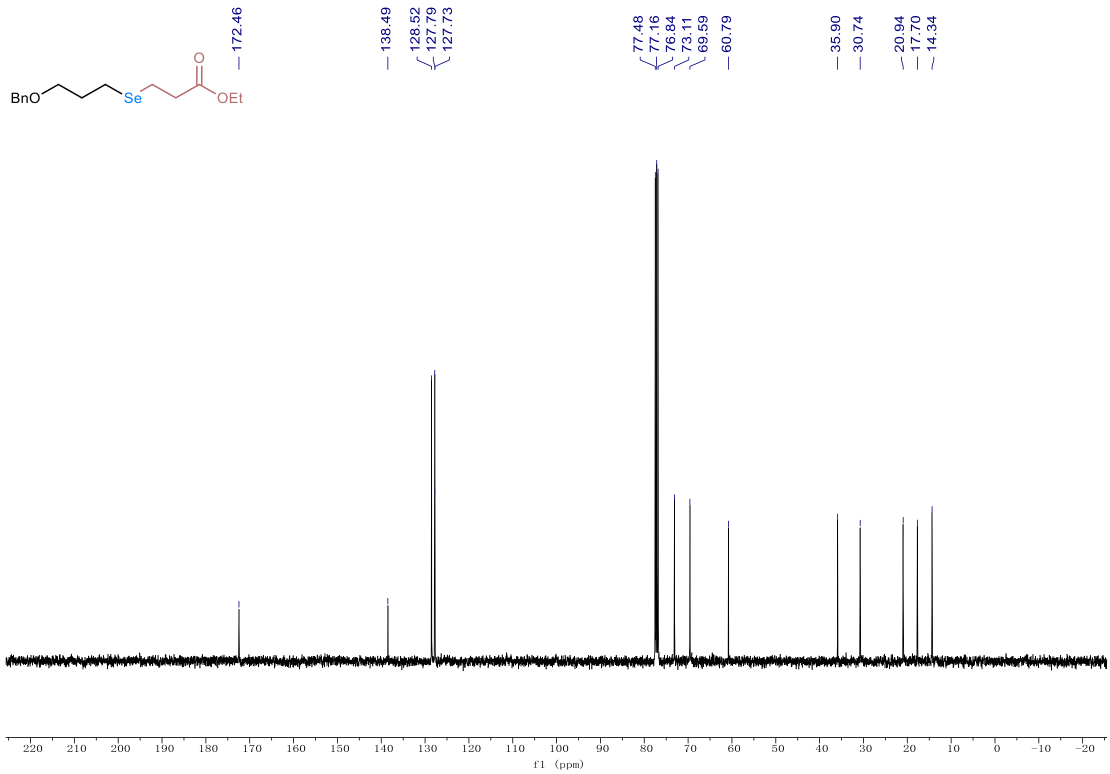

^{1}H NMR of 3k (400 MHz, Chloroform-*d*)

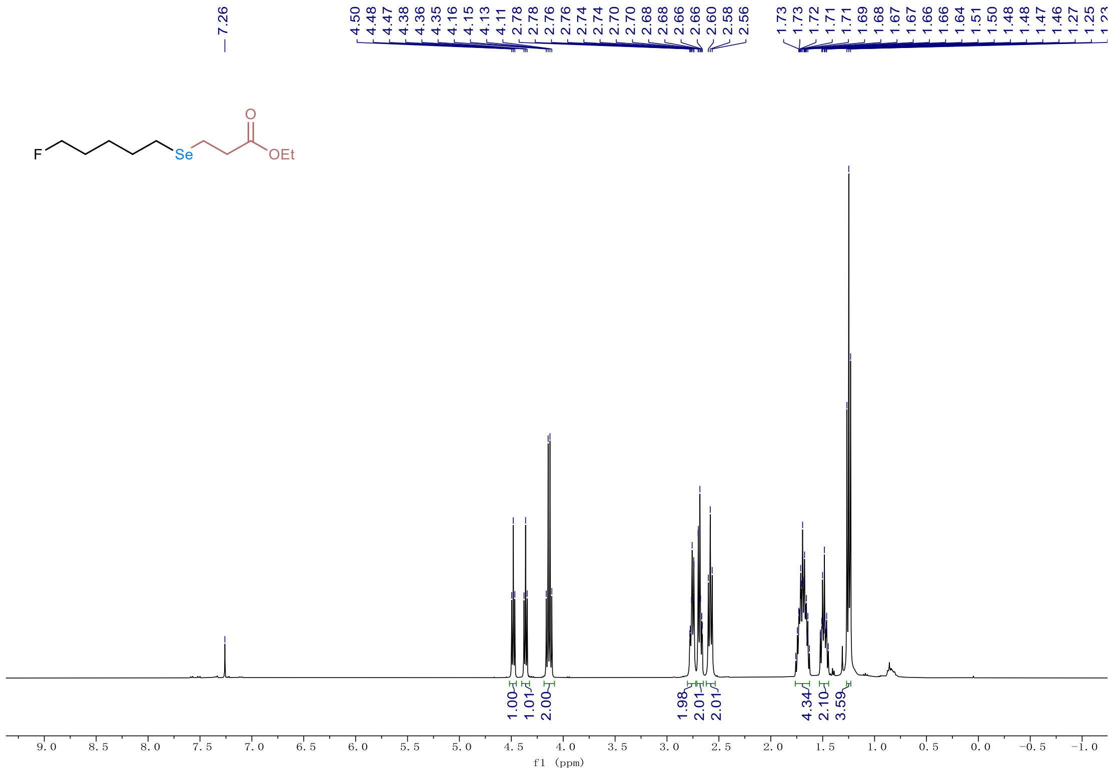

^{13}C NMR of 3k (101 MHz, Chloroform-*d*)

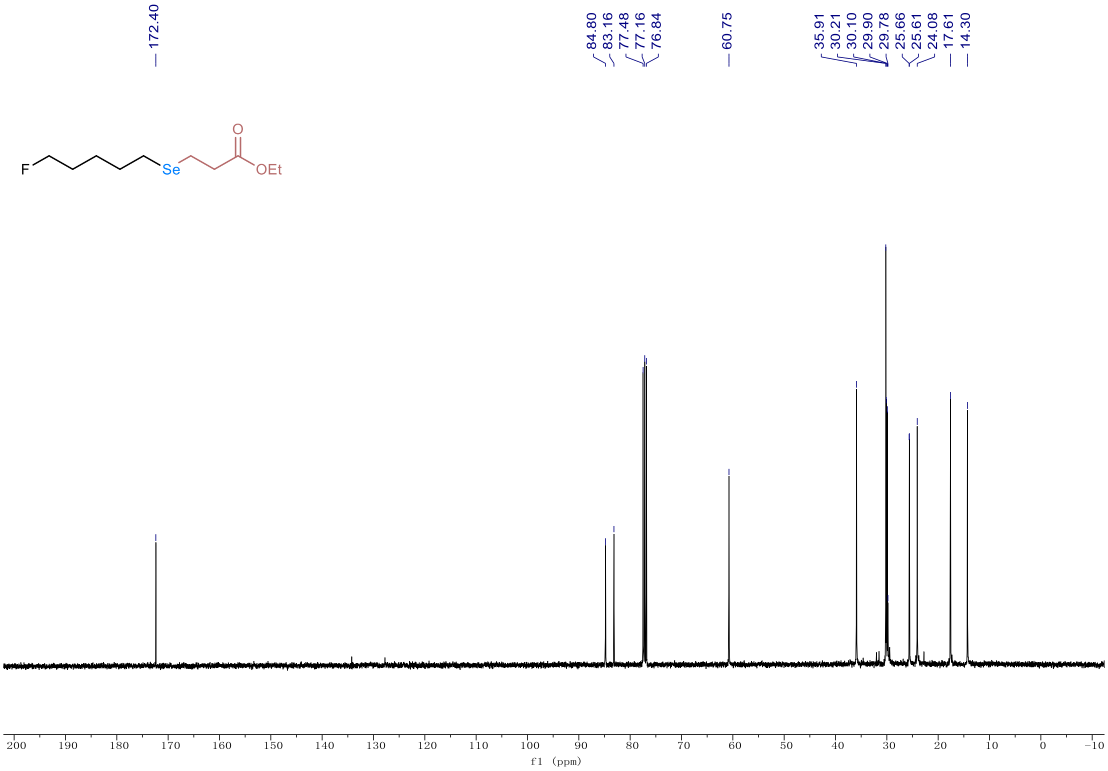

^{19}F NMR of 3k (376 MHz, Chloroform-*d*)

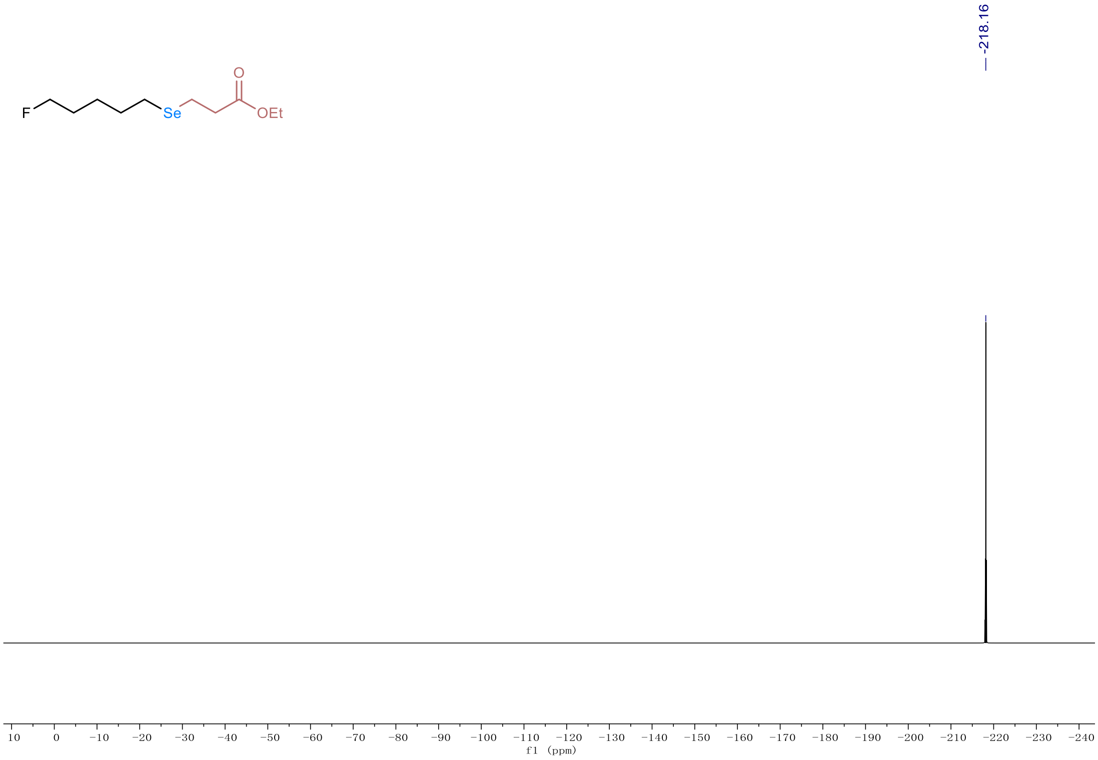

^{1}H NMR of 3l (400 MHz, Chloroform-*d*)

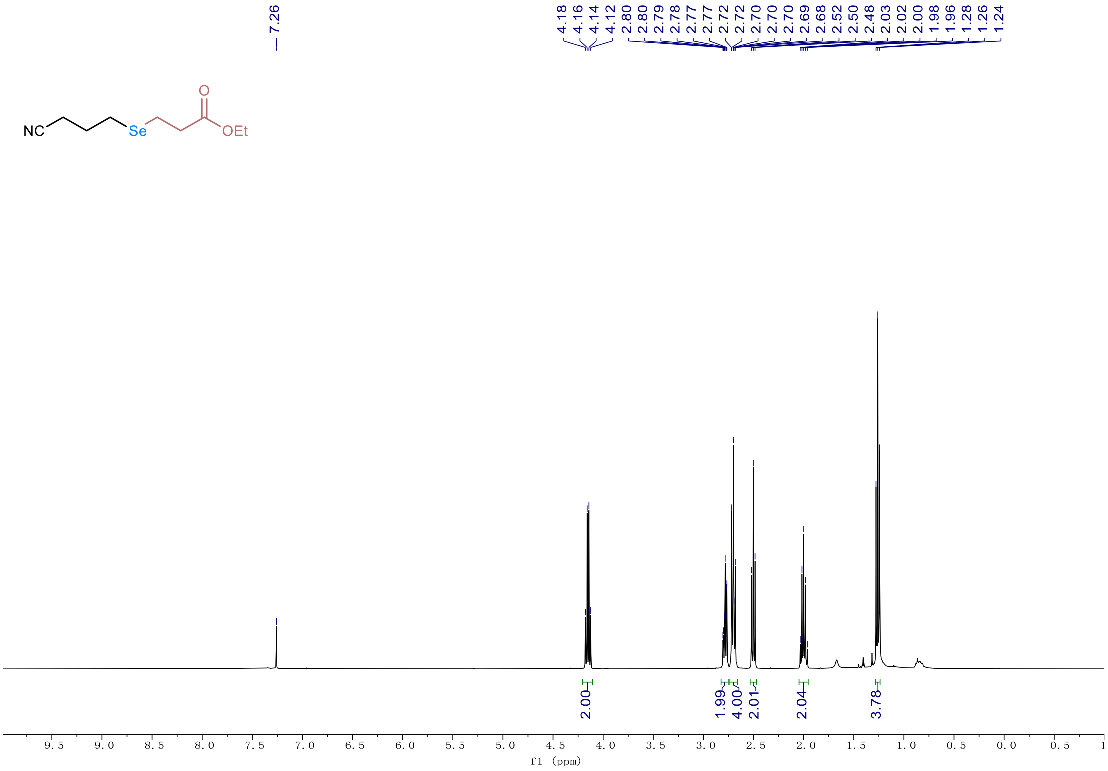

^{13}C NMR of 3l (101 MHz, Chloroform-*d*)

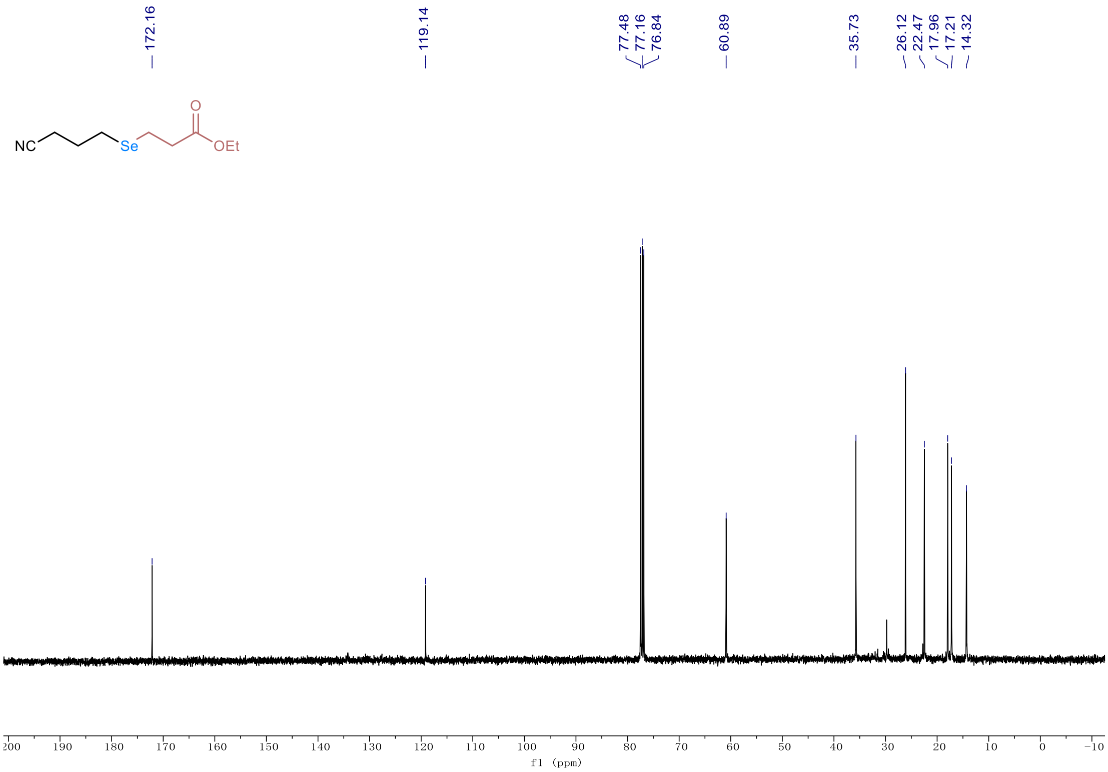

^{1}H NMR of 3m (400 MHz, Chloroform-*d*)

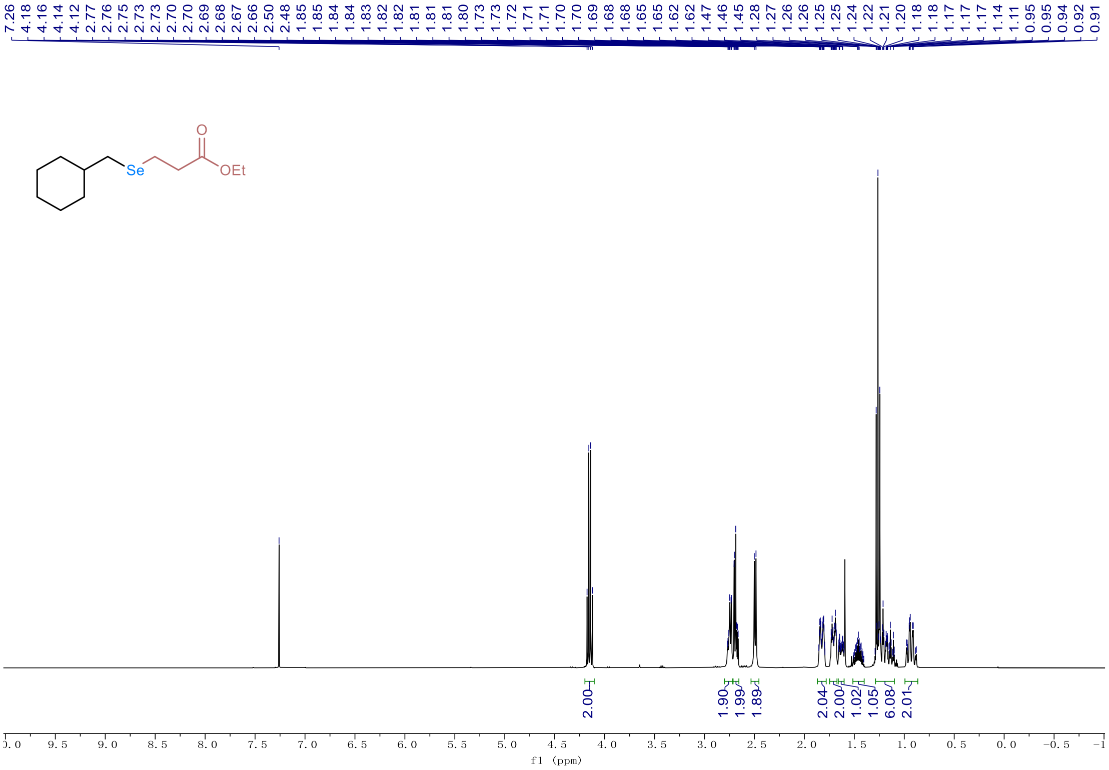

^{13}C NMR of 3m (101 MHz, Chloroform-*d*)

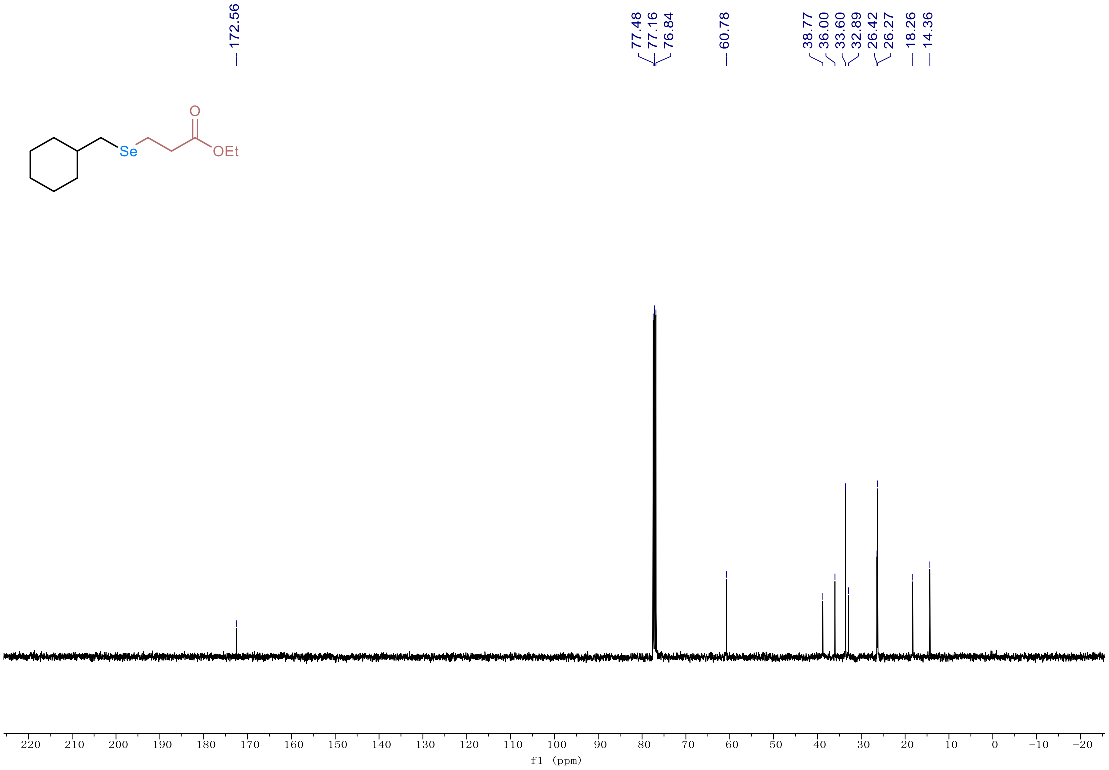

^{1}H NMR of 3n (400 MHz, Chloroform-*d*)

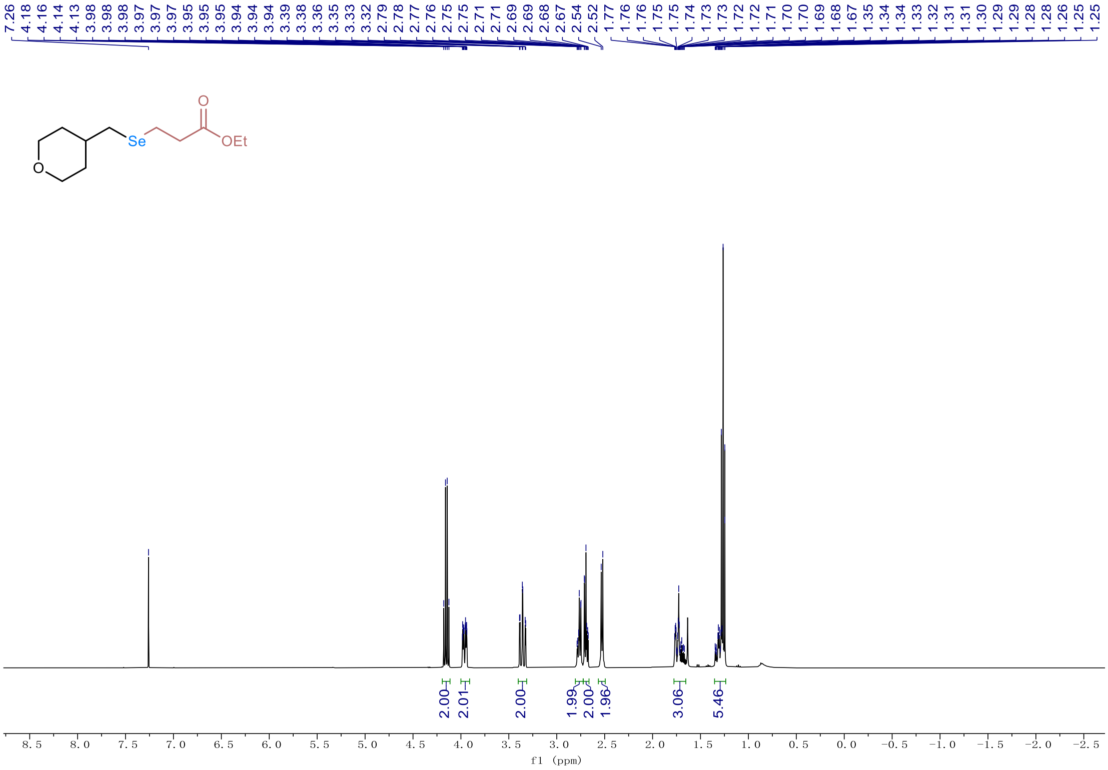

^{13}C NMR of 3n (101 MHz, Chloroform-*d*)

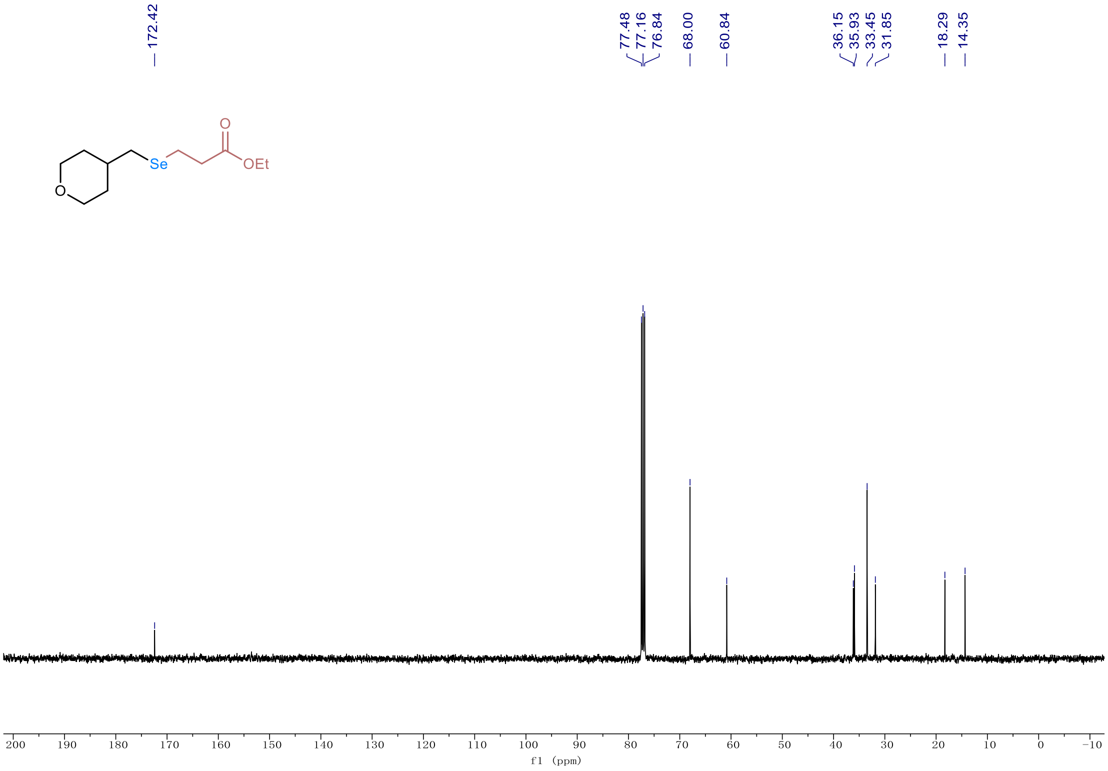

^{1}H NMR of 3o (400 MHz, Chloroform-*d*)

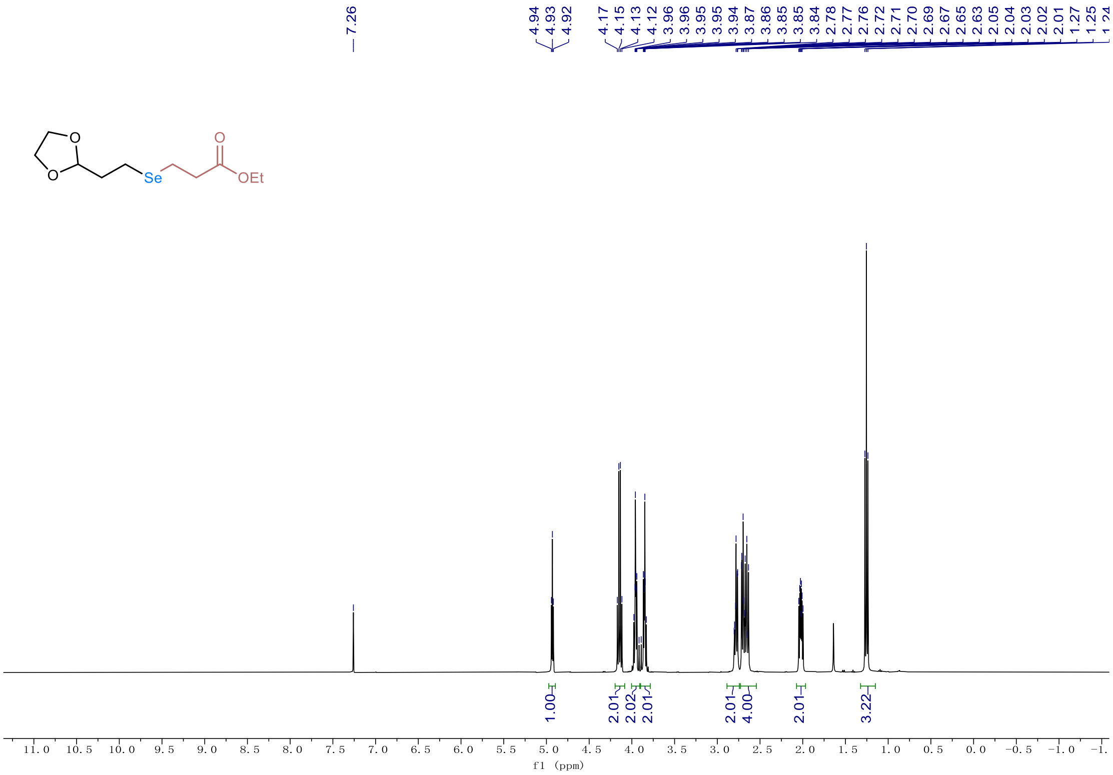

^{13}C NMR of 3o (101 MHz, Chloroform-*d*)

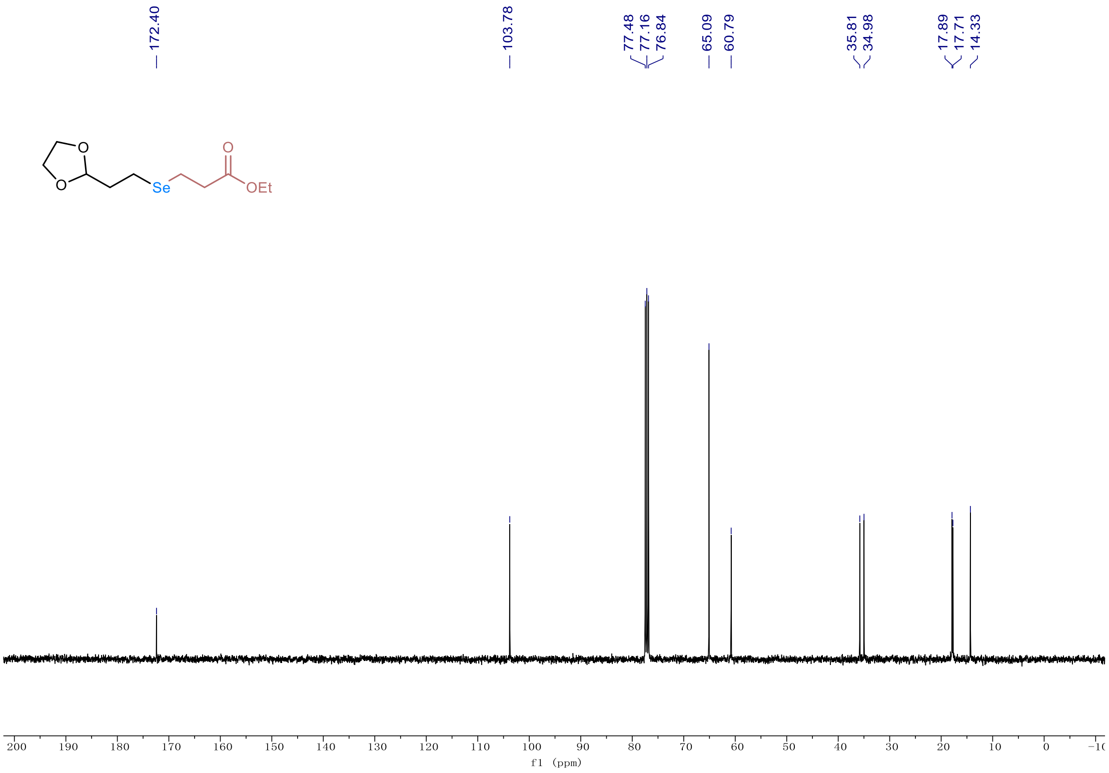

^{1}H NMR of 3p (400 MHz, Chloroform-*d*)

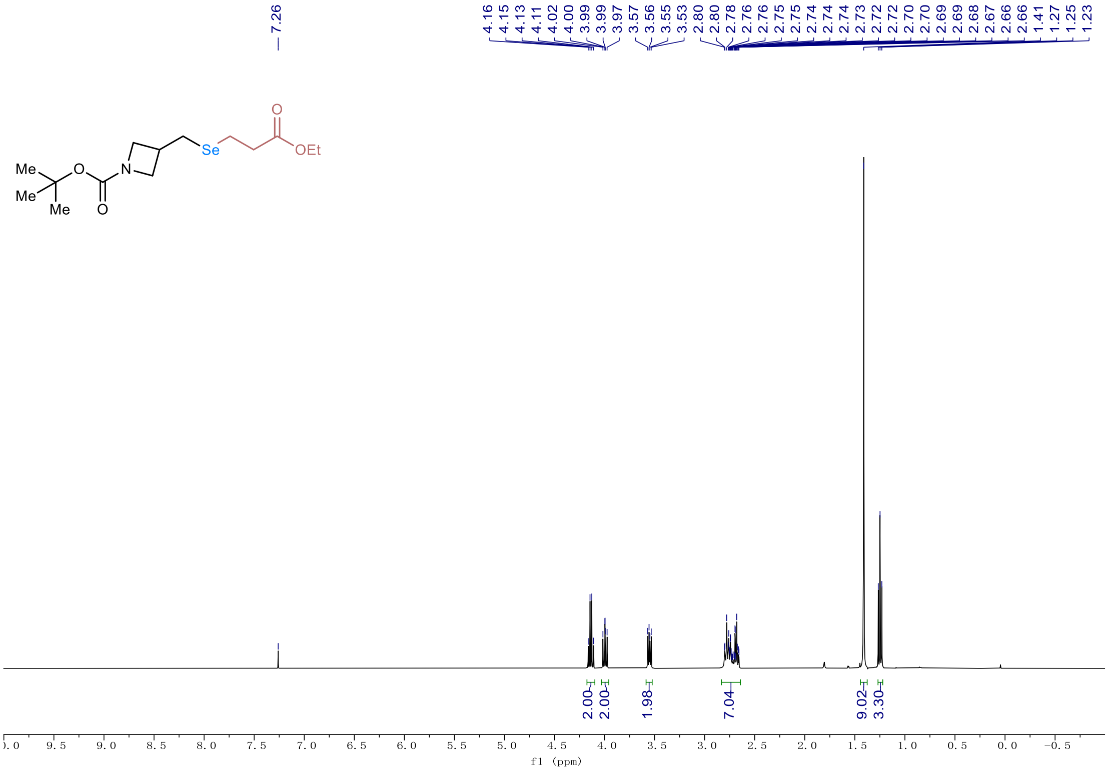

^{13}C NMR of 3p (101 MHz, Chloroform-*d*)

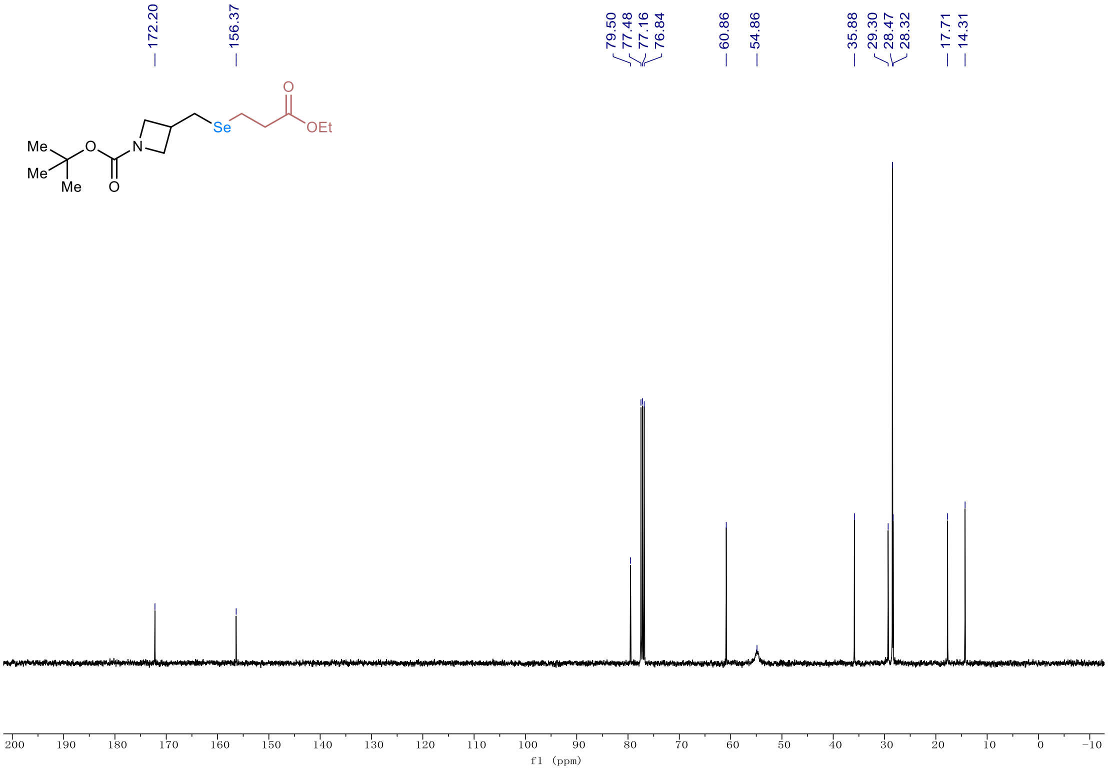

^{1}H NMR of 3q (400 MHz, Chloroform-*d*)

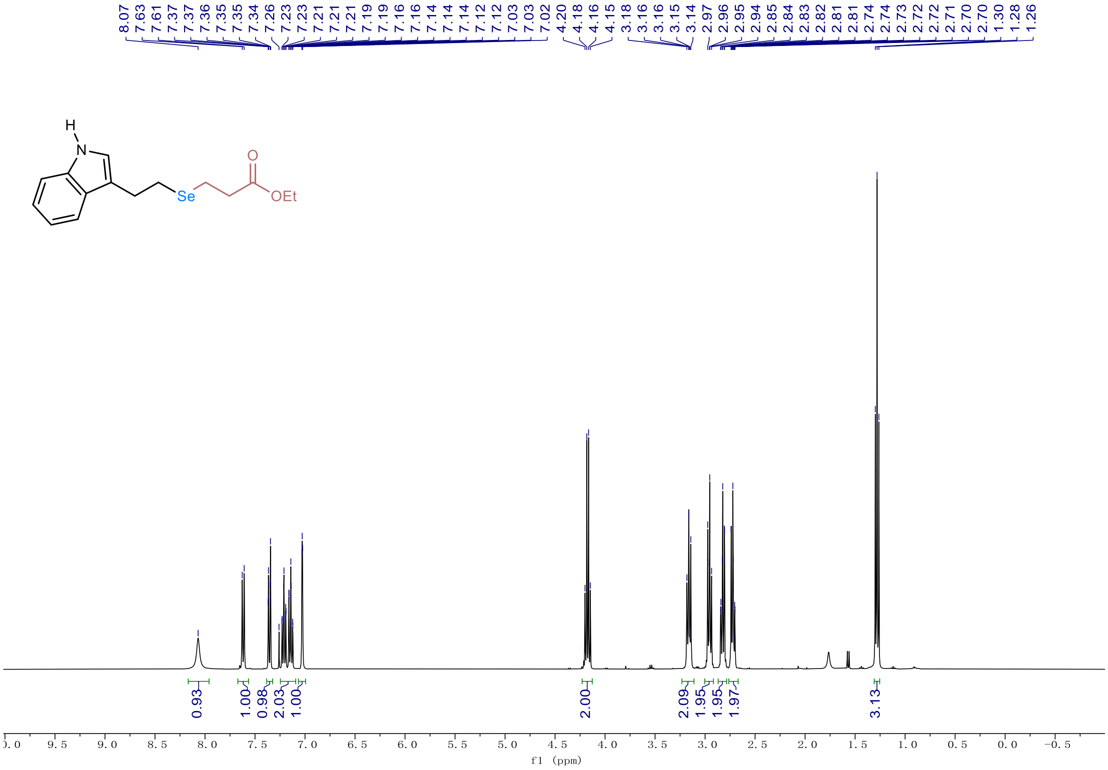

^{13}C NMR of 3q (101 MHz, Chloroform-*d*)

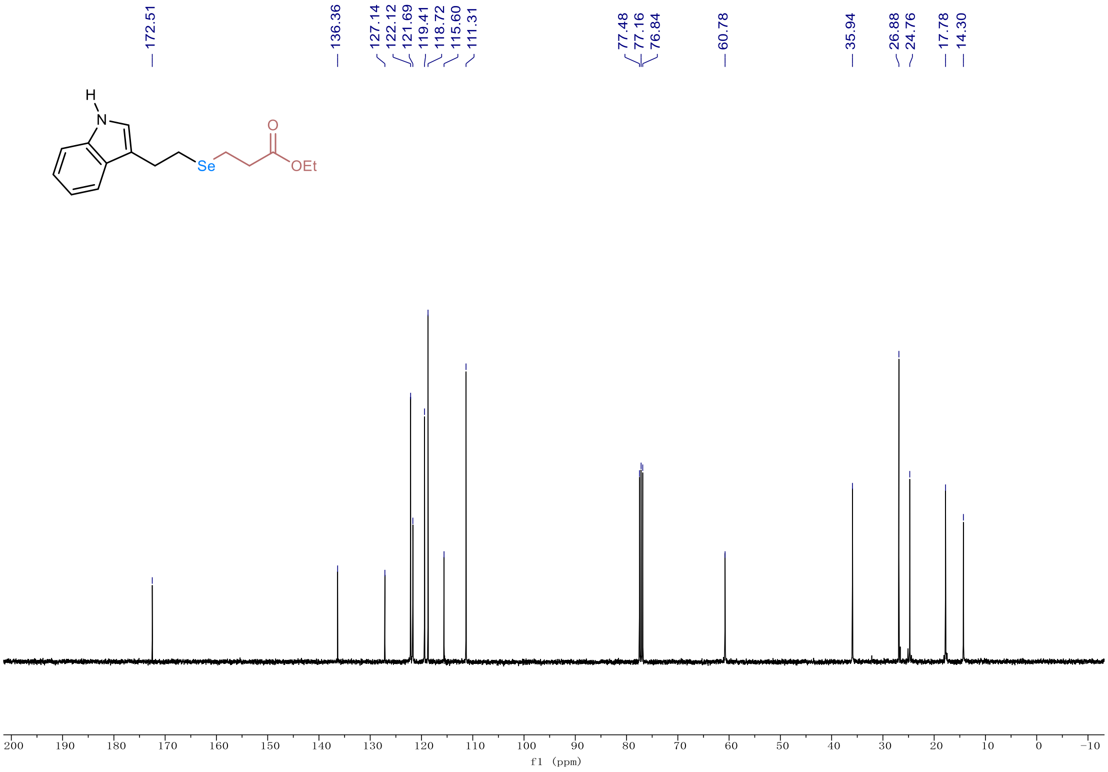

^{1}H NMR of 3r (400 MHz, Chloroform-*d*)

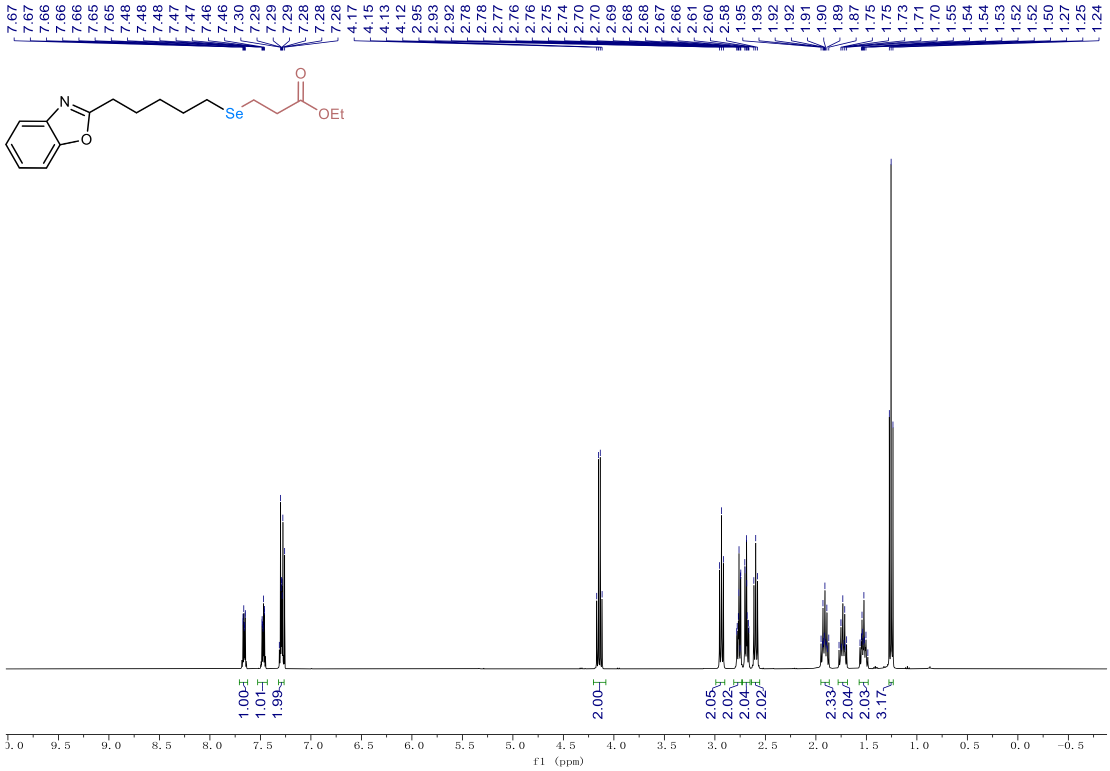

^{13}C NMR of 3r (101 MHz, Chloroform-*d*)

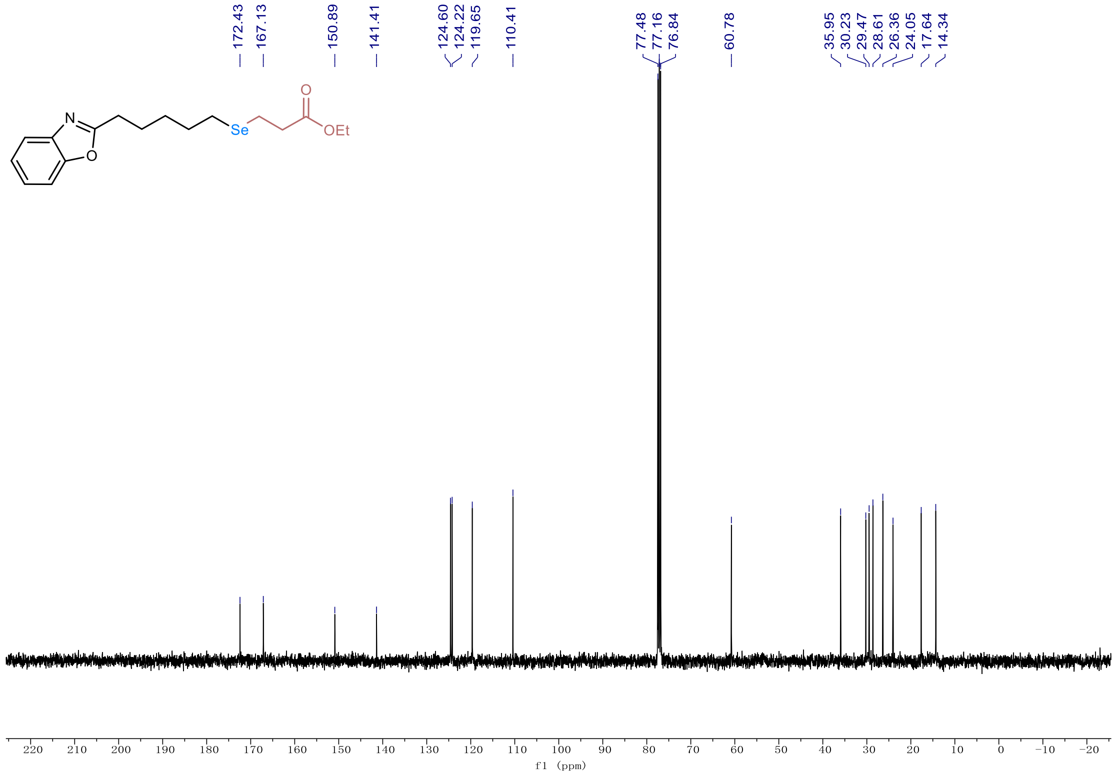

^{1}H NMR of 3s (400 MHz, Chloroform-*d*)

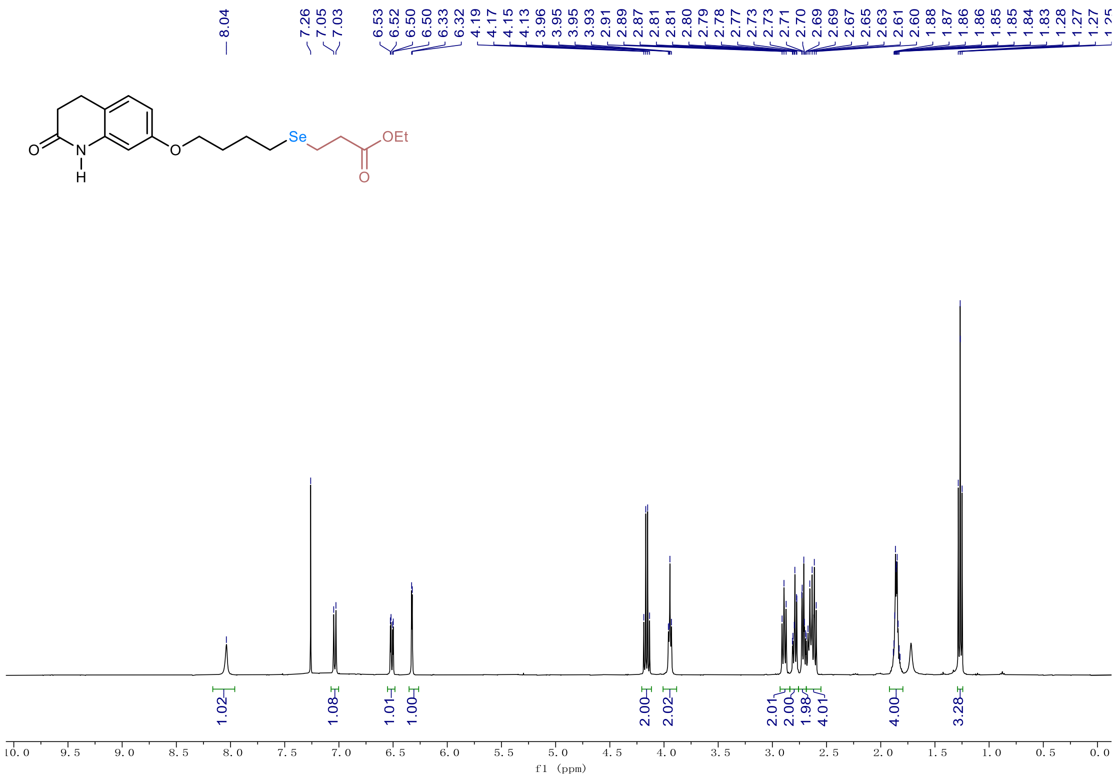

^{13}C NMR of 3s (101 MHz, Chloroform-*d*)

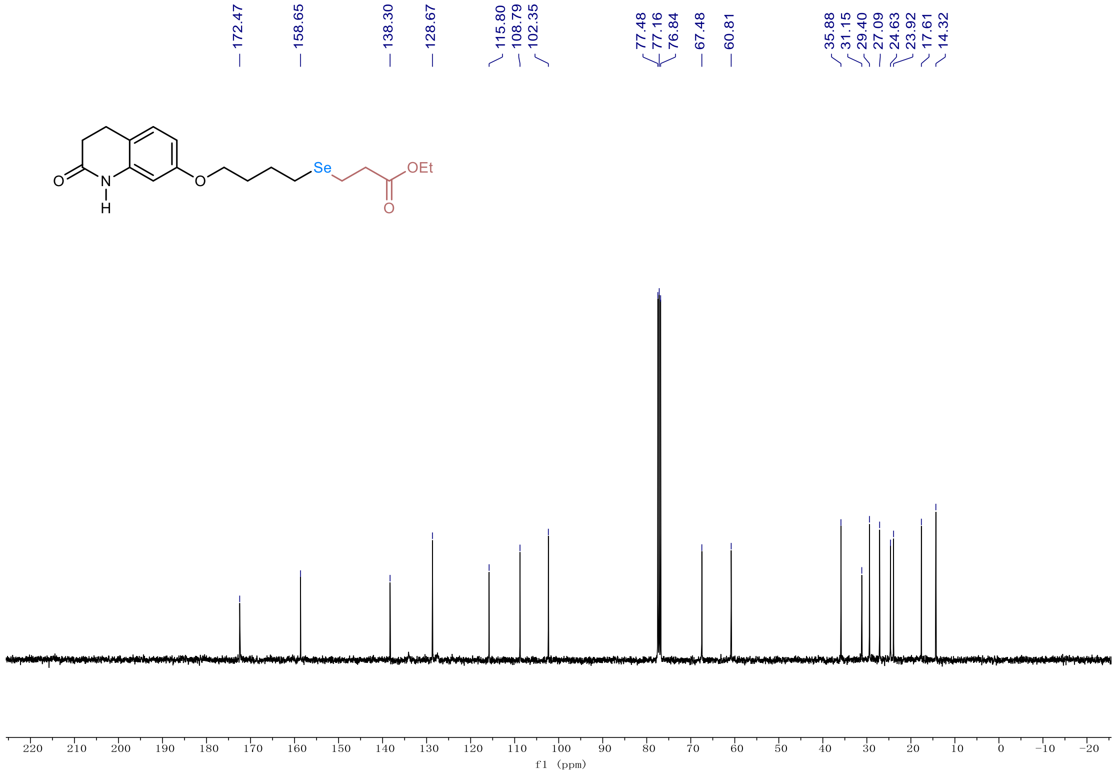

^{1}H NMR of 3t (400 MHz, Chloroform-*d*)

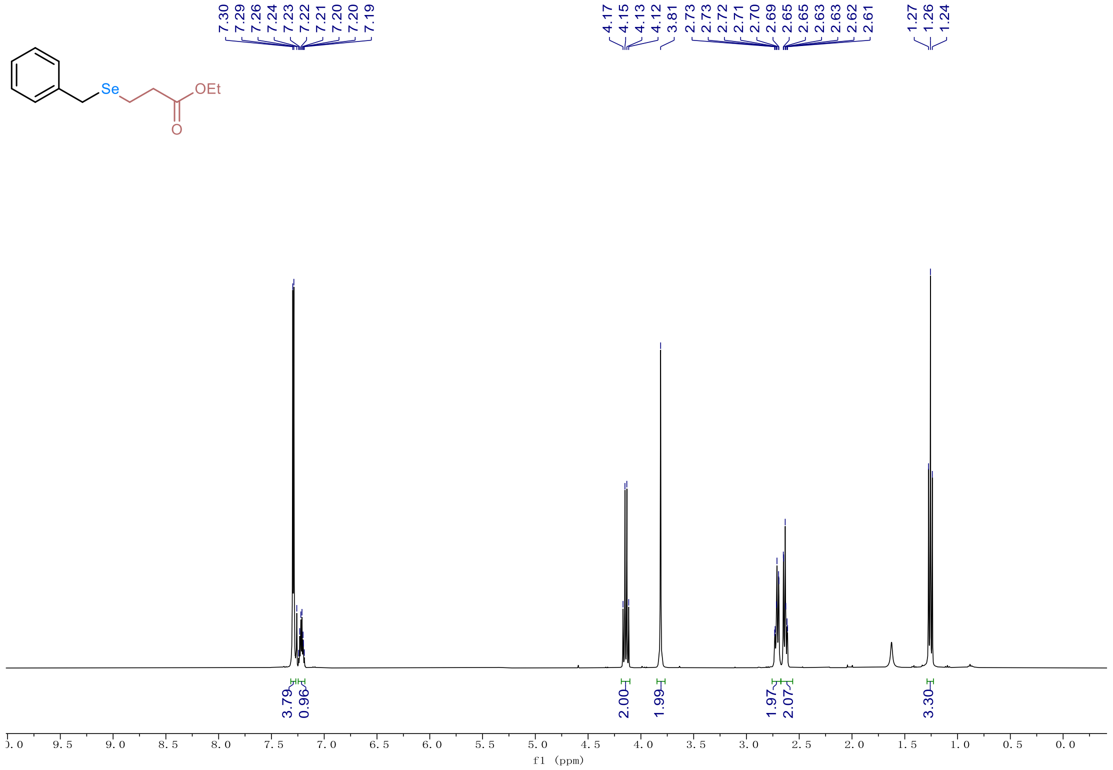

^{13}C NMR of 3t (101 MHz, Chloroform-*d*)

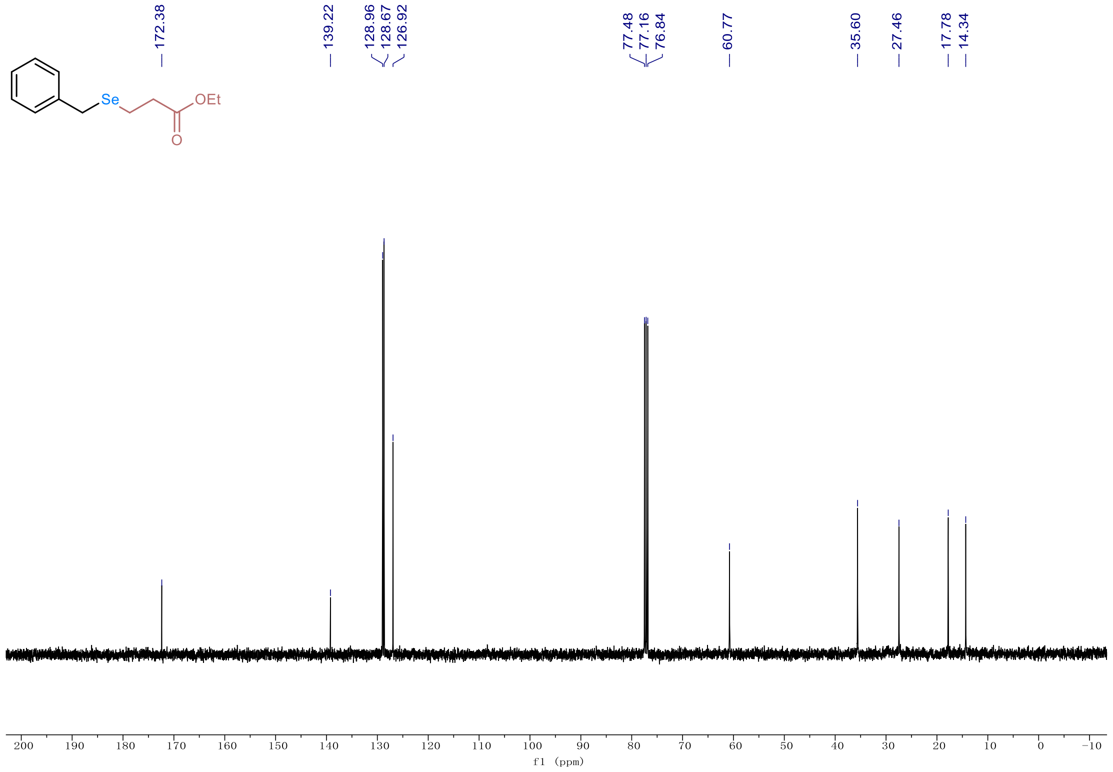

^{1}H NMR of 3u (400 MHz, Chloroform-*d*)

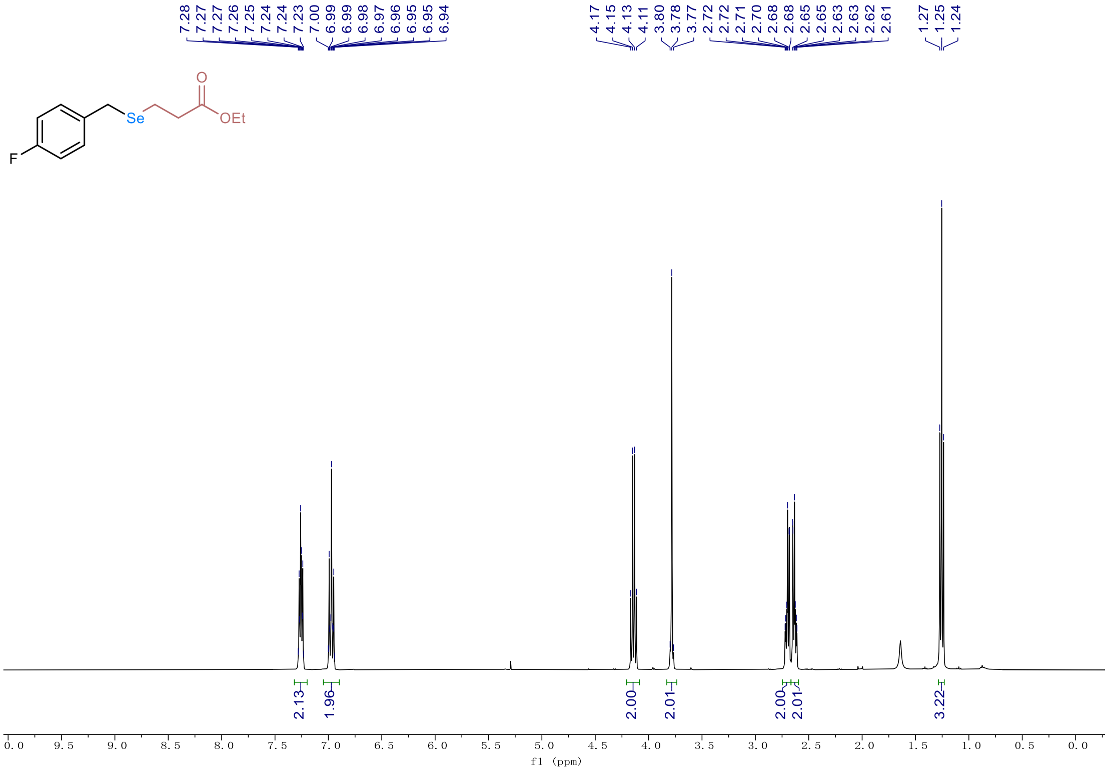

^{13}C NMR of 3u (101 MHz, Chloroform-*d*)

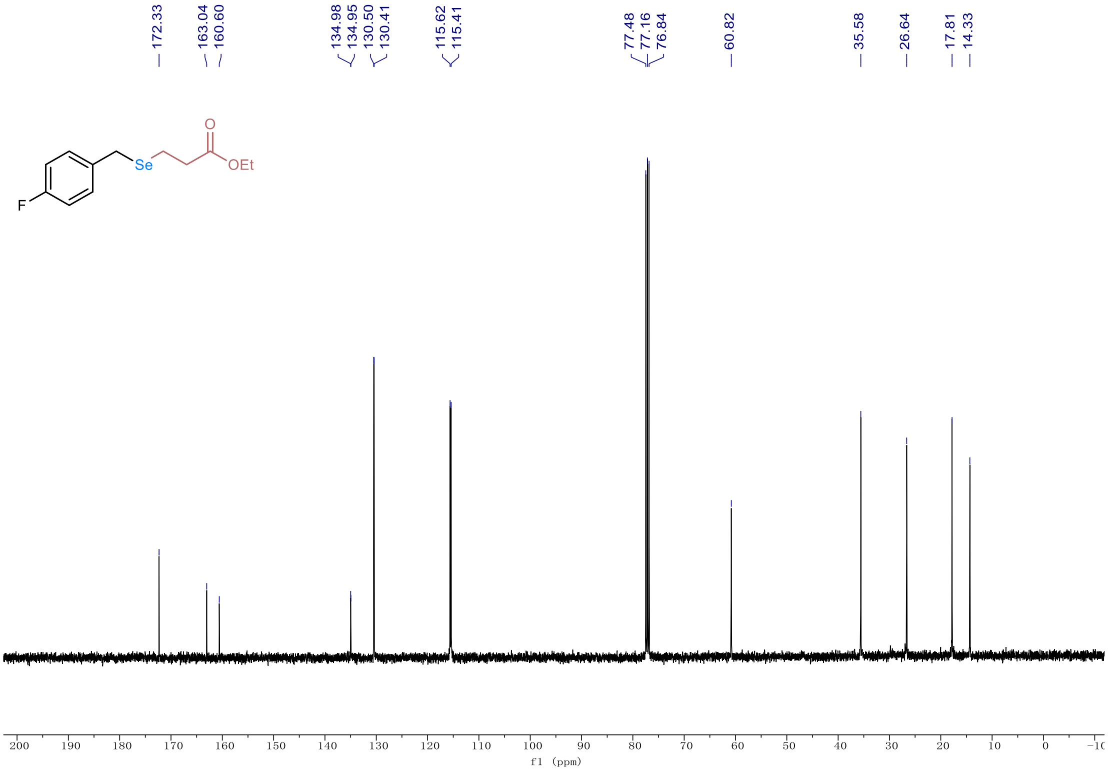

^{19}F NMR of 3u (376 MHz, Chloroform-*d*)

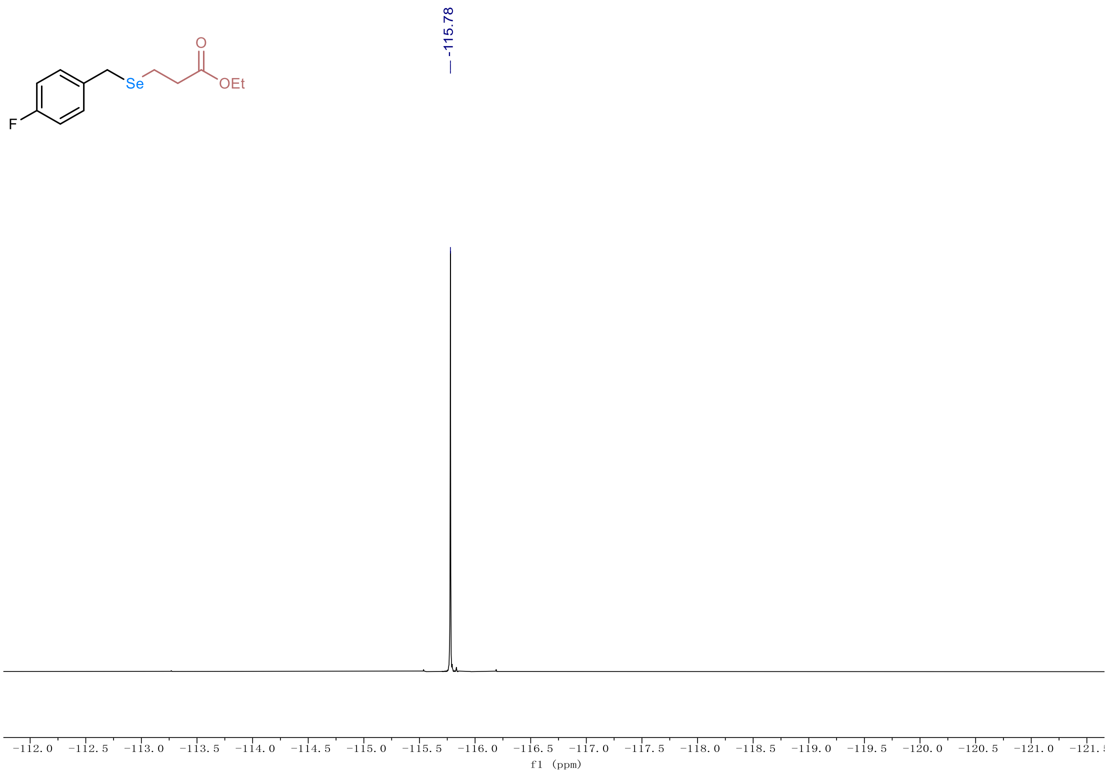

^{1}H NMR of 3v (400 MHz, Chloroform-*d*)

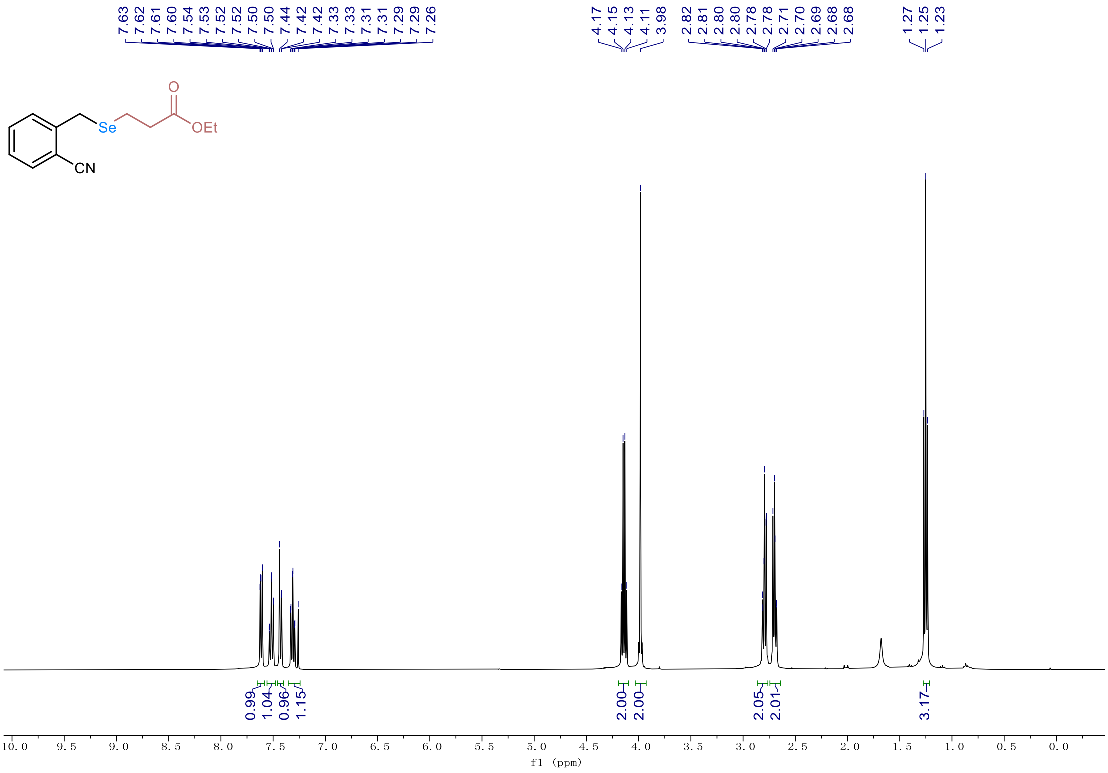

^{13}C NMR of 3v (101 MHz, Chloroform-*d*)

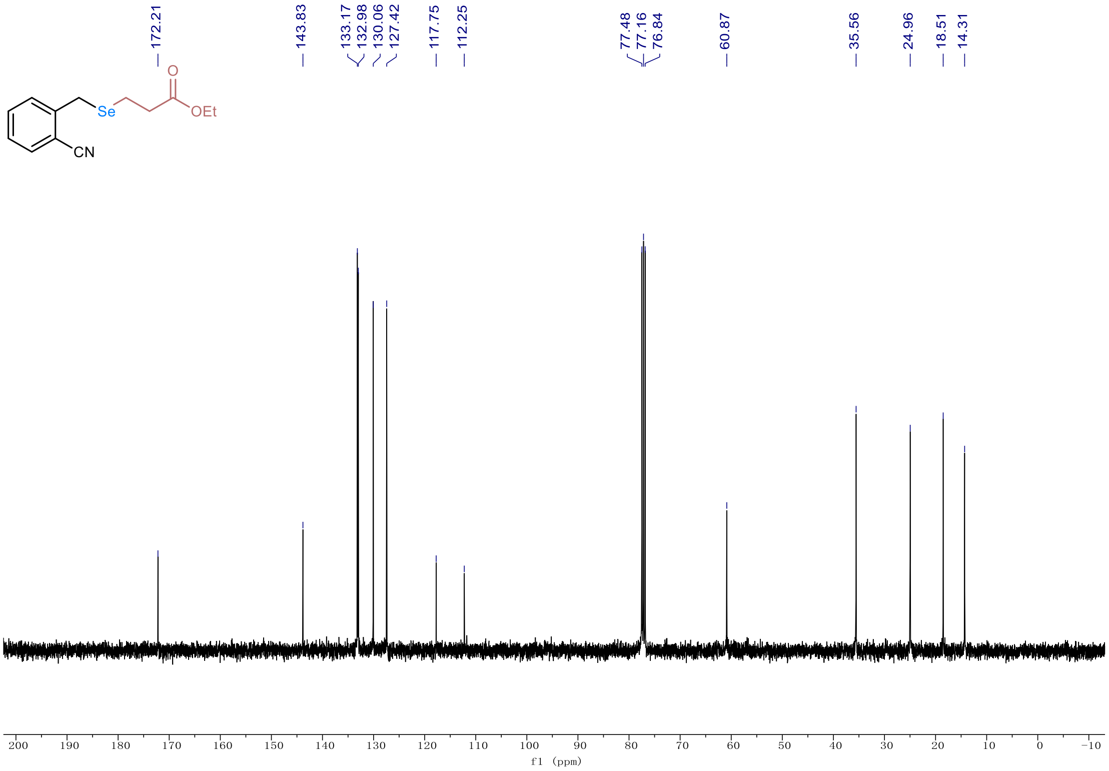

^{1}H NMR of 3w (400 MHz, Chloroform-*d*)

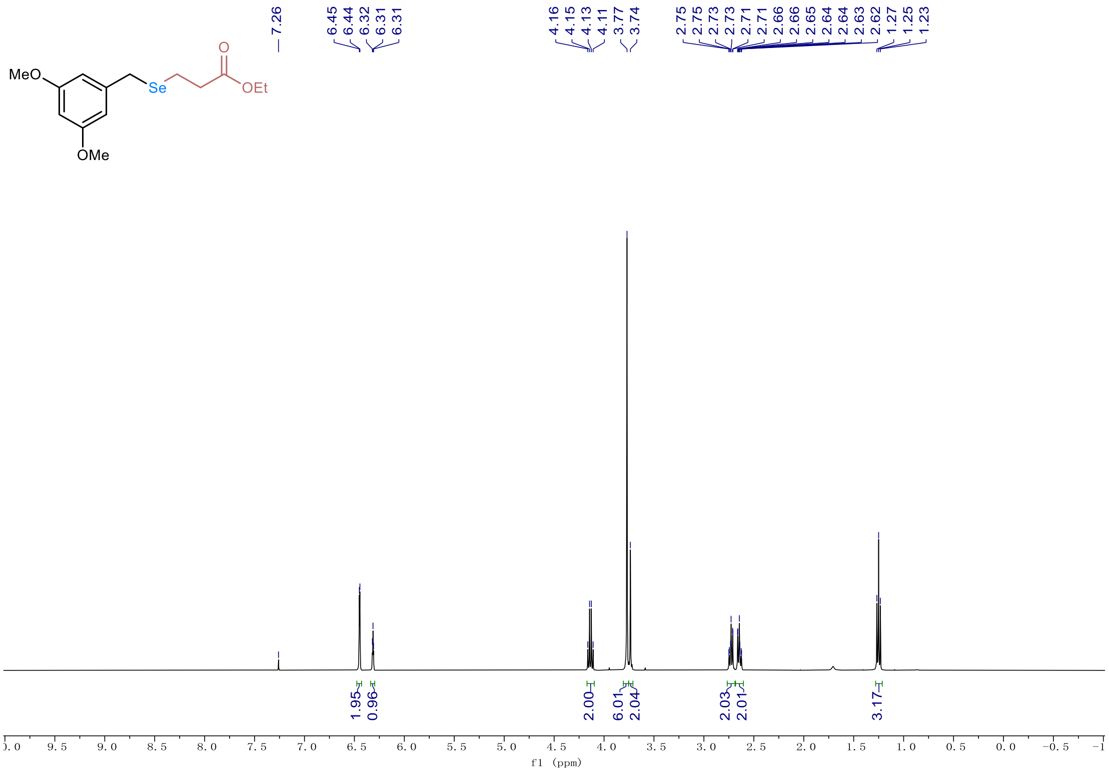

^{13}C NMR of 3w (101 MHz, Chloroform-*d*)

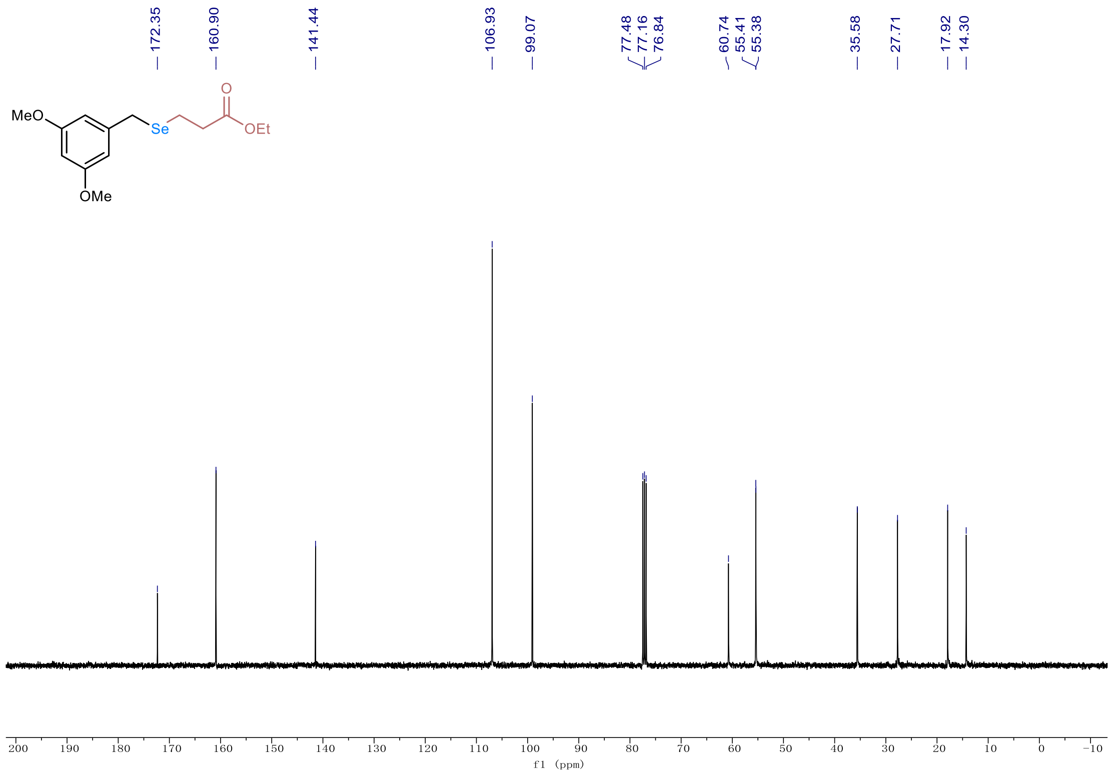

^{1}H NMR of 3x (400 MHz, Chloroform-*d*)

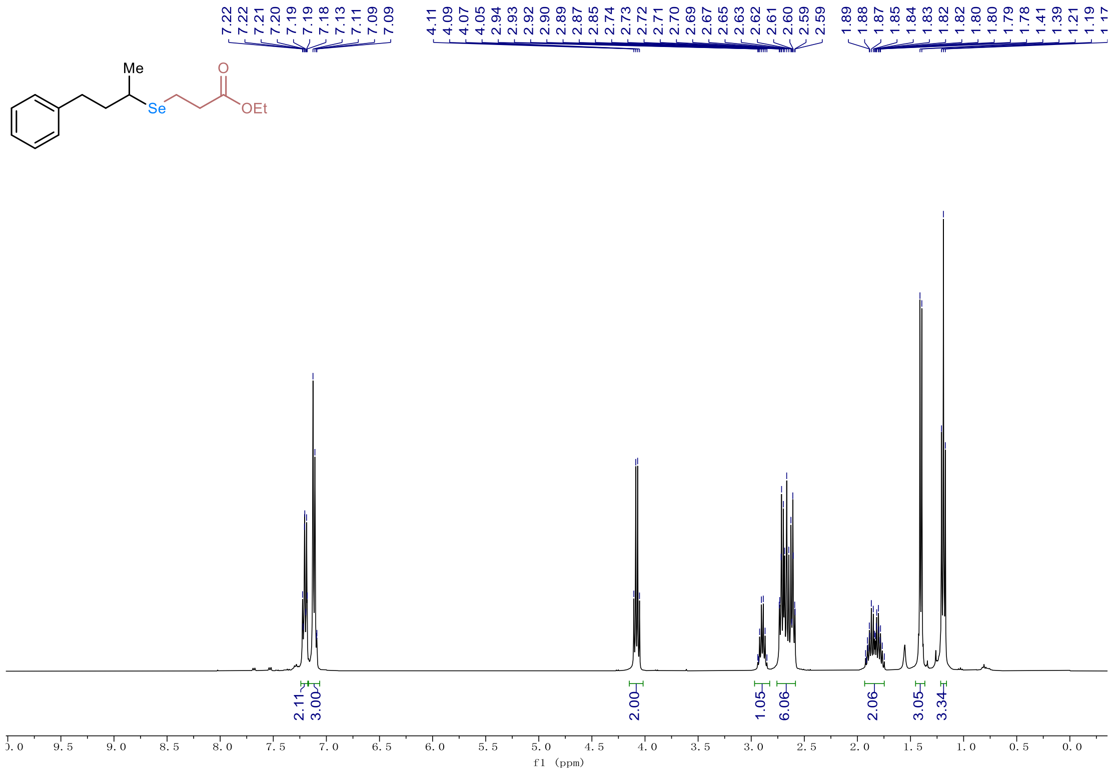

^{13}C NMR of 3x (101 MHz, Chloroform-*d*)

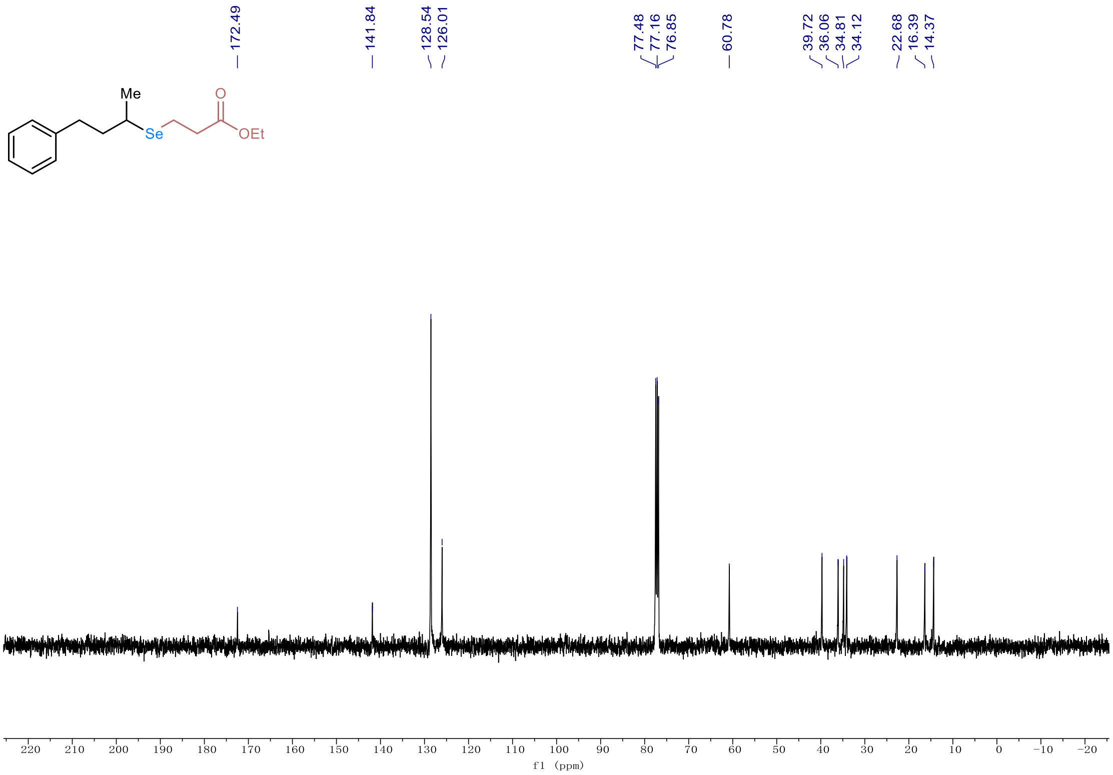

^{1}H NMR of 3y (400 MHz, Chloroform-*d*)

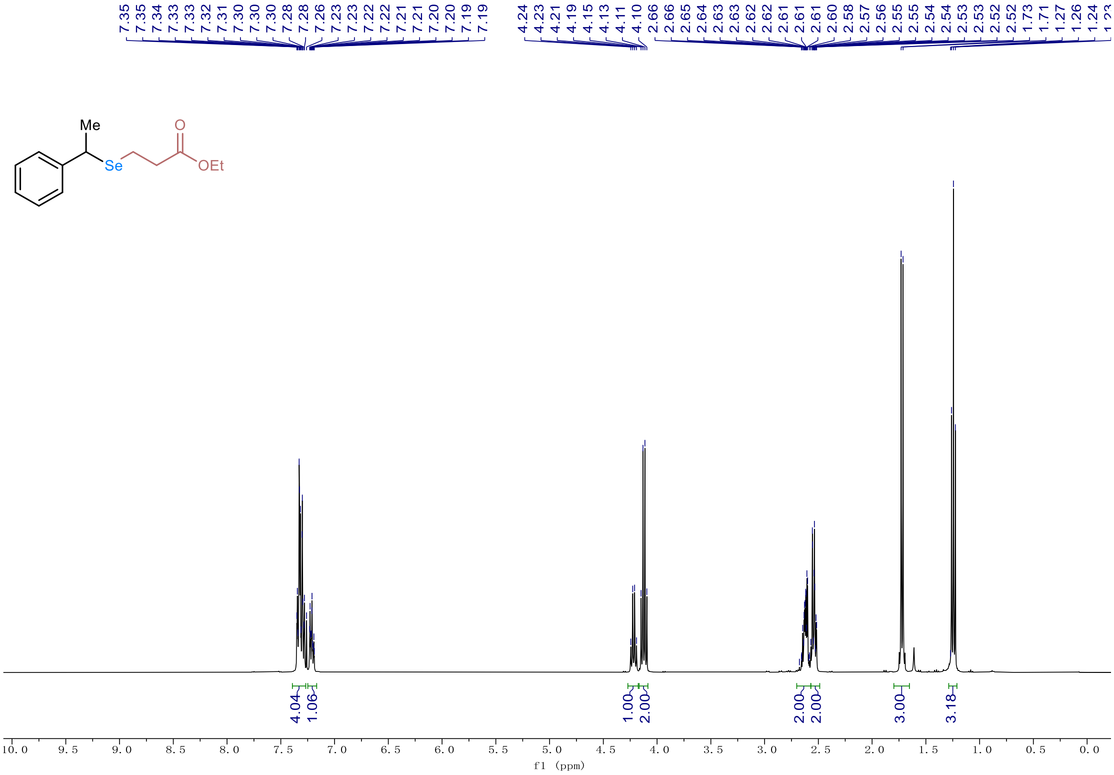

^{13}C NMR of 3y (101 MHz, Chloroform-*d*)

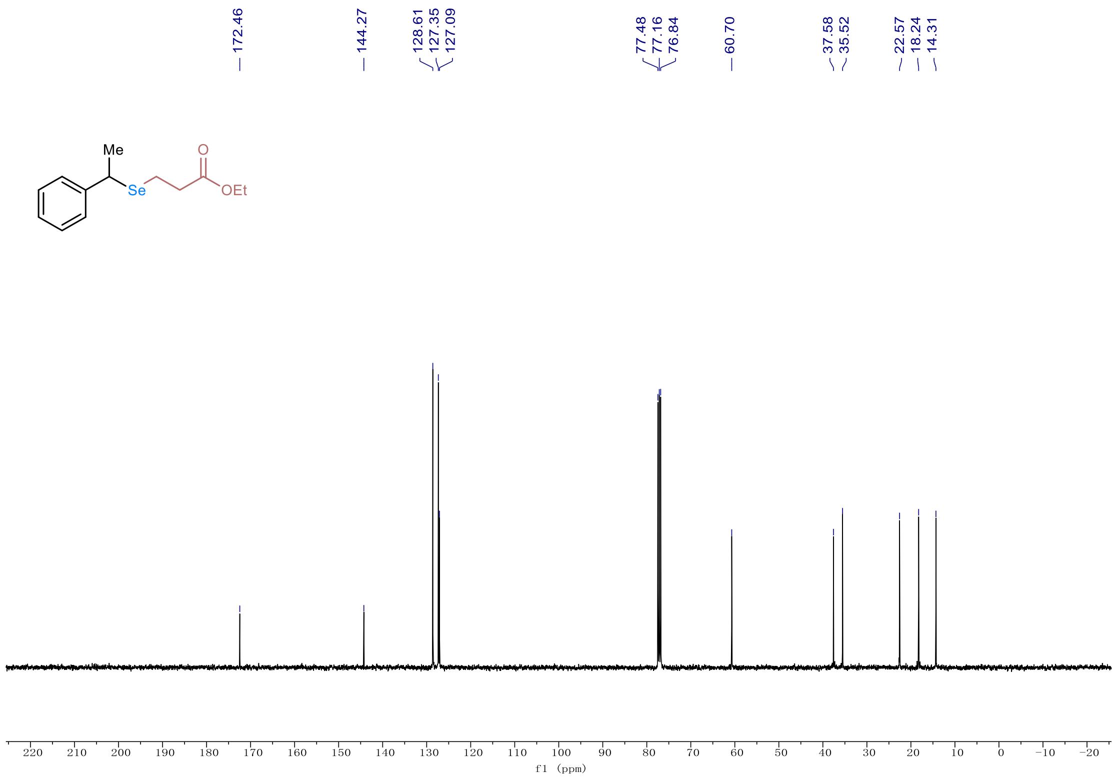

^{1}H NMR of 3z (400 MHz, Chloroform-*d*)

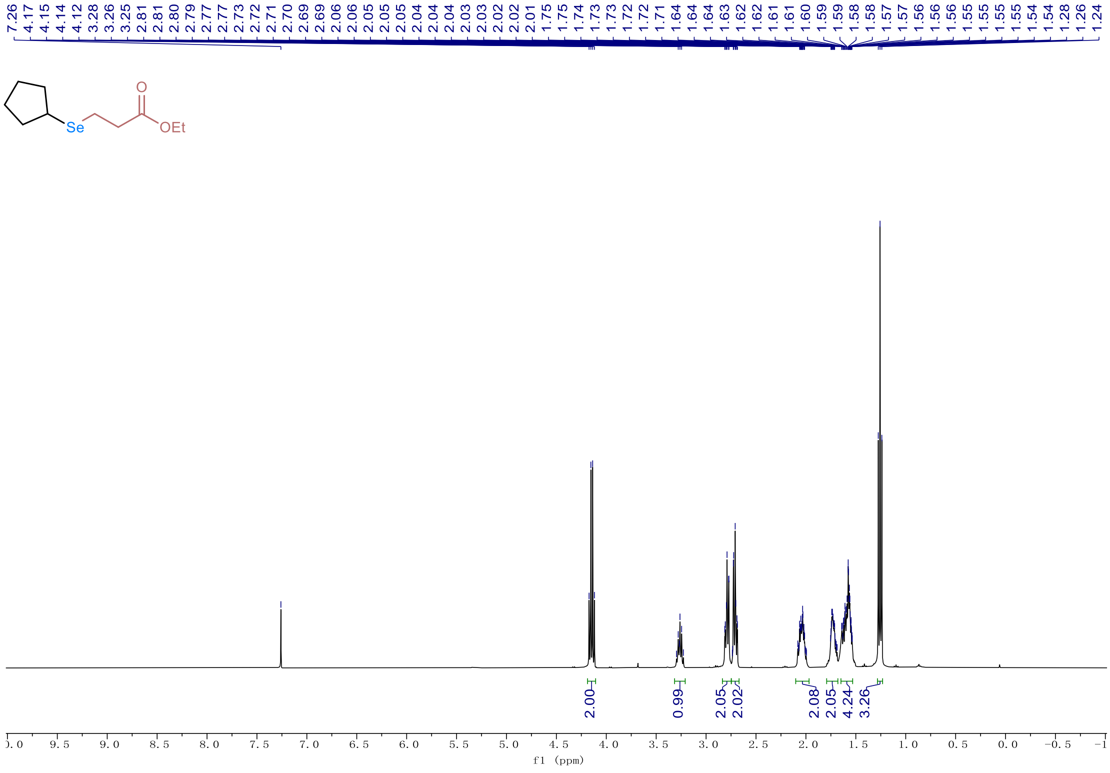

^{13}C NMR of 3z (101 MHz, Chloroform-*d*)

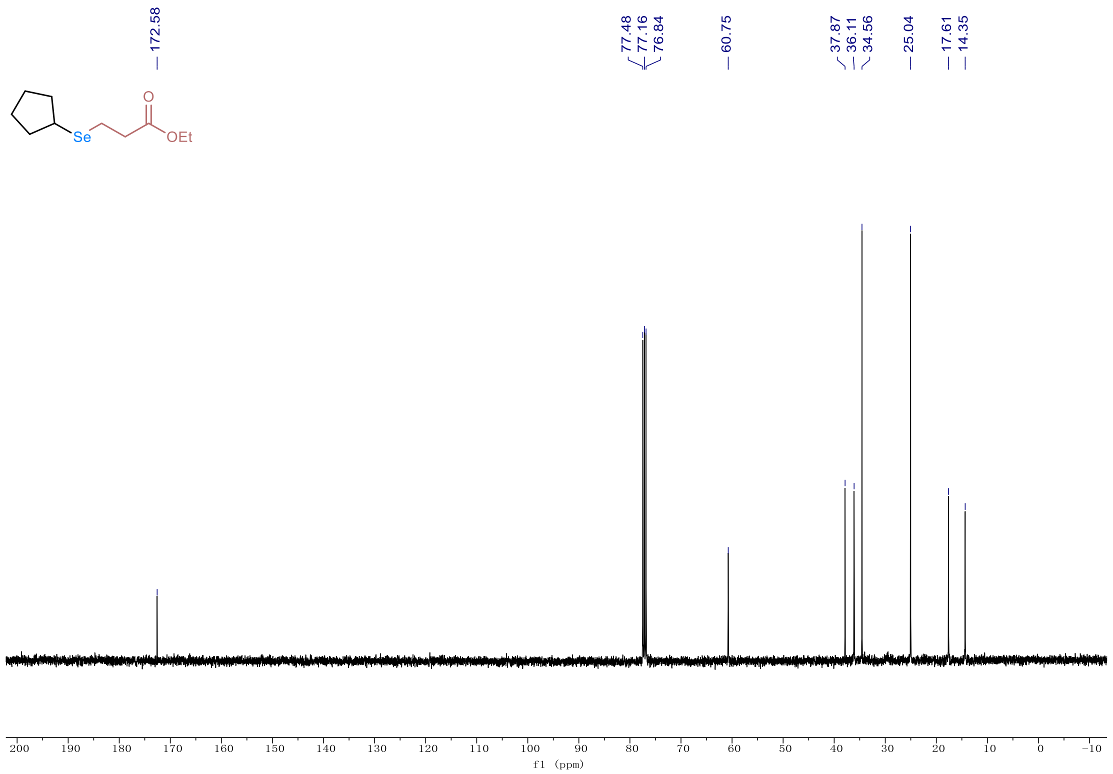

^{1}H NMR of 3aa (400 MHz, Chloroform-*d*)

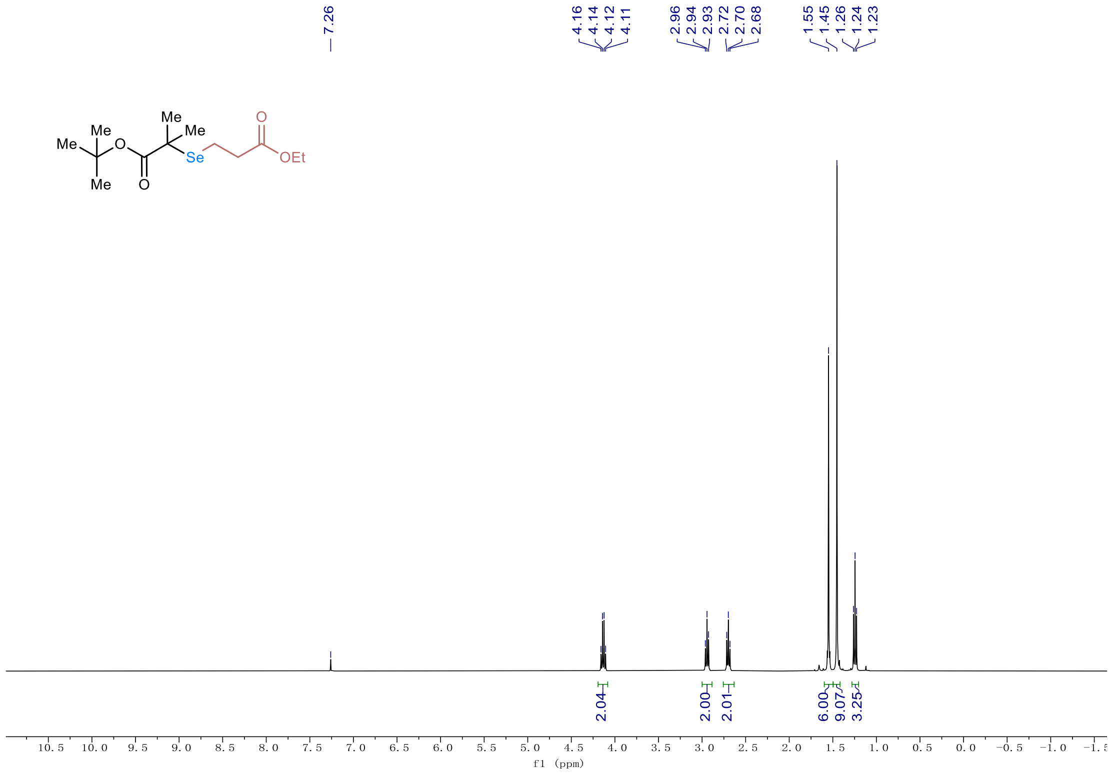

^{13}C NMR of 3aa (101 MHz, Chloroform-*d*)

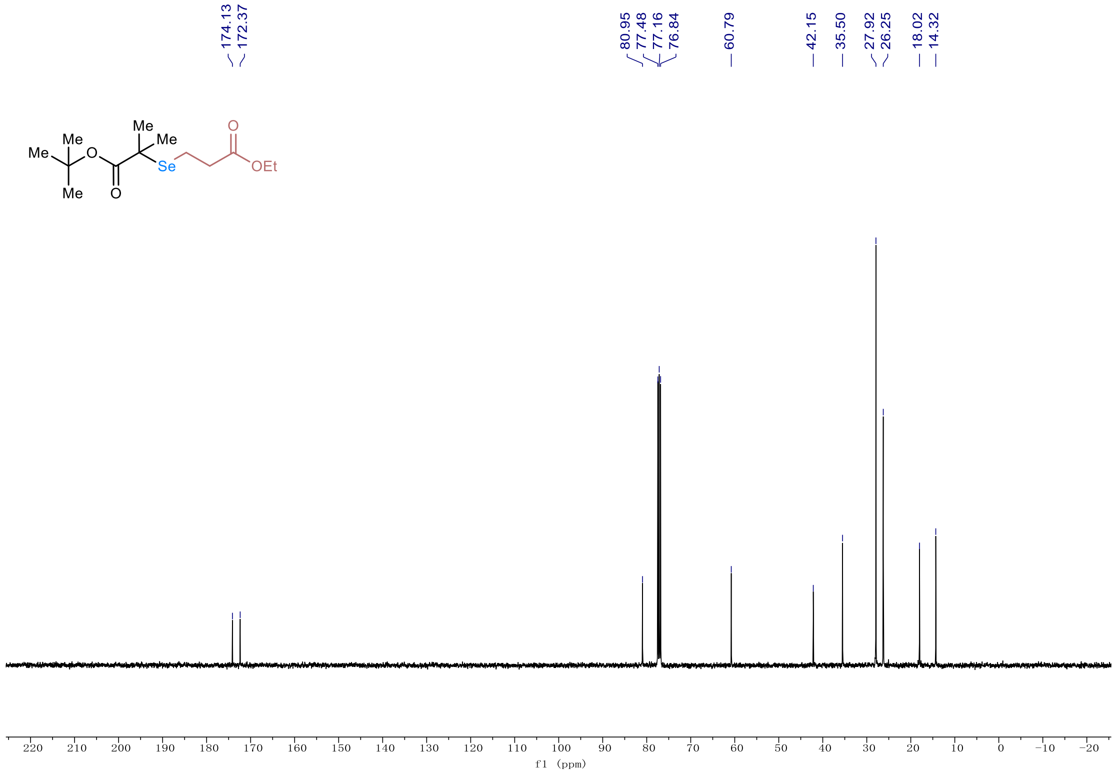

^{1}H NMR of 3ab (400 MHz, Chloroform-*d*)

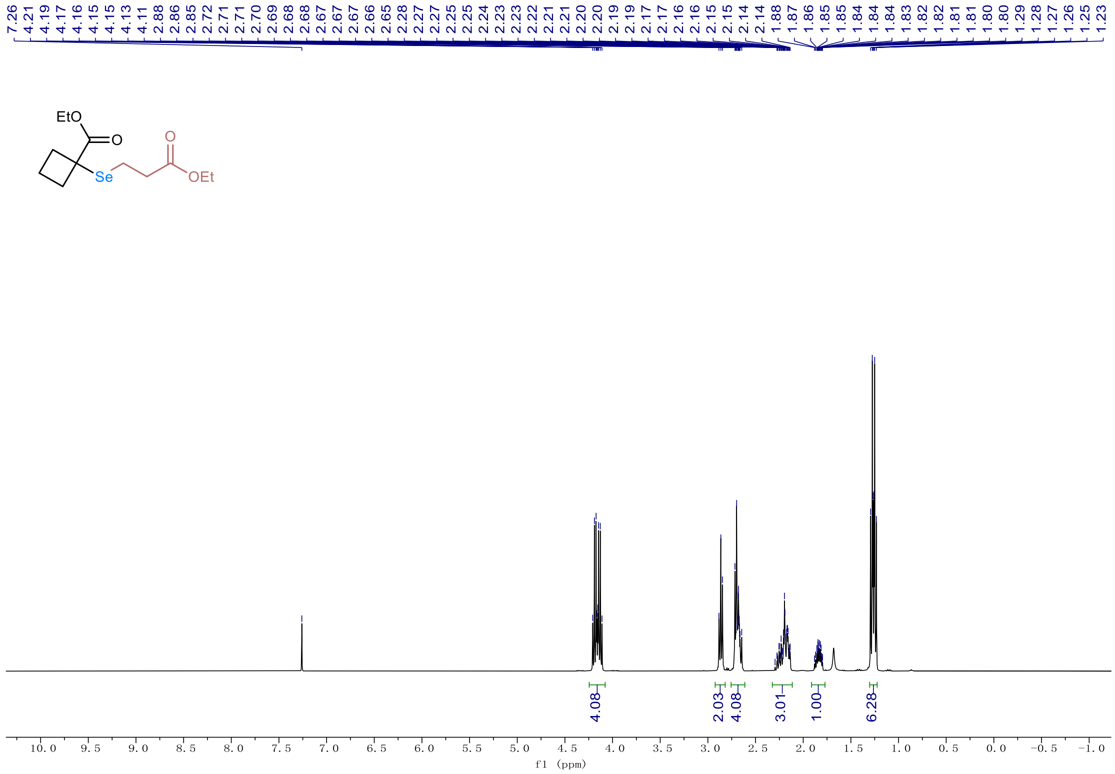

^{13}C NMR of 3ab (101 MHz, Chloroform-*d*)

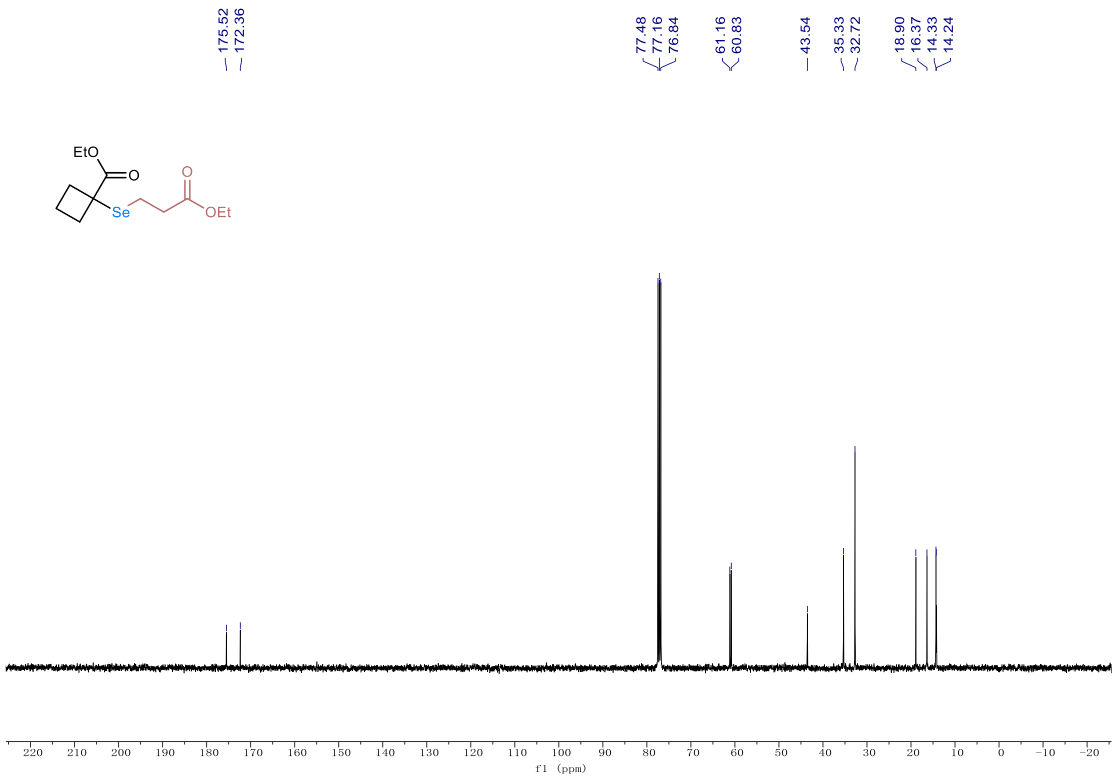

^{1}H NMR of 3ac (400 MHz, Chloroform-*d*)

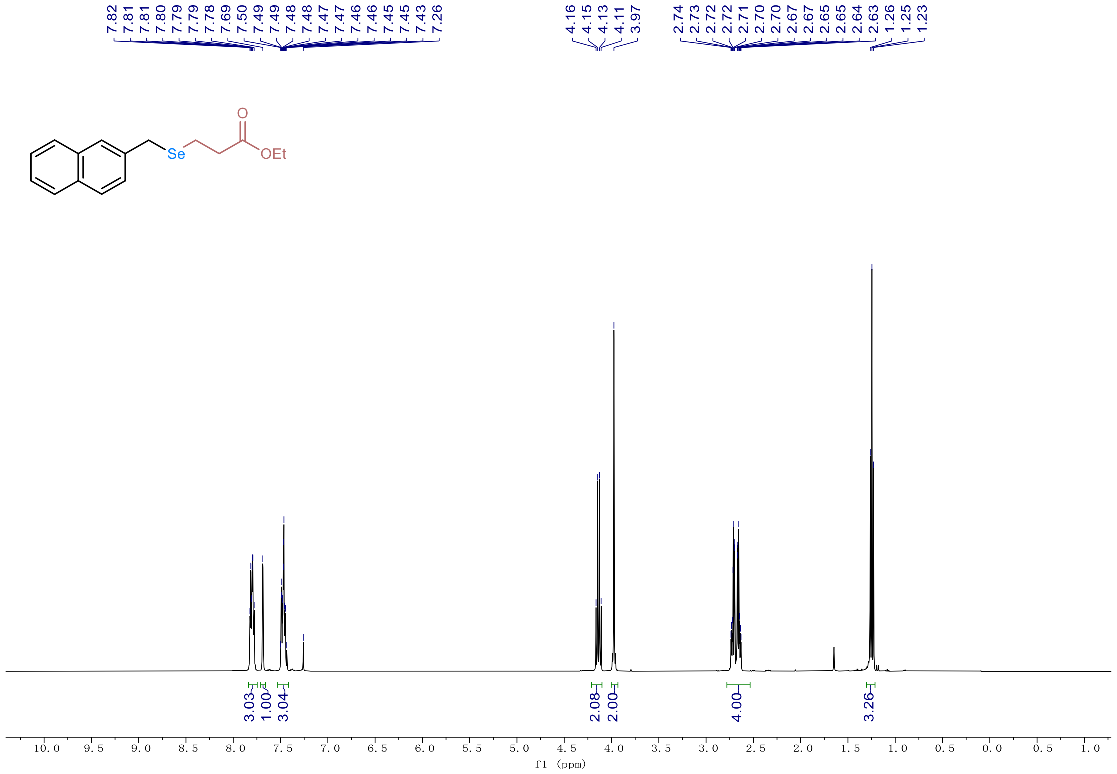

^{13}C NMR of 3ac (101 MHz, Chloroform-*d*)

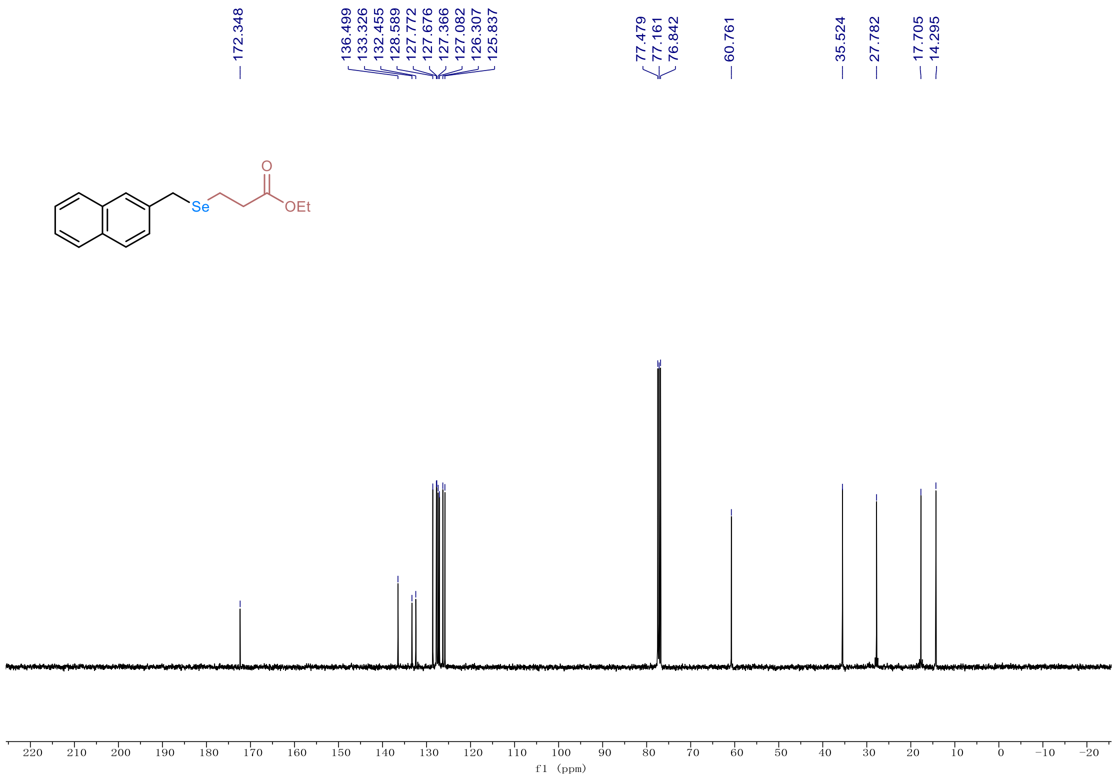

^{1}H NMR of 3ad (400 MHz, Chloroform-*d*)

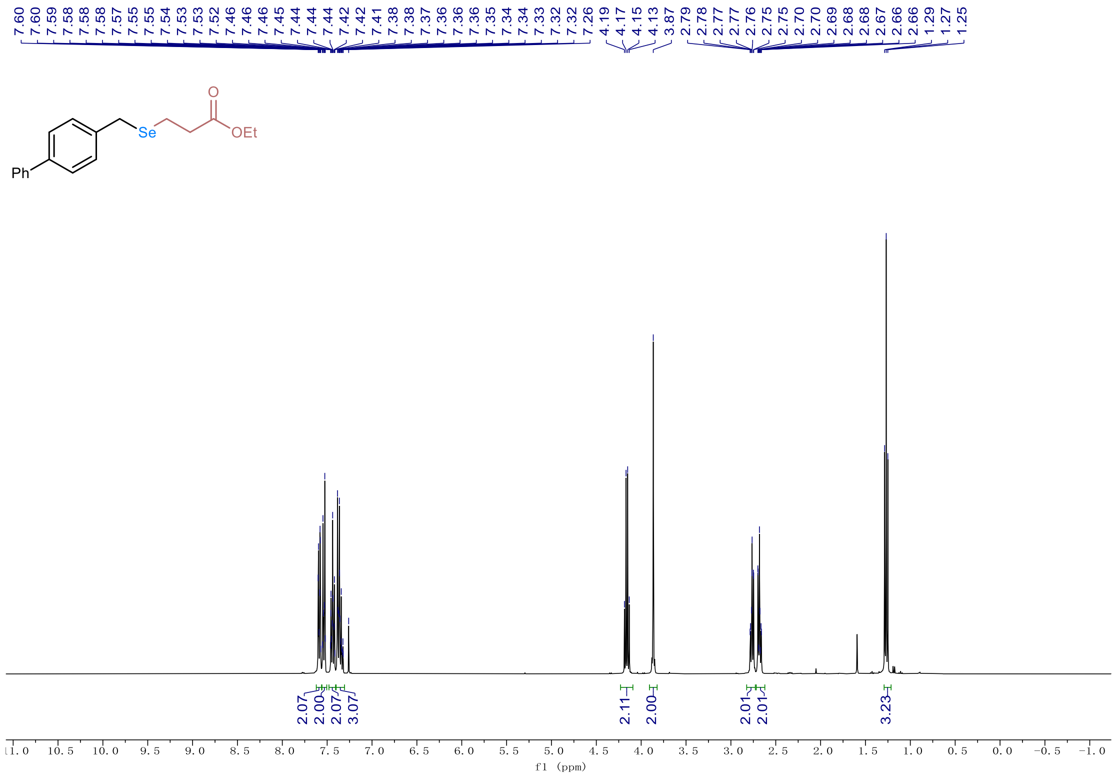

^{13}C NMR of 3ad (101 MHz, Chloroform-*d*)

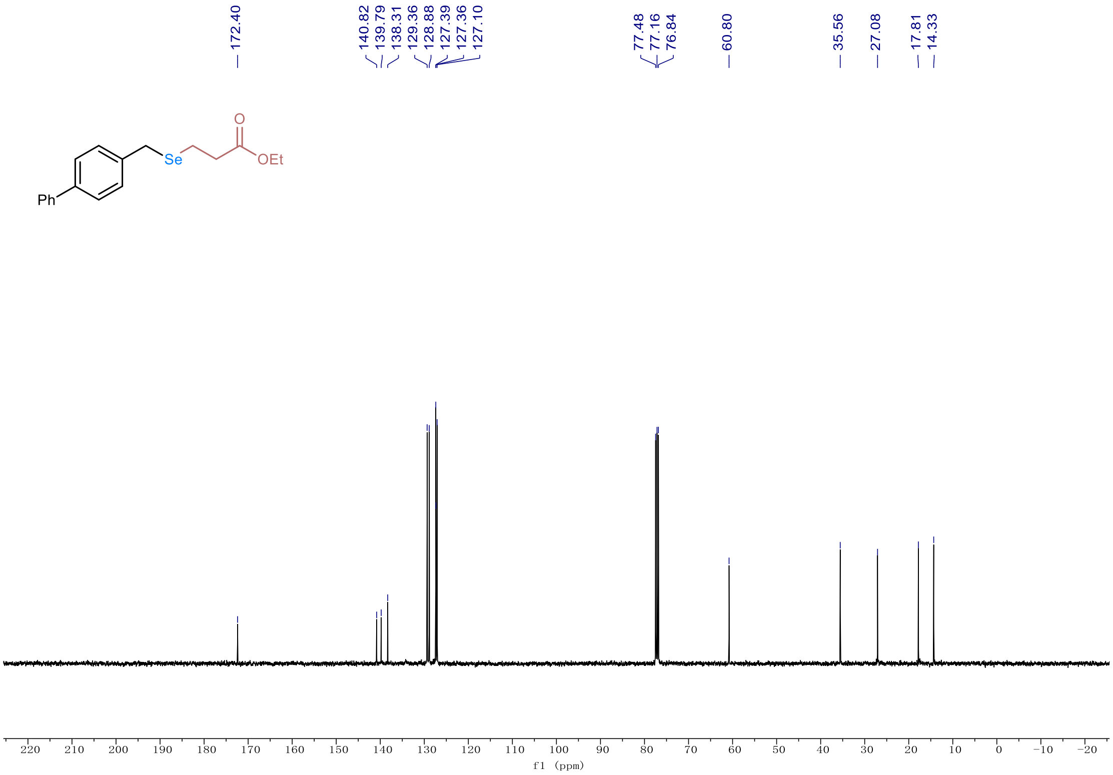

^{1}H NMR of 3ae (400 MHz, Chloroform-*d*)

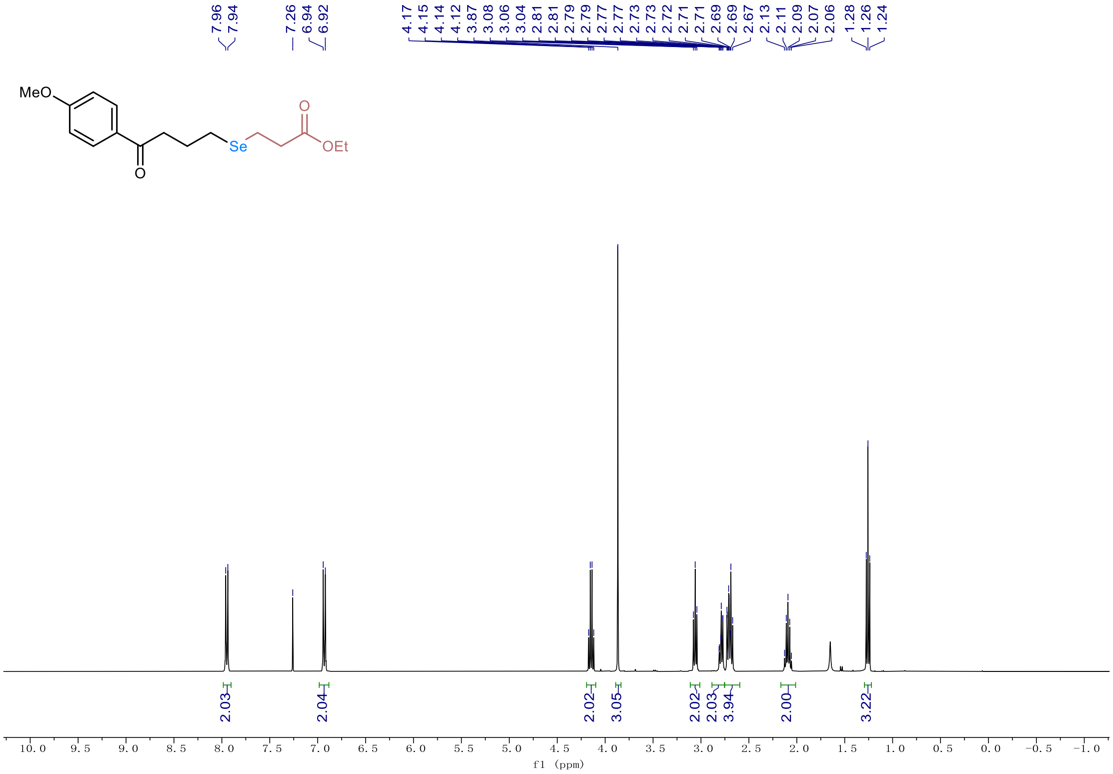

^{13}C NMR of 3ae (101 MHz, Chloroform-*d*)

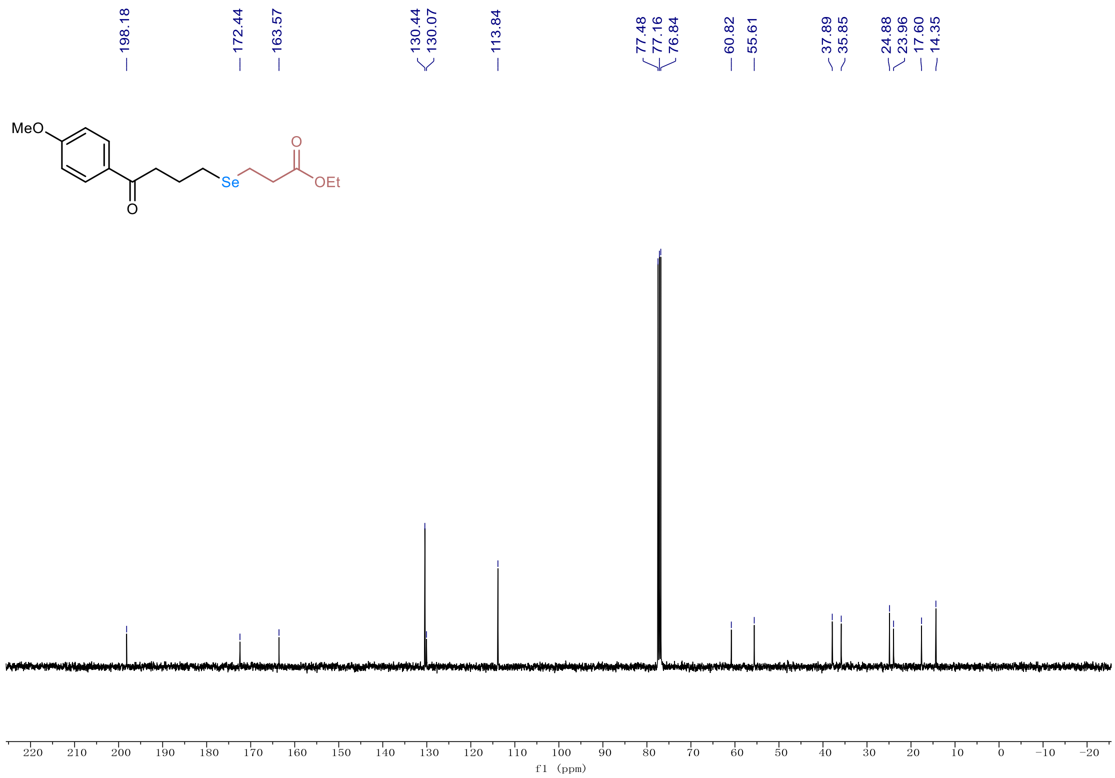

^{1}H NMR of 3af (400 MHz, Chloroform-*d*)

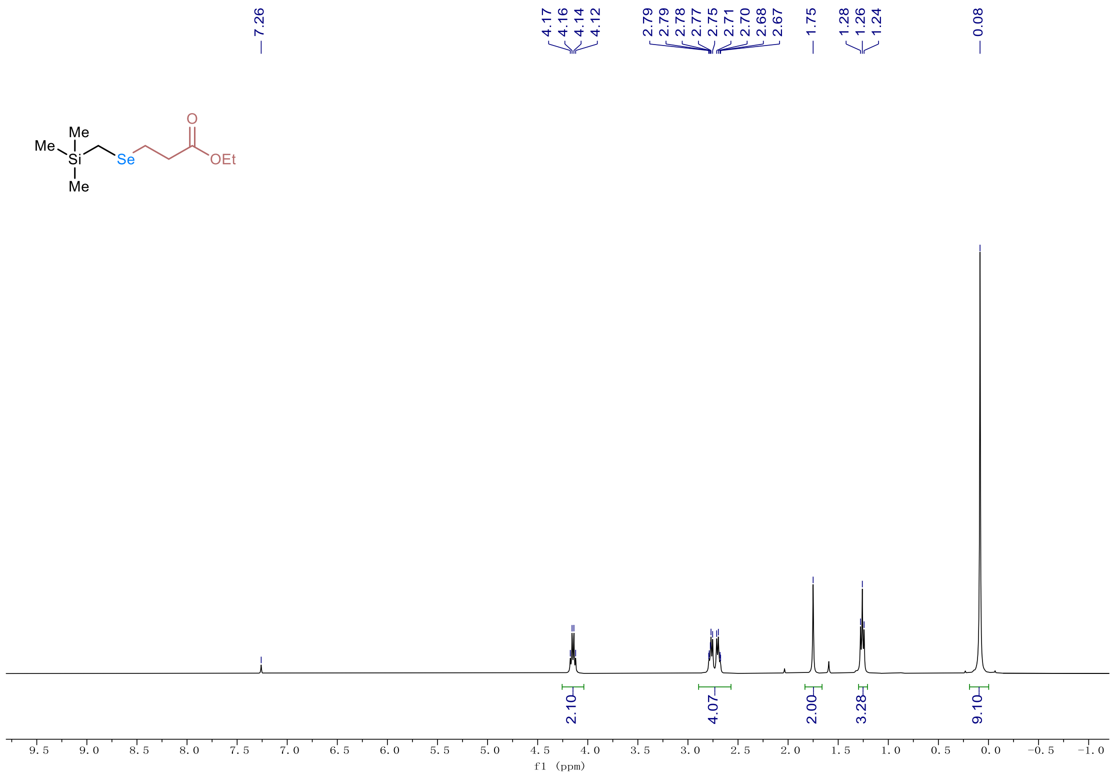

^{13}C NMR of 3af (101 MHz, Chloroform-*d*)

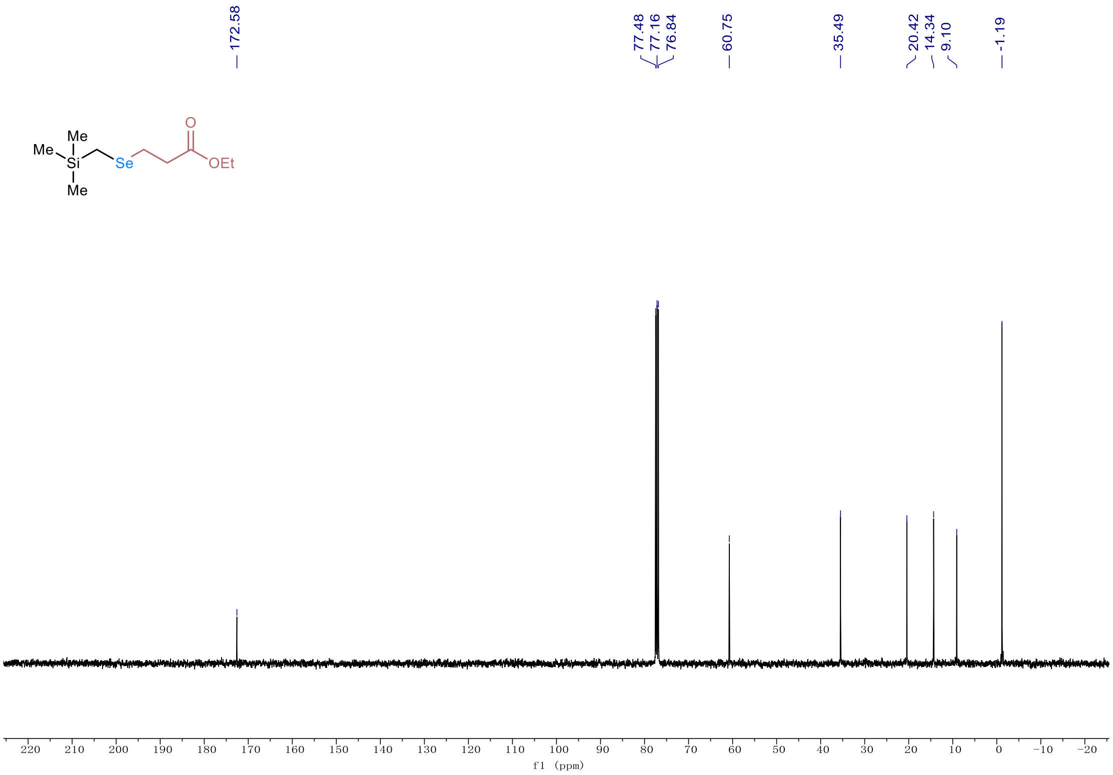

^{1}H NMR of 3ag (400 MHz, Chloroform-*d*)

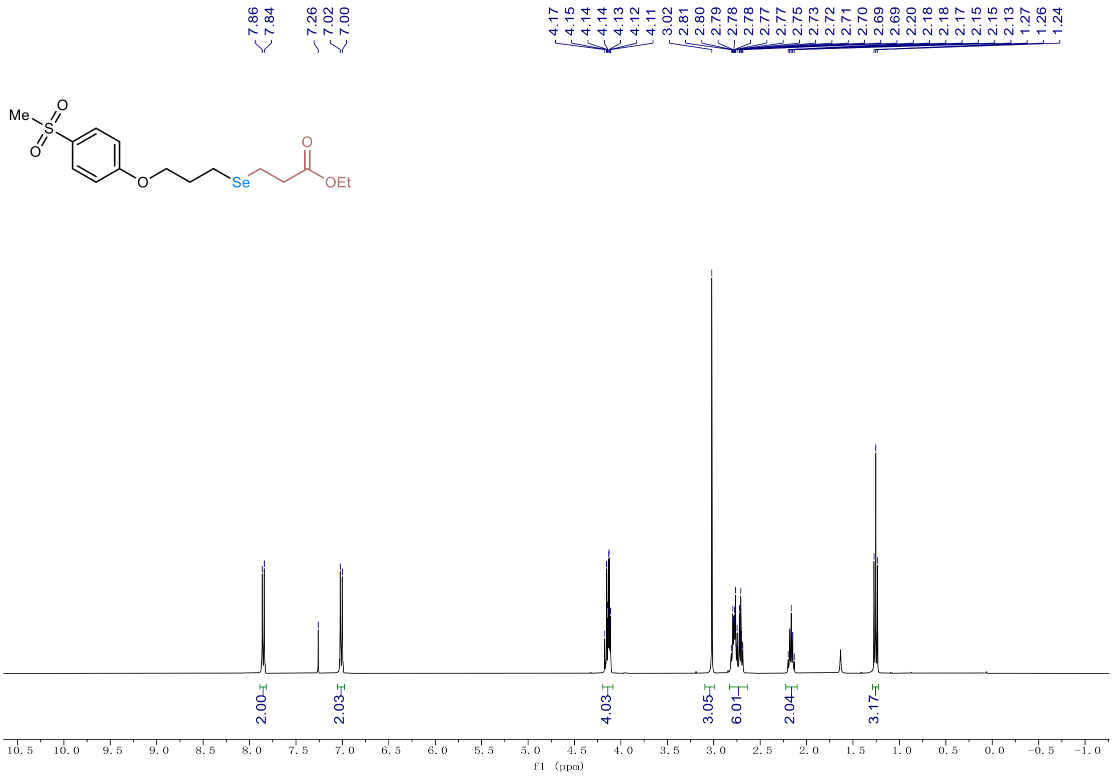

^{13}C NMR of 3ag (101 MHz, Chloroform-*d*)

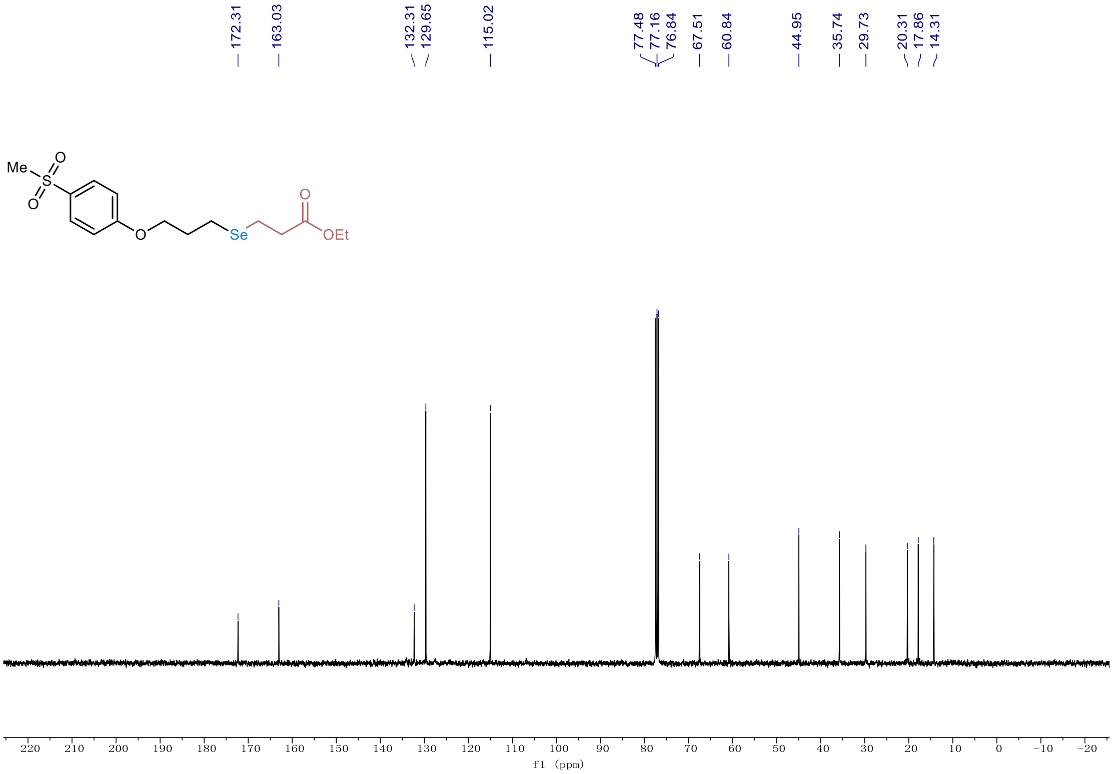

^{1}H NMR of 3ah (400 MHz, Chloroform-*d*)

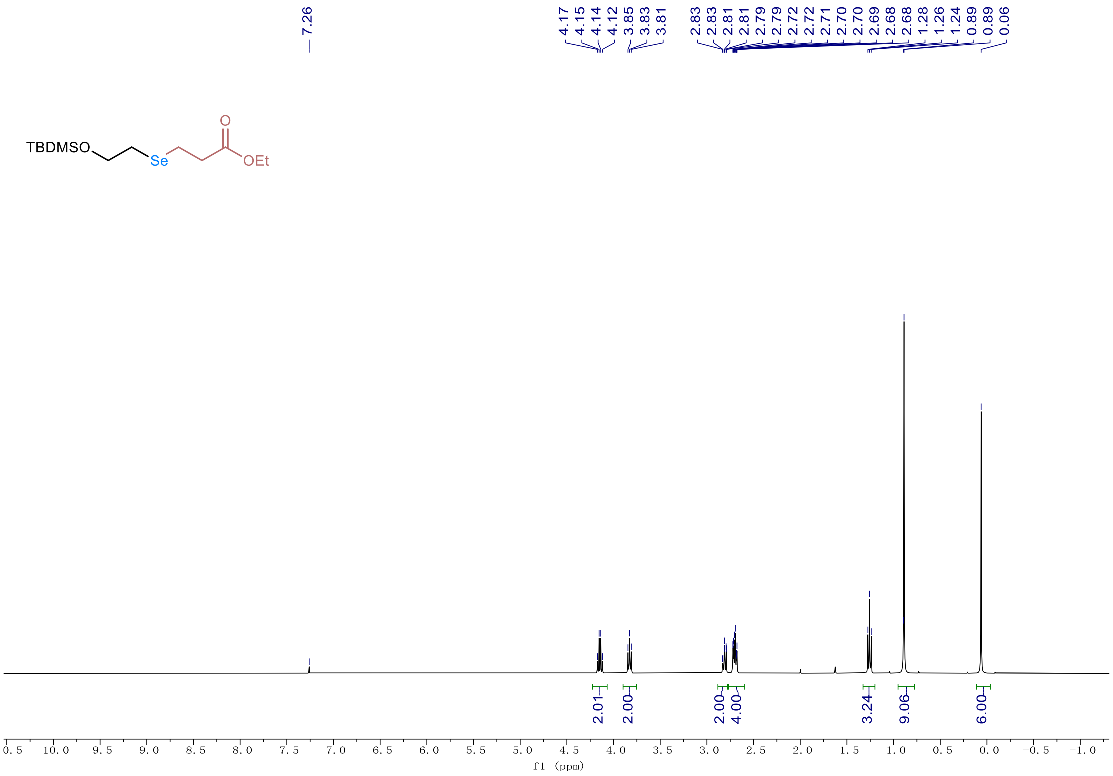

^{13}C NMR of 3ah (101 MHz, Chloroform-*d*)

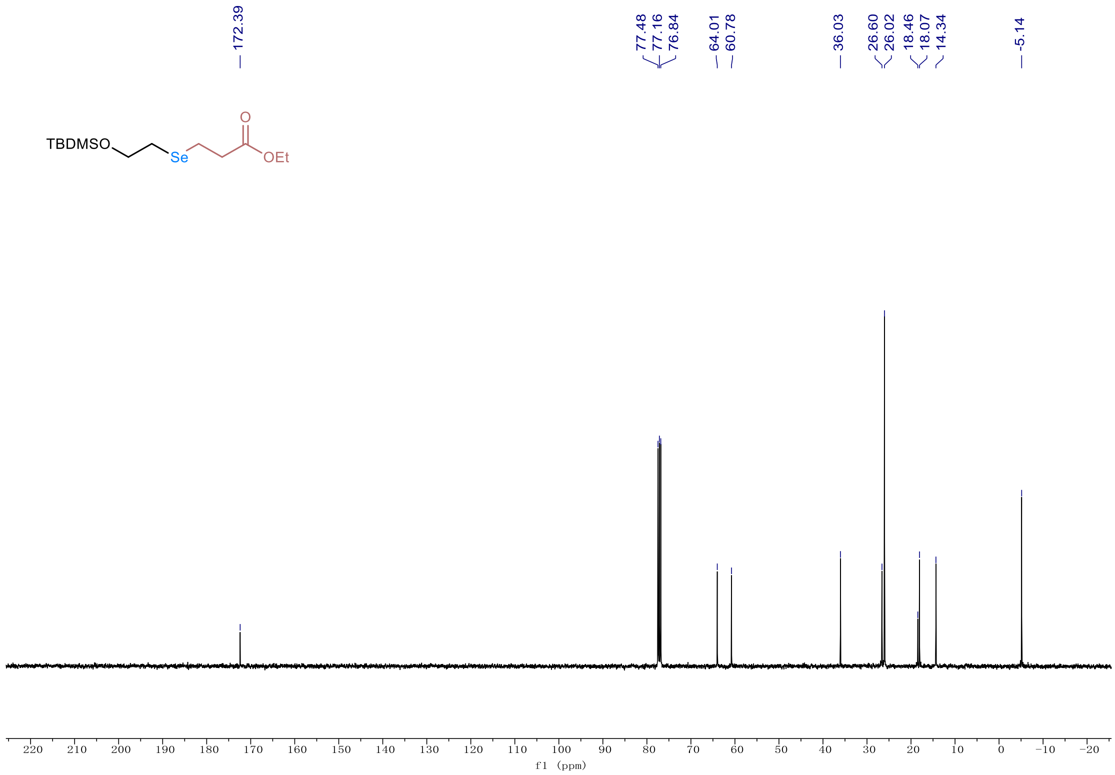

^{1}H NMR of 3ai (400 MHz, Chloroform-*d*)

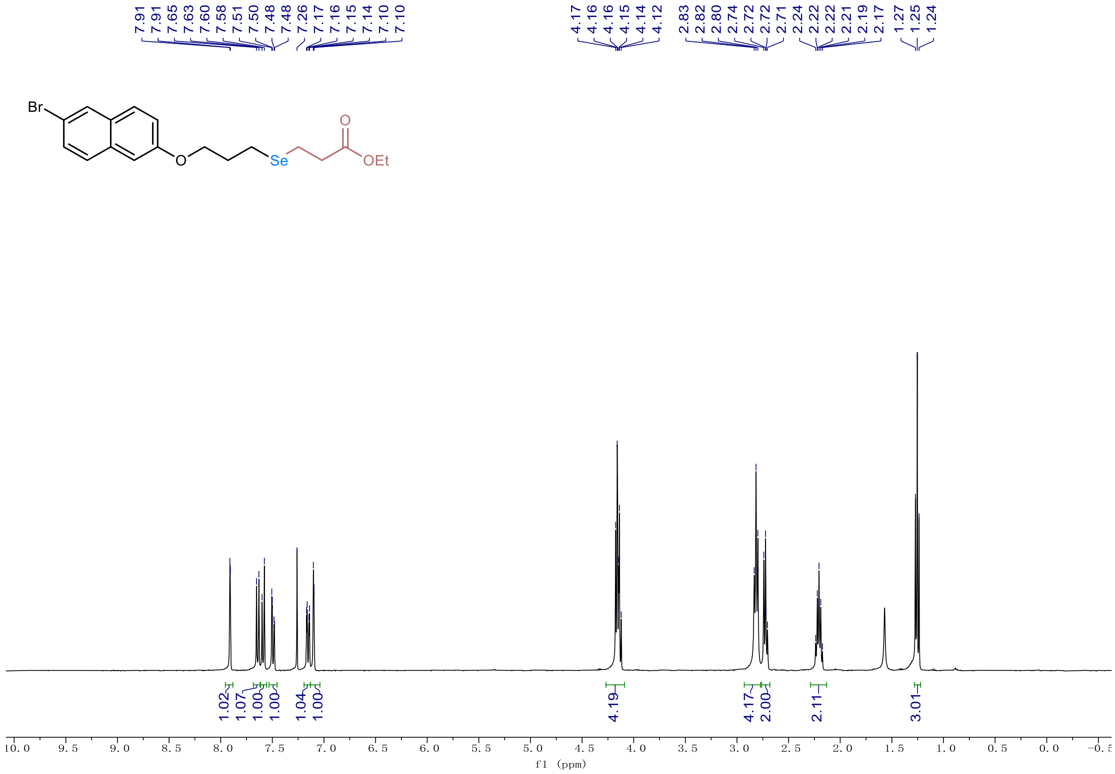

^{13}C NMR of 3ai (101 MHz, Chloroform-*d*)

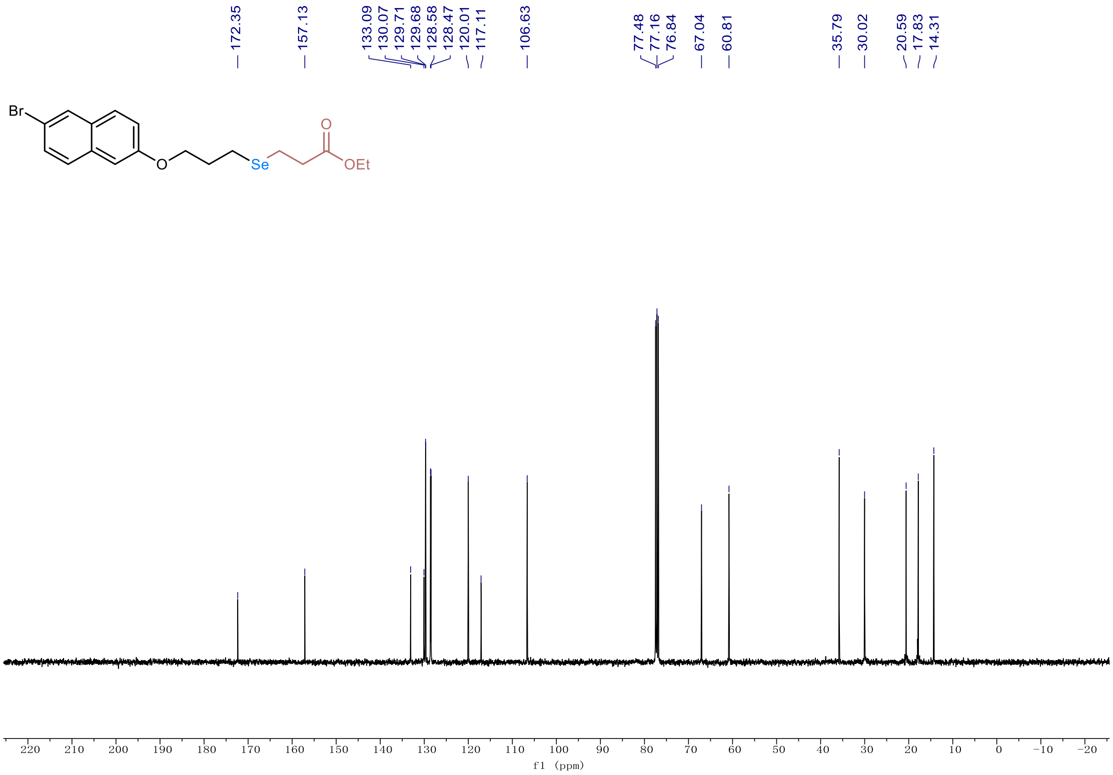

^{1}H NMR of 3aj (400 MHz, Chloroform-*d*)

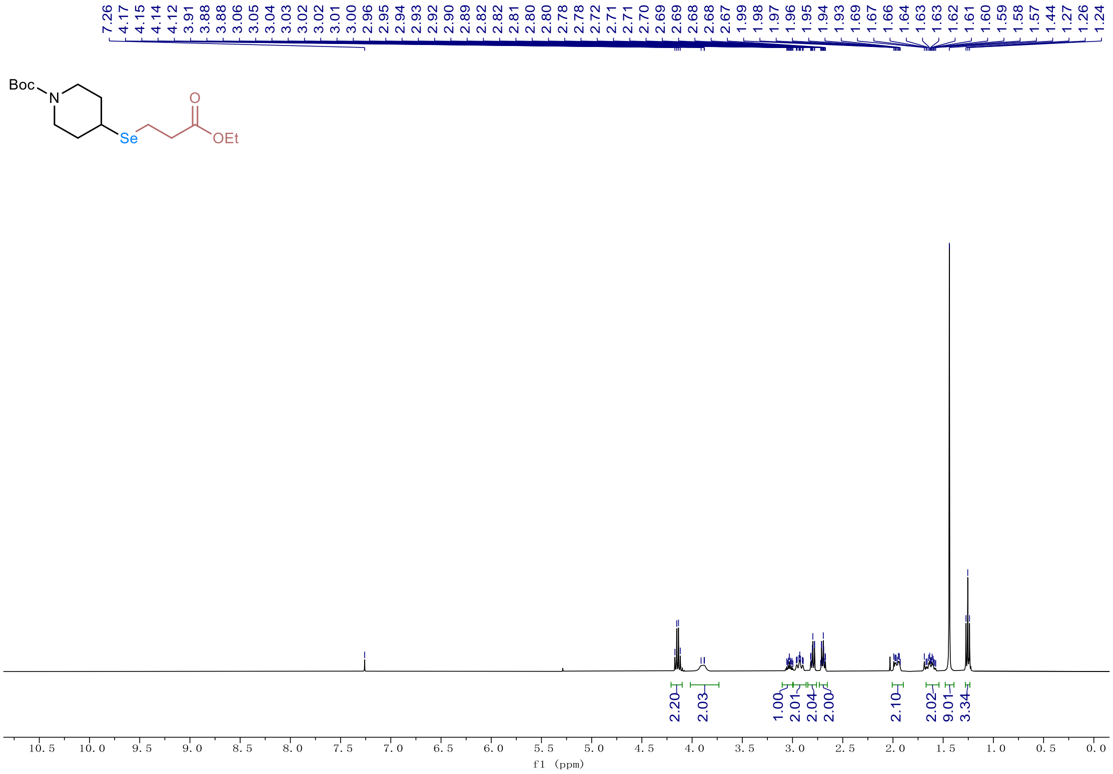

^{13}C NMR of 3aj (101 MHz, Chloroform-*d*)

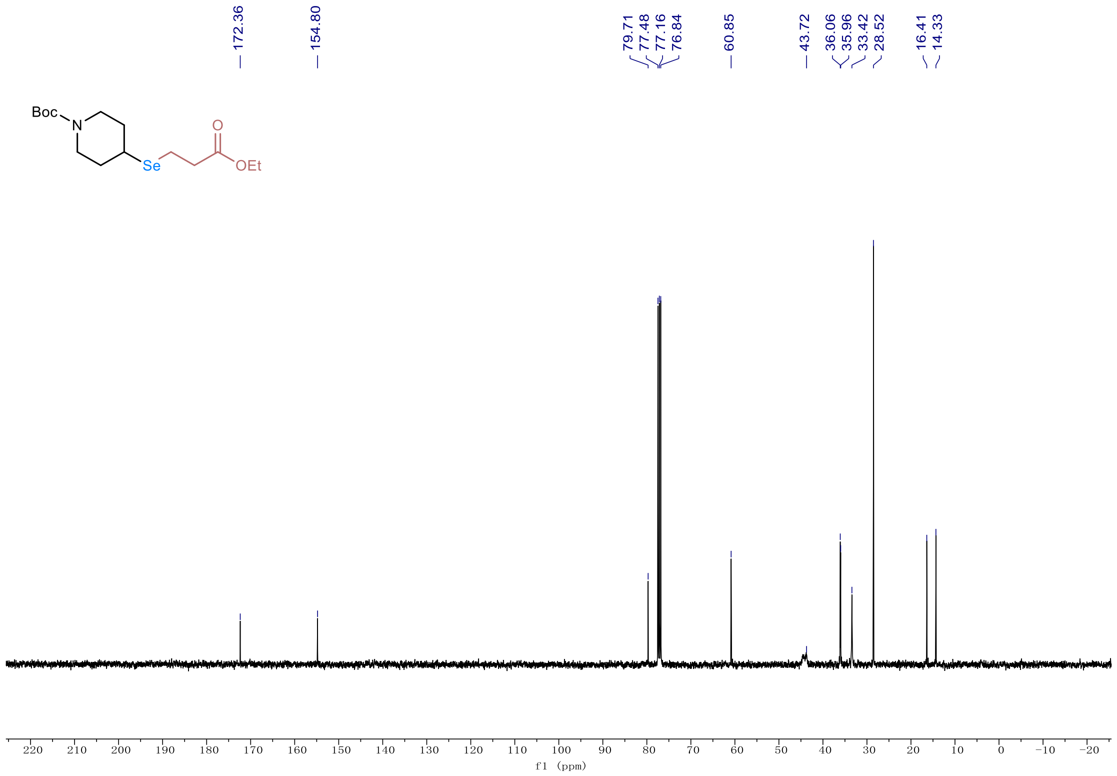

^{1}H NMR of 3ak (400 MHz, Chloroform-*d*)

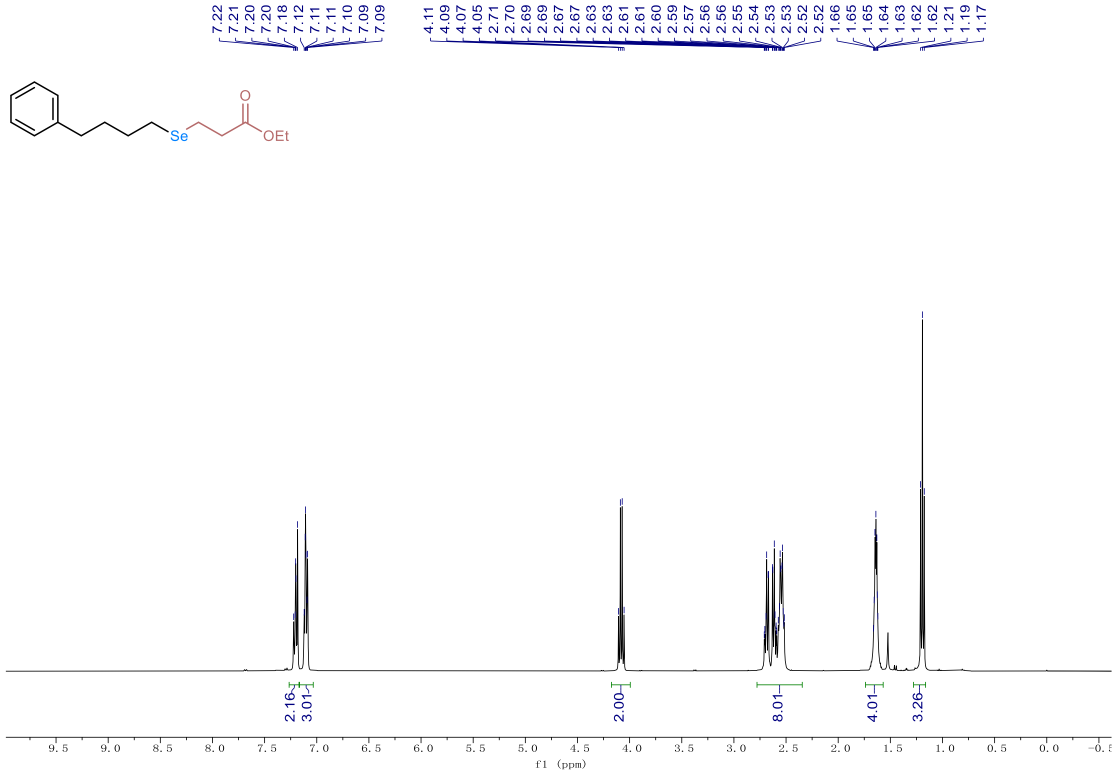

^{13}C NMR of 3ak (101 MHz, Chloroform-*d*)

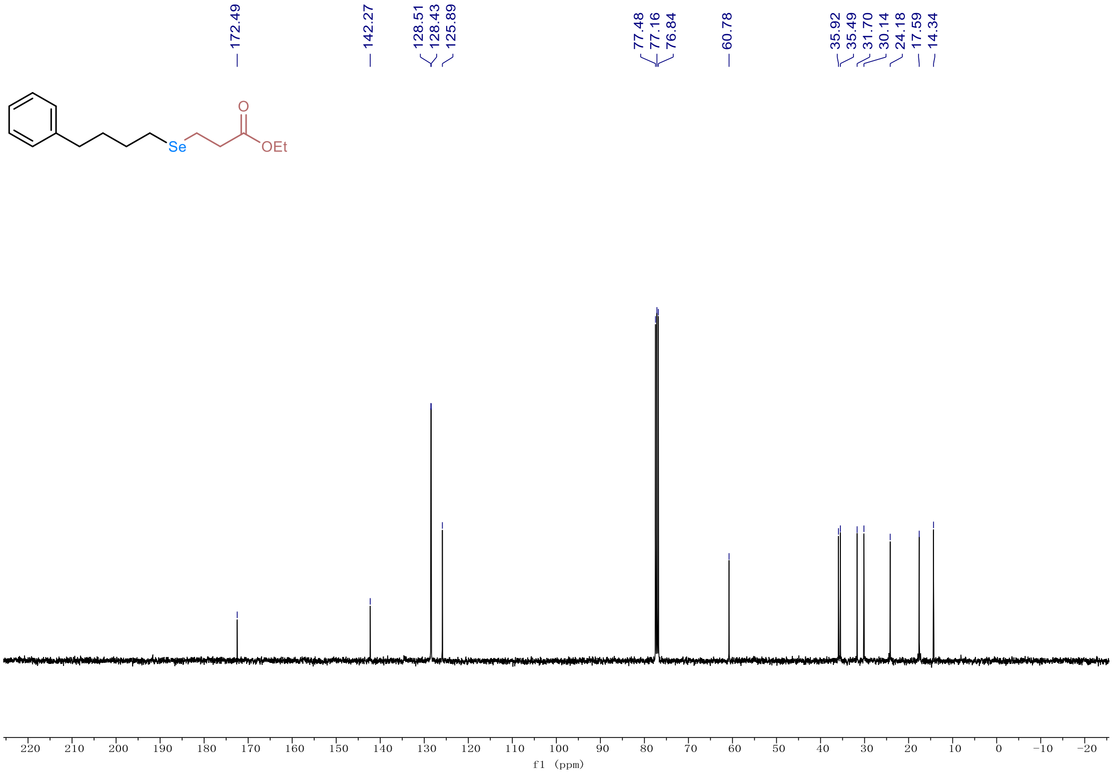

^{1}H NMR of 3al (400 MHz, Chloroform-*d*)

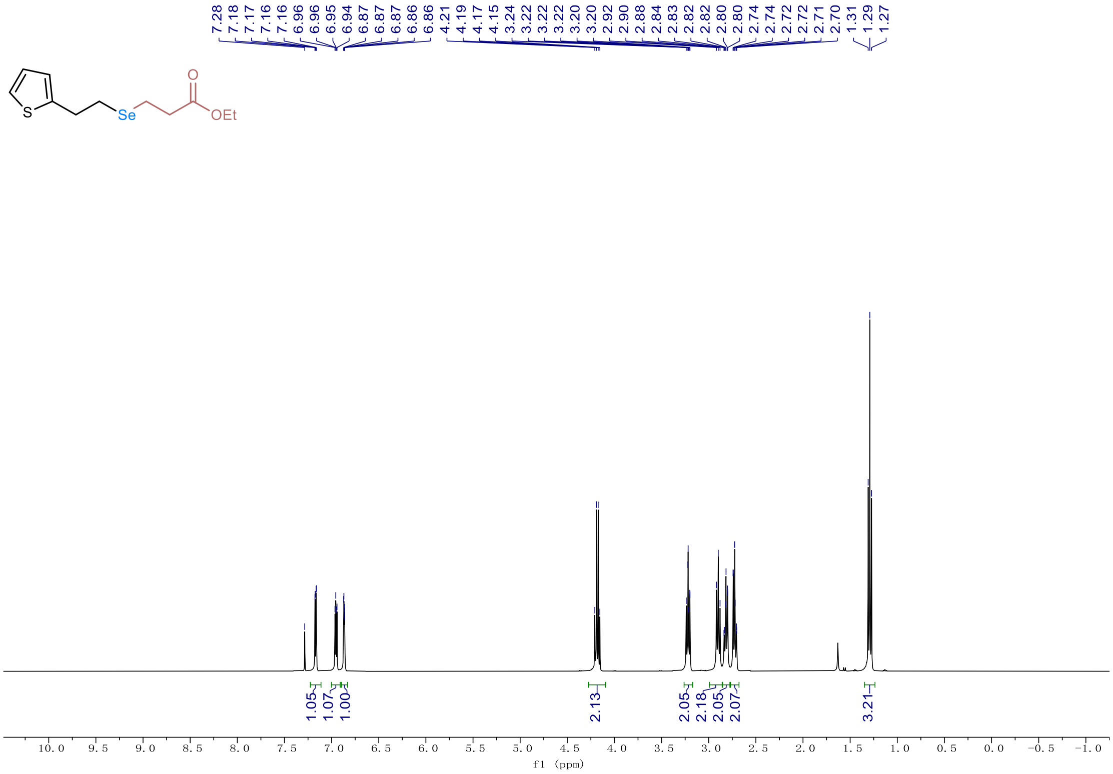

^{13}C NMR of 3al (101 MHz, Chloroform-*d*)

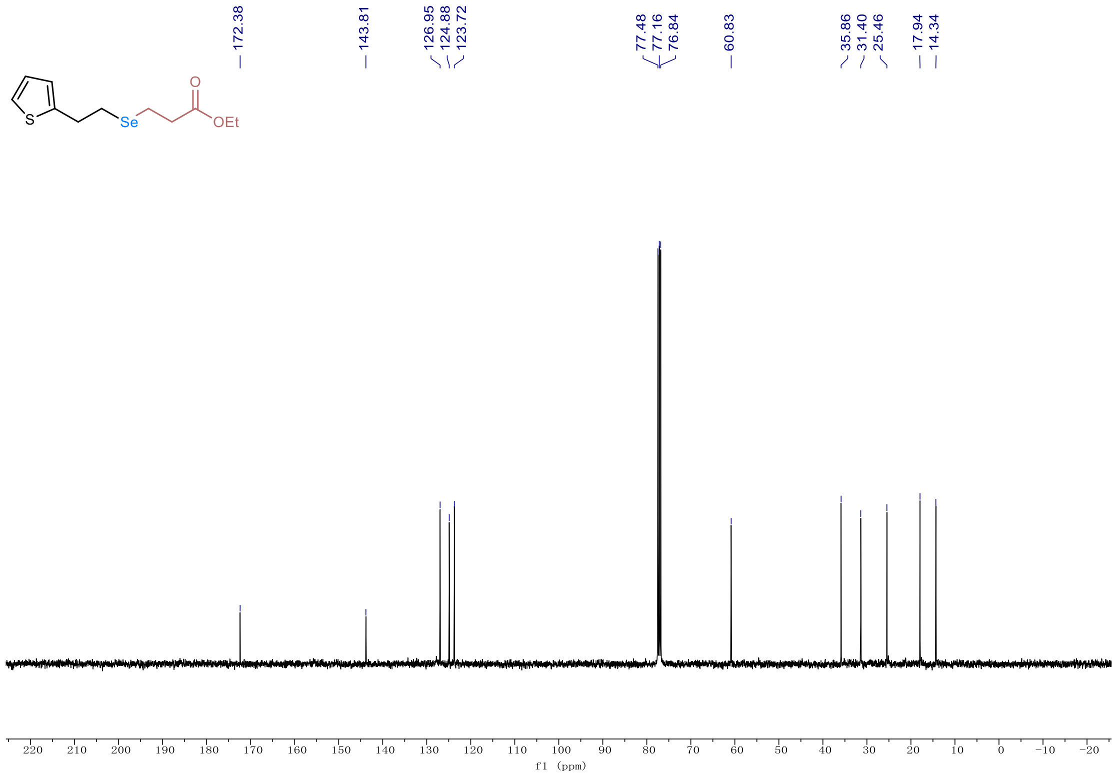

^{1}H NMR of 3am (400 MHz, Chloroform-*d*)

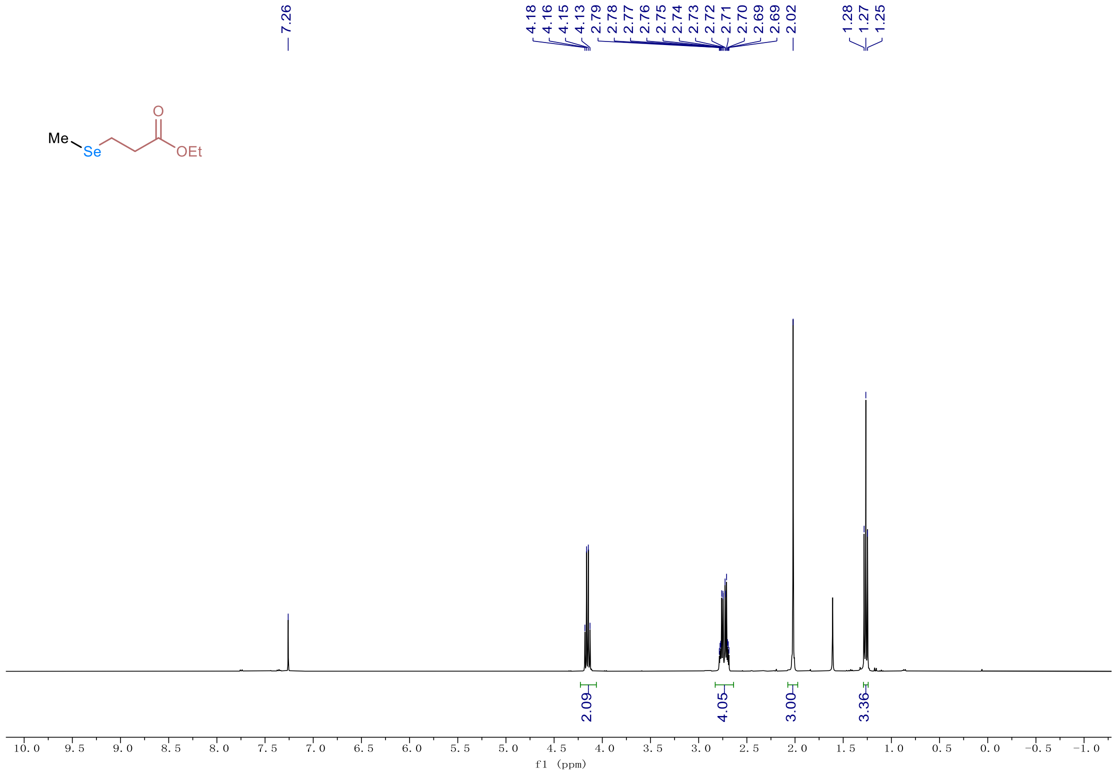

^{13}C NMR of 3am (101 MHz, Chloroform-*d*)

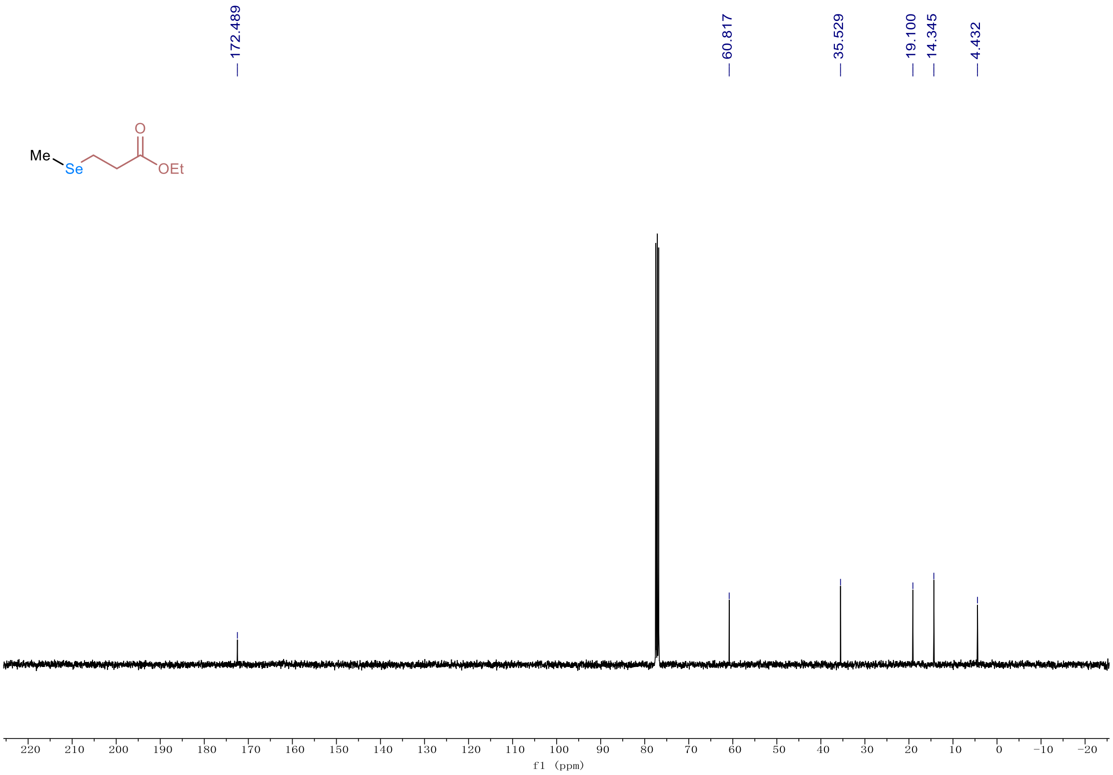

^{1}H NMR of 3an (400 MHz, Chloroform-*d*)

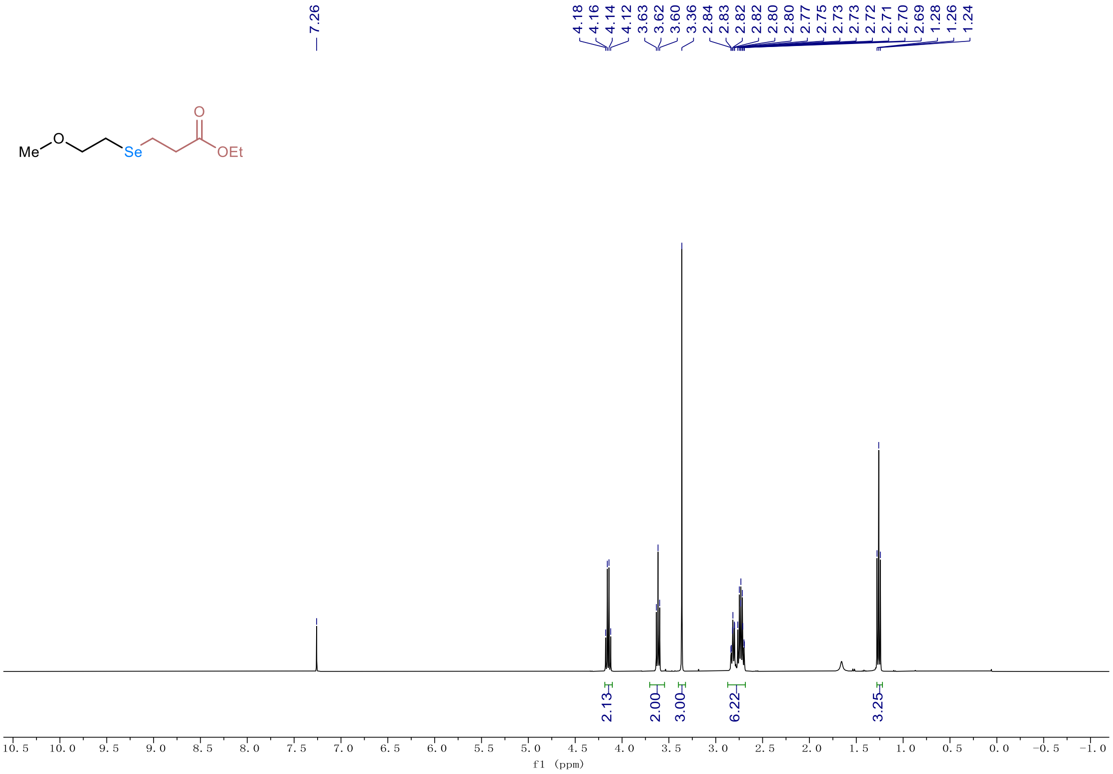

^{13}C NMR of 3an (101 MHz, Chloroform-*d*)

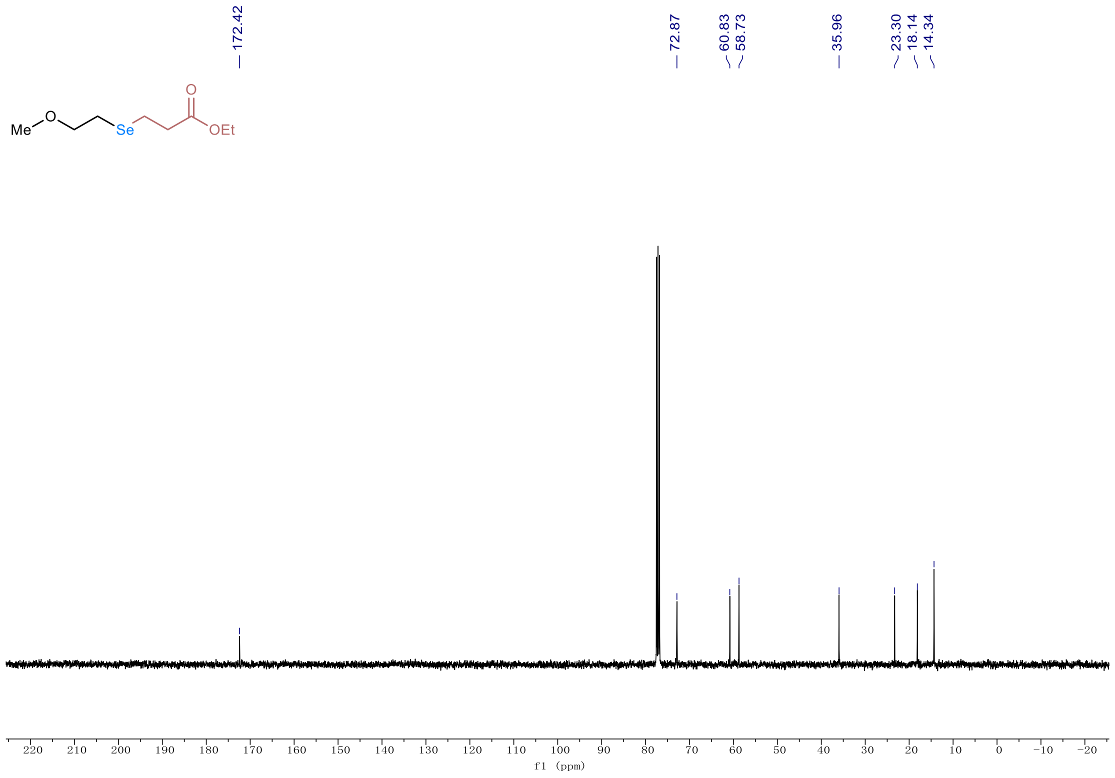

^{1}H NMR of 4a (400 MHz, Chloroform-*d*)

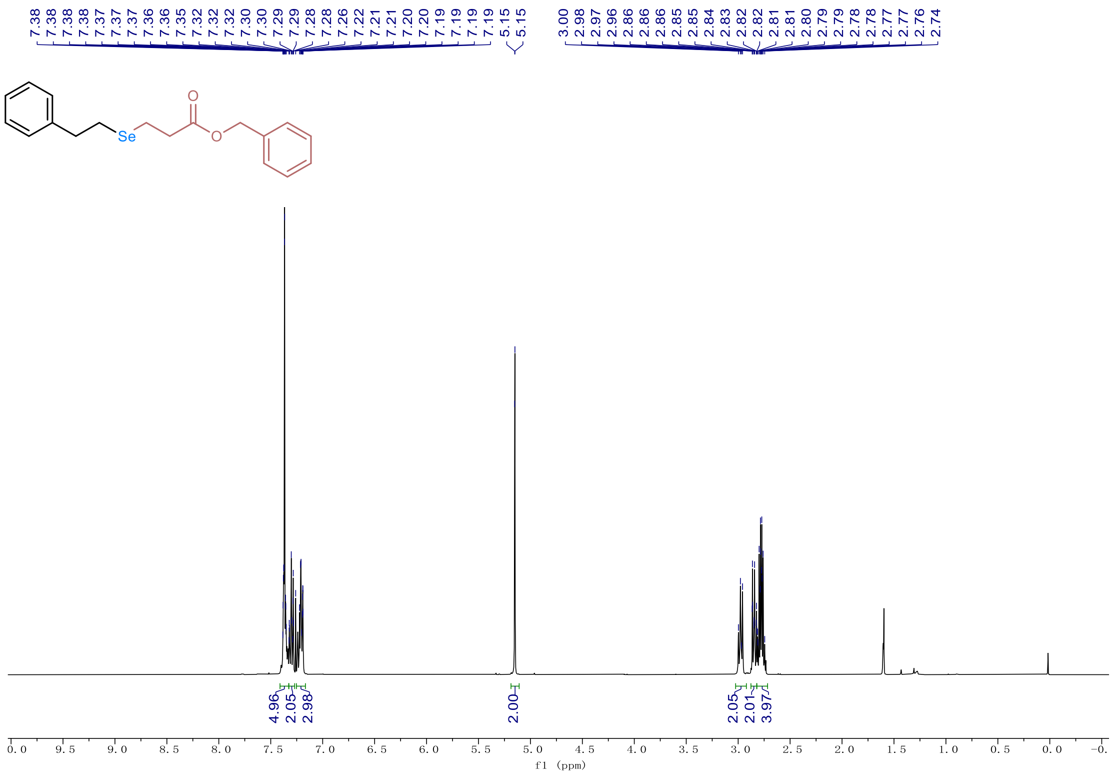

^{13}C NMR of 4a (101 MHz, Chloroform-*d*)

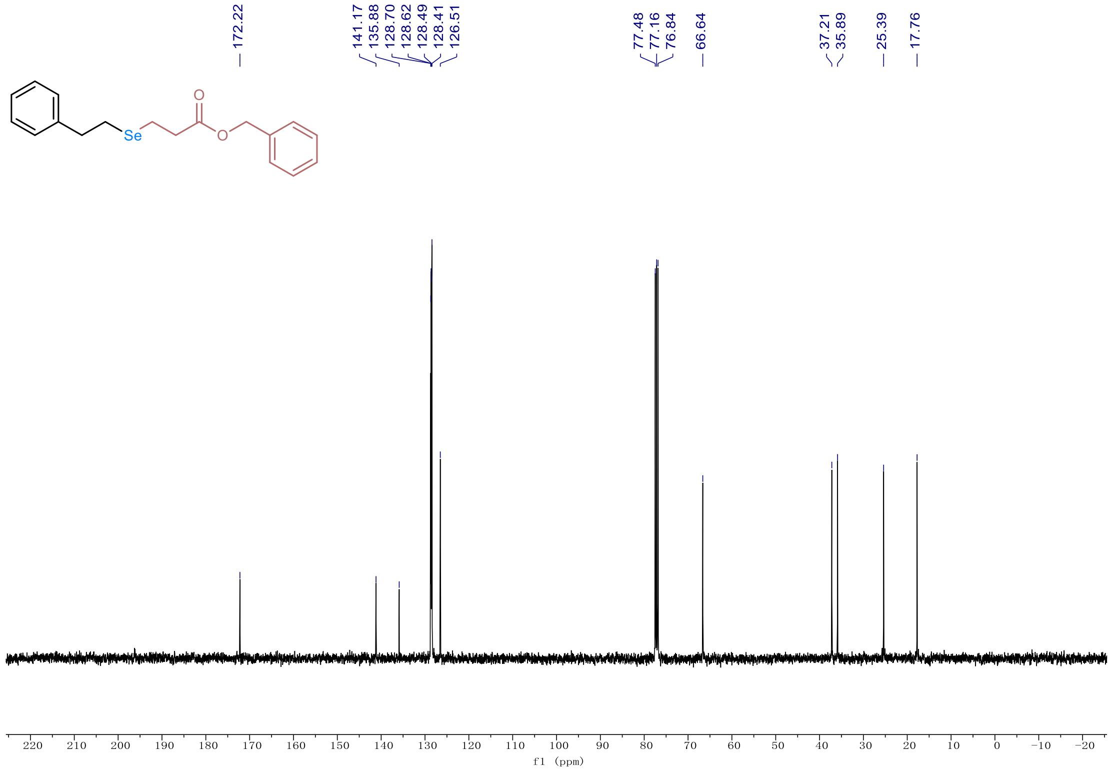

^{1}H NMR of 4b (400 MHz, Chloroform-*d*)

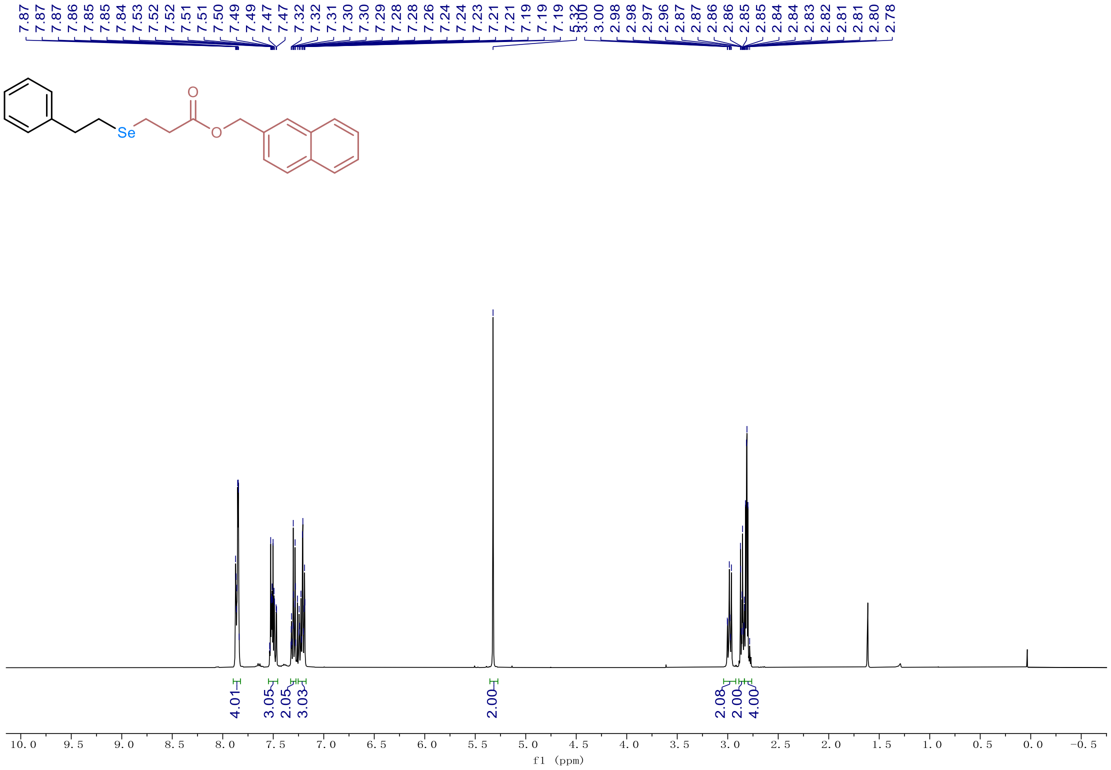

^{13}C NMR of 4b (101 MHz, Chloroform-*d*)

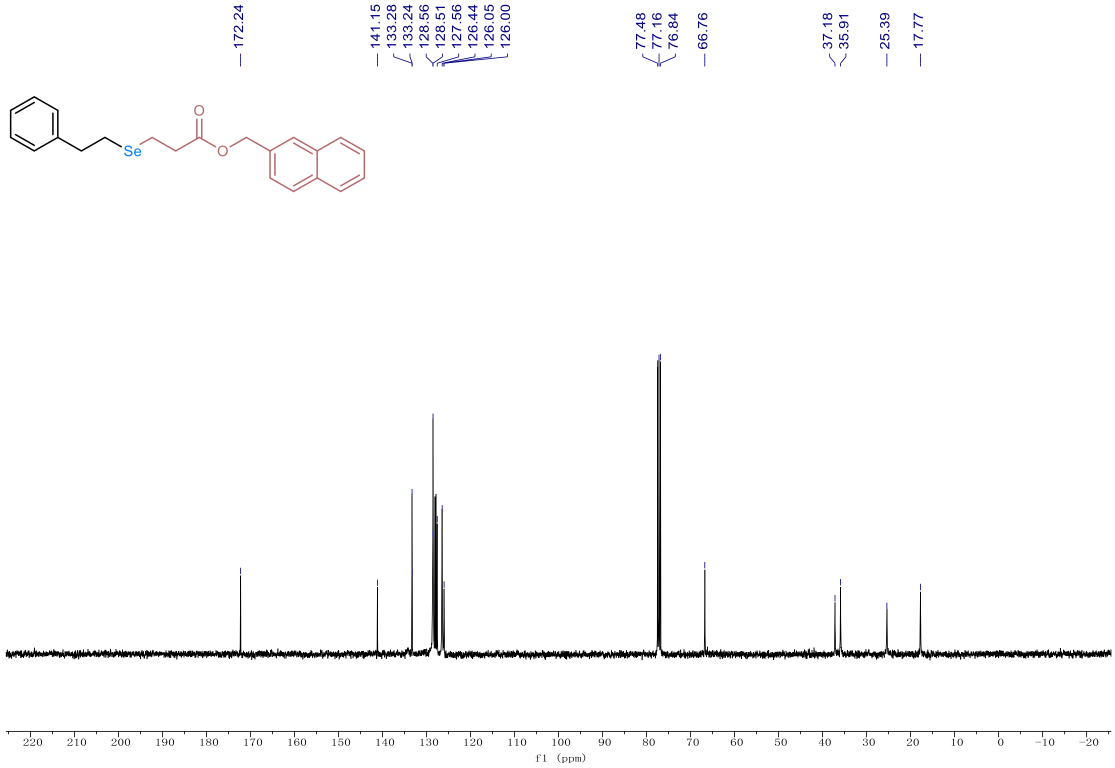

^{1}H NMR of 4c (400 MHz, Chloroform-*d*)

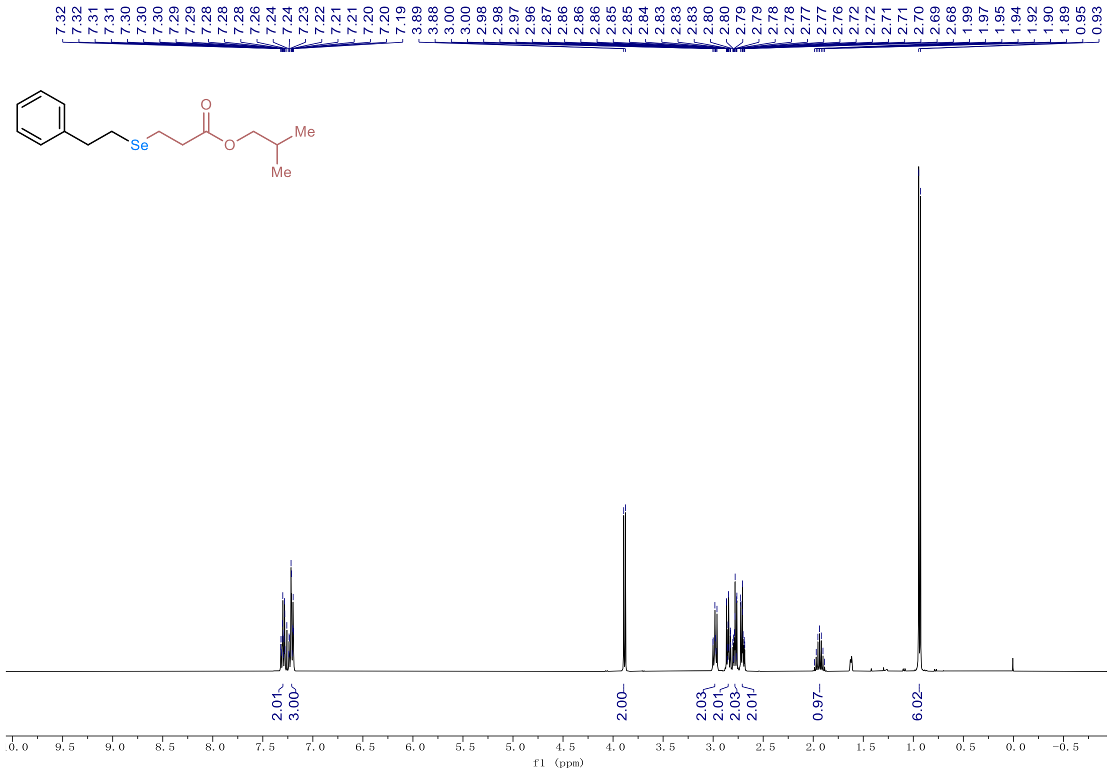

^{13}C NMR of 4c (101 MHz, Chloroform-*d*)

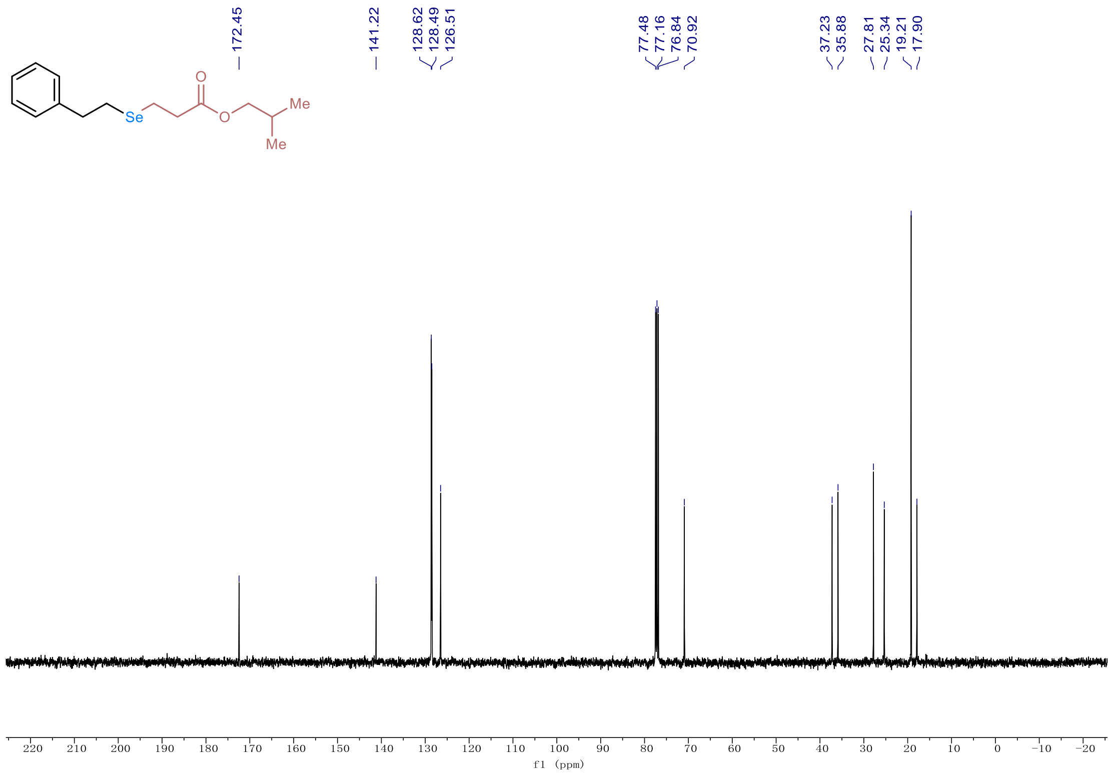

^{1}H NMR of 4d (400 MHz, Chloroform-*d*)

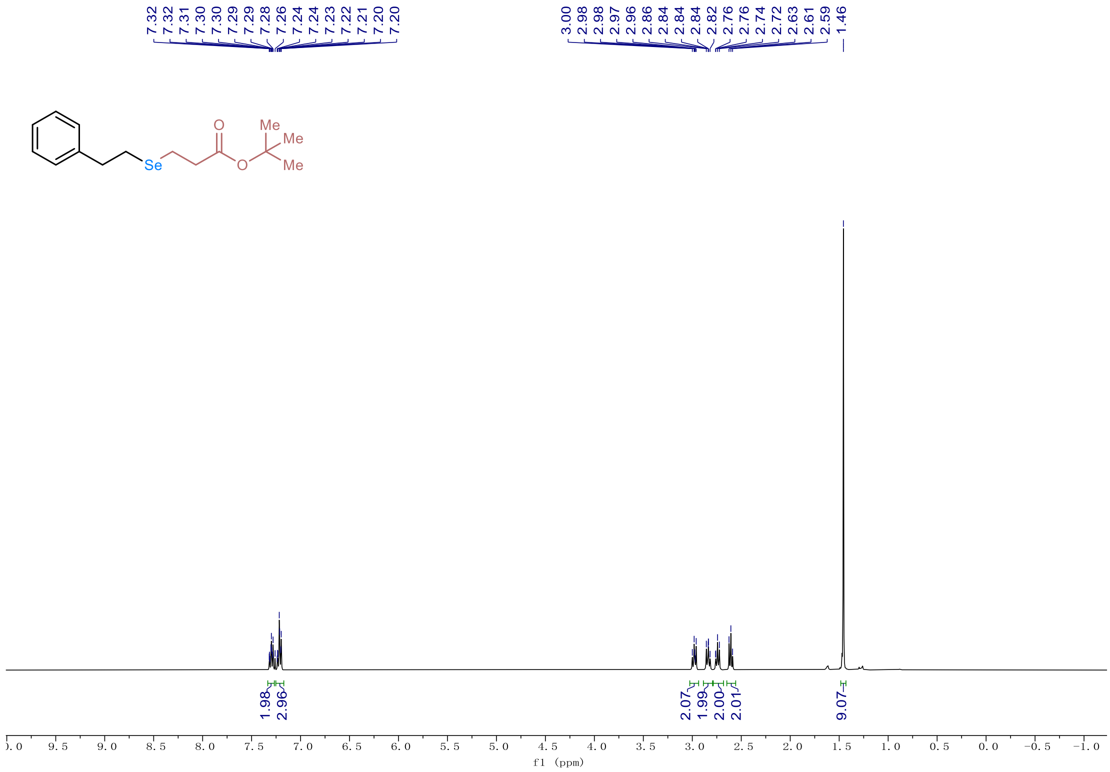

^{13}C NMR of 4d (101 MHz, Chloroform-*d*)

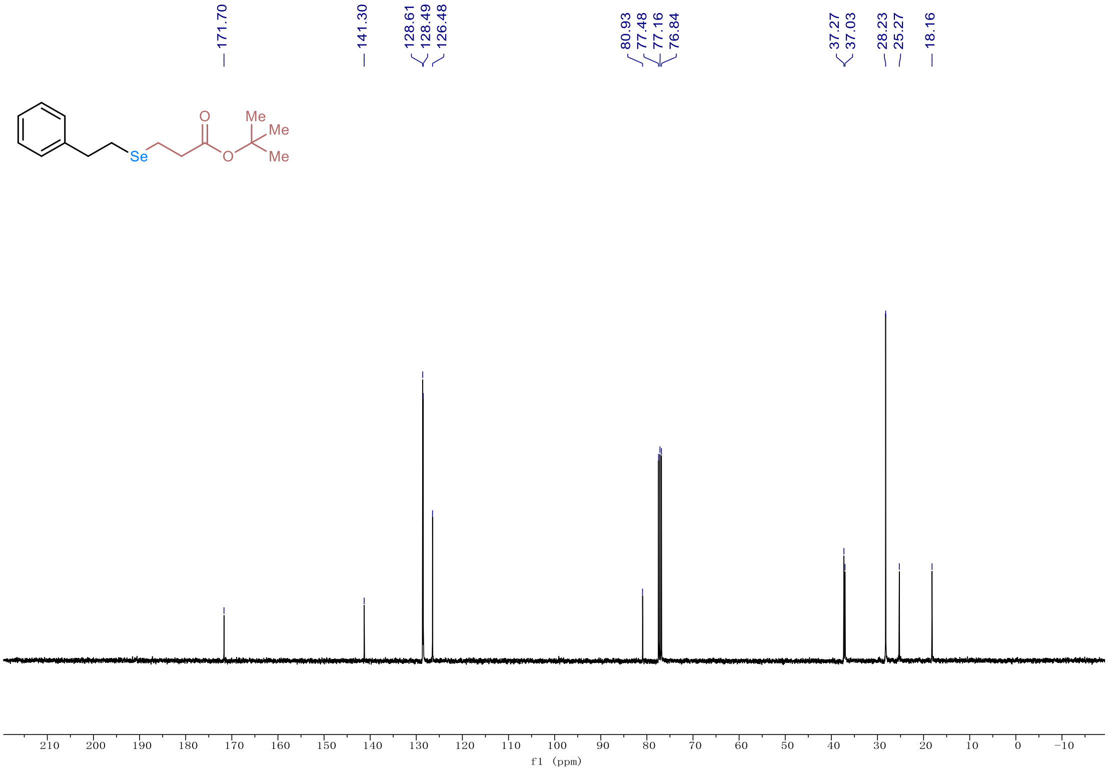

^{1}H NMR of 4e (400 MHz, Chloroform-*d*)

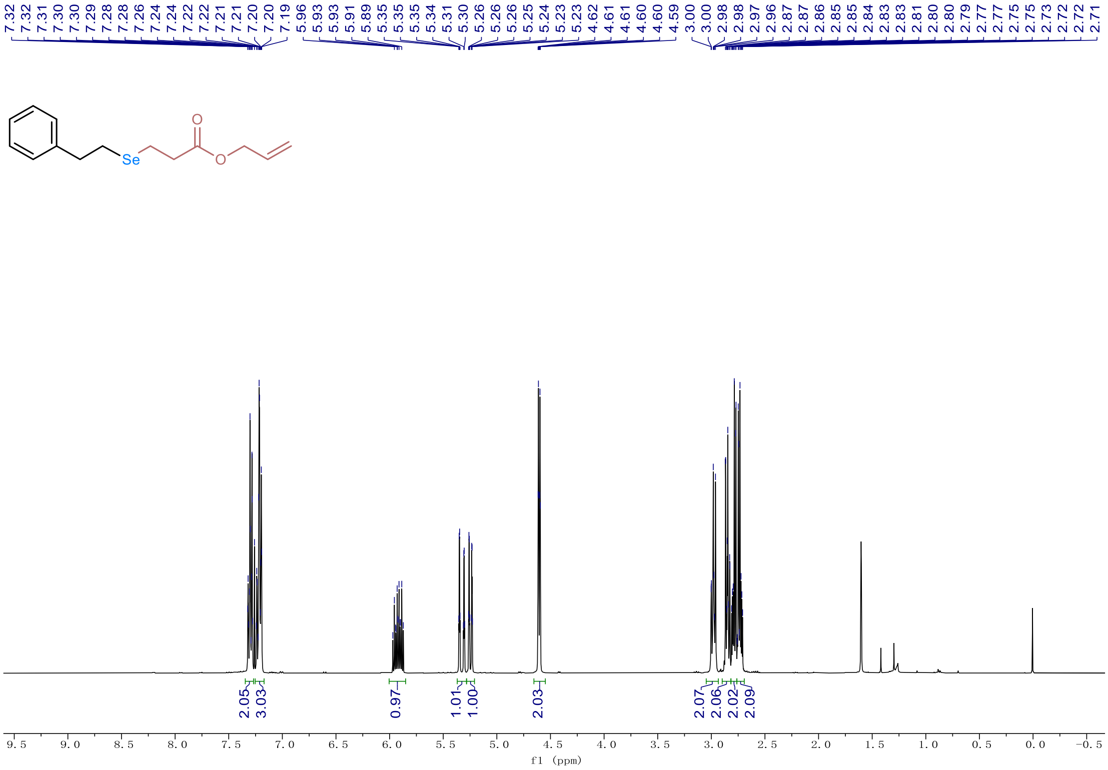

^{13}C NMR of 4e (101 MHz, Chloroform-*d*)

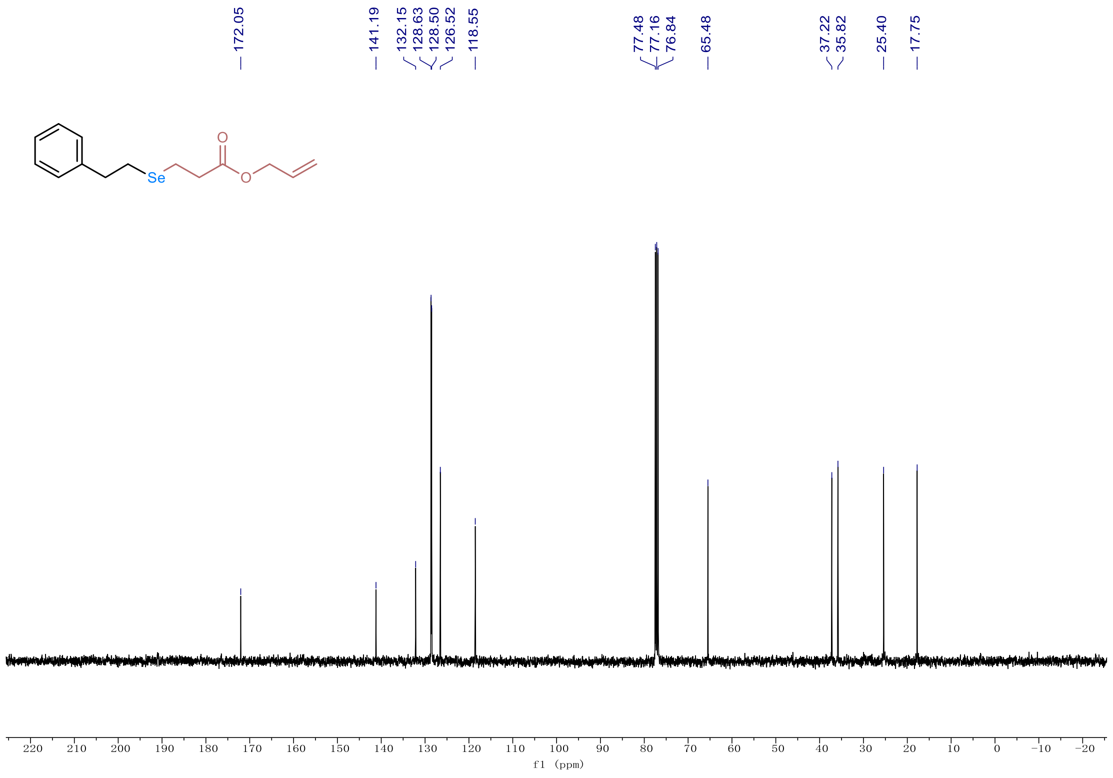

^{1}H NMR of 4f (400 MHz, Chloroform-*d*)

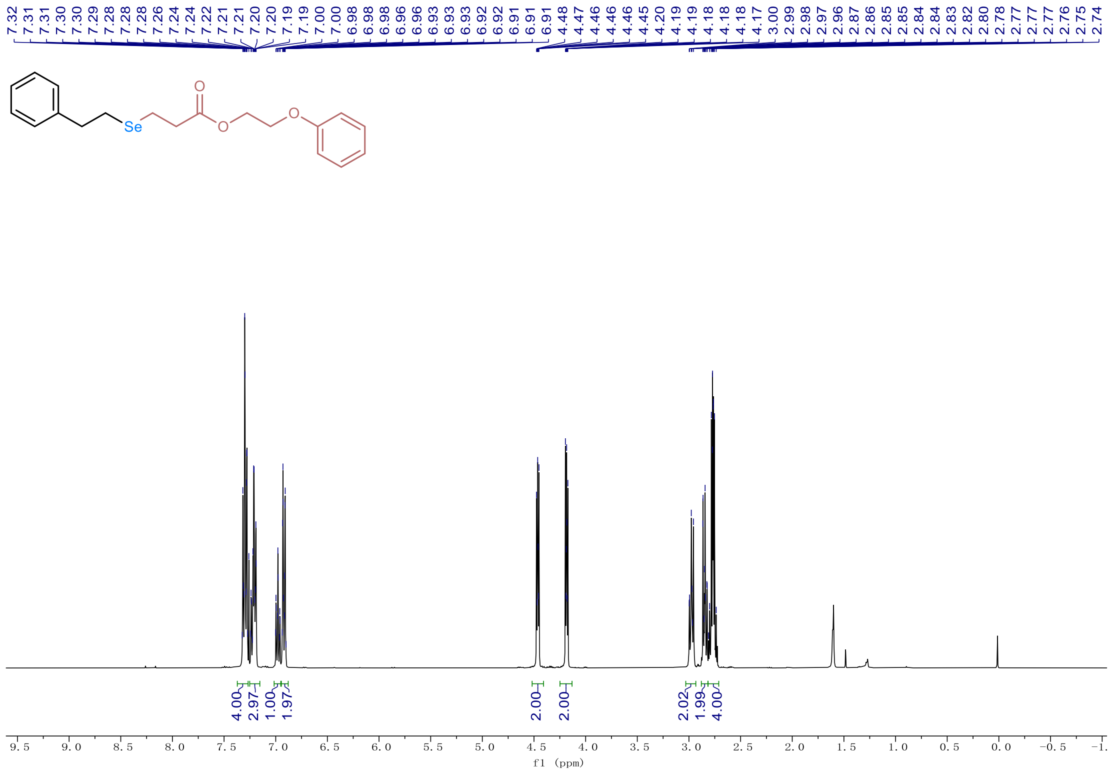

^{13}C NMR of 4f (101 MHz, Chloroform-*d*)

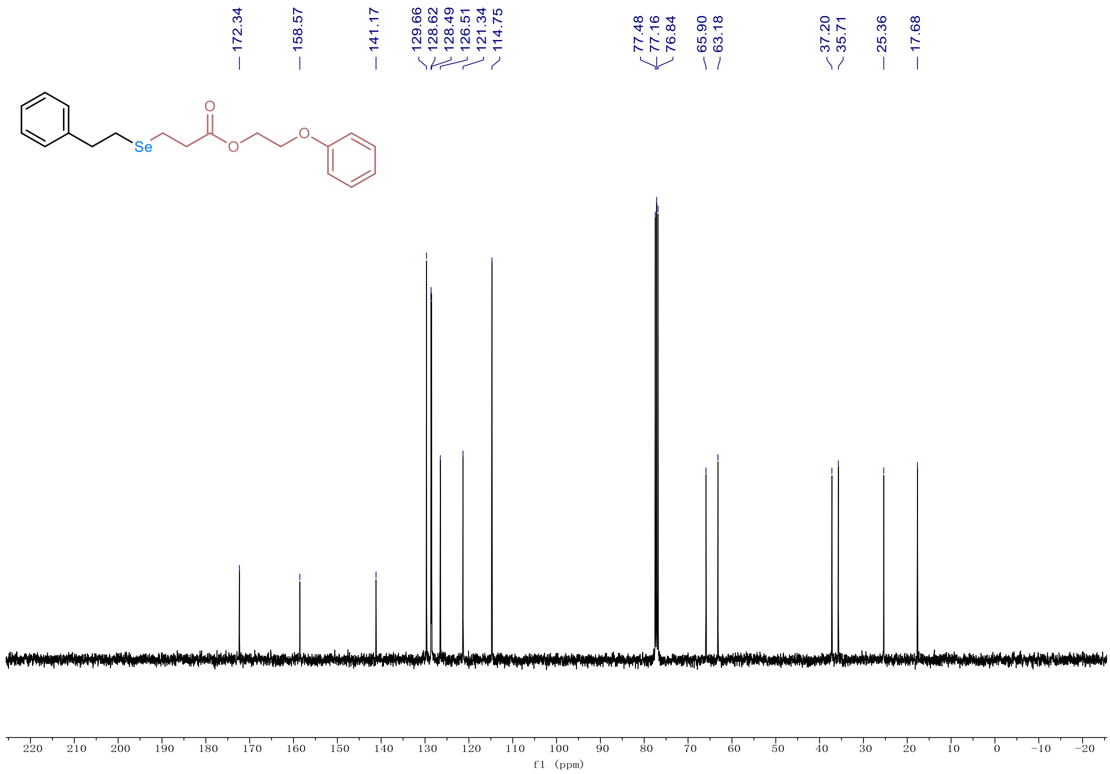

^{1}H NMR of 4g (400 MHz, Chloroform-*d*)

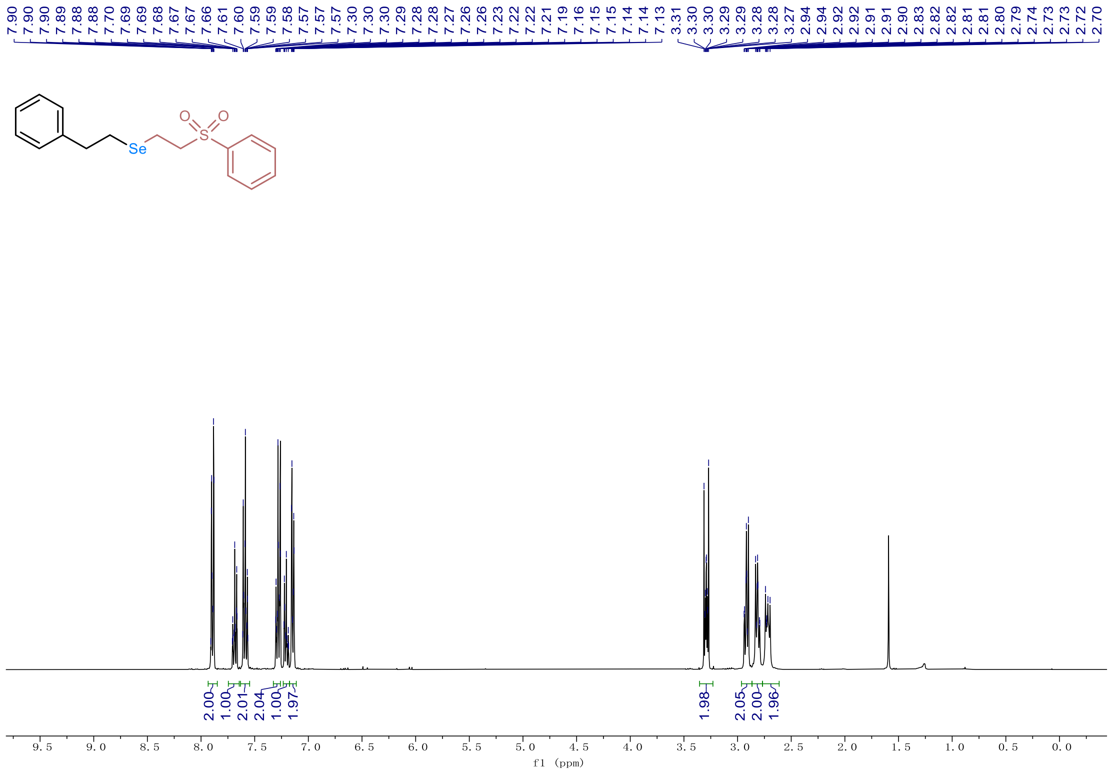

^{13}C NMR of 4g (101 MHz, Chloroform-*d*)

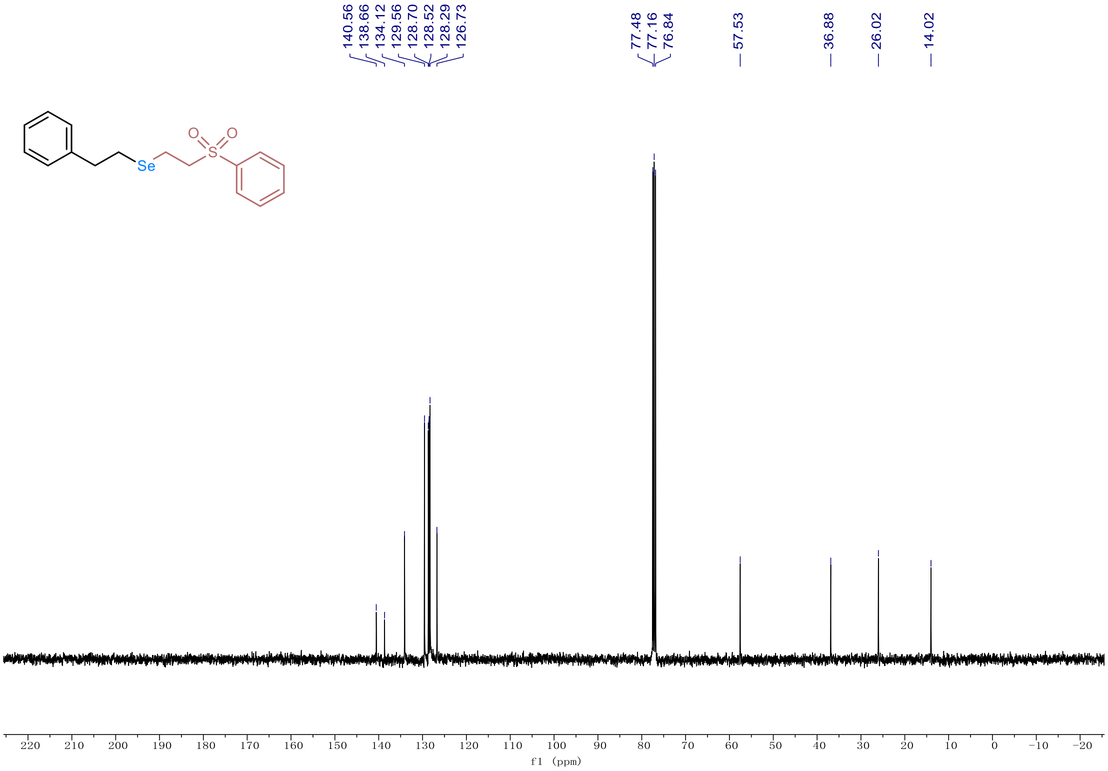

^{1}H NMR of 4h (400 MHz, Chloroform-*d*)

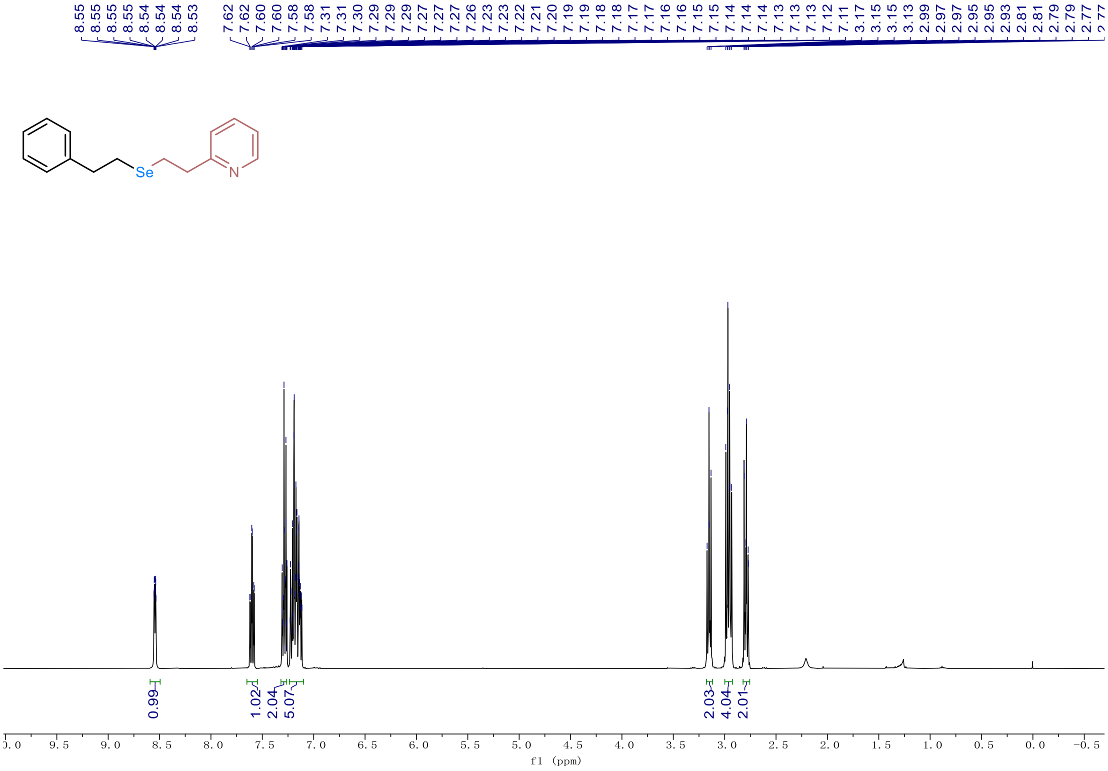

^{13}C NMR of 4h (101 MHz, Chloroform-*d*)

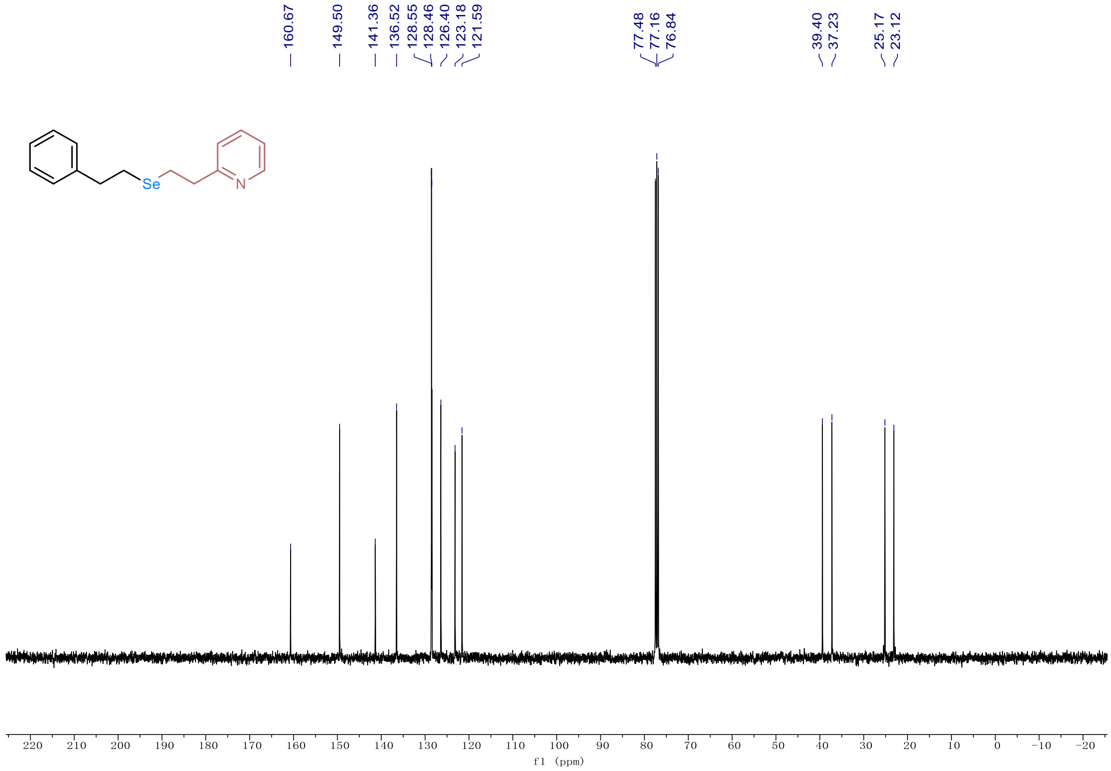

^{1}H NMR of 4i (400 MHz, Chloroform-*d*)

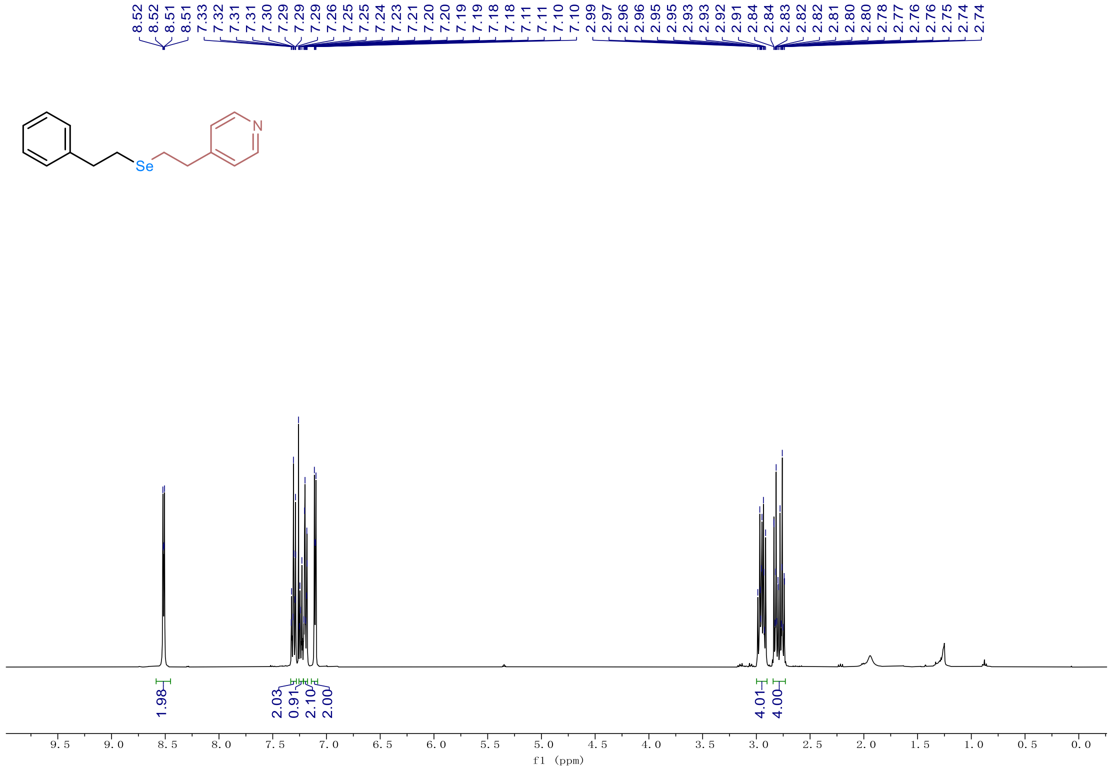

^{13}C NMR of 4i (101 MHz, Chloroform-*d*)

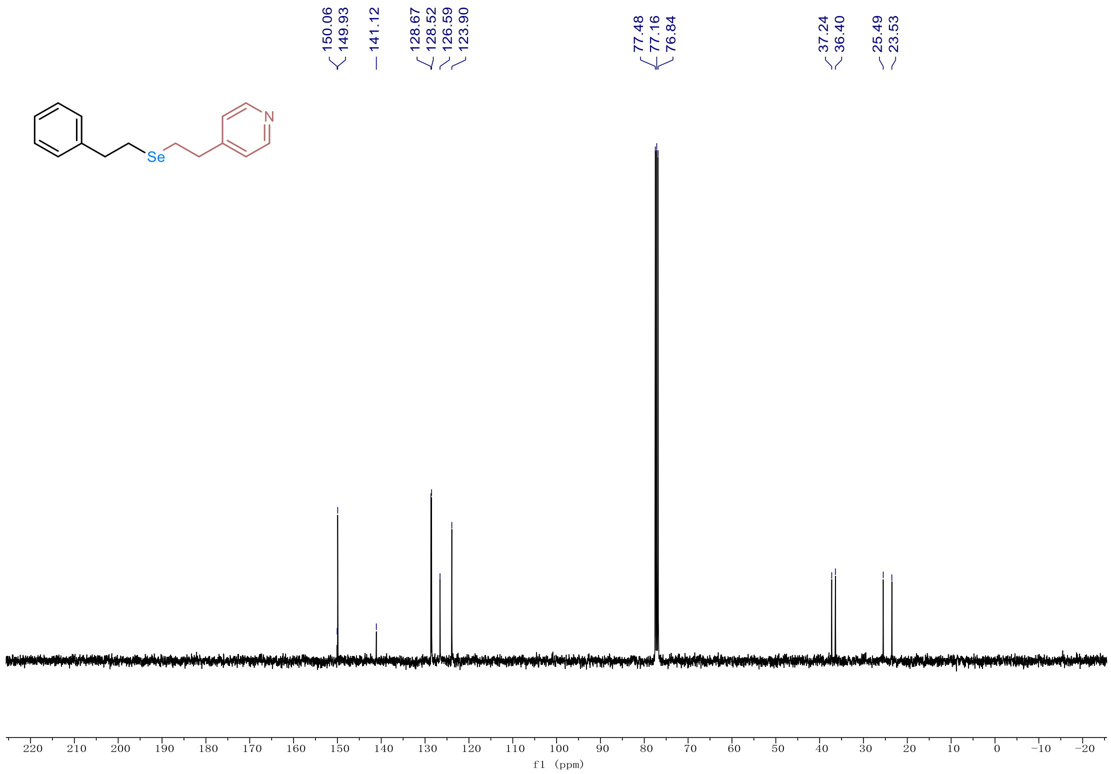

^{1}H NMR of 4j (400 MHz, Chloroform-*d*)

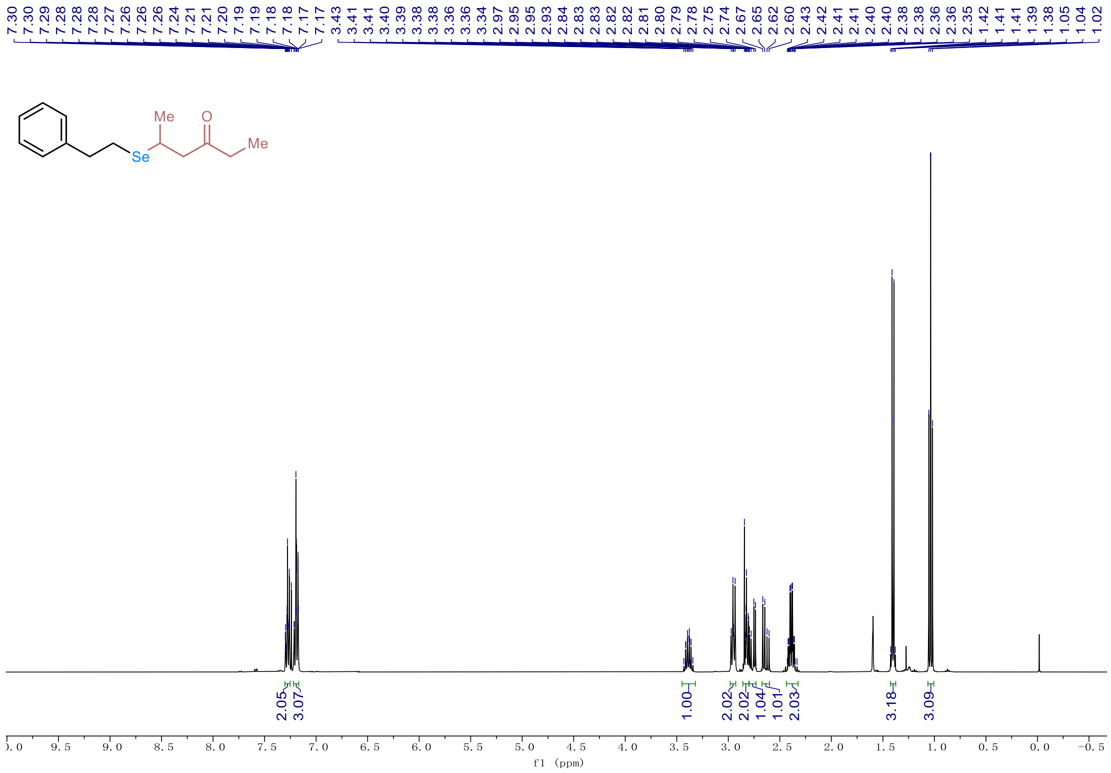

^{13}C NMR of 4j (101 MHz, Chloroform-*d*)

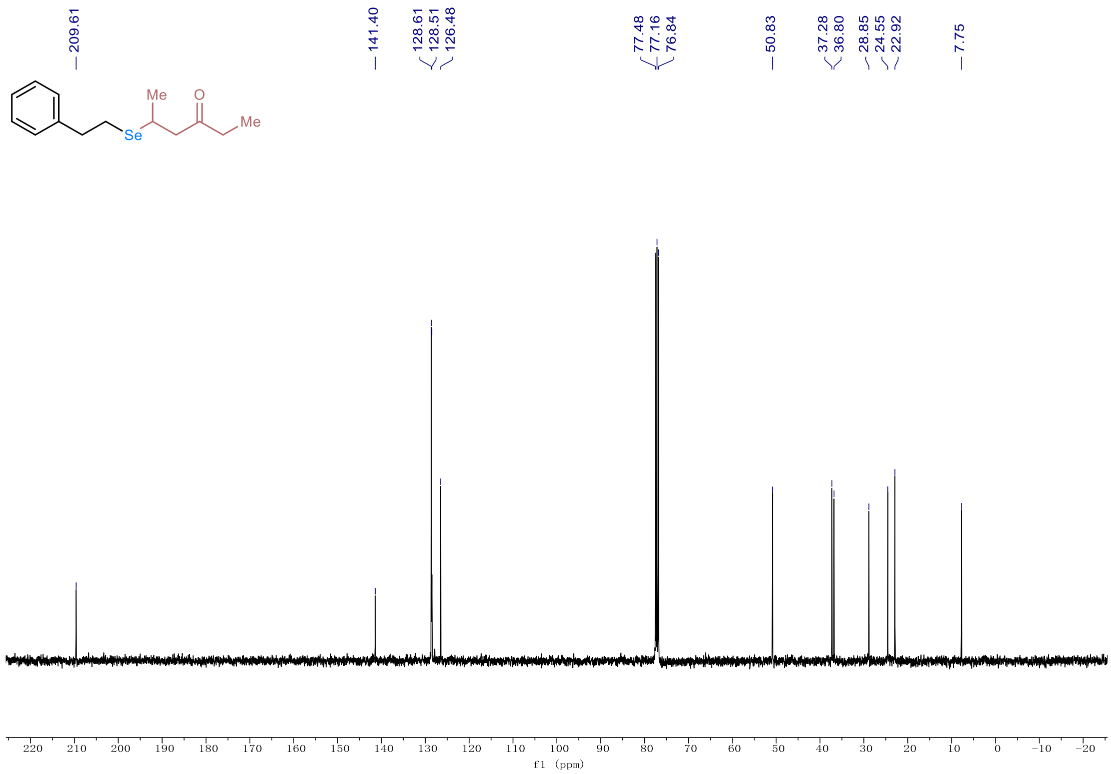

^{1}H NMR of 4k (400 MHz, Chloroform-*d*)

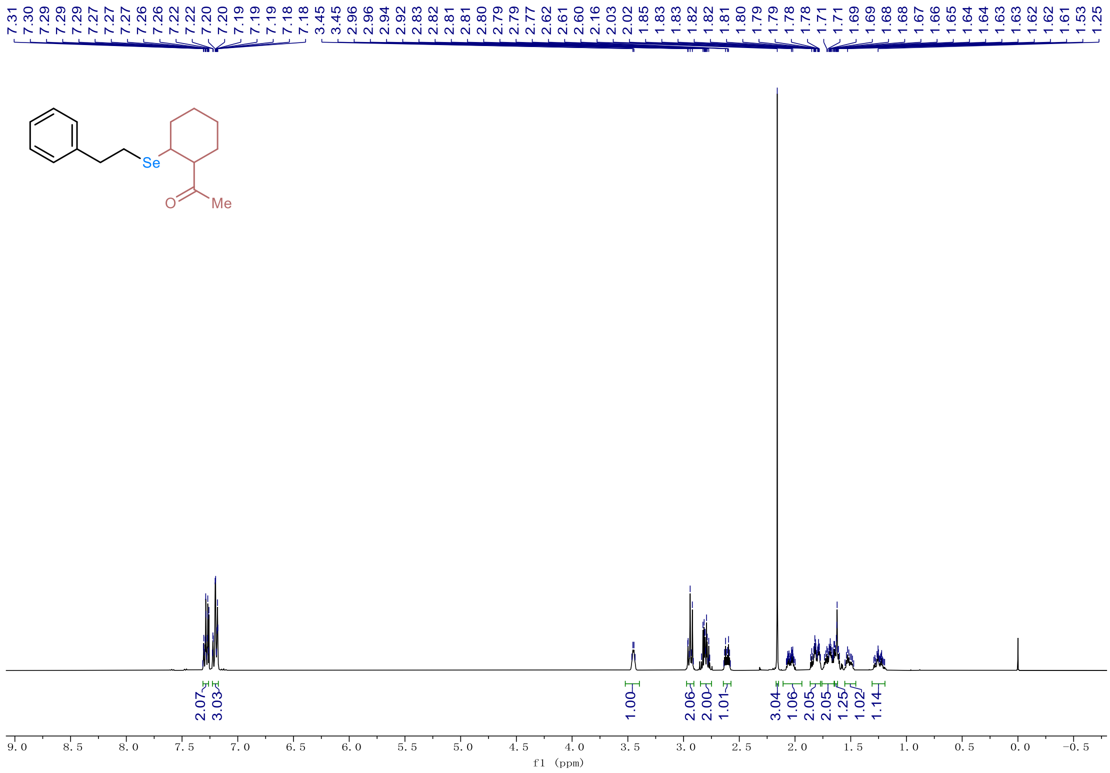

^{13}C NMR of 4k (101 MHz, Chloroform-*d*)

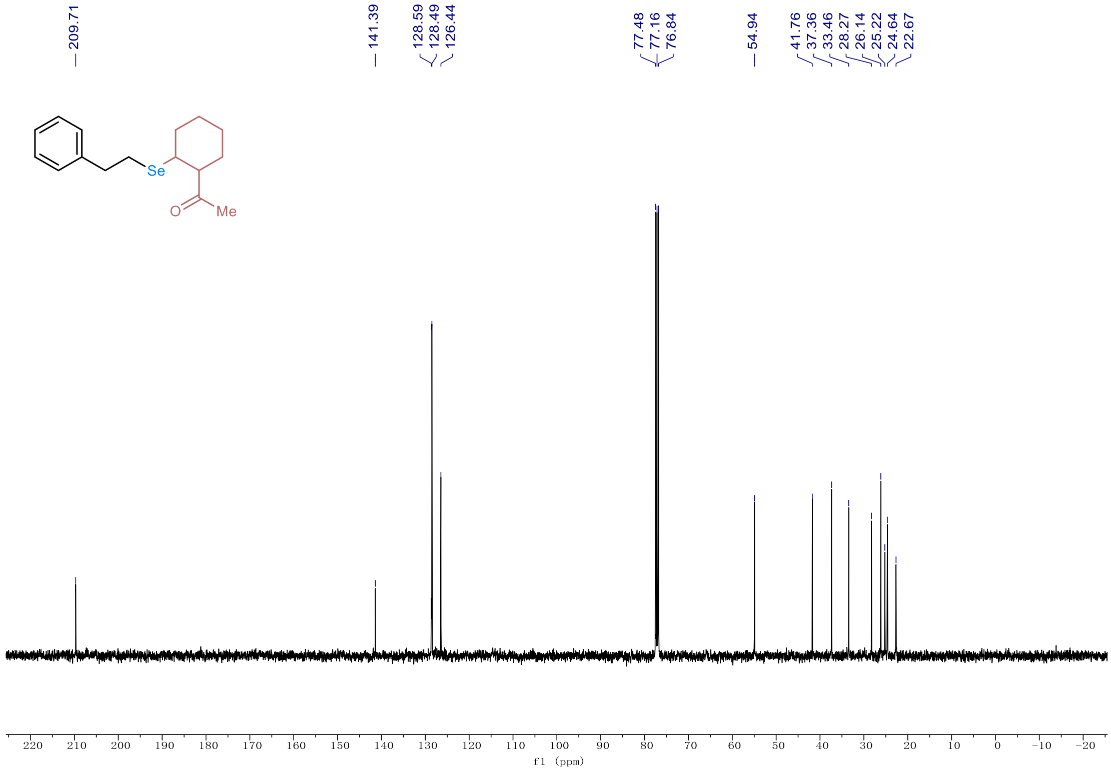

^{1}H NMR of 4l (400 MHz, Chloroform-*d*)

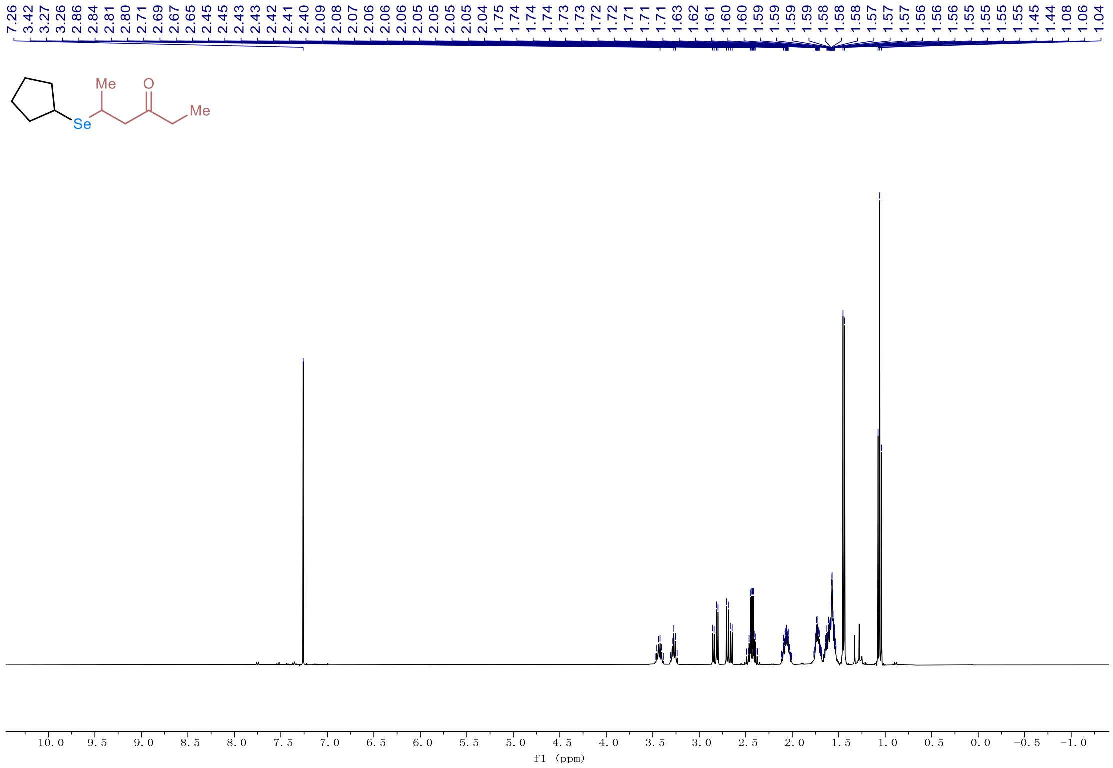

^{13}C NMR of 4l (101 MHz, Chloroform-*d*)

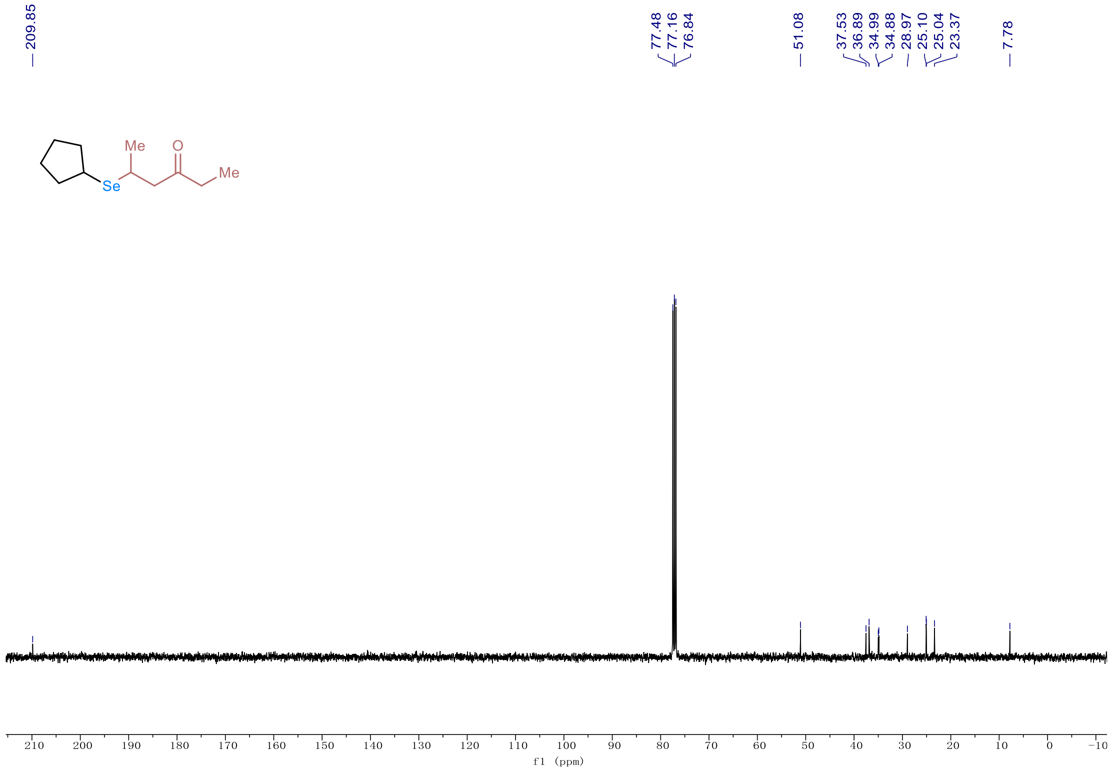

^{1}H NMR of 4m (400 MHz, Chloroform-*d*)

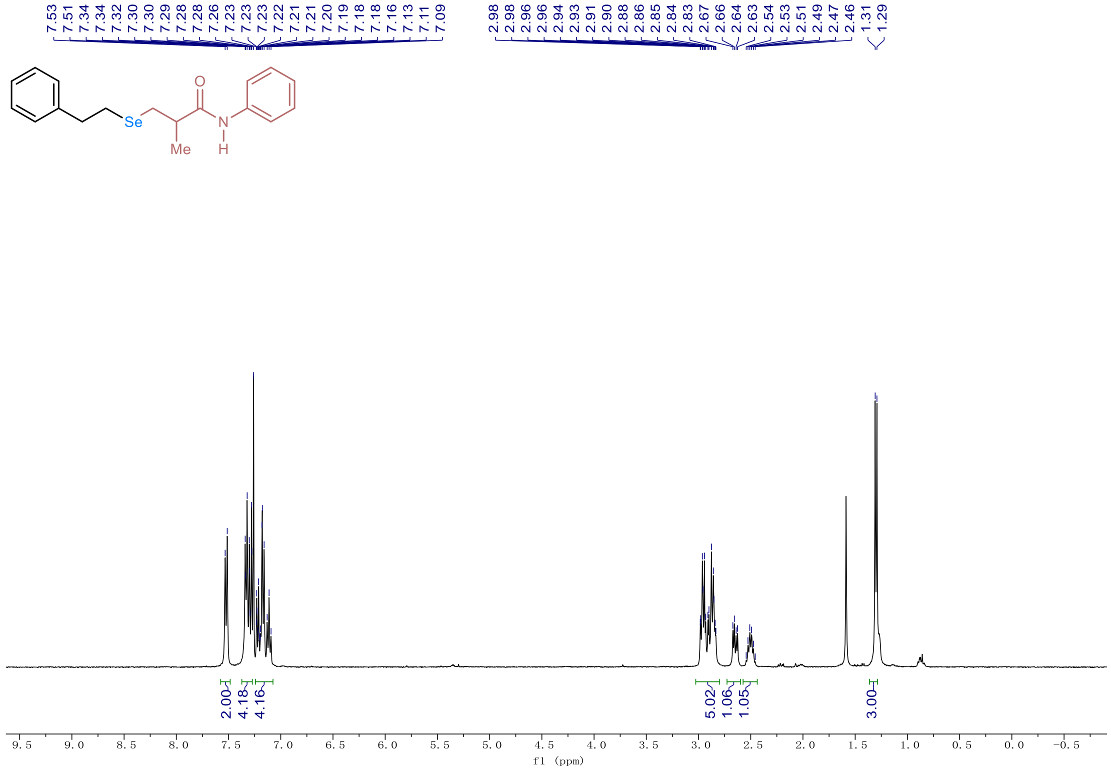

^{13}C NMR of 4m (101 MHz, Chloroform-*d*)

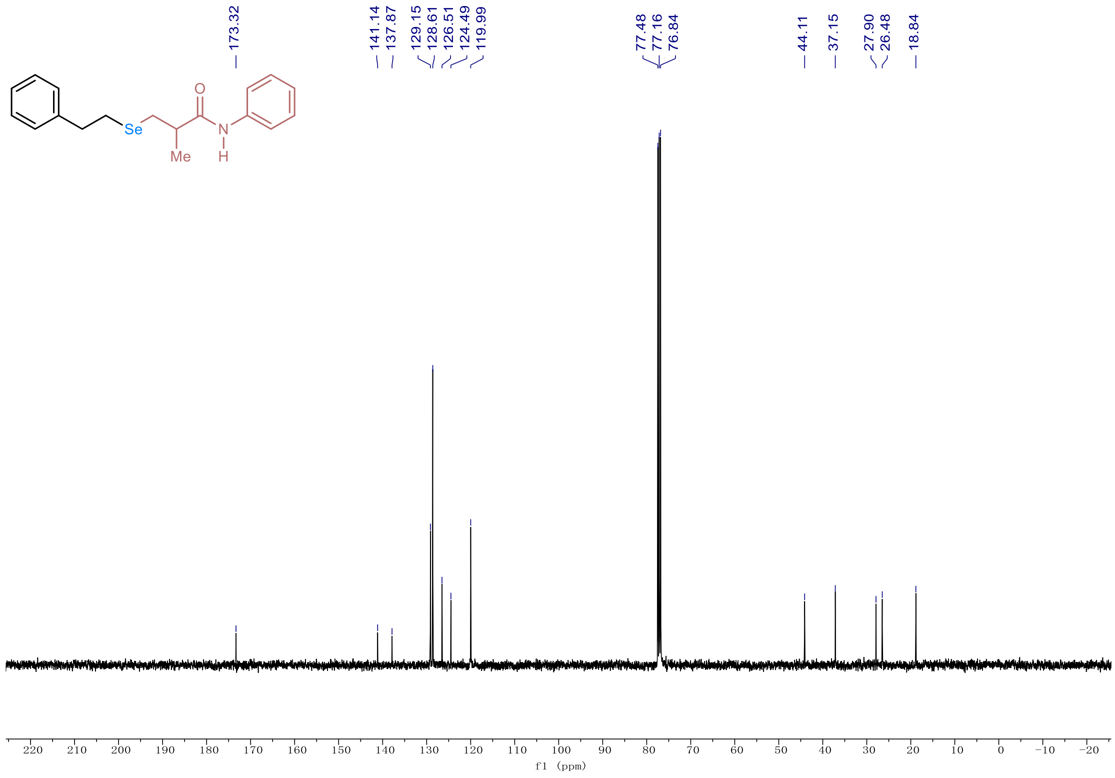

^{1}H NMR of 4n (400 MHz, Chloroform-*d*)

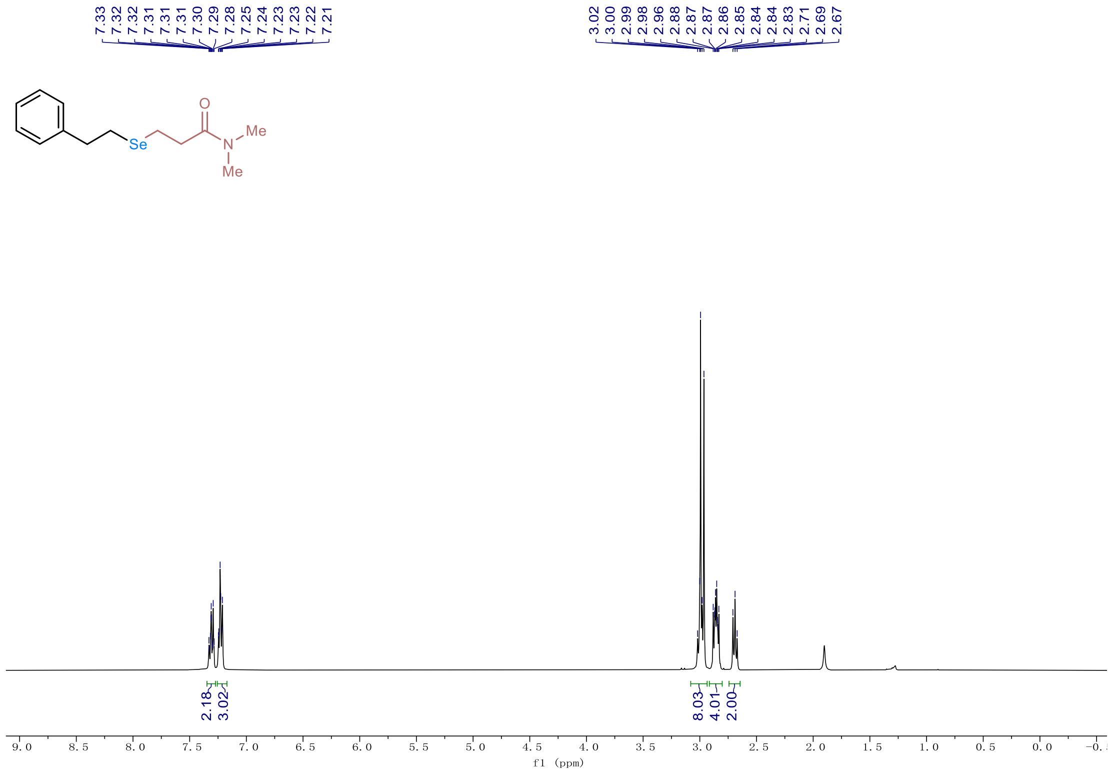

^{13}C NMR of 4n (101 MHz, Chloroform-*d*)

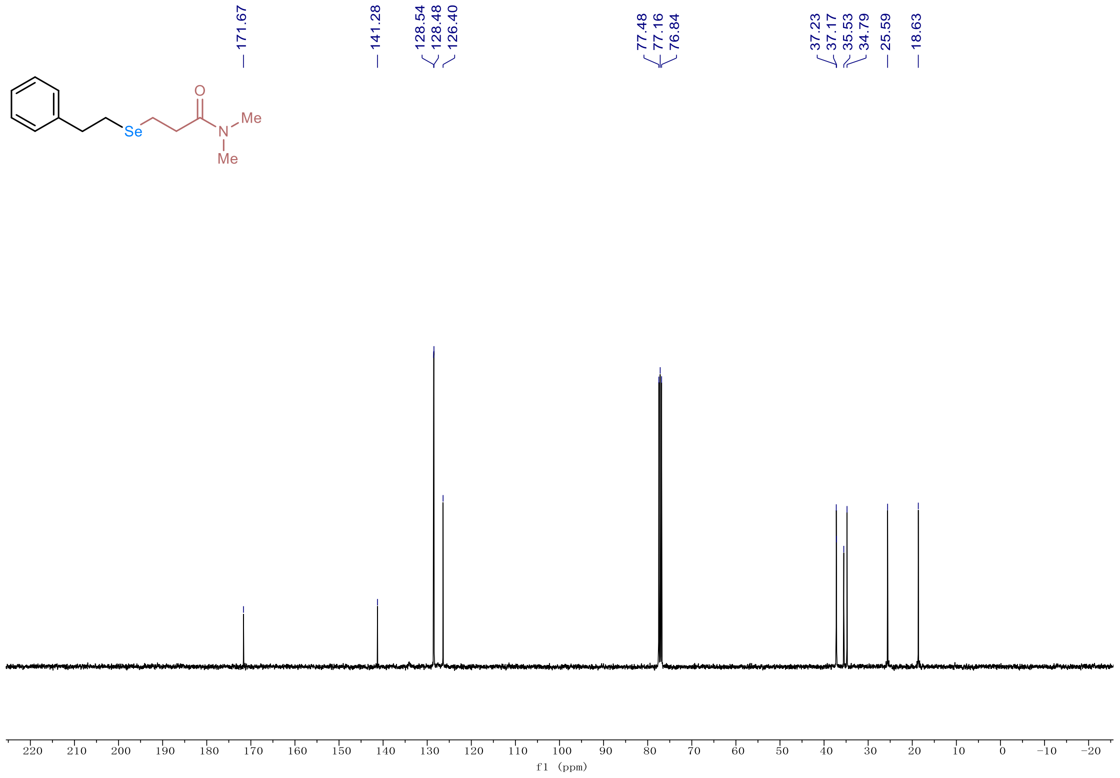

^{1}H NMR of 4o (400 MHz, Chloroform-*d*)

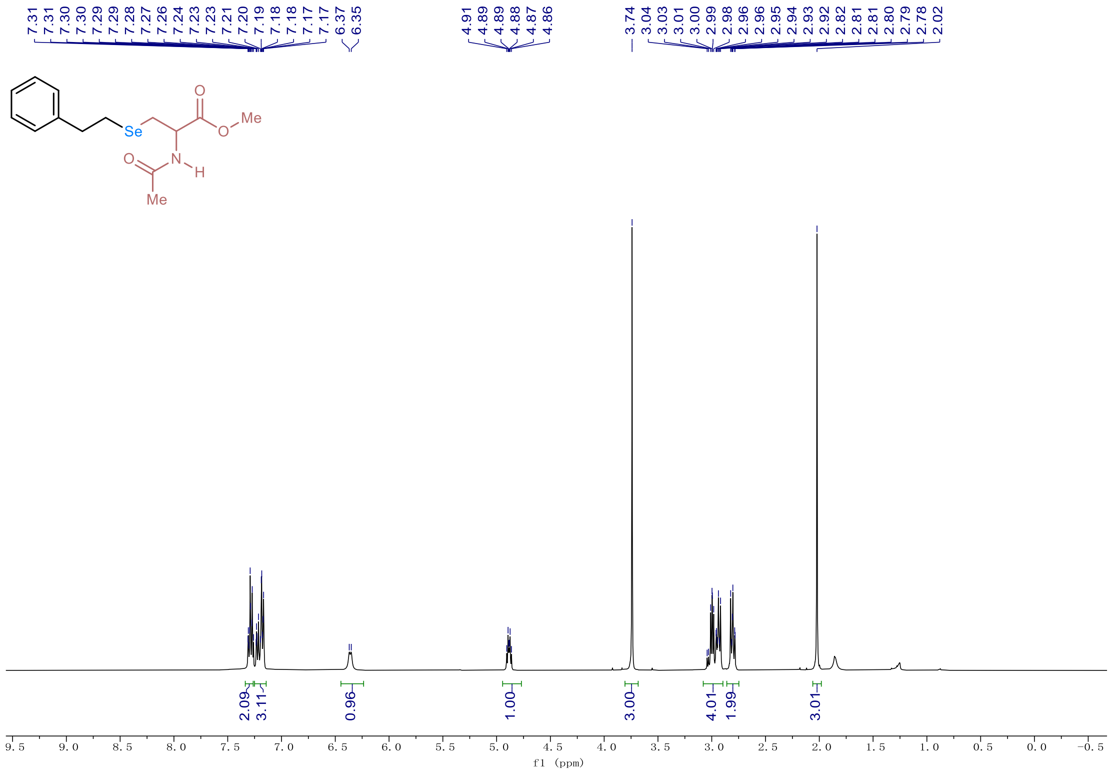

^{13}C NMR of 4o (101 MHz, Chloroform-*d*)

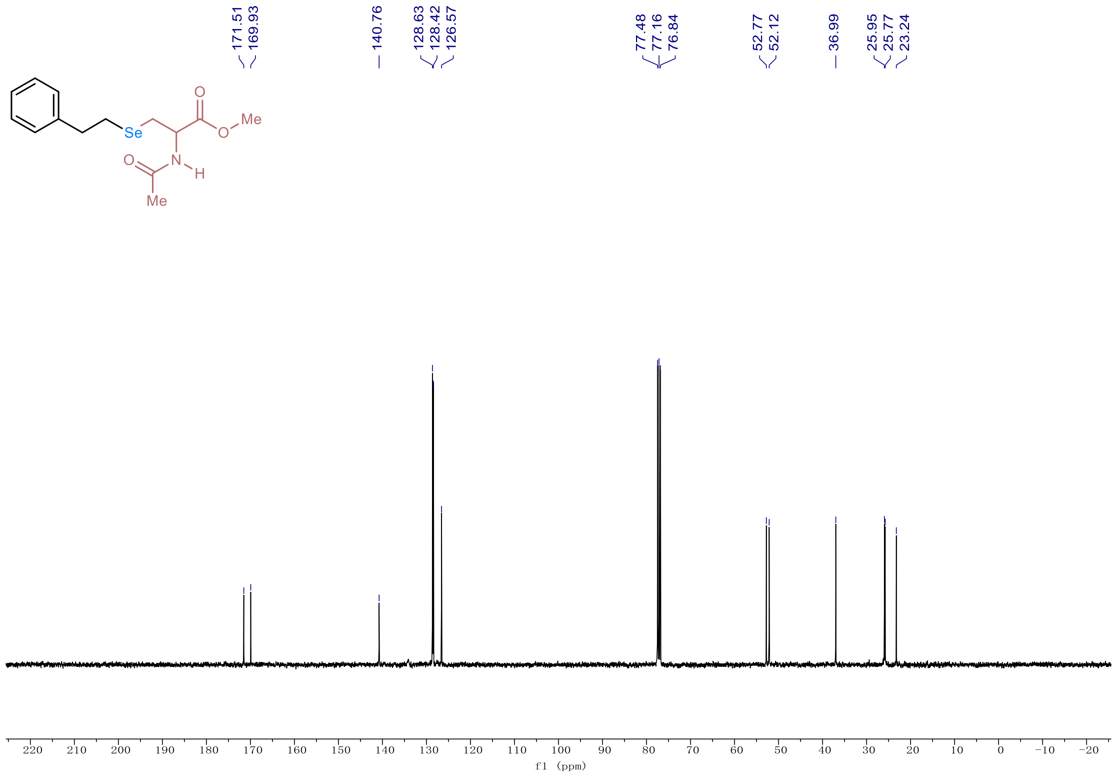

^{1}H NMR of 4p (400 MHz, Chloroform-*d*)

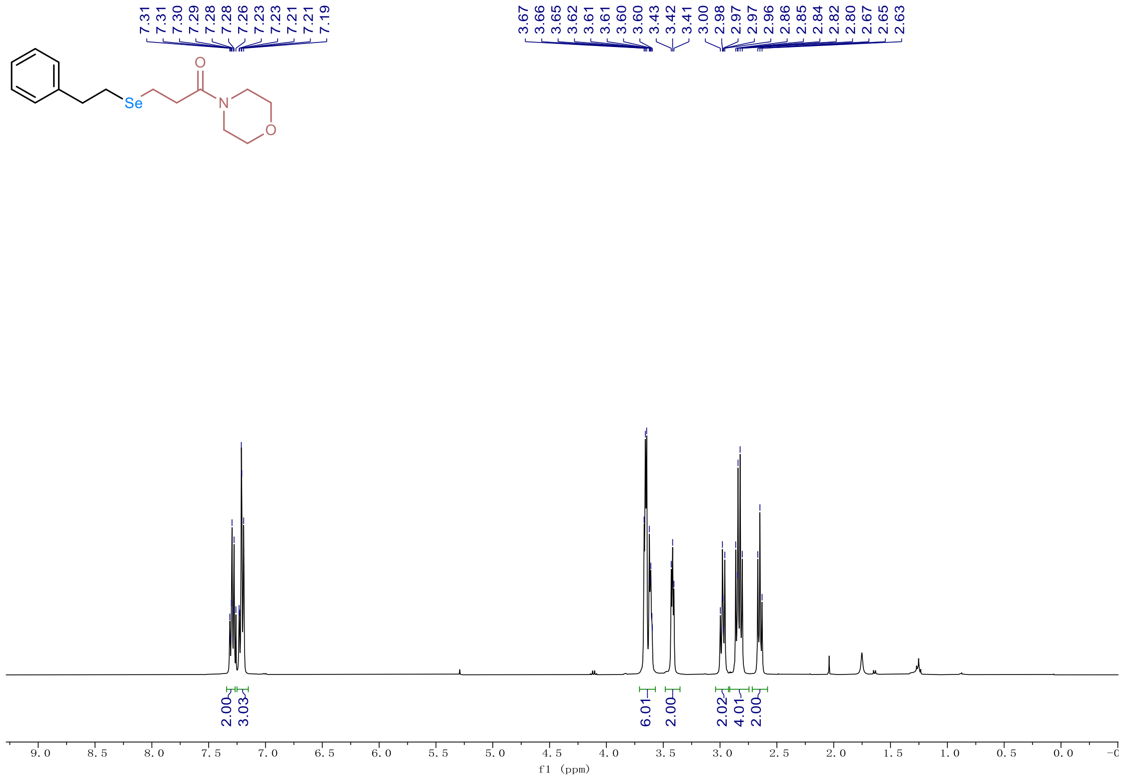

^{13}C NMR of 4p (101 MHz, Chloroform-*d*)

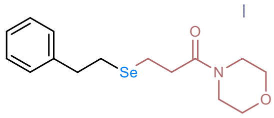

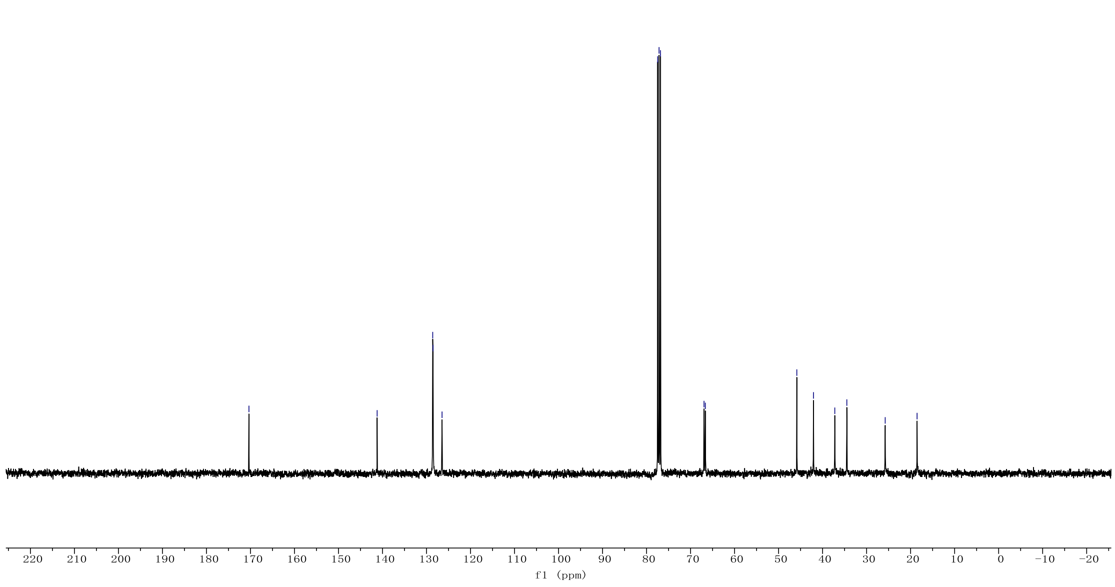

^{1}H NMR of 4q (400 MHz, Chloroform-*d*)

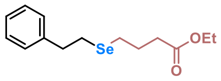

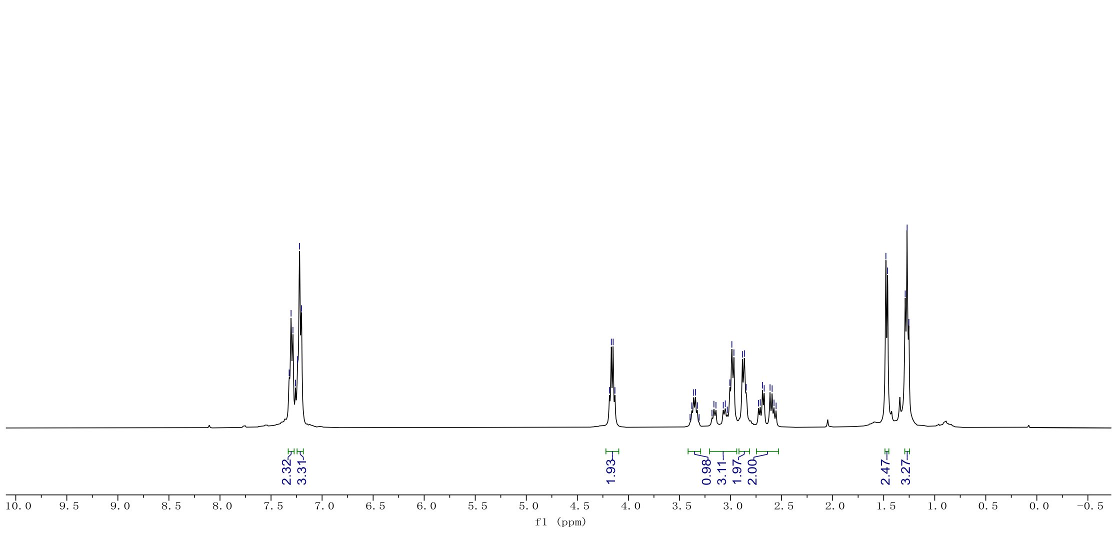

^{13}C NMR of 4q (101 MHz, Chloroform-*d*)

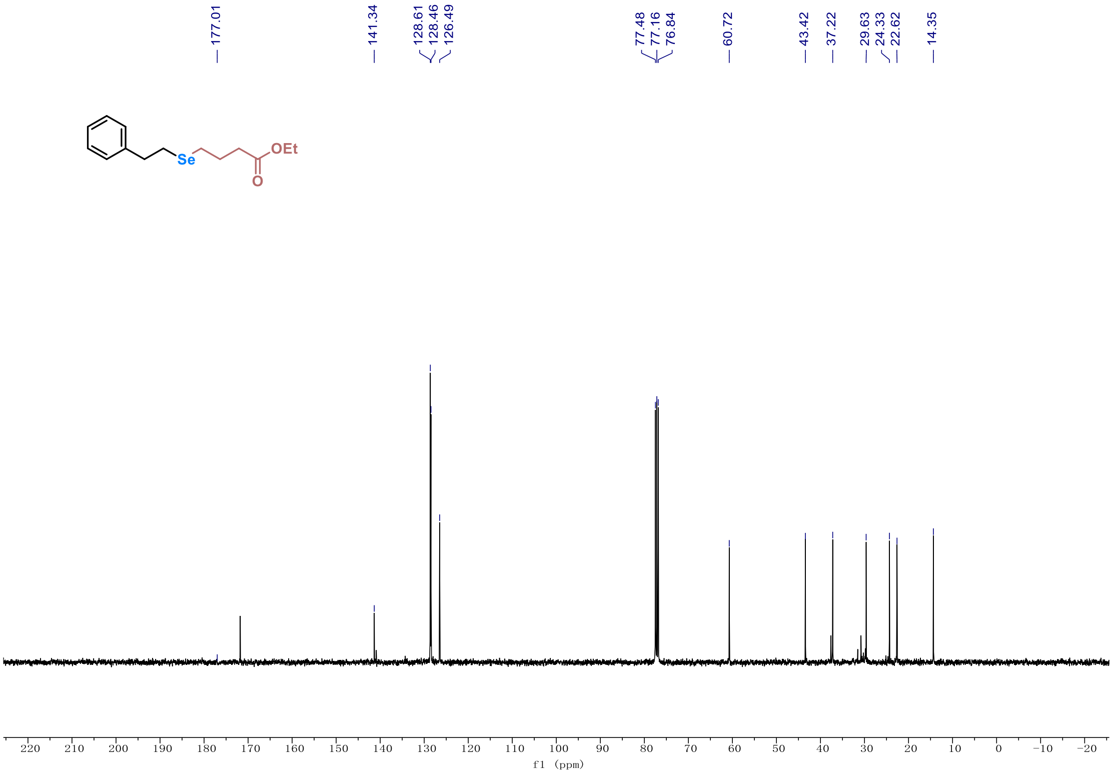

^{1}H NMR of 4r (400 MHz, Chloroform-*d*)

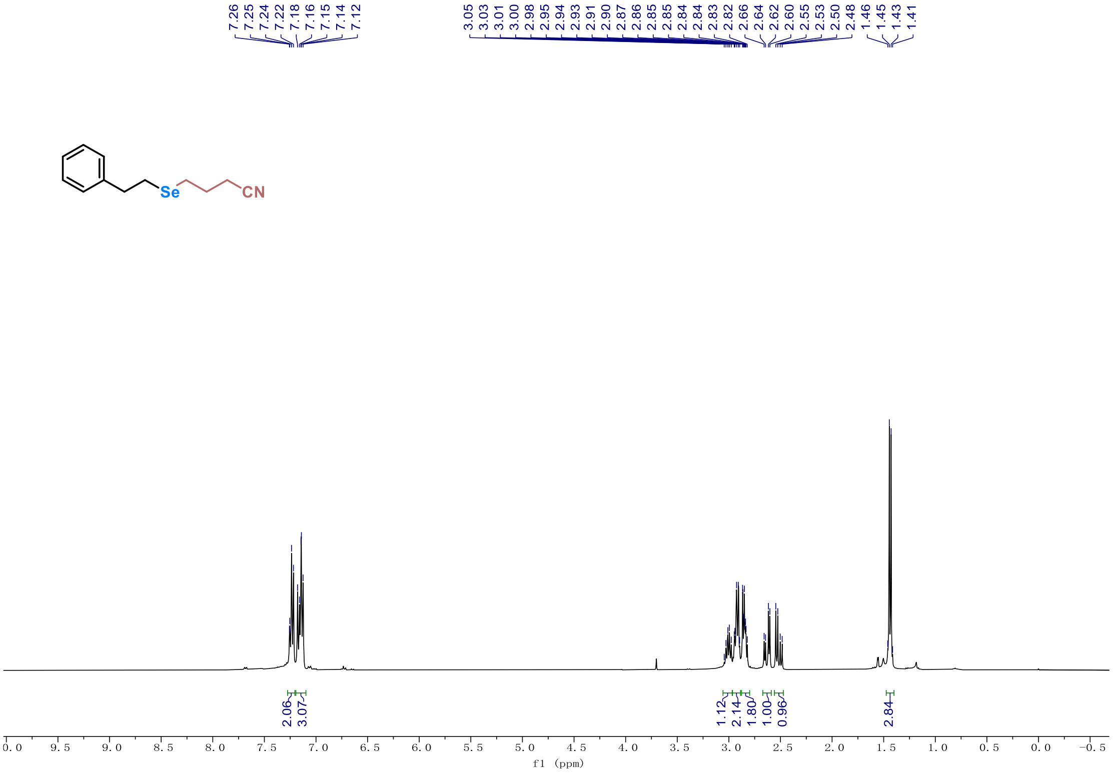

^{13}C NMR of 4r (101 MHz, Chloroform-*d*)

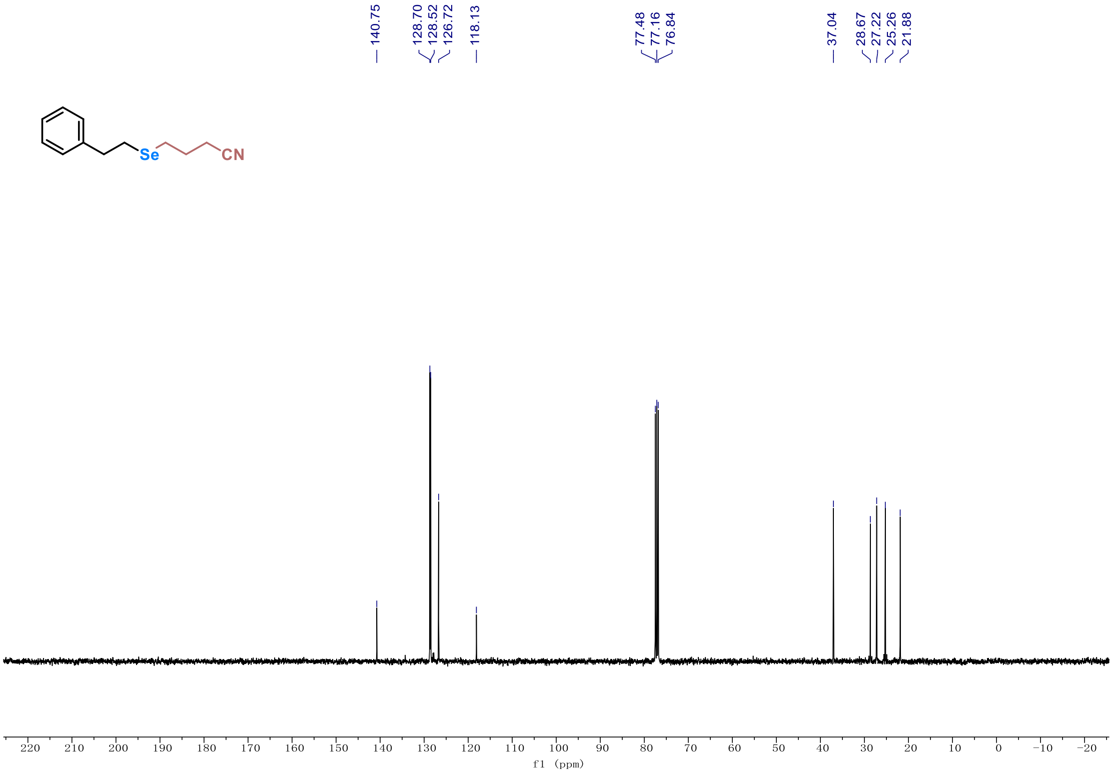

^{1}H NMR of 4s (400 MHz, Chloroform-*d*)

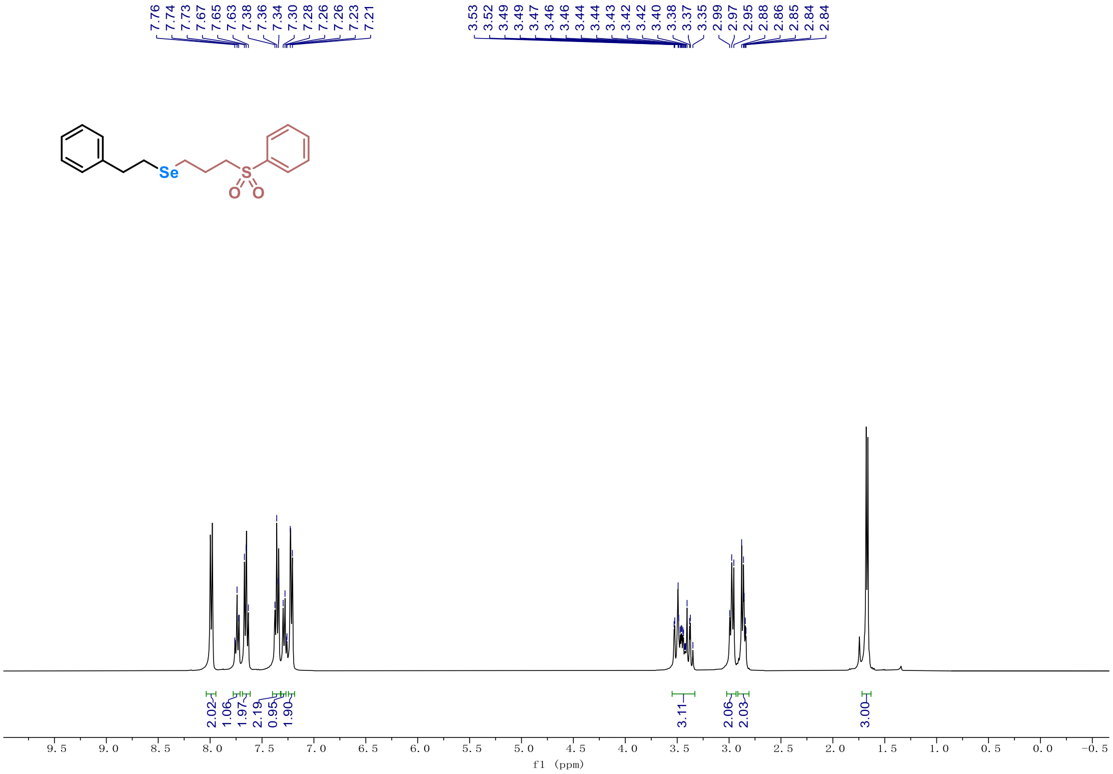

^{13}C NMR of 4s (101 MHz, Chloroform-*d*)

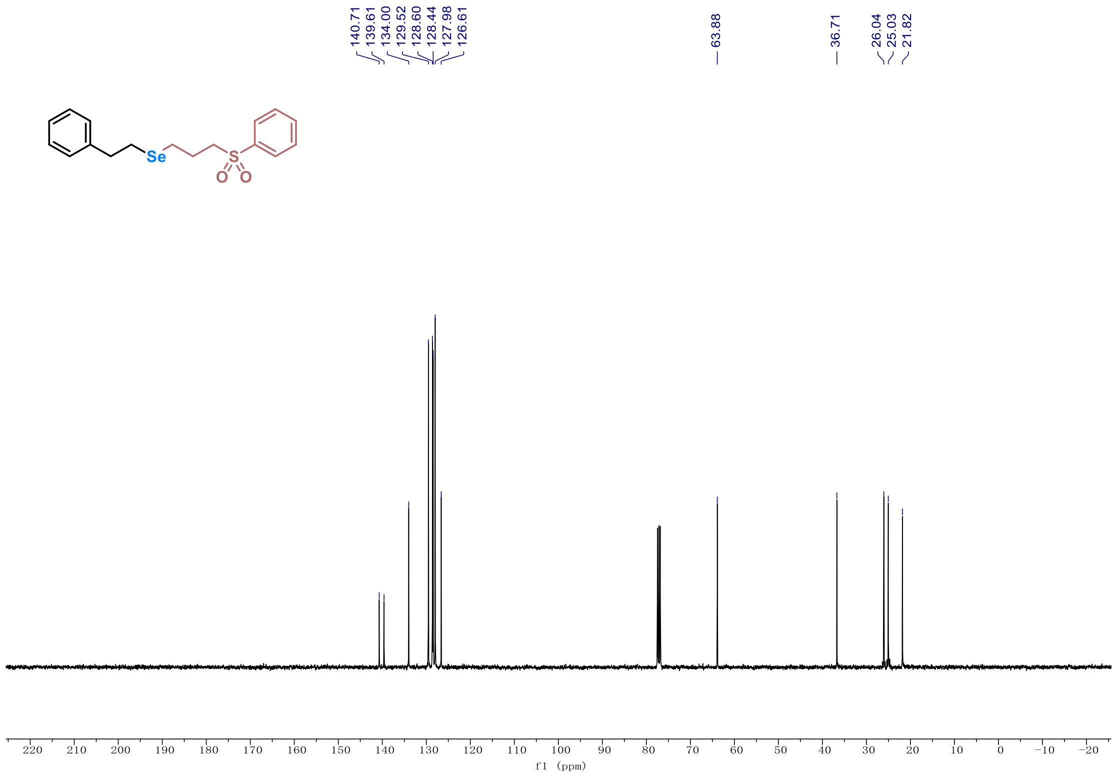

^{1}H NMR of 4t (400 MHz, Chloroform-*d*)

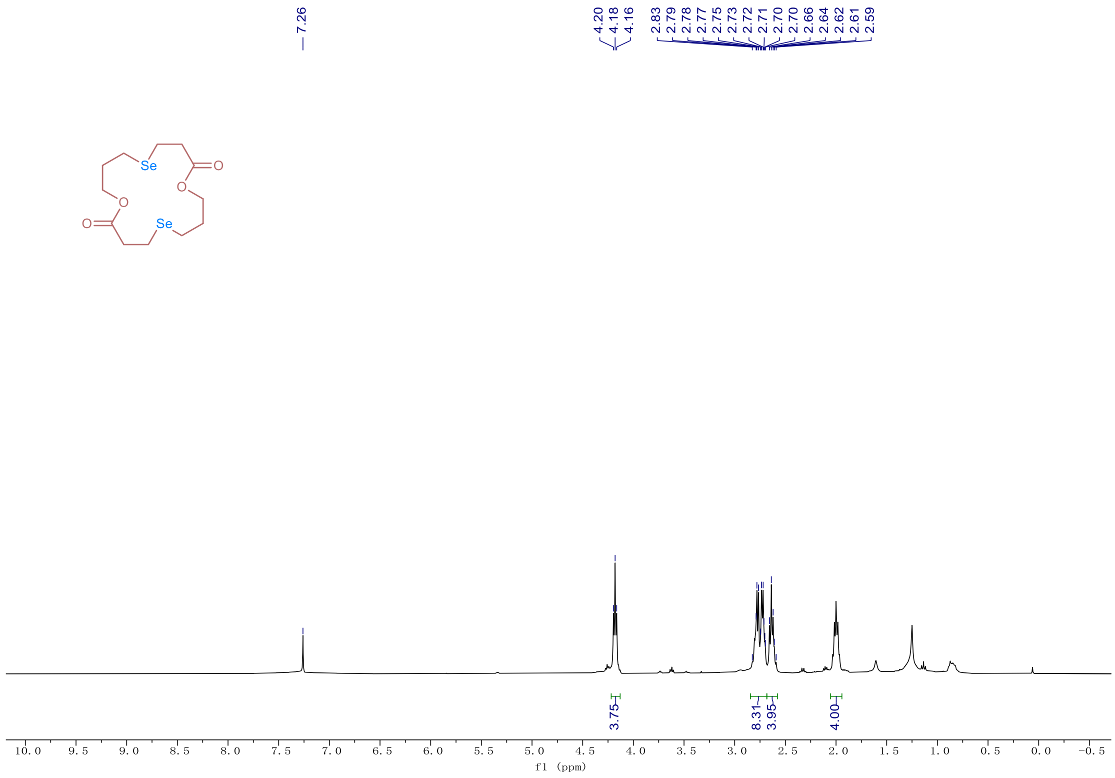

^{13}C NMR of 4t (101 MHz, Chloroform-*d*)

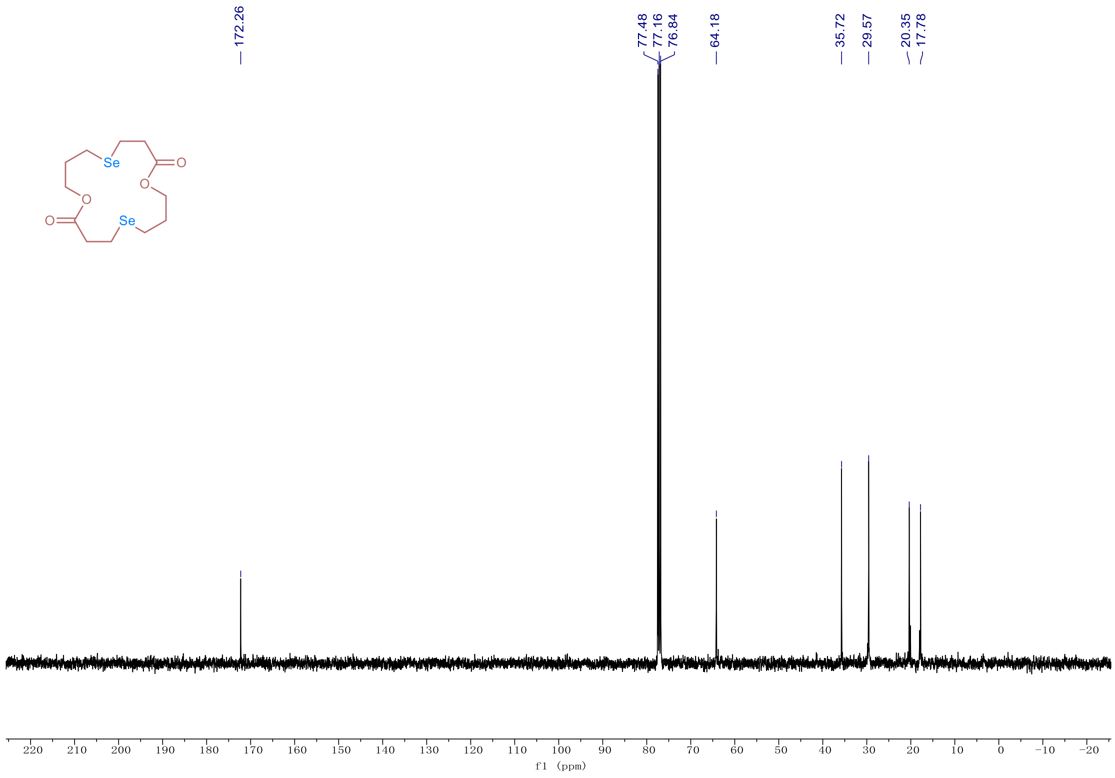

^{1}H NMR of 4u (400 MHz, Chloroform-*d*)

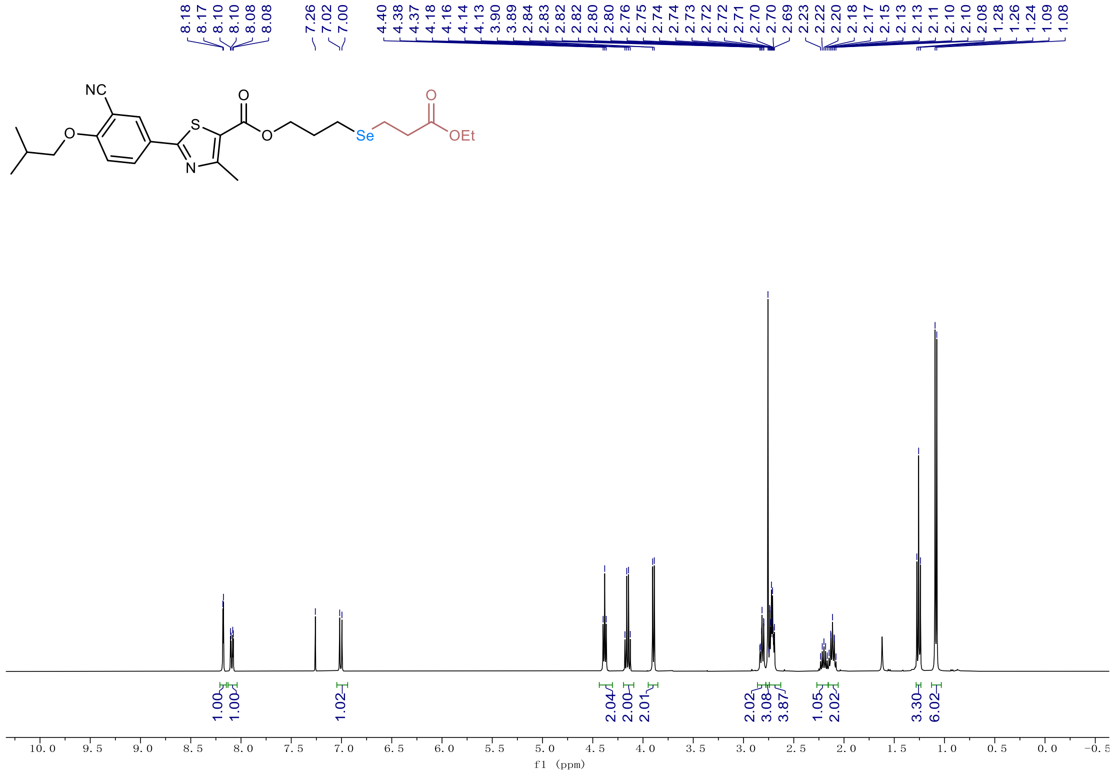

^{13}C NMR of 4u (101 MHz, Chloroform-*d*)

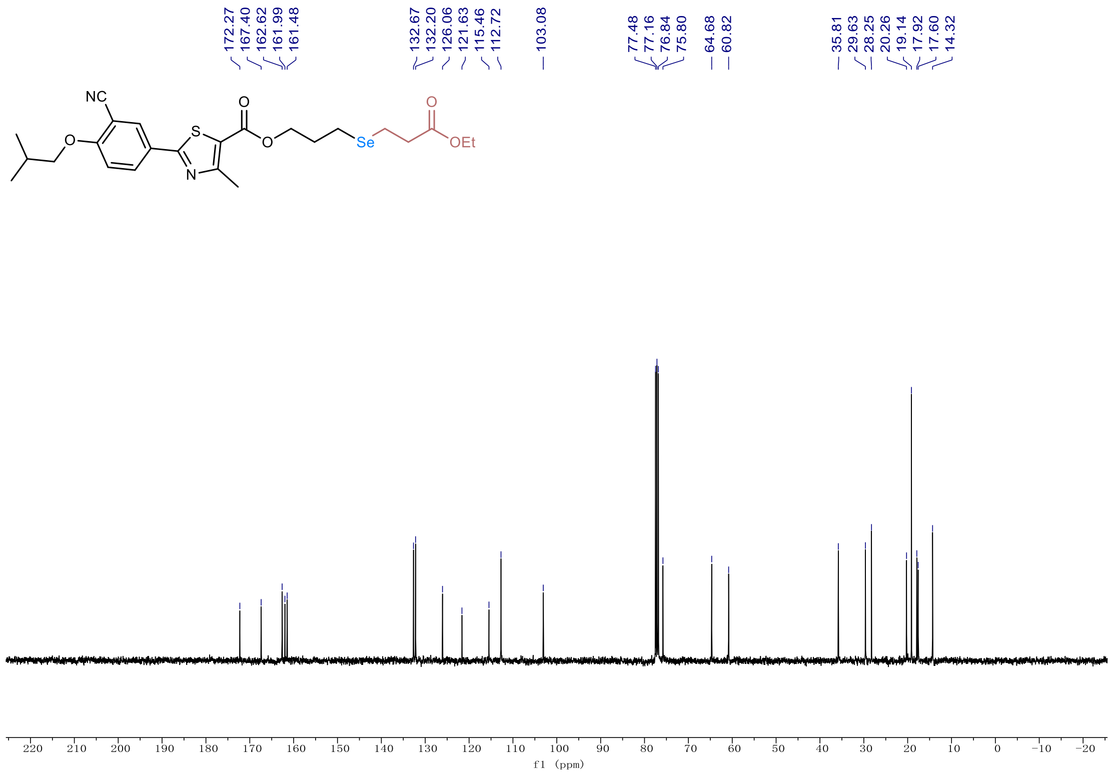

^{1}H NMR of 4v (400 MHz, Chloroform-*d*)

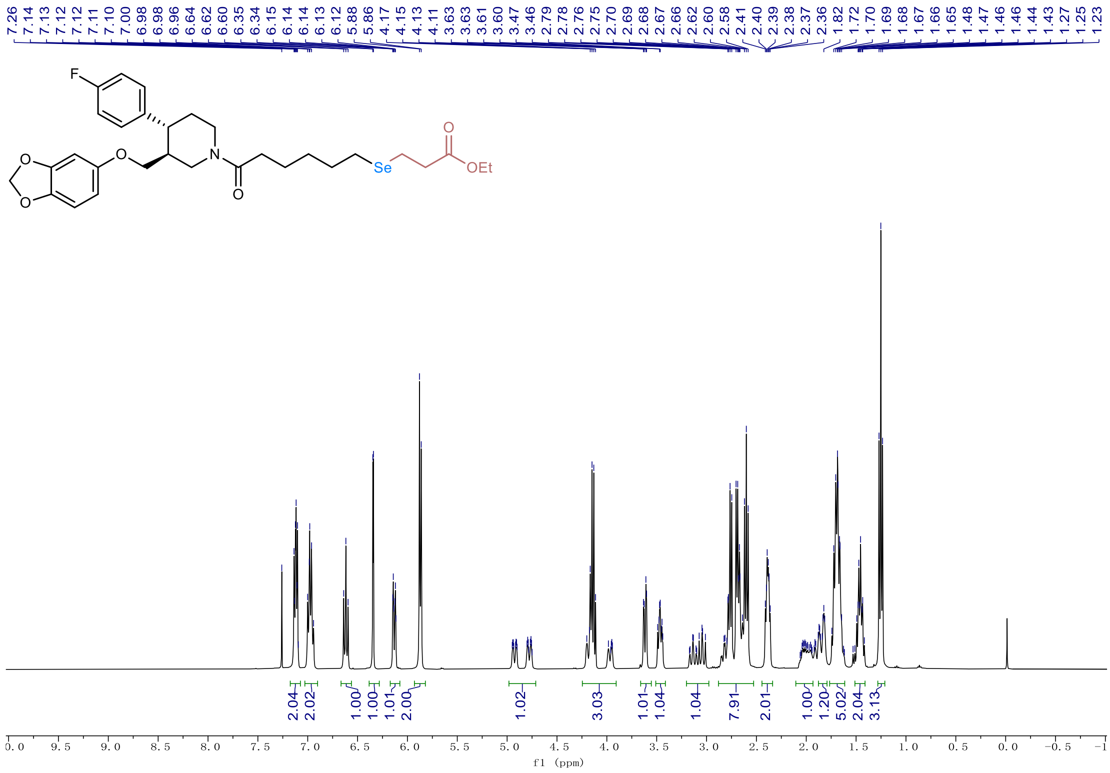

^{13}C NMR of 4v (101 MHz, Chloroform-*d*)

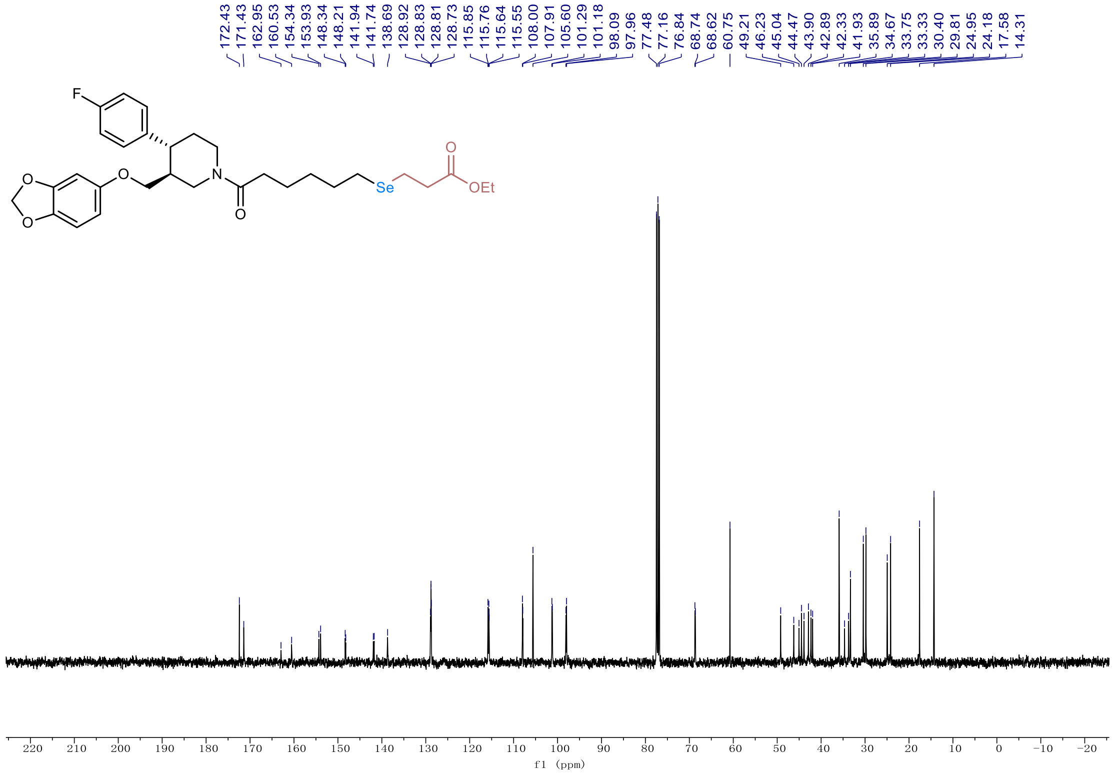

^{19}F NMR of 4v (376 MHz, Chloroform-*d*)

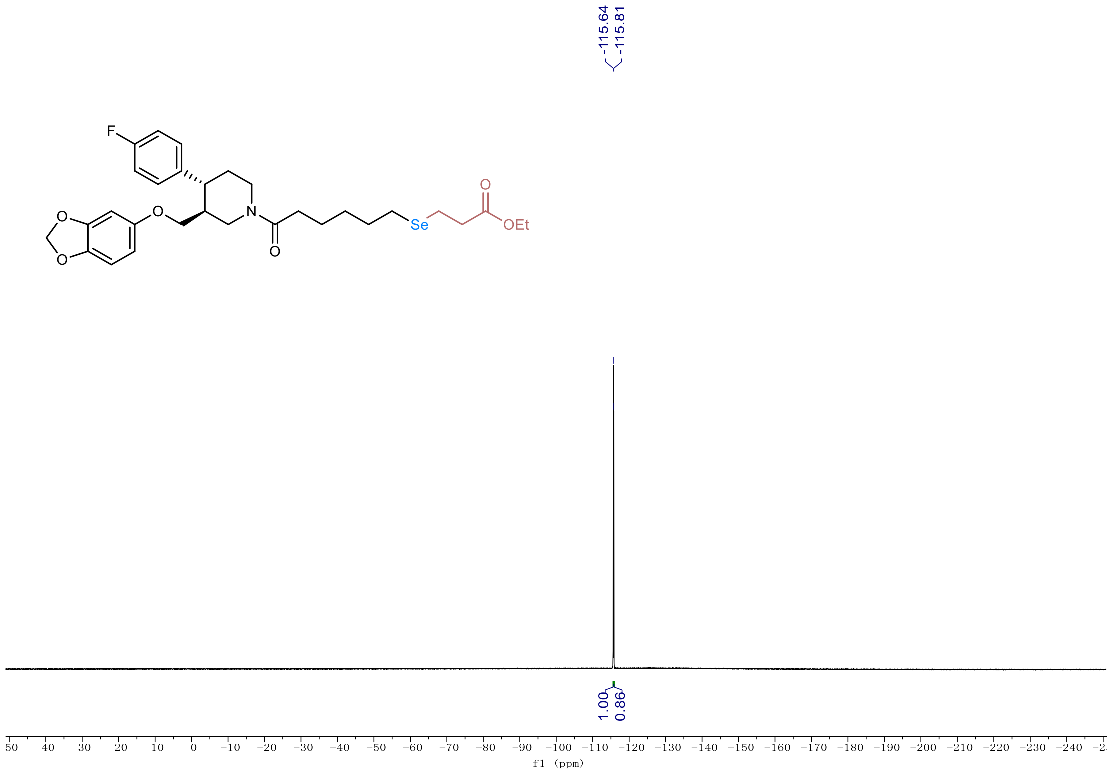

^{1}H NMR of 4w (400 MHz, Chloroform-*d*)

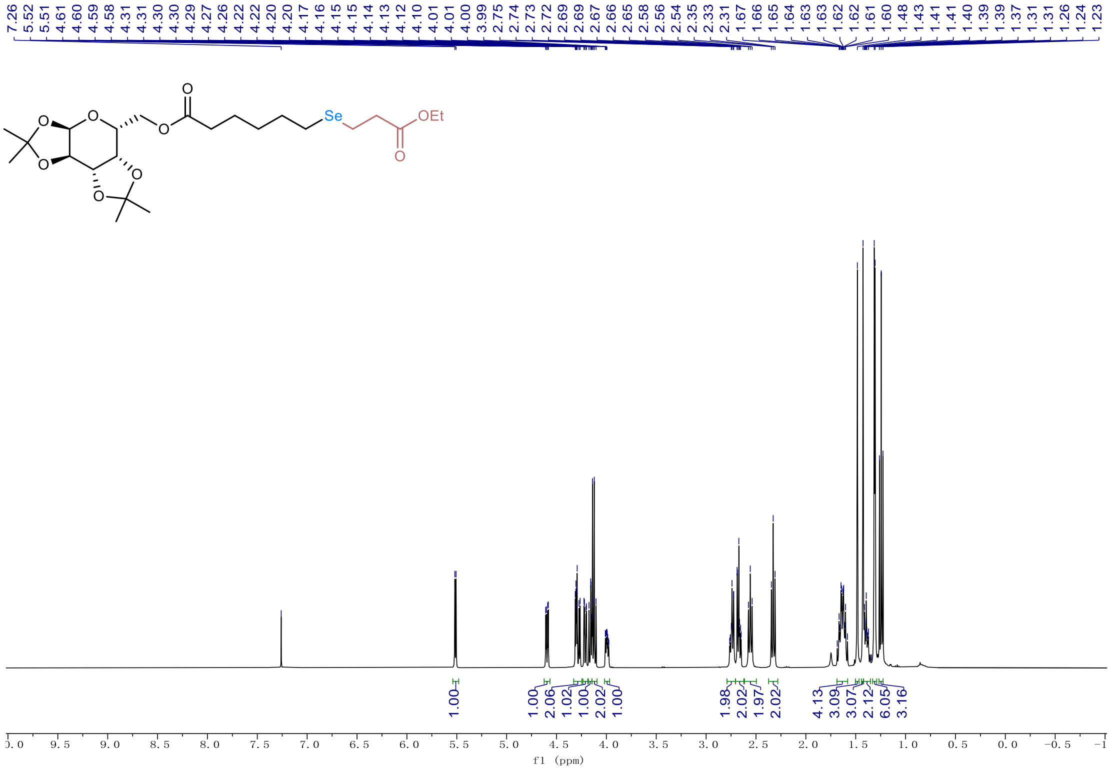

^{13}C NMR of 4w (101 MHz, Chloroform-*d*)

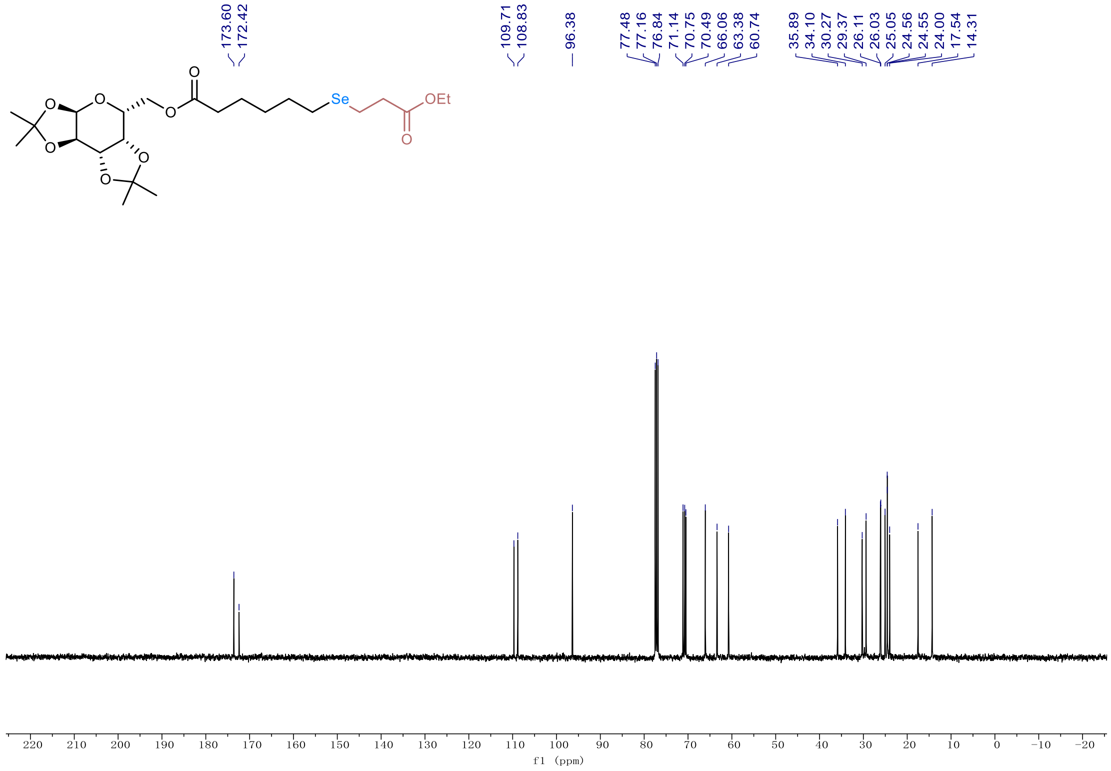

^{1}H NMR of 4x (400 MHz, Chloroform-*d*)

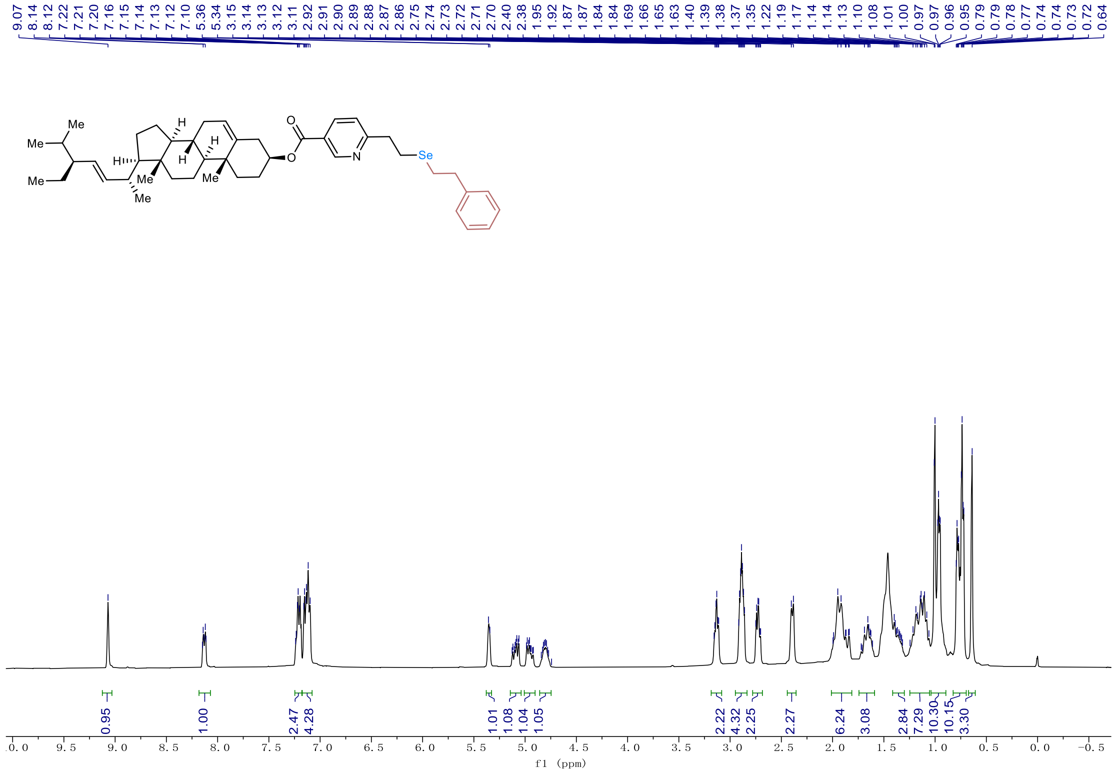

^{13}C NMR of 4x (101 MHz, Chloroform-*d*)

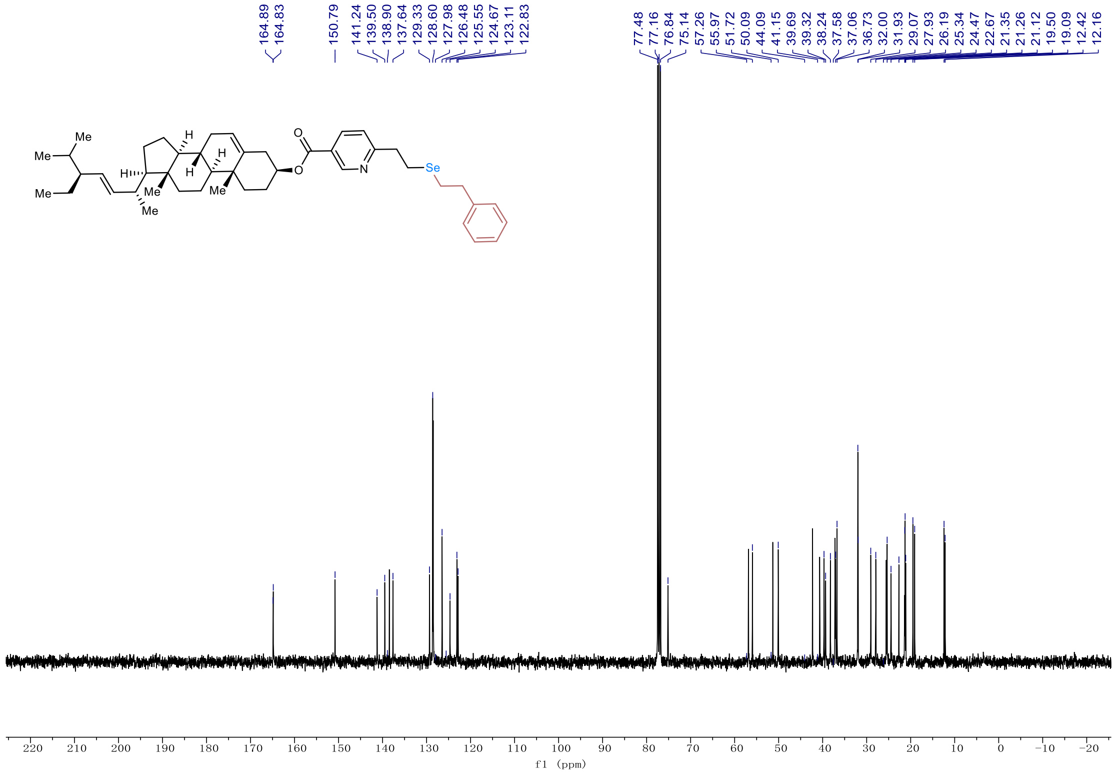

^{1}H NMR of 4y (400 MHz, Chloroform-*d*)

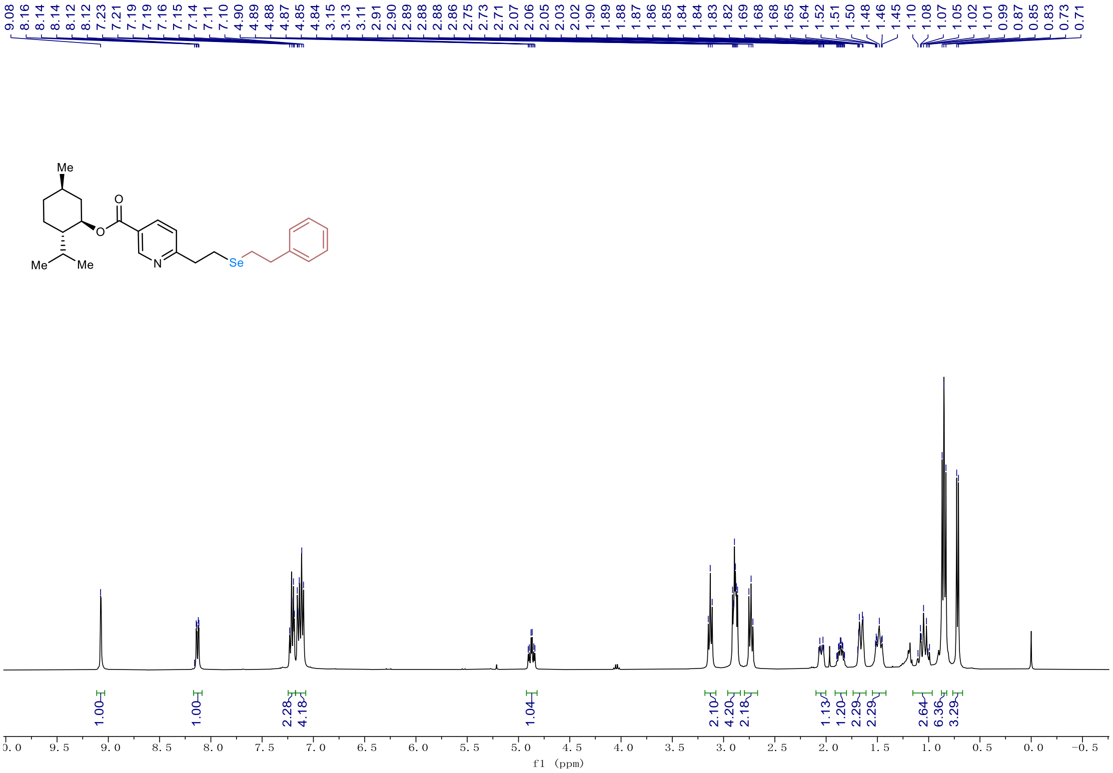

^{13}C NMR of 4y (101 MHz, Chloroform-*d*)

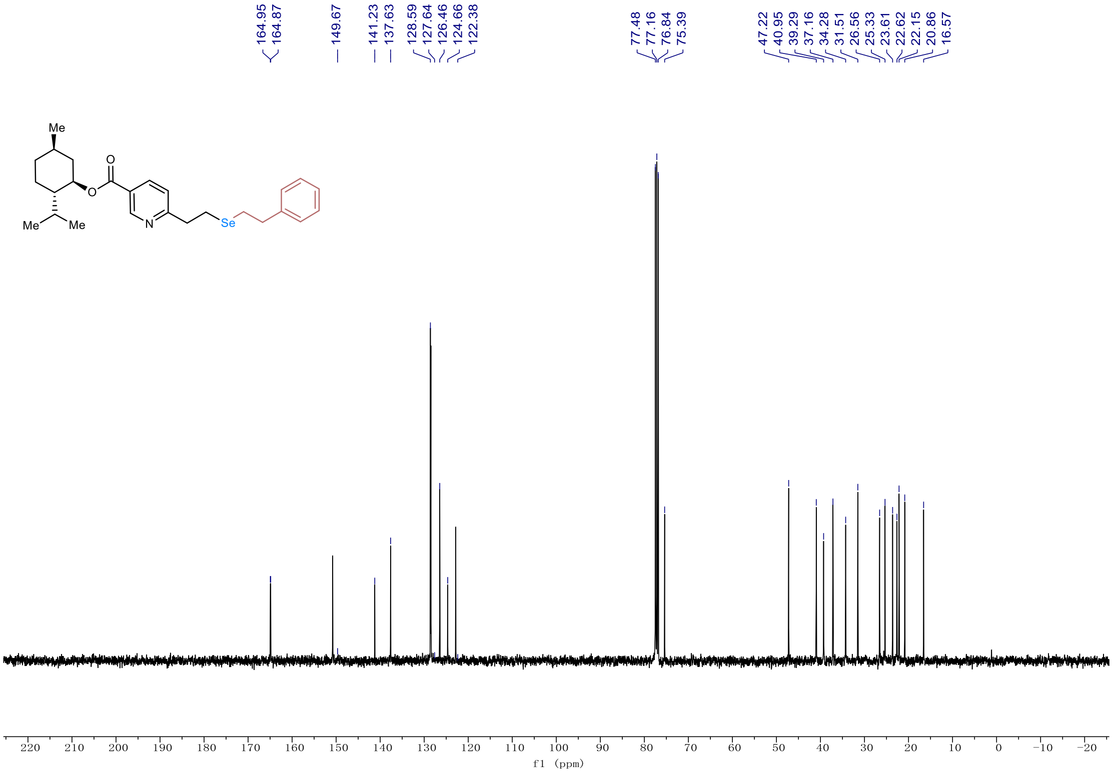

^{1}H NMR of 4z (400 MHz, Chloroform-*d*)

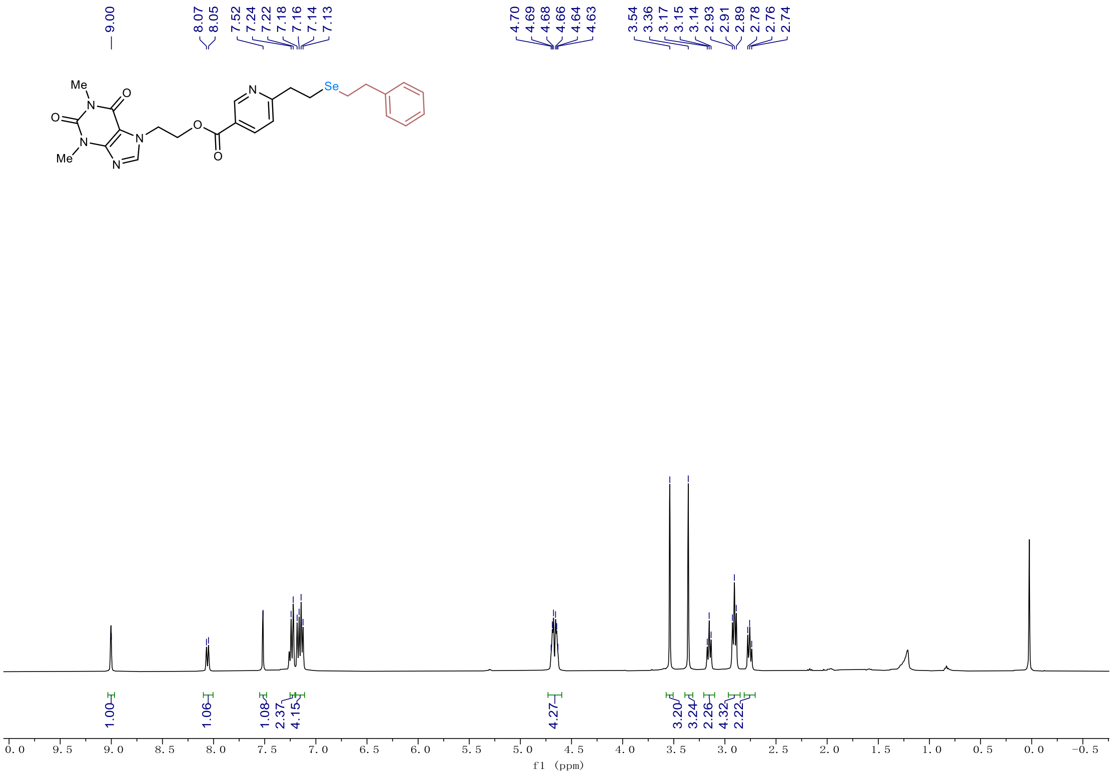

^{13}C NMR of 4z (101 MHz, Chloroform-*d*)

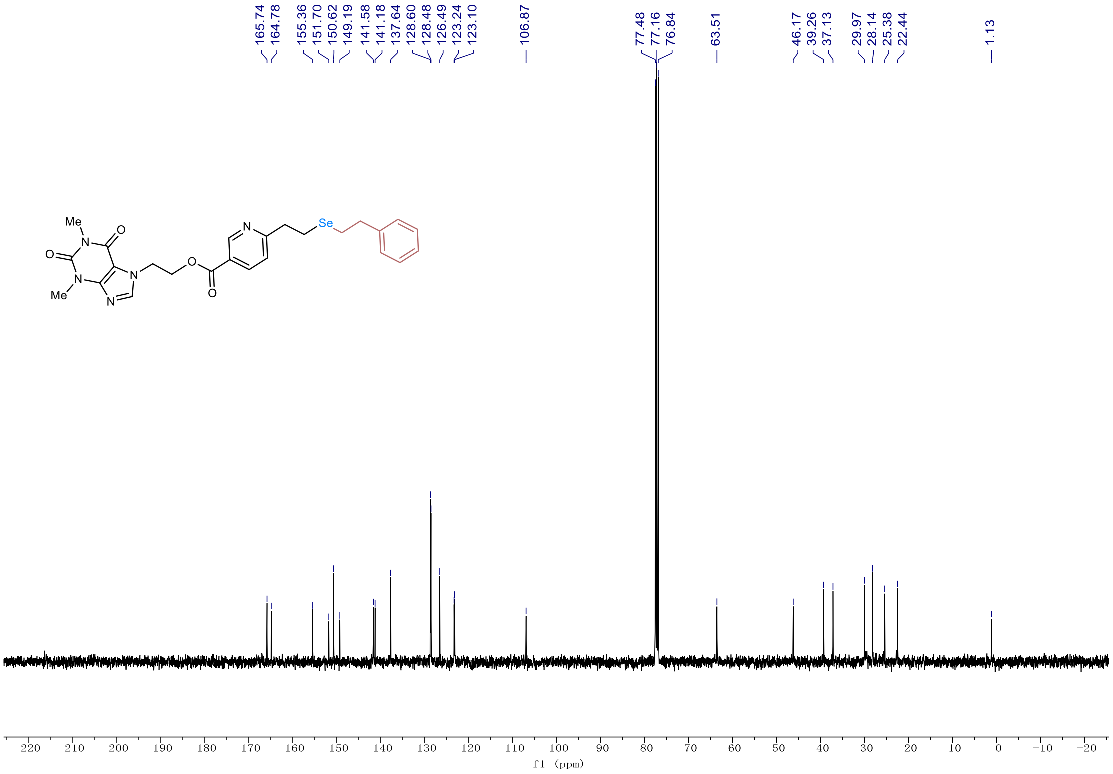

^{1}H NMR of 6 (400 MHz, Chloroform-*d*)

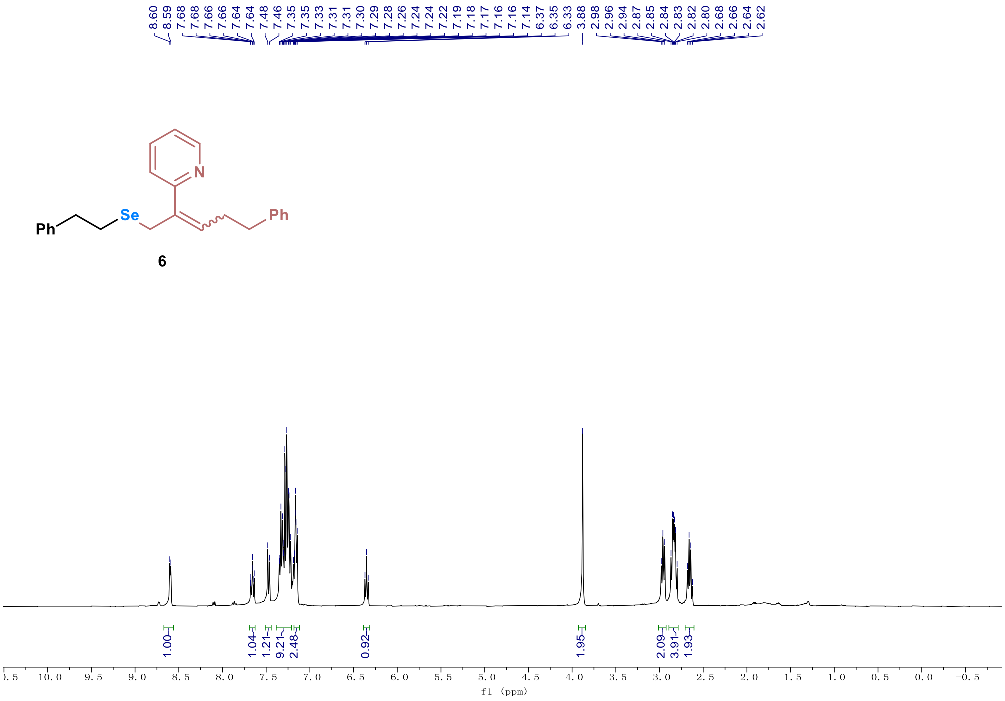

^{13}C NMR of 6 (101 MHz, Chloroform-*d*)

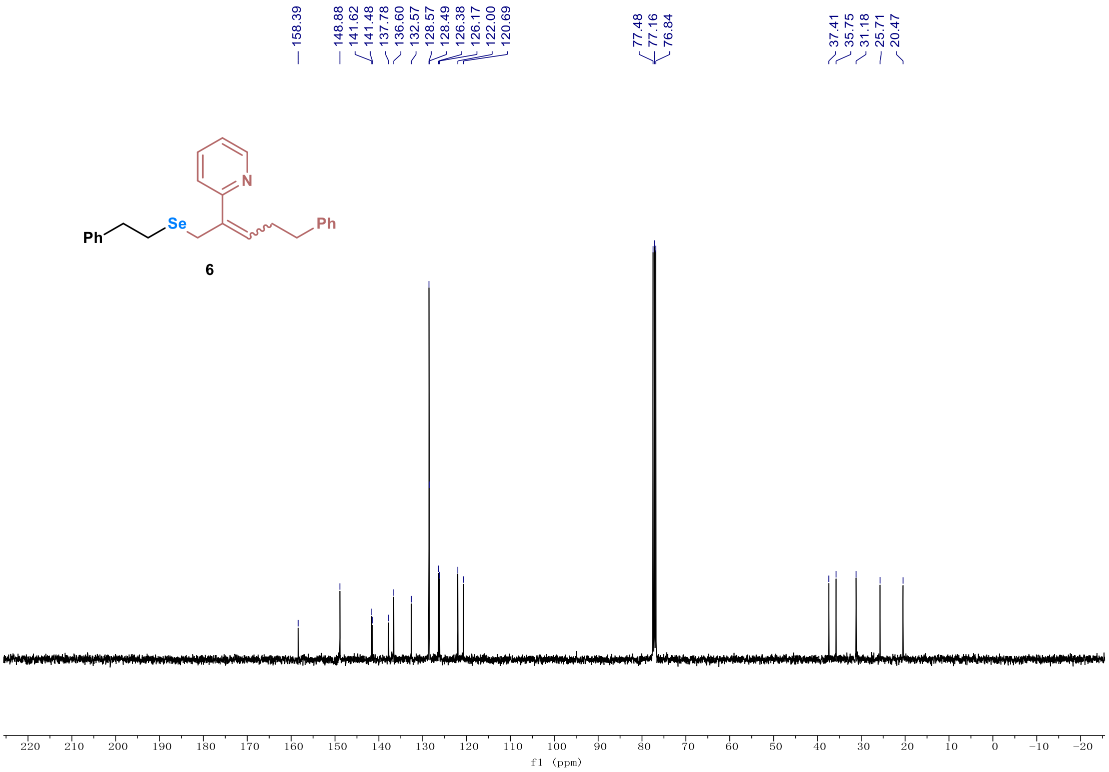

Supplement: Supplementary file 1 — Supporting File: advs74730‐sup‐0001‐SuppMat.pdf [file ADVS-13-e22609-s001.pdf]
